# Similar Binding Mode of a 5‑Sulfonylthiouracil Derivative Antagonist at Chemerin Receptors CMKLR1 and GPR1

**DOI:** 10.1021/acs.jmedchem.5c00135

**Published:** 2025-05-16

**Authors:** Tina Schermeng, Alexander Fürll, Fabian Liessmann, Lukas von Bredow, Jan Stichel, C. David Weaver, Maik Tretbar, Jens Meiler, Annette G. Beck-Sickinger

**Affiliations:** a Institute of Biochemistry, 9180 Leipzig University, Leipzig 04103, Germany; b Institute for Drug Discovery, 9180 Leipzig University, Leipzig 04103, Germany; c Center for Scalable Data Analytics and Artificial Intelligence ScaDS.AI and School of Embedded Composite Artificial Intelligence SECAI, Leipzig 04105, Germany; d Department of Chemistry, Department of Pharmacology and Institute of Chemical Biology, 5718 Vanderbilt University, Nashville, Tennessee 37235, United States

## Abstract

Several studies have linked chemerin/chemokine-like receptor 1 (CMKLR1) to inflammation, leukocyte recruitment, and obesity. Reduced cellular activation may reduce inflammation in adipose tissues. High-throughput screening identified a novel antagonist (VU0514009), which was optimized to compound **16** as a full and competitive antagonist (IC_50_ = 37 μM). Mutagenesis studies elucidated relevant interactions of compound **16** at CMKLR1 residues Y6.51 and L7.35 as well as F7.31, S7.32, and T7.39 forming the binding pocket. Based on active CMKLR1/chemerin-9 structures and the inactive AlphaFold model, in silico docking was performed in the inactive model, with compound **16** most likely binding orthosterically. Considering the sequence similarity of CMKLR1 and GPR1, compound **16** was docked to GPR1, indicating a similar binding. At GPR1, compound **16** showed a slightly lower effect on chemerin-9-mediated arrestin recruitment and internalization.

## Graphical abstract

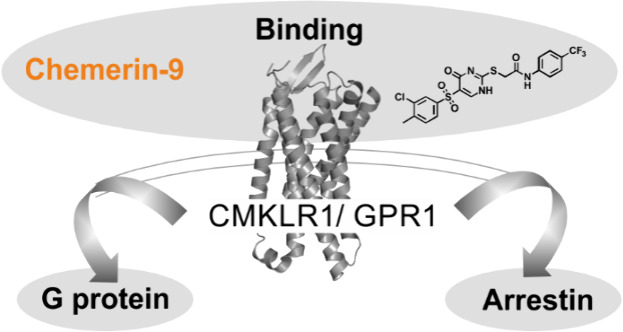

## Introduction

Over one-third of all FDA-approved drugs address G protein-coupled receptors (GPCRs).^1^ Interestingly, 92% of the FDA-approved drugs for GPCRs are small molecules, besides enzymes, plant extracts, proteins, and peptides.^2^ Small molecules are organic compounds with a molecular weight of less than 500 g/mol.^3^ Thus, small molecules are easy to modify, usually not immunogenic, frequently orally available, metabolically more stable than peptide-based drugs, and less complex to produce.^4,5^ They can bind either in an orthosteric binding site, such as agonists and antagonists, or in an allosteric binding site as allosteric modulators.

A promising target for the treatment of inflamed adipose tissues with small molecules is the chemokine-like receptor 1 (CMKLR1, also named ChemR23, or chemerin_1_). It is a rhodopsin-like GPCR, which transmits the signal from the endogenous protein–ligand chemerin by Gα_i/o_ protein-mediated pathways. Two further receptors, G protein-coupled receptor 1 (GPR1, CMKLR2, chemerin_2_) and CC-motif chemokine receptor-like 2 (CCRL2), are also considered to bind the chemerin protein.^6^ CMKLR1 and GPR1 share more than 50% similarity in their sequence. The chemerin protein is encoded by the retinoic acid receptor responder 2 gene (*Rarres2*) and is processed in different active isoforms: chemerinF156, chemerinS157, and chemerinK158.^7^ The most active isoform chemerinS157 (ChemS157) consists of 136 amino acids and acts as a chemoattractant and adipokine with an important role in inflammation and obesity.^8–12^ Its role in cancer is discussed controversially as it apparently can act as an antitumor and tumor-promoting protein depending on circumstances.^13^ The C terminus of ChemS157 was identified as a binding motif at CMKLR1. This nonapeptide called chemerin-9 (C9, Y^149^FPGQFAFS^157^) exhibits the same activity in G protein activation as the full-length ChemS157 but over 40-fold less potency in IP_1_ accumulation equilibrium readout (Figure 1).^14–16^ Two cryo-EM structures of CMKLR1 bound to C9 and G_i_ protein were published recently (PDB 7YKD and 8SG1).^17,18^ Currently, only a few antagonists are known to address CMKLR1. In 2014, the first antagonist 2-(α-naphthoyl)-ethyl-trimethylammonium iodide (α-NETA) was published by Graham et al. as an inhibitor for chemerin-mediated CMKLR1^+^ cell migration and arrestin recruitment with an IC_50_ value of 0.38 μM.^19–21^ In our investigations with α-NETA, the antagonistic effect only appears when a prestimulation of 30 min is applied to CMKLR1 *in vitro*. Nevertheless, in order to obtain reliable data regarding the binding domain of CMKLR1, a more potent antagonist was necessary.

**Figure 1:**
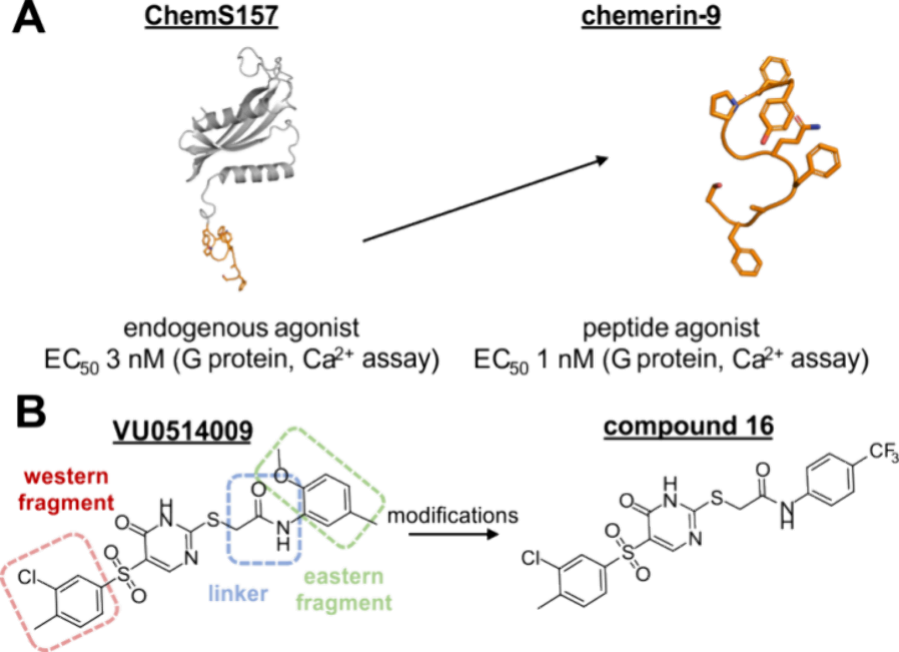
Agonists and the newly identified antagonist at CMKLR1. (A) Structures of agonists at CMKLR1. Chemerin-9 (C9) derives from the C terminus of chemerinS157 (ChemS157). The ChemS157 structure is virtually modeled based on the AlphaFold prediction. The C9 structure is abstracted from the cryo-EM C9-CMKLR1-Gi structure (PDB 8SG1). (B) Structure of the initial hit compound VU0514009 identified in high-throughput screening for CMKLR1 with highlighted fragments being modified to obtain compound **16**, which was the most effective compound in this study.

The antagonist CCX832, reported by ChemCentryx was highly studied in vasculature, but it has failed phase I trials.^22–26^ Two other antagonists were reported recently: Imaizumi et al. (compound **14f**)^27–29^ and Ko et al. (compound (*S*)-**26d**).^30^ Except for the (*S*)-**26d**, all molecules lack the description of binding and interaction within the receptor. Most studies only investigate the impact of their compounds on disease models and with regard to CMKLR1, but only a few, like Graham et al., Kumar et al., and Kennedy et al., have had a closer look at the similar chemerin receptor GPR1.^19,23,24^

In this study, we screened for small-molecule inhibitors such as negative allosteric modulators or antagonists at CMKLR1 to inhibit the chemerin-mediated CMKLR1 activity. In a high-throughput screening of 9280 compounds from the “Discovery Collection” drug-like library at Vanderbilt University, we obtained the hit structure VU0514009 and improved the inhibitory activity by rational chemical synthesis, yielding compound **16**. Together with molecular docking and mutagenesis, we identified the binding pose of the antagonist compound **16** at CMKLR1. Because arrestin recruitment and internalization were only slightly differently affected by compound **16** at GPR1 compared to CMKLR1, we also predicted a possible binding pose for GPR1, which showed a similar binding mode.

## Results and Discussion

### Identification of a Novel Antagonist at CMKLR1: SAR Studies

High-throughput screening (HTS) of a drug-like library “Discovery Collection” from Vanderbilt University was performed by using a Ca^2+^ flux readout for G protein activation with chemerin-9-stimulated HEK293 cells containing the CMKLR1-eYFP-Gα_Δ6qi4myr_ construct. The hit structure compound **1**, VU0514009, was identified as an inhibitor of C9-mediated CMKLR1 G protein activation (Figure 1). A selectivity test against the human Y1 receptor (hY1R) in the same assay showed no effect (Figure S96). The hit structure was checked for Pan-assay interference compounds (PAINS, promiscuous compounds)^31^ with ZINC online filter software^32,33^ resulting in no conflict. A similarity screening in the ZINC database resulted in similar structures, which only differed in the eastern phenyl ring.

Accordingly, a synthesis strategy was devised to resynthesize the initial hit compound **1** and further derivatize the eastern fragment (R1, Scheme 1) and the linker (R2, Scheme 1) to yield a small molecule that inhibits the C9-mediated effect on CMKLR1 more effectively than the original hit structure. This strategy focused on introducing the variable residues in the final step in order to improve efficiency and potency further. To realize the western fragment, the aniline **X1** was transformed to the disulfide **X2** via a Sandmeyer-like reaction based on an approach of a *BASF AG* patent (Scheme 1a–c).^34^ For this matter **X1** was treated with sodium nitrite and aq. HCl to form the corresponding diazonium salt. Subsequent reaction with potassium *O*-ethyl-carbonodithioate followed by hydrolysis yielded the disulfide **X2**. **X2** was purified by column chromatography and obtained with an overall yield of up to 53%. The disulfide was treated with sodium borohydride in ethanol (Scheme 1d). Direct isolation was unsuccessful, as the disulfide formed again. Consequently, ethyl 2-bromoacetate was directly added to the reduction mixture to carry out the nucleophilic substitution (Scheme 1e). The desired thioether **X4** was obtained with a yield of up to 69% over the two steps. Next, the thioether **X4** was oxidized with *meta-*chlorperoxybenzoic acid (*m*CPBA) to give sulfone **X5** with up to 82% yield (Scheme 1f). Based on ref 35, the sulfone **X5** was treated with ethyl orthoformate and acetic anhydride to receive the enol **X6** with up to 40% yield (Scheme 1g). The subsequent treatment with thiourea under basic conditions (NaOEt) produced the uracil with a yield of 91% (Scheme 1h). In the last step, the eastern fragments have been varied by nucleophilic substitutions with various bromated phenylacetamides (Scheme 1i). In order to modify the linker, the corresponding longer-chain amides were applied. The final compounds **1**–**26** were obtained with yields between 19 and 99%. All tested compounds had purities above 95% by HPLC analysis and were characterized by NMR and HR-MS.

**Scheme 1:**
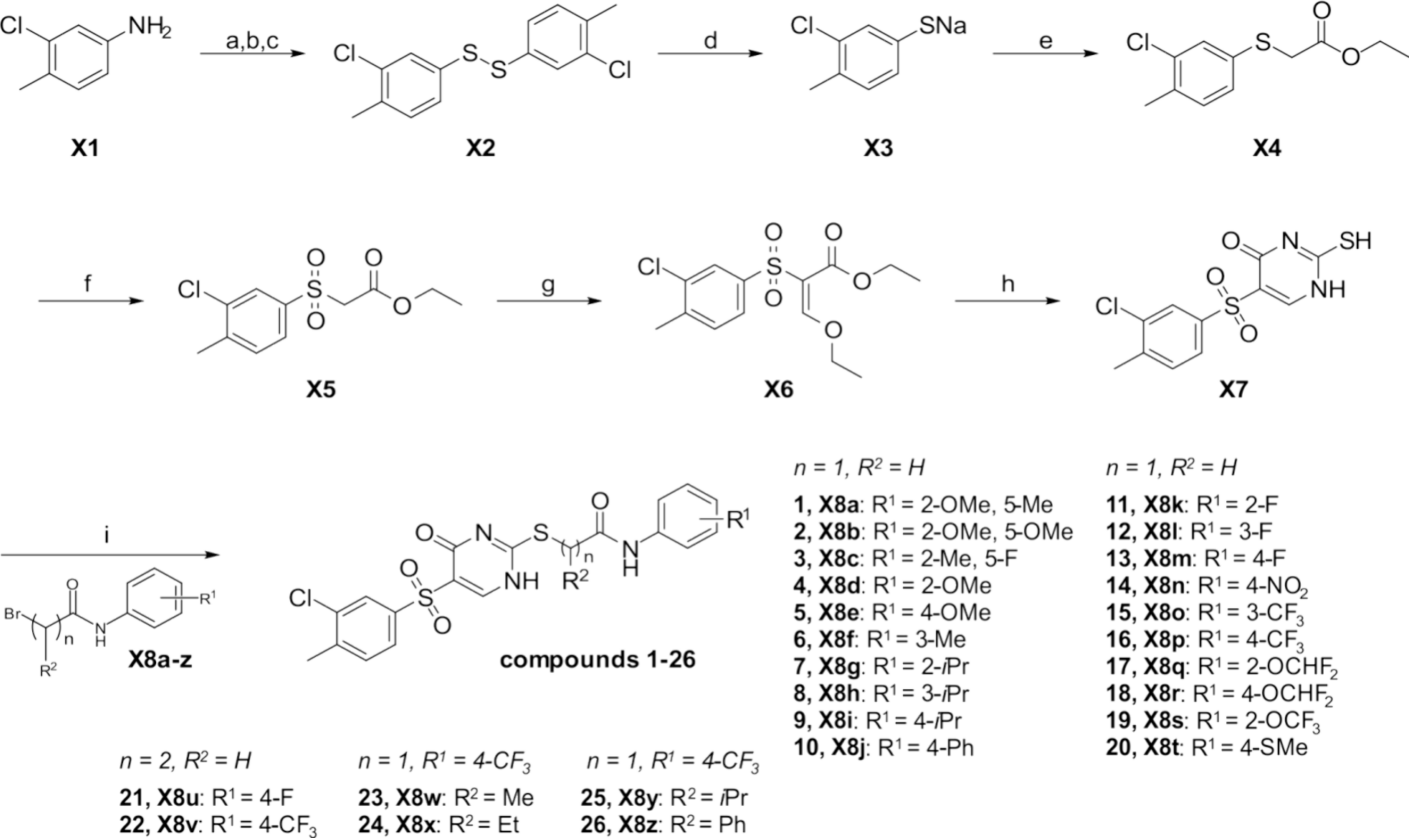
(a–i) Synthesis of the Hit and Lead Compound and Various Derivatives with R^1^ Substitution in the Eastern Fragment and R^2^ Substitutions in the Linker^a^

a Reagents, conditions, and yields: (a) NaNO_2_, HCl, H_2_O, 0 °C, 1 h; (b) Na_2_CO_3_, potassium *O*-ethyl-carbonodithioate, H_2_O, 70 °C, 2 h; (c) NaOH, EtOH, 100 °C, 14 h, 53%;^34^ (d) NaBH_4_, THF/EtOH (1:1), RT, 48 h, unisolated used in (e) ethyl bromoacetate, THF/EtOH (1:1), RT, 5 days, 69%; (f) mCPBA, DCM, RT, 3 days, 82%; (g) (diethoxymethoxy)­ethane, Ac_2_O, ZnCl_2_, 140 °C, 5 days, 40%; (h) thiourea, NaOEt, EtOH, RT, 16 h, 91%; (i) Na_2_CO_3_, ACN/THF (1:1), 35 °C, 16 h, 19–99%. The reaction in (g) and (h) was published by von Bredow et al.^35^

The hit compound **1** shows a methoxy and a methyl group at positions 2 and 5 of the eastern ring structure, respectively. These positions were investigated first due to their easier synthetic accessibility. The methyl group was exchanged by a methoxy group (**2**), as well as methyl and fluorine in the 2 and 5 positions (**3**). Both changes showed no significant differences in activity compared to hit **1**. Methoxy groups alone in positions 2 and 4 (**4** and **5**) and a methyl group in position 3 (**6**) were also tested. None of these changes had an influence greater than 2-fold compared to the DMSO control (ctl, Table 1). Bulkier, nonpolar, and even stronger positive electron-donating groups like isopropyl (**7**–**9**) and phenyl in position 4 (**10**) were tested as well. The phenyl derivative (**10**) showed the highest increase to a 6.5-fold EC_50_ shift compared to the control. Electron-withdrawing groups like fluorine in positions 2–4 (**11**–**13**) or even stronger nitro groups in position 4 (**14**) and trifluoromethyl in positions 2 and 4 (**15** and **16**) were also explored. The nitro group exhibited not the strongest effect but shifted the potency of C9-mediated CMKLR1 G protein activation 3.6-fold (Table 1). An even stronger effect was achieved by applying −CF_3_ to the hit structure in position 4 (**16**), but not in position 3 (**15**). After that, we evaluated groups like di- and trifluoromethoxy (**17**–**19**) and thiomethyl (**20**), which represent elongations or transitions between methoxy and trifluoromethyl groups. These modifications showed no improvement compared to compound **16**, which proved to be the most effective compound in the Ca^2+^ screen with an EC_50_ shift of 13.3-fold compared to the control.

**Table 1:** Results of the Ca^2+^ Flux Assay of C9 on CMKLR1 in the Presence of Compounds Derived from Compound **1** ^a^

**comp no.**	**EC**_**50**_ **[nM]^b^**	*E*_**max**_ **± SEM [%]^b^**	**pEC**_**50**_ **± SEM^b^**	**EC**_**50**_ **fold over ctl^b^**
ctl	0.8	99 ± 1.4	9.1 ± 0.04	1.0
1	2.0	93 ± 3.7	8.7 ± 0.11	2.6
2	1.3	102 ± 2.9	8.9 ± 0.08	1.6
3	1.4	92 ± 4.7	8.9 ± 0.13	1.8
4	0.8	108 ± 2.2	9.1 ± 0.06	1.1
5	1.6	87 ± 5.8	8.8 ± 0.17	2.1
6	1.1	89 ± 2.9	8.9 ± 0.08	1.5
7	1.4	99 ± 7.1	8.9 ± 0.18	1.8
8	1.7	86 ± 4.5	8.8 ± 0.13	2.2
9	3.6	94 ± 7.8	8.4 ± 0.19	4.7
10	5.0	96 ± 3.1	8.3 ± 0.07	6.5
11	0.3	105 ± 3.2	9.6 ± 0.09	0.3
12	1.4	90 ± 3.3	8.9 ± 0.09	1.8
13	1.4	100 ± 2.7	8.9 ± 0.07	1.8
14	2.8	101 ± 2.1	8.6 ± 0.05	3.6
15	1.5	97 ± 3.2	8.8 ± 0.08	2.0
**16**	**10.2**	**97 ± 3.2**	**8.0 ± 0.07**	**13.3**
17	1.8	95 ± 2.9	8.8 ± 0.07	2.3
18	3.4	95 ± 4.9	8.5 ± 0.12	4.4
19	2.6	89 ± 3.6	8.6 ± 0.09	3.4
20	4.3	93 ± 2.8	8.4 ± 0.07	5.6
21	0.8	98 ± 1.8	9.1 ± 0.06	1.0
22	5.3	95 ± 2.7	8.3 ± 0.06	6.9
23	6.1	96 ± 3.0	8.2 ± 0.07	7.9
**24**	**8.2**	**97 ± 6.5**	**8.1 ± 0.15**	**10.6**
25	5.4	94 ± 6.1	8.3 ± 0.15	7.0
26	2.8	95 ± 3.4	8.6 ± 0.09	3.6
27	2.5	98 ± 4.4	8.6 ± 0.10	3.2
28	2.4	113 ± 8.0	8.6 ± 0.17	3.2
29	2.8	91 ± 2.7	8.6 ± 0.07	3.6
**30**	**9.7**	**109 ± 9.7**	**8.0 ± 0.2**	**12.5**
31	7.4	110 ± 7.8	8.1 ± 0.16	9.6
32	2.6	113 ± 7.9	8.6 ± 0.16	3.4
33	2.0	95 ± 3.4	8.7 ± 0.10	2.6
34	3.2	104 ± 5.0	8.5 ± 0.11	4.2
35	1.8	111 ± 4.0	8.7 ± 0.09	2.4
36	2.0	119 ± 6.4	8.7 ± 0.13	2.6
37	2.8	97 ± 4.8	8.6 ± 0.12	3.6
38	5.0	97 ± 4.8	8.3 ± 0.11	6.5
39	1.1	97 ± 5.0	9.0 ± 0.14	1.4
40	5.7	95 ± 3.9	8.2 ± 0.09	7.4
41	1.4	102 ± 3.6	8.9 ± 0.09	1.8
42	2.1	97 ± 4.0	8.7 ± 0.10	2.7
43	6.4	102 ± 3.2	8.2 ± 0.07	8.2

a HEK293 cells stably expressing hCMKLR1-eYFP and a chimeric G protein Gα_Δ6qi4myr_ were stimulated with different concentrations of chemerin-9 (C9) and constant 30 μM compound resulting in double concentration response curves (dCRC). Control (ctl) contains 0.3% DMSO instead of the compound. Thus, all values represent the effect of C9 on CMKLR1 in the presence of the compound or DMSO. Thus, the higher the EC_50_ value, the greater the inhibitory effect. The three most effective compounds are highlighted in bold. Data represent the means ± SEM. All measurements were performed at least three times in duplicates. comp means compound.

b Of C9 in the presence of a 30 μM compound.

In the second step, we modified the linker by adding a C1 extension. The increased carbon chain in compound **22** (*n* = 2, R^1^ = 4-CF_3_) led to a 50% loss of the effect from compound **16**. To compensate for the extension to a certain extent, we exchanged the trifluoromethyl group with a fluorine in the eastern fragment (**21**), but this compound showed no activity. Thus, the following modifications at R^2^ and R^3^ were added to *n* = 1 and R^1^ = 4-CF_3_ (**16**, Scheme 2). Residue R^2^, originally hydrogen, was exchanged to alkyl moieties such as methyl, ethyl, isopropyl, or a phenyl group (**23**–**26**), which led to higher EC_50_ values compared to the control but to less potency compared to compound **16**.

**Scheme 2:**
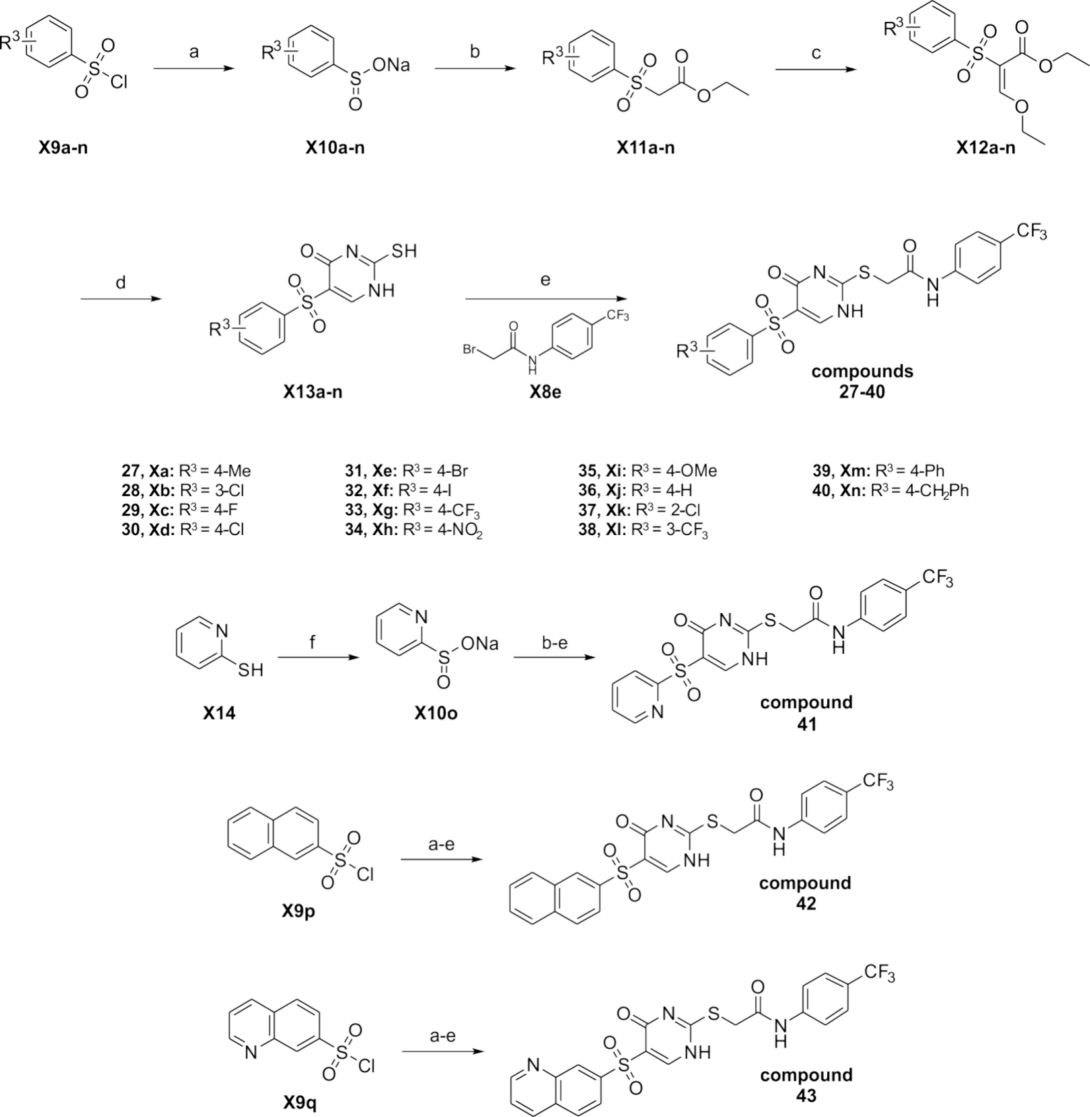
Synthesis of Thiouracil Derivatives with Various Substitutions on the Western Fragment^a^

a Reagents, conditions, and yields: (a) NaHCO_3_, Na_2_SO_3_, H_2_O, 70 °C, 86–99%;^37^ (b) ethyl bromoacetate, ACN/THF (1:1), 35 °C, 24 h, 32–76%; (c) (diethoxymethoxy)­ethane, Ac_2_O, ZnCl_2_, 140 °C, 5 days, 20–42%; (d) thiourea, NaOEt, EtOH, RT, 16 h, 16–92%; (e) Na_2_CO_3_, ACN/THF (1:1), 35 °C, 16 h, 37–96%;^35^ (f) aq. H_2_O_2_, aq. NaOH, EtOH, RT, 21 h, 95%.^36^

In this set of compounds with the modified eastern fragment, the ethyl derivative (**24**) showed the strongest influence on G protein activation at CMKLR1, being the most inhibitory compound next to compound **16**. Accordingly, a hydrogen in this position is essential to maintain the inhibitory effect in the Ca^2+^ response of compound **16**.

For further modification of the western fragment of compound **16**, a synthetic route similar to that shown in Scheme 1 was applied. The readily available sulfonyl chlorides **X9a**–**q** were used as starting points. Only **X10o** was derived from its thiol.^36^ The sulfonyl chlorides **X9a**–**q** were converted to the corresponding sulfinates **X10a**–**q** by reduction with sodium hydrogen carbonate and sodium sulfite (Scheme 2a).^37^ After a nucleophilic substitution with ethyl bromoactetate to form the sulfones **X11a**–**q** (Scheme 2b), the final compounds **27**–**43** were prepared as described for compounds **1**–**26**. Following the identification of the eastern fragment and linker in compound **16** (*n* = 1, R^1^ = 4-CF_3_, R^2^ = H) as the most active modification, we proceeded with modifications of the western fragment. We started with the 3-Cl, 4-Me substitution on the phenyl ring of the western fragment in compound **16** and proceeded to split this motif with only methyl at position 4 (**27**) or chlorine at position 3 (**28**). Both modifications resulted in a 3.2-fold EC_50_ shift relative to the control, accompanied by a notable loss of potency compared to compound **16**. This highlights the significance of both substituents. Subsequent to this, position 4 was subjected to extensive investigation with halogens (**29**–**32**), trifluoromethyl (**33**), nitro (**34**), methoxy (**35**), and even no substitution (**36**) on the phenyl ring. The halogens were found to exhibit varying degrees of activity, with chlorine (**30**) demonstrating the most notable increase (EC_50_ shift = 12.5). This trend was observed to diminish with increasing and decreasing halogen size, with fluorine (**29**) and iodine (**32**) exhibiting comparable influences. A shift of the chlorine to positions 2 and 3 (**37** and **28**, respectively) resulted in a potency loss of up to 70%. The introduction of electron-withdrawing groups, such as trifluoromethyl at positions 3 (**38**), at position 4 (**33**), and nitro (**34**) at position 4, led to a reduced activity as well. The replacement via an electron-donating group with a methoxy at the *para* position (**35**, EC_50_ shift = 2.4) had no influence compared to a trifluoromethyl group (**33**, EC_50_ shift = 2.6). Notably, the absence of a substituent on the phenyl ring (**36**, R^3^ = H) resulted in a similar effect on the potency, with a 2.6-fold EC_50_ shift. The western fragment, 2-pyridine (**41**), exhibited even lower inhibition (EC_50_ shift = 1.8). A bulky and nonpolar modification with phenyl (**39**) led to a biphenyl as the western fragment, resulting in an almost complete loss of activity (EC_50_ shift = 1.4). Conversely, an even bulkier moiety with benzyl (**40**) led to a significant increase in potency (EC_50_ shift = 7.4), indicating the importance of the flexibility of this residue compared to the rigid structure of the biphenyl (**39**). As anticipated, naphthalene (**42**) exhibited only weak inhibitory potency (EC_50_ shift = 1.8). In contrast, the similarly rigid 8-quinoline (**43**) had a significant increase in activity, with an 8.2-fold EC_50_ shift. This makes it the fourth most active compound among the western fragment modifications. In comparison, *para*-chloro, *para*-bromo, and 8-quinoline had the greatest impact on inhibition within the western fragment modifications.

In conclusion, none of the synthesized compounds exhibited a significant change in the efficacy (*E*_max_, Table 1). This was the first indication of an antagonist rather than an allosteric modulator. On the other hand, some modifications led to a more than 3-fold change in potency (change in EC_50_ for **9**, **10**, **15**–**17**, **19**, **20**, **22**–**34**, **37**, **38**, **40**, and **43**). Modifications at R^1^ in position 4, such as in compounds **9**, **10**, **14**, **16**, **18**, and **20**, demonstrate the importance of this position for CMKLR1 antagonistic activity. Moreover, the trifluoromethyl group is preferred over other substitutions. Additional alkyl substitution at R^2^ (**23**–**26**) reduces the effect of compound **16**. The diminishment of the −CF_3_ group to a single fluorine for the compounds with *n* = 1 (**16** to **8**) or *n* = 2 (**22** to **21**) was unsuccessful in maintaining the compound **16**- or **22**-mediated effect. Therefore, the −CF_3_ group is essential and cannot be improved by this replacement. With regard to the western fragment, compounds **30** (R^3^ = 4-Cl), **31** (R^3^ = 4-Br), and **43** (R^4^ = quinolone) contain residues that are useful for the inhibition of CMKLR1 G protein activation of C9 but do not reach the 3-Cl, 4-Me substitution effects of compound **16**. To figure out whether only parts of compound **16** are sufficient for this inhibitory effect, we tested the western and the eastern fragment **X7** and **X8p** alone and in a mixture (v/v, 1:1), but no effect was determined (Figure S97). Thus, the full structure is needed to reveal necessary interactions within the receptor binding pocket.

### Characterization of the Novel Antagonist Compound **16** at CMKLR1

Compound **16** was identified in the first SAR screening as the compound with the highest potency (Table 1). Ca^2+^ flux readout reveals a significant 70-fold EC_50_ shift when 60 μM compound **16** (*p* < 0.0001) was applied to chemerin-9-stimulated HEK293 cells stably expressing the CMKLR1 (Figure 2A). Figure 2B illustrates its effect on different chemerin-9 concentrations. The curves show a strong influence of compound **16** on 10^–8^ M chemerin-9 and higher concentrations. The Ca^2+^ response was furthermore analyzed in a Schild plot^38^ presenting a slope of 1.1, which means that the effect is slightly reduced in lower compound concentrations (slope >1.0). No saturation of the effect has been observed. Thus, this compound interacts most likely at the orthosteric binding site. The parallel concentration–response curves and unaltered maximal response characterize the action of a competitive antagonist.^39^ Moreover, compound **16** generates a p*A*_2_ value of 5.96 (Figure 2C). The p*A*_2_ value represents the −log­(*c*_antagonist_), which causes a 2-fold shift of the agonist concentration–response curve. Reported studies about antagonists at CMKLR1 mostly provide the IC_50_ value instead of p*A*_2_ values.^19,29,30^ For comparison, the IC_50_ value was determined with a submaximal Ca^2+^ response setup. CMKLR1 was stimulated by a submaximal concentration of chemerin-9 (15 nM corresponding to EC_80_) and increasing concentrations of the compound **16** (Figure 2D), resulting in an IC_50_ = 37 μM.

**Figure 2:**
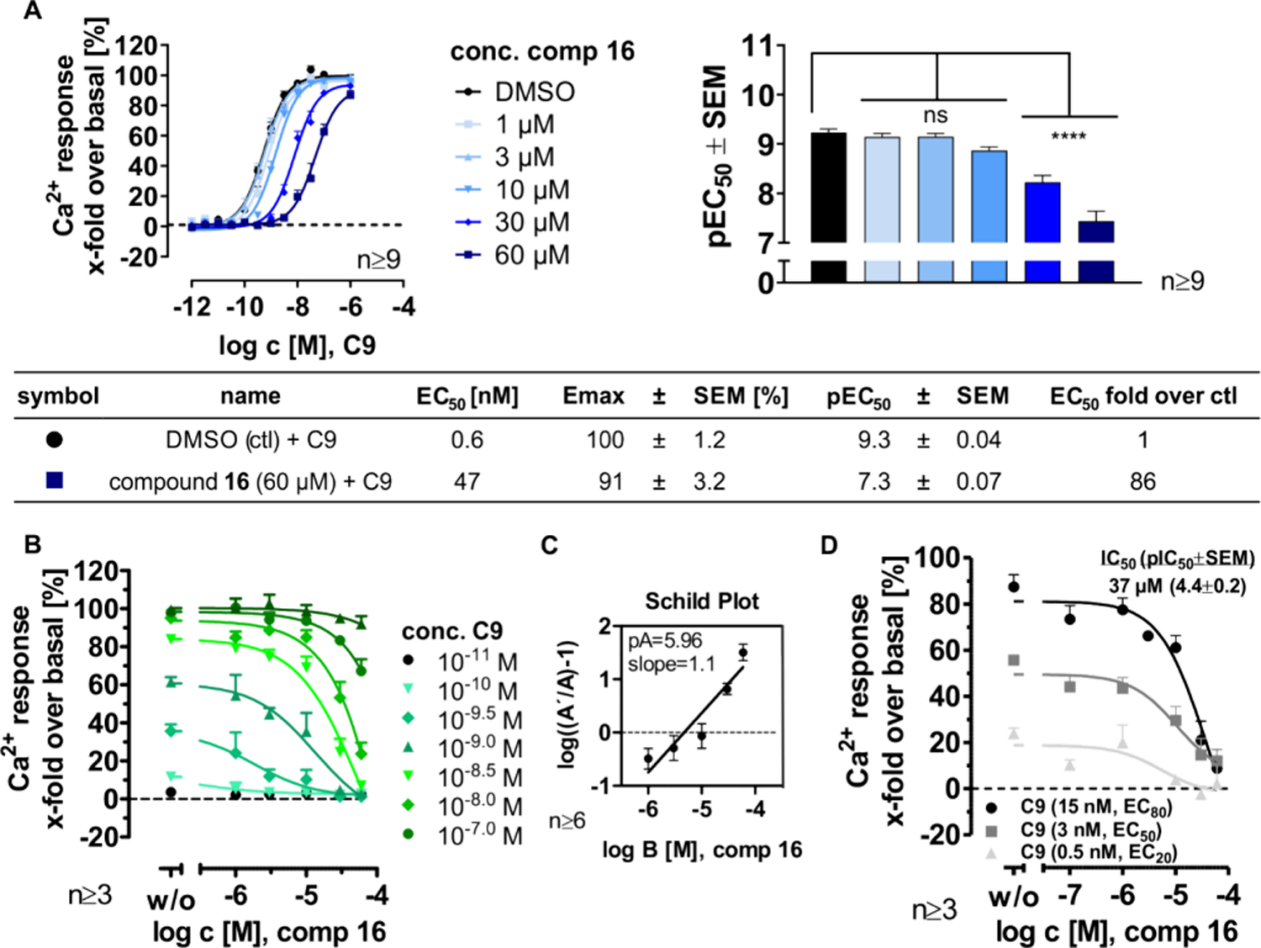
Influence of compound **16** at CMKLR1 on the G protein signaling. Ca^2+^ flux assay performed with HEK293 stably transfected with CMKLR1-eYFP and a chimeric G protein Gα_Δ6qi4myr_. (A, B) Cells were stimulated with increasing chemerin-9 (C9, 1 μM–100 nM) and increasing concentration of compound **16** (1–60 μM), yielding a double concentration–response curve (dCRC), demonstrated with chemerin-9 on the *x*-axis (A) and with compound **16** concentration on the *x*-axis (B). The bar graph in (A) shows the significance of the pEC_50_ decrease from dCRC. Statistical test: ANOVA with Dunnett’s multiple comparison test was performed using GraphPad Prism version 10. ns, not significant, **** *p* < 0.0001. (C) The Schild plot analysis of the dCRC reveals a straight, linear line. *A′* means the EC_50_ value with the antagonist, and *A* means the EC_50_ value without the added antagonist. (D) Submaximal Ca^2+^ response: CMKLR1 was constantly stimulated with EC_80/50/20_ of C9 (15 nM (black), 3 nM (dark gray), and 0.5 nM (light gray)) and an increasing concentration of compound **16**. All assays shown here were performed at least three times in duplicates. Data are represented as means ± SEM.

To compare the activity of this compound to the widely investigated α-NETA antagonist, the same Ca^2+^ assay setup was performed for G protein signaling with compound **16** and α-NETA. No influence on CMKLR1 G protein signaling of α-NETA has been observed (Figure S98); thus, a preincubation step with different compound concentrations was applied for 30 min as reported before.^19,28,30^ Next, the preincubated receptor was stimulated with 2.5 nM C9 as the peptide ligand. The effect of compound **16** is slightly enhanced when only 10 μM compound **16** (*p* = 0.003) was used for preincubation and furthermore comparable to α-NETA at the higher concentrations tested. Figure 3 illustrates the improvement from compound **1** (VU0514009) as the hit structure to compound **16**.

**Figure 3:**
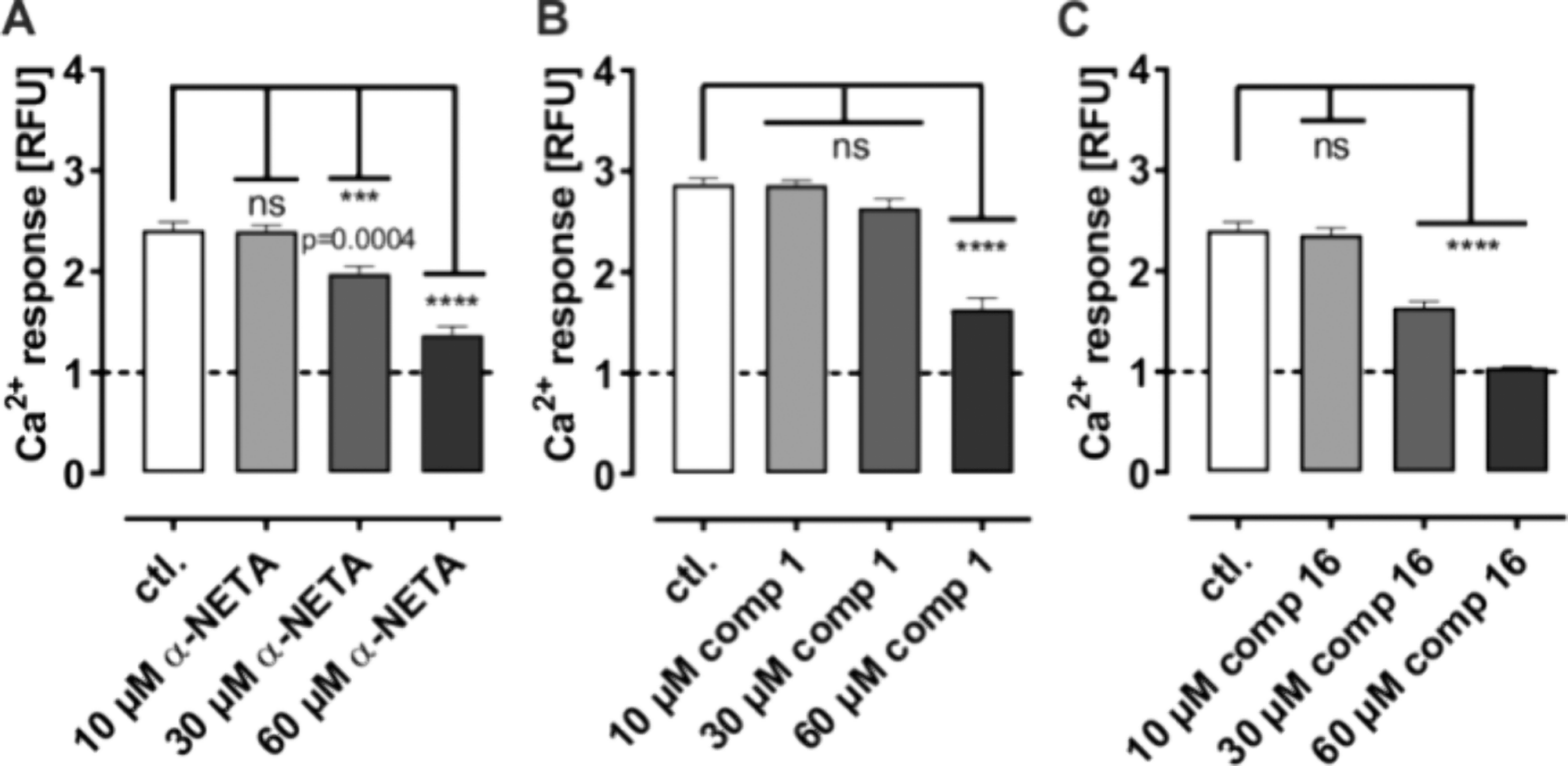
Comparison of the inhibitory effect of α-NETA and compounds **1** and **16**. Stably transfected HEK293 cells containing CMKLR1-eYFP and a chimeric G protein Gα_Δ6qi4myr_ were preincubated with α-NETA (A), compound **1** (B), or **16** (C) for 30 min, and the Ca^2+^ response was measured directly after adding a constant concentration of 2.5 nM of the ligand chemerin-9 (EC_50_ value). The assay was performed in quadruplicate at least three times. Data are shown as means ± SEM. The ANOVA with Dunnett’s multiple comparison test was performed with Prism version 10 software for statistical analysis. ***p < 0.001; ****p < 0.0001; ns, not significant.

In summary, compound **16** is, on the one hand, less active with an IC_50_ value of 37 μM compared to the published IC_50_ value of α-NETA with 0.38 μM.^19^ However, compound **16** is active without any preincubation step in contrast to α-NETA. This suggests a different mode of action for compound **16** and a much faster interaction with CMKLR1. Nevertheless, a control experiment without any preincubation step (Figure S98) revealed a slightly weaker inhibitory effect of compound **16** on C9-stimulated CMKR1 G protein activation. The stronger inhibition through longer incubation time elucidates the slope value of >1.0 in the dCRC experiments performed without any incubation time of the compounds (Figure 2C).

### Selectivity of Compound **16** with regard to Chemerin Receptors

Owing to the similarity of the CMKLR1 and GPR1 sequences of over 50%, the influence of the optimized compound **16** was further investigated. No G protein signaling has been detected for GPR1 in the Ca^2+^ flux assay, which has also been reported in several studies.^40,41^ Thus, arrestin recruitment was investigated for CMKLR1 and GPR1 to study the antagonistic effect within the chemerin receptor family. Arrestin-3 (arr-3, β-arrestin-2) and arrestin-2 (arr-2, β-arrestin-1) can be investigated because of their ubiquitous expression.^42^ Considering that GPR1 favors arrestin-3 over arrestin-2,^40,43^ the influence of compound **16** was tested on both chemerin receptors for its impact on C9-mediated arr-3 recruitment (Figure 4A,C). CMKLR1 exhibits an EC_50_ value of 96 nM, which was shifted to higher EC_50_ values when 30 or 60 μM compound **16** was added. The inhibition is clearly visible for CMKLR1 but not determinable due to the lack of saturation of the concentration–response curve. Chemerin-9-mediated arr-3 recruitment to GPR1 was observed with an EC_50_ value of 2 nM, which is shifted to 7-fold or 22-fold when 30 or 60 μM compound **16** is applied, respectively. Thus, C9 reveals a lower EC_50_ value at GPR1 arr-3 recruitment than at CMKLR1. Compound **16**-mediated inhibition is reduced at GPR1 compared to CMKLR1. The overall potency shift produced by compound **16** is approximately 22-fold for GPR1 and at least 5 times higher with >100-fold for CMKLR1 arr-3 recruitment.

**Figure 4:**
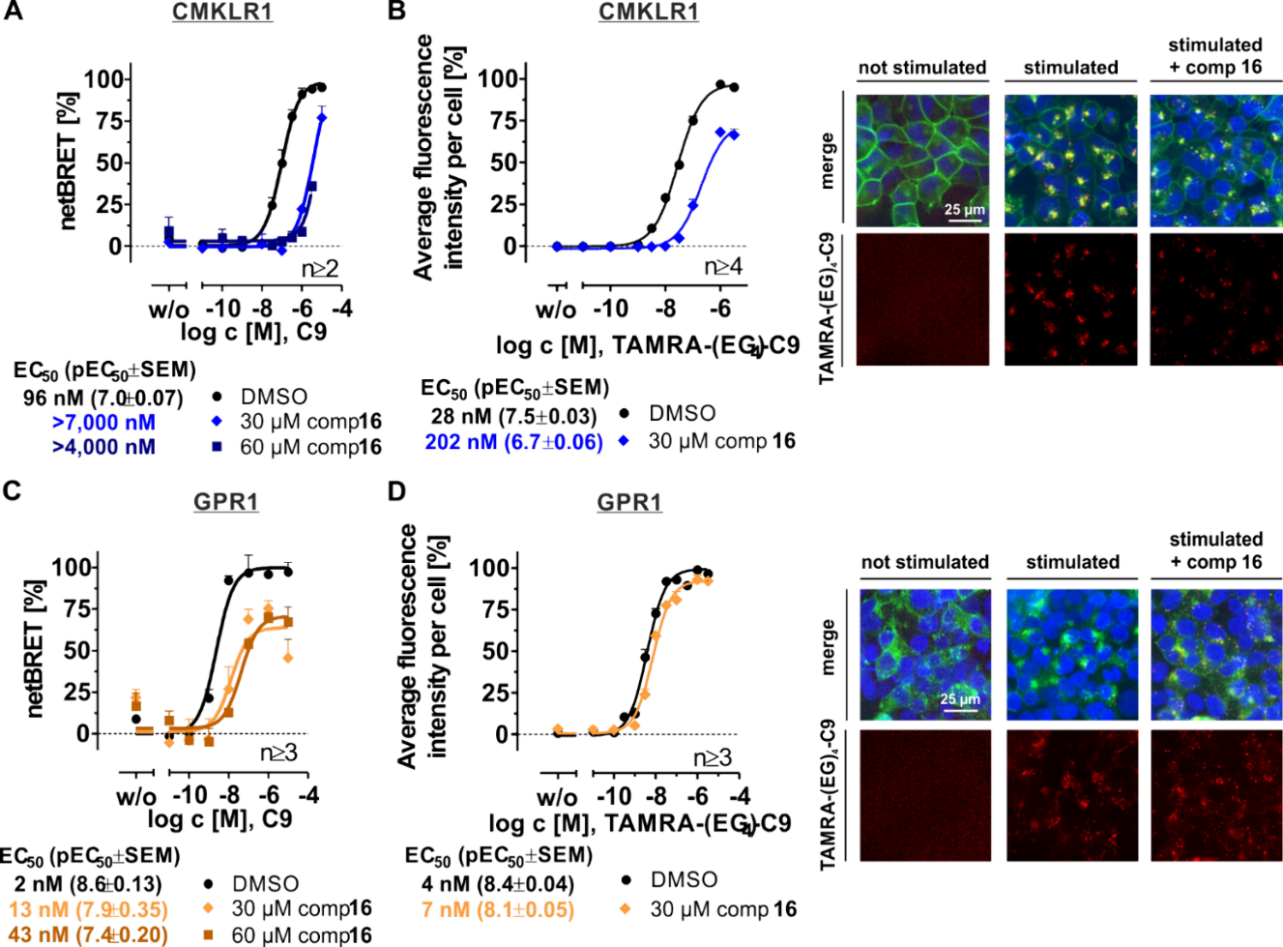
Characterization of compound **16** at chemerin receptors with regard to internalization and arr-3 recruitment. (A, C) Arrestin-3 recruitment BRET: HEK293 were transiently transfected with Nluc-arr-3 and CMKLR1-eYFP (A) or GPR1-eYFP (C). Compounds, peptides, and coelenterazine h solution were added, and 15 min afterward, the resulting BRET ratio was detected. Cells are stimulated with increasing chemerin-9 (C9) and 30 or 60 μM compound **16** or DMSO as a negative control, revealing a double concentration–response curve. (B, D) An internalization assay was recorded using a high-content imaging system (Molecular Devices). Stably transfected HEK293 cells containing hCMKLR1-eYFP-Gα_Δ6qi4myr_ (B, receptor in green fluorescence) or hGPR1_eYFP (D, receptor in green fluorescence) were stimulated with TAMRA-(EG)_4_-C9 (red fluorescence) in the presence of 30 μM compound **16** or DMSO as a negative control. A granularity module determined the intracellular internalized TAMRA-labeled peptide. Therefore, nuclei (blue fluorescence) were analyzed with 5–30 μm diameter and 10 gray levels above the background and granules by TAMRA-peptide (red fluorescence) with 2–5 μm diameter and 100 gray levels above the background. The microscopic images show a control without compound and peptide stimulation (left panel), with 10^–5.5^ M TAMRA-(EG)_4_-C9 stimulation but without the compound (middle panel), and the fluorescence of TAMRA-peptide in the presence of compound **16** (right panel). Scale bar, 25 μm. Data are shown as means ± SEM. All assays were performed at least twice in technical duplicates for internalization and in triplicates for BRET studies.

Moreover, the receptor internalization was determined with a high-throughput imaging system and HEK293 cells stably expressing hCMKLR1-eYFP-Gα_Δ6qi4myr_ (as it was used for Ca^2+^ flux experiments) or hGPR1-eYFP by detecting the receptor-mediated internalization of the 5-carboxytetramethylrhodamine (TAMRA)-labeled chemerin-9 peptide (Figure 4B,D). Compound **16** reduces the C9-induced CMKLR1 internalization 7-fold when 30 μM compound **16** is applied but only 2-fold for GPR1. Surprisingly, this is a less strong effect compared to the influence on arr-3 recruitment at GPR1 (Figure 4C). These results suggest a slight selectivity for CMKLR1 over GPR1.

Taken together, compound **16** shows an antagonistic behavior on arr-3 at both chemerin receptors with a preference for CMKLR1. Microscopic images produced for the quantification of receptor-mediated peptide uptake show, as expected, that CMKLR1 is expressed in the membrane, whereas GPR1 is only partially available for ligand binding in the membrane and is partially located in the intracellular compartment (see not stimulated images in Figure 4B,D).^15,18,44^ This is due to the constitutive activity of GPR1, which has been reported before.^43,44^ Similar results have been shown for the protease-activated receptor 1 (PAR1) or the C–X–C motif chemokine receptor type 4 (CXCR4) with arrestin- and agonist-independent internalization.^45^ Therefore, GPR1 is partially not accessible for antagonist interaction and its mediated reducing effect, and similarly, it is not accessible for C9 binding. Consequently, there should be analogous potency shifts for arr-3 recruitment and receptor internalization. However, TAMRA-labeled C9 is internalized with 4 nM potency at GPR1 (Figure 4D) and thus is nearly as effective as the C9-stimulated arr-3 recruitment with EC_50_ = 2 nM at GPR1 (Figure 4C). We concluded that the binding of TAMRA-labeled C9 itself is preferred over the binding of the antagonist compound **16** at GPR1 but not at CMKLR1. This is in accordance with the higher *K_i_* value for C9 binding at CMKLR1 over GPR1 (19.0 and 4.9 nM) published in 2022 by Czerniak et al.^16^ Thus, we suggest that the binding of compound **16** is weaker at GPR1 than at CMKLR1 because the agonist binding is stronger at GPR1 than at CMKLR1 and therefore the agonist is not easily displaceable by the antagonistic compound **16**. To investigate this hypothesis, a displacement BRET assay and computational modeling of compound **16** docked into CMKLR1 or GPR1 were performed.

### Binding Characteristics of Compound **16** to Chemerin Receptors

Nanoluciferase (Nluc) constructs of GPR1 and CMKLR1 (Nluc-CMKLR1 and Nluc-GPR1) were used to investigate the displacement of the bound TAMRA-labeled agonist by compound **16** (Figure 5). The assay is based on the interaction by bioluminescence resonance energy transfer (BRET) of Nluc, fused to the N terminus of CMKLR1 or GPR1, and the fluorophore TAMRA, which is bound to the peptide ligand (TAMRA-C9) or protein ligand ([K^141^(TAMRA)]-ChemS157). The displacement of bound TAMRA-labeled ligands with the unlabeled peptide ligand (C9) or protein (ChemS157) or compound **16** at both Nluc constructs was performed as recently described.^16^ Compound **16** and the hit compound **1** were able to displace bound TAMRA-C9 at GPR1 with *K_i_* > 1000 nM (Figure 5B) and [K^141^(TAMRA)]-ChemS157 with a not determinable *K_i_* value (Figure 5D). Compared to the displacement of TAMRA-C9 with C9 (*K_i_* = 28 nM, Figure 5B), the compounds are 50-fold less effective, which is even higher for the displacement of [K^141^(TAMRA)]-ChemS157 with ChemS157 (*K_i_* = 4 nM to n.d., Figure 5B).

**Figure 5:**
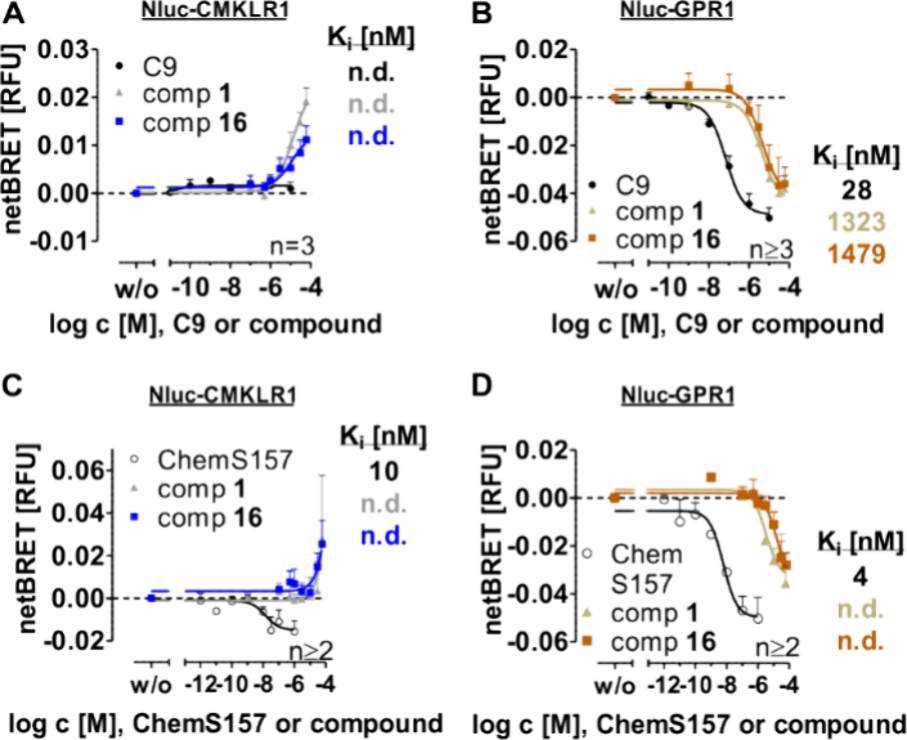
Displacement BRET of Nluc-fused chemerin receptor constructs with TAMRA-C9 and [K^141^(TAMRA)]-ChemS157. All displacement BRET assays were performed using transiently transfected HEK293 cells expressing Nluc-CMKLR1 (A, C) or Nluc-GPR1 (B, D). A defined constant TAMRA-C9 (A, B) or [K^141^(TAMRA)]-ChemS157 (C, D) concentration is bound to the respective receptor. Increasing concentration of an unlabeled ligand (C9 or ChemS157) or compound was tested for displacement of a bound TAMRA-labeled ligand. Data are shown as means ± SEM from at least two independent experiments performed in triplicates. n.d., not determinable; Nluc, Nanoluciferase.

Consequently, the protein binds more strongly to GPR1 than the peptide ligand, which has already been described by de Henau et al. and Czerniak et al.^16,40^ This occurs mainly because the protein chemerin contributes with more than only one binding site and a different interaction mode.^46^

On the other hand, we know that TAMRA-C9 cannot be removed with C9 from the binding site at Nluc-CMKLR1, but surprisingly, it also cannot be removed with our compounds (Figure 5A). They show a positive netBRET value increasing with higher compound concentrations, meaning that the bound TAMRA-labeled ligand interacts even better with CMKLR1 in the presence of compound **1** or **16**. Consequently, the compounds might change the orientation of TAMRA to the Nluc at CMKLR1. The same increased BRET ratio happens for the displacement of [K^141^(TAMRA)]-ChemS157 with the compounds at Nluc-CMKLR1, whereas ChemS157 is able to replace TAMRA-labeled ChemS157 with *K_i_* = 10 nM (Figure 5C). These results support the possibility that compound **16** binds slightly differently to GPR1 than to CMKLR1 (Figure 5), which can explain the stronger inhibition of CMKLR1 over GPR1 signaling (Figure 4) by weakening the agonist binding through an overlap within the orthosteric binding site.

### Molecular Docking Study

Next, we aimed to identify the binding pose of compound **16** bound to CMKLR1. The cryo-EM structure of C9-CMKLR1-G_i_ ^18^ supported an insight into the orthosteric binding site and, hence, possible interactions for compound **16**. Different CMKLR1 variants were chosen, incubated with increasing compound concentrations, and then stimulated with their respective EC_80_ value in a submaximal activation Ca^2+^ response assay (Figure 6A).

**Figure 6:**
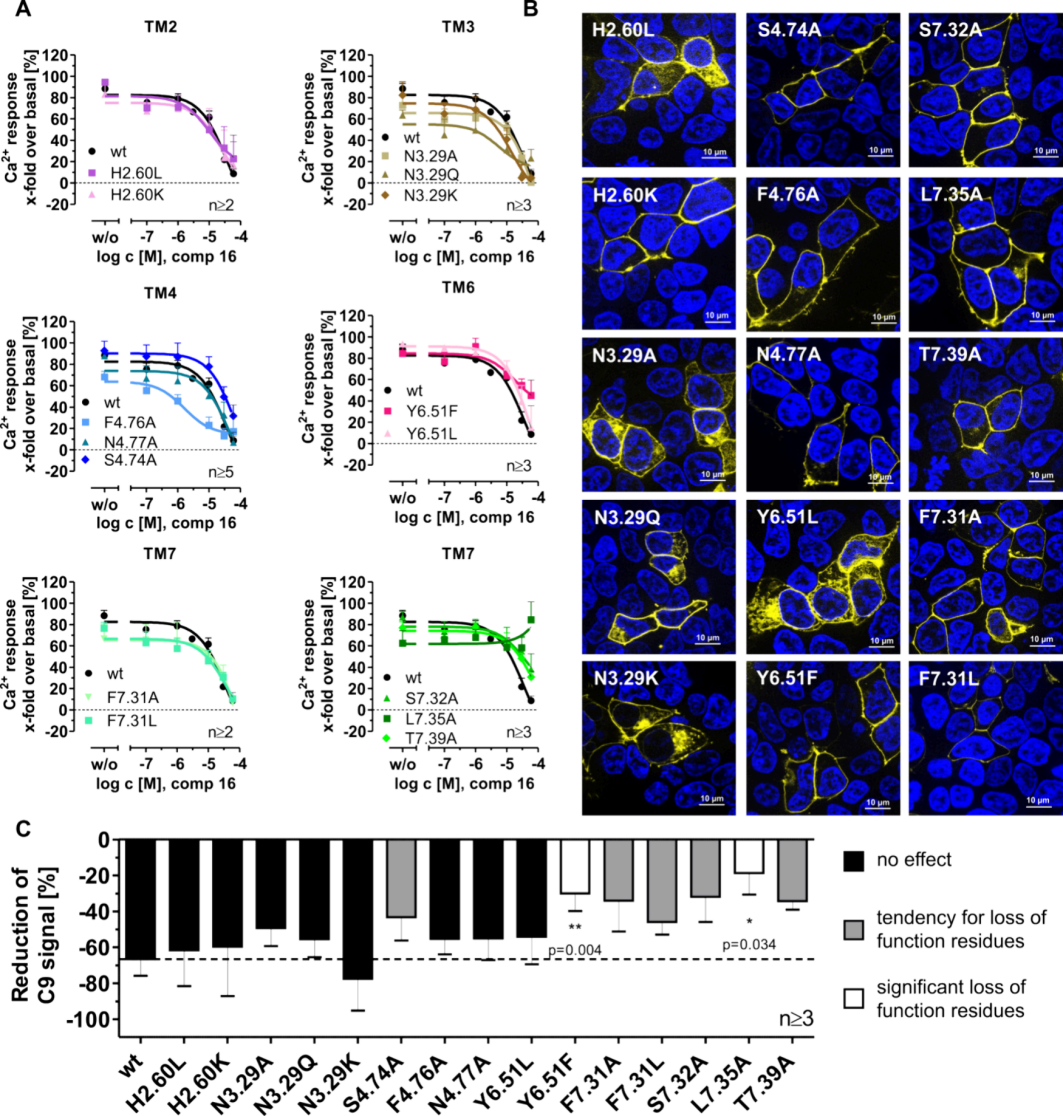
Examining the binding site of compound **16** at CMKLR1. (A) The chemerin receptor CMKLR1 was stimulated with a submaximal chemerin-9 concentration (C9 EC_80_) and increasing concentrations of compound **16**. Residues in transmembrane helix (TM) 2, 3, 4, 6, and 7 were investigated using the Ca^2+^ flux assay, whereby HEK293 cells transiently expressing the respective receptor-eYFP construct and the chimeric G protein Gα_Δ6qi4myr_. H2.60 (residue 95), N3.29 (residue 116), S4.74 (residue 188), F4.76 (residue 190), N4.77 (residue 191), Y6.51 (residue 276), F7.31 (residue 294), S7.32 (residue 295), L7.35 (residue 298), and T7.39 (residue 302). (B) Receptor localization after transient transfection of HEK293 cells with 1000 ng of receptor-eYFP variants (yellow fluorescence) in a μ-slide 8 well (Ibidi). Cell nuclei were stained with the dye Hoechst 33342 (blue fluorescence). Scale bar, 10 μm. This experiment was done twice in duplicates, whereby one representable image is shown. (C) Reduced Ca^2+^ response in the presence of 30 μM compound **16** after stimulation with an EC_80_ value of chemerin-9 (C9). The unpaired *t* test with Welch’s correction was used for statistical analysis of the receptor variants compared to the wild type (wt) in Prism version 9. **p < 0.01, *p < 0.05. Data are shown as means ± SEM from at least three independent experiments performed in triplicates. Receptor residues are named according to the nomenclature of Ballesteros and Weinstein.^53^

The CMKLR1 variants H2.60A, N3.29R, and Y6.51A were not investigated in the submaximal Ca^2+^ response assay because of their poor membrane expression compared to the CMKLR1 wild-type expression (Figure S99B) and therefore inadequate Ca^2+^ response (Figure S99A). The variants N3.29A/Q/K, H2.60L, and Y6.51L were expressed partially in the membrane but could be activated to 80% G protein activation (Figure 6A). Other amino acids were introduced at position H2.60 and Y6.51 to investigate these further. All other CMKLR1 variants presented in Figure 6 are expressed like the wild type. To summarize the submaximal Ca^2+^ response assay results, a bar graph illustrates the reduction at 30 μM compound **16** of the Ca^2+^ response for various variants when activated to 80%. A significant influence of compound **16** is detectable for L7.35A (*p = 0.034) and Y6.51F (**p = 0.004) as shown by a lower reduction in Ca^2+^ response. Although not statistically significant, but still, a reduced Ca^2+^ response was observed for S4.74A, F7.31A/L, S7.32A, and T7.39A. Interestingly, S7.32A and T7.39A are not involved in chemerin-9 binding (Figure S99A). Thus, these positions are exclusively interacting with compound **16**. One gain of function mutation for F4.76A was detected, suggesting that a smaller side chain like alanine is preferred over the phenyl ring in position 4.76 for compound **16** activity.

Based on the mutagenesis experiments, we performed computational docking of compound **16** to the inactive structure of CMKLR1, which shows similarities to the two published cryo-EM CMKLR1 structures (Figure S100). In consideration of the exact input structure for compound **16**, it is essential to recognize the potential for three distinct tautomeric forms. In principle, the isomers pyrimidin-4-ol, dihydropyrimidin-4­(1*H*)-one, and dihydropyrimidin-4­(3*H*)-one are conceivable. In our molecular docking study, we chose to use dihydropyrimidin-4­(3*H*)-one. This tautomer has been determined to exhibit the highest probability of occurrence according to our calculations (e.g., with the “Tautomer Search” from Rowan Scientific^47^), relevant literature,^48^ and our own NMR experiments. Additionally, this isomeric state has been shown to exhibit additional intramolecular stabilization in our models. The possible occurrence of the dihydropyrimidin-4­(1*H*)-one isomer is not expected to significantly influence the proposed binding mode. The protein models were generated with AlphaFold2, and various docking tools were used including RosettaLigand,^49,50^ DiffDock,^51^ and DynamicBind.^52^ Here, the experimentally observed mutation effects were applied as docking constraints to guide the selection of compatible binding poses. While RosettaLigand successfully generated poses that satisfied all of these constraints, both DiffDock and DynamicBind predictions failed to do so. Despite their advanced capabilities, discrepancies between the computational predictions and the experimental results were observed. In agreement with all experimental *in vitro* constraints, we propose here a computational model with RosettaLigand of the possible binding mode of compound **16** at CMKLR1 (Figure 7).

**Figure 7:**
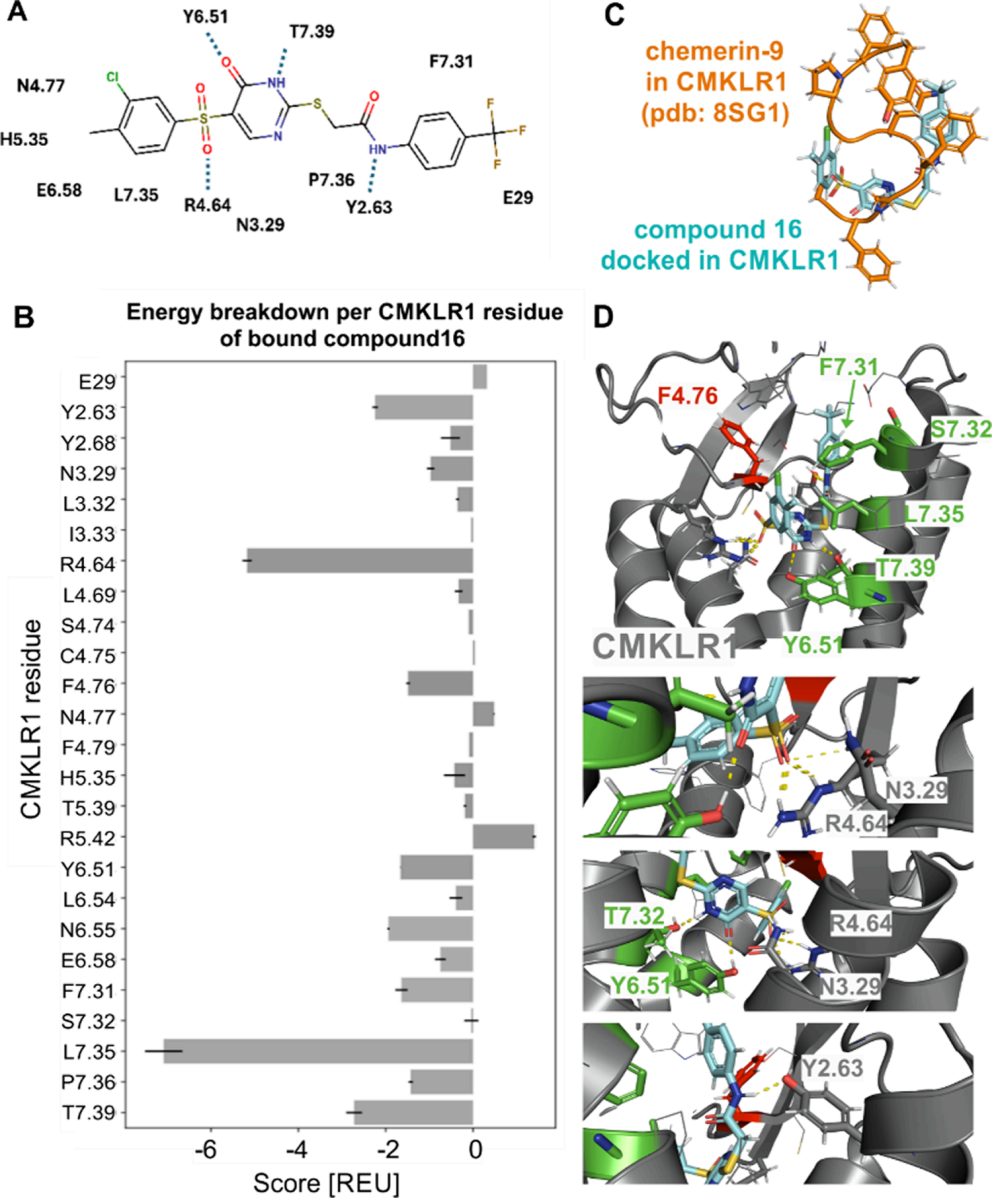
Model of compound **16** binding into an inactive CMKLR1 structure. (A) Schematic representation of interactions between CMKLR1 residues and compound **16**. (B) Energy breakdown per residues created by using RosettaLigand. The bar graph represents the results (means ± SEM) from the three best fitting models. The CMKLR1 residues were named based on the Ballesteros–Weinstein nomenclature,^53^ whereby CMKLR1 residues of the N and C terminus or the loops were named according to their position. (C) Orientation of chemerin-9 from the cryo-EM structure (PDB 8SG1, orange) in comparison to compound **16** (cyan) from the best computational models. The model illustrates the inactive CMKLR1 structure based on the AlphaFold2-predicted structure. (D) Side view into the inactive CMKLR1 (gray) model. Residues in green are the relevant residues for compound **16** binding identified by mutagenesis studies. The residue in red depicts the gain of function position F4.76, which was elucidated by mutagenesis studies. Zoom-ins into the model focus on polar interactions (yellow dashed lines) between compound **16** and CMKLR1 residues.

To summarize the results from the submaximal Ca^2+^ flux assay (Figure 7A) and the energy breakdown performed with RosettaLigand (Figure 7A,B), strong interactions of compound **16** were mainly identified within transmembrane helices 6 and 7. The highest energy loss for the interaction of compound **16** is detected for L7.35A (Figure 7B,D), meaning that L7.35 contributes the most to compound **16** orientation in the binding pocket. Additionally, the model exhibits several polar interactions with Y2.63, N3.29, R4.64, F6.51, and T7.39 (Figure 7). To investigate position N3.29, it was mutated to an asparagine and leucine variant, which were completely inactive when stimulated with chemerin-9 (data not shown). Thus, only N3.29A, Q, and K, which can be activated, were screened with compound **16**.

None of the variants N3.29A/Q/K contributed to compound **16**-mediated reduction of Ca^2+^ response (Figure 6A), which also presents a low energy loss (Figure 7C). In addition, R4.64 could not be further investigated in detail for the compound **16** influence on the CMKLR1 activity because the receptor variants R4.64A/K/Q did not show any activity anymore when they were stimulated with C9.^18^ A mutation of this position reveals a strong interaction with C9, mainly to form the “S-shape” of C9 in the CMKLR1 orthosteric binding pocket.^17,18^ Further, Y6.51 contributed with a −OH group to a polar interaction (Figure 7D). The tested variant Y6.51F misses this hydroxyl group and leads to a break of the polar interaction between the hydrogen of the hydroxyl group and the oxygen from compound **16**, which is nicely represented in the gained model (Figure 7A,D). Compound **22**, which contains an additional methylene group (Scheme 1), shows a less effective inhibition compared to compound **16**. Although the additional methylene group is located in the linker part (Scheme 1), it could influence the whole orientation as well as the interaction of the CMKLR1 residue Y6.51 to the compound structure. F7.31 seems to define the size of the binding pocket to guide the compound into it but does not show a direct contact. The receptor residue F4.76 also does not point directly to the compound. However, the receptor variant F4.76A shows a gain of function in the submaximal Ca^2+^ assay, leading to the conclusion that the bigger phenylalanine hinders compound **16** to reduce the Ca^2+^ response activity more efficiently, and alanine is favored here for this reducing effect.

Finally, the model demonstrates exclusive interactions between the compound and CMKLR1, which are not relevant for C9-mediated receptor activation, and thus, the model exhibits differences between antagonistic and agonistic interaction. Because compound **16** also showed antagonistic behavior at GPR1, we used the AlphaFold2 and RosettaLigand approach, successfully applied at CMKLR1, to model an inactive GPR1 structure with docked compound **16** (Figure 8). The strongest polar interactions were formed with R4.64, F4.76, and Y6.51 of GPR1 to compound **16**. H2.60 contributed with lower but also polar contacts to compound **16**. I7.35 revealed no clear interaction partner but contributed the most energy (energy breakdown Figure 8B), as observed for compound **16** docked into CMKLR1 (Figure 7B). Altogether, the computational models of compound **16** at GPR1 and CMKLR1 support the conclusion from the displacement and binding experiments that antagonist **16** is bound to GPR1 slightly weaker than to CMKLR1.

**Figure 8:**
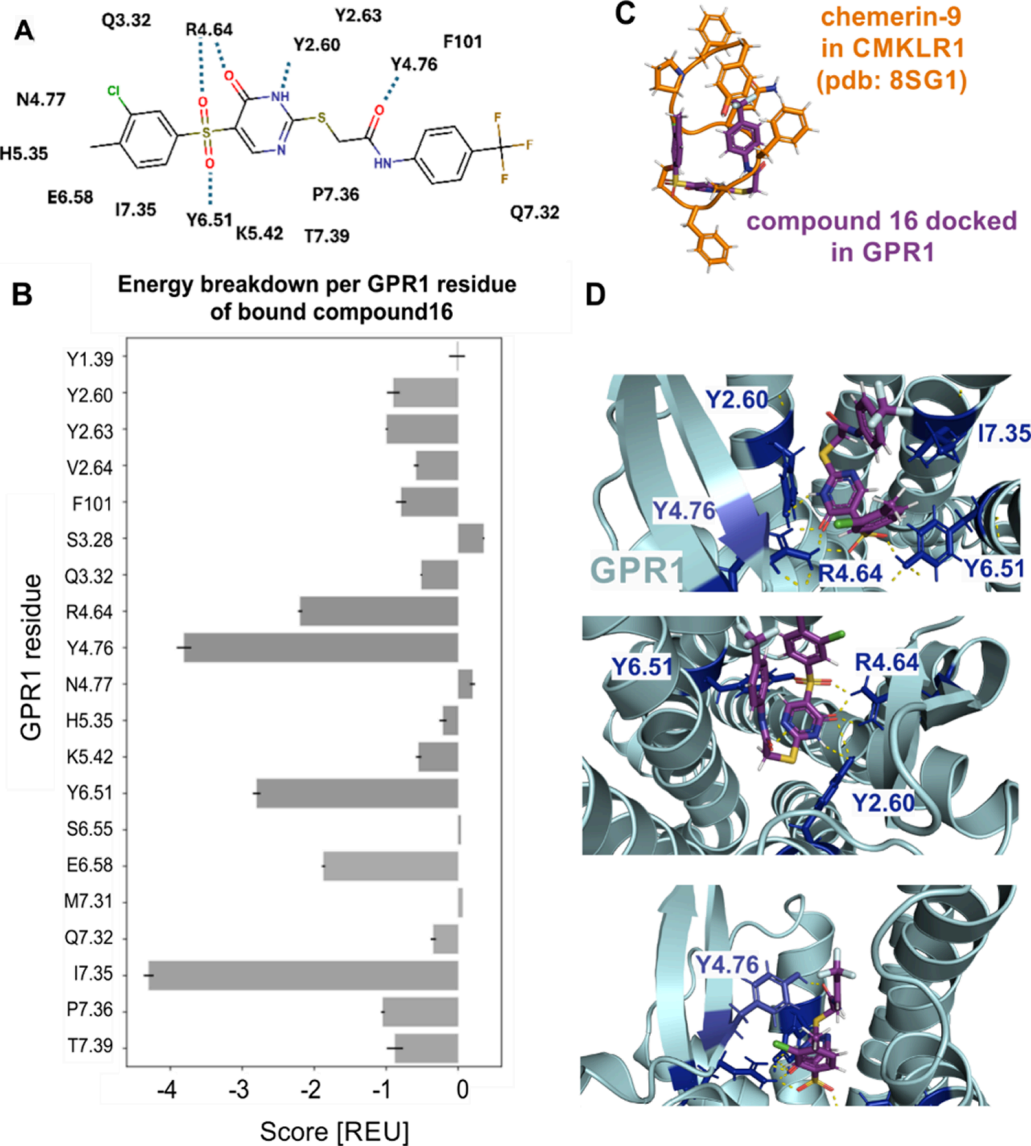
Model of compound **16** binding into an inactive GPR1 structure. (A) Schematic representation of interactions between GPR1 residues and compound **16**. (B) Energy breakdown per residues was created by using RosettaLigand. The bar graph represents the results (means ± SEM) from the three best fitting models. The GPR1 residues were named based on the Ballesteros–Weinstein nomenclature,^53^ whereby GPR1 residues of the N and C terminus or the loops were named according to their position. (C) Orientation of chemerin-9 from the cryo-EM structure (PDB 8SG1, orange) in comparison to compound **16** docked in GPR1 (violet). (D) Top and side view into the inactive GPR1 (aquamarine) structure. The model illustrates the inactive receptor structures based on the AlphaFold2 structure. Residues in blue are the relevant residues for compound **16** interaction at GPR1. Polar interactions are presented as yellow dashed lines.

The orientation of compound **16** at CMKLR1 and GPR1 is very similar and interacts in the same orthosteric binding pocket (Figure 9A). All receptor residues that interact with compound **16** and are found in both, CMKLR1 and GPR1, are summarized in Figure 9B. Several residues, as Y/F2.68, L/Q3.32, N4.77, H5.35, S/Q7.32, and P7.36, contribute equally to CMKLR1 and GPR1, whereas some residues interact more with CMKLR1 (Y2.63, R.4.64, N6.55, M7.31, L7.35, and T7.39) and some residues more with GPR1 (Y4.76, K5.42, Y6.51, and E6.58).

**Figure 9:**
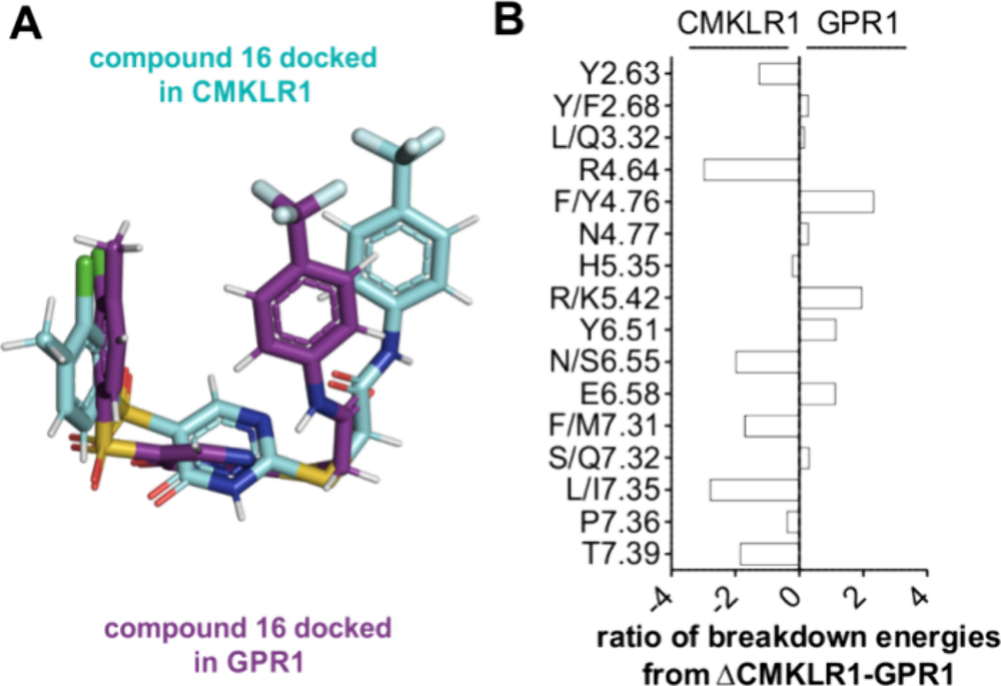
Comparison of compound **16** at both chemerin receptors CMKLR1 and GPR1. (A) Comparison of compound **16** docked in the CMKLR1 inactive model (cyan) and in the GPR1 inactive model (violet). (B) Energy breakdown per residues was created by using RosettaLigand of the three best fitting models. The mean of the energy breakdown per residue of GPR1 was subtracted from the mean of the CMKLR1 energy breakdown per residue. When CMKLR1 and GPR1 do not contain the same residue, the first letter belongs to CMKLR1 and the second letter to GPR1. Negative values describe the preference for CMKLR1 and positive values the preference for GPR1 interaction. Residues interacting with only one receptor are not shown.

## Conclusions

We investigated the antagonistic effect of a novel set of compounds at human CMKLR1. SAR studies resulted in the identification of the micromolar active antagonist **16** at CMKLR1 with a p*A*_2_ value of 5.96 in G protein signaling. This small molecule has been observed to inhibit the arrestin-3 recruitment at CMKLR1 and GPR1. Dual targeting could enhance the effect, particularly in the context of applications targeting tissues where both chemerin receptors are expressed, such as in skin tissues for psoriasis patients. In this study, we have combined SAR, receptor mutagenesis, and molecular modeling to clarify the binding mode of an antagonist at the chemerin receptor. For the very first time, we described the binding mode of an antagonist at CMKLR1, verified by mutagenesis experiments. All observed *in vitro* results were found to be in alignment with the computational CMKLR1 model. The slightly reduced impact of compound **16** on C9-mediated GPR1 activation could occur because the nonapeptide C9 is bound more strongly at GPR1 than at CMKLR1. A similar binding mode of compound **16** at GPR1 and CMKLR1 is supported by *in silico* modeling. Our research contributes to the understanding of antagonistic interactions within the binding pockets of chemerin receptors. This increases the possibility of treating symptoms of type 2 diabetes or obesity in general with linkage to inflammation.

## Experimental Section

### Compound Synthesis

Unless otherwise specified, all reagents and solvents were purchased from commercial suppliers and used without further purification. Dry ethanol and dry sodium ethanolate were purchased from Thermo Scientific in sealed glass bottles. Proton, carbon, and fluorine NMR spectra were recorded using a Varian MERCURY PLUS or a Bruker ADVANCE III HD 400 (frequencies: ^1^H, 400 and 300 MHz; ^19^F, 282 and 376 MHz; ^13^C­{^1^H}, 101 and 75 MHz). Chemical shifts (δ) were reported in parts per million (ppm) and for proton and carbon NMR relative to the solvent residual signals CDCl_3_ (^1^H: 7.26 ppm, ^13^C: 77.00 ppm) and DMSO-*d*_6_ (^1^H: 2.50 ppm, ^13^C­{^1^H}: 39.52 ppm). NMR raw data were processed using Mestre Lab MESTRENOVA. NMR data are reported as follows: chemical shift, multiplicity (s = singlet, d = doublet, br s = broad singlet, t = triplet, q = quartet, hept = septet, m = multiplet, dd = doublet of doublets), coupling constants (Hz), and for 1H integrals. High-resolution masses were obtained using a Bruker Daltonics MicrOTOF (HR-MS (ESI)). Low-resolution masses were obtained using an Agilent 8890/5977B GC/MSD (LR-MS (EI)) or an Advion expression (LR-MS (ESI)). Reaction monitoring was performed with thin-layer chromatography, using precoated plates from Merck (silica gel 60, F^254^), and RP-HPLC using a Thermo Fisher Scientific ULTIMATE 3000. The purity of all compounds of >95% was determined prior to biological testing by HPLC analysis using the previously mentioned HPLC system with a Macherey-Nagel 100-5 C18ec column (5 μm, 250 × 4.6 mm), eluted with a linear gradient solvent system (CH_3_CN/H_2_O). Purification of reaction products by column chromatography was carried out using Teledyne Isco COMBIFLASH 300+ and REDISEP RF SILICA GEL disposal flash columns.

#### 1,2-Bis­(3-chloro-4-methylphenyl)­disulfan (**X2**)

Based on the patent from BASF AG (WO 2020/074964 A1),^34^ 3-chloro-4-methylaniline (**X1**, 9.0 mL, 75.0 mmol, 1.0 equiv) was dissolved in ice (20.3 g) and conc. HCl (37%, 9.75 mL). At 0 °C, a solution of NaNO_2_ (5.23 g, 75.75 mmol, 1.01 equiv) in H_2_O (50 mL) was slowly added. The reaction was stirred at 0 °C for 1 h. In a second flask, a solution of Na_2_CO_3_ (12.15 g, 112.5 mmol, 1.5 equiv) and potassium *O*-ethyl-carbonodithioate (15.63 g, 97.5 mmol, 1.3 equiv) in 37.5 mL of H_2_O was prepared and heated to 70 °C. The diazonium suspension was slowly added to the solution, and the resulting mixture was stirred at 70 °C for 2 h. A solution of NaOH (12.0 g, 300 mmol, 4.0 equiv) in 300 mL of EtOH was added, and the reaction was stirred overnight at 100 °C. The solution was concentrated in vacuo, and the resulting crude product was neutralized with H_2_O and aq. HCl. The suspension was extracted with dichloromethane. The combined organic phases were dried over Na_2_SO_4_ and concentrated in vacuo. The crude product was purified by column chromatography (isocratic 100% dichloromethane) to obtain **X2** as a yellow solid (6.26 g, 53% yield). ^1^H NMR (CDCl_3_, 400 MHz): δ 7.55 (d, *J* = 1.9 Hz, 2H), 7.38–7.34 (m, 2H), 7.27–7.22 (m, 2H), 2.44 (s, 6H) ppm. ^13^C­{^1^H} NMR (101 MHz, CDCl_3_): δ 135.8 (2C), 135.6 (2C), 135.2 (2C), 131.6 (2C), 128.6 (2C), 126.6 (2C), 19.9 (2C) ppm. LR-MS (EI) *m*/*z* calc. for (M^+•^): 313.9, found: 314.0.

#### Ethyl 2-[(3-Chloro-4-methylphenyl)­sulfanyl]­acetate (**X4**)

**X2** (6.54 g, 20.75 mmol, 1.0 equiv) was dissolved in 250 mL of a 1:1 mixture of THF/EtOH. NaBH_4_ (5.89 g, 155.65 mmol, 7.5 equiv) was added at 0 °C. The reaction was warmed to RT and stirred for 48 h to receive **X3**. Without isolation, ethyl 2-bromoacetate (6.93 mL, 62.25 mmol, 1.5 equiv) was added, and the solution was stirred for 5 days. The reaction was neutralized with aq. HCl and extracted with ethyl acetate. The combined extracts were washed with brine, dried over Na_2_SO_4_, filtered, and concentrated in vacuo. The crude product was purified by column chromatography (cyclohexane/dichloromethane 3:1) to obtain **X4** as a yellow liquid (7.29 g, 69% yield). ^1^H NMR (400 MHz, CDCl_3_): δ 7.42 (d, *J* = 1.9 Hz, 1H), 7.21 (dd, *J* = 8.0, 1.9 Hz, 1H), 7.15 (dd, *J* = 7.8, 0.8 Hz, 1H), 4.17 (q, *J* = 7.1 Hz, 2H), 3.59 (s, 2H), 2.34 (s, 3H), 1.24 (t, *J* = 7.1 Hz, 3H) ppm. ^13^C­{^1^H} NMR (101 MHz, CDCl_3_): δ 169.6, 135.3, 134.9, 133.6, 131.5, 130.8, 128.9, 61.8, 37.2, 19.8, 14.2 ppm. LR-MS (EI) *m*/*z* calc. for (M^+•^): 244.0, found: 244.1.

#### Ethyl 2-(3-Chloro-4-methylbenzenesulfonyl)­acetate (**X5**)

**X4** (6.19 g, 25.31 mmol, 1.0 equiv) was dissolved in 250 mL of DCM. mCPBA (20.38 g, 88.59 mmol, 75%, 3.5 equiv) was added at 0 °C. The reaction was stirred for 3 h, warmed to RT, and stirred for 3 days. The reaction mixture was washed with saturated aq. Na_2_CO_3_ and brine, dried over Na_2_SO_4_, filtered, and concentrated in vacuo to obtain the product **X5** as a yellow liquid (5.75 g, 82% yield). ^1^H NMR (400 MHz, CDCl_3_): δ 7.91 (d, *J* = 1.9 Hz, 1H), 7.72 (dd, *J* = 8.0, 1.9 Hz, 1H), 7.44 (dd, *J* = 8.0, 0.9 Hz, 1H), 4.16 (q, *J* = 7.2 Hz, 2H), 4.10 (s, 2H), 2.47 (s, 3H), 1.22 (t, *J* = 7.1 Hz, 3H) ppm. ^13^C­{^1^H} NMR (101 MHz, CDCl_3_): δ 162.3, 143.6, 137.7, 135.5, 131.7, 129.3, 62.6, 61.2, 20.6, 14.0 ppm. LR-MS (EI) *m*/*z* calc. for (M^+•^): 276.0, found: 276.0.

#### Ethyl (2*E*)-2-(3-Chloro-4-methylbenzenesulfonyl)-3-ethoxyprop-2-enoate (**X6**)

Based on ref 35, **X5** (5.75 g, 20.76 mmol, 1.0 equiv) was dissolved in acetic anhydride (39.25 mL, 415.2 mmol, 20.0 equiv), and ethyl orthoformate (68.75 mL, 415.2 mmol, 20.0 equiv) and ZnCl_2_ (0.85 g, 6.23 mmol, 0.3 equiv) were added. The reaction vessel was closed, and the reaction was stirred at 140 °C under an argon atmosphere. After 24 h, additional acetic anhydride (19.63 mL, 207.6 mmol, 10.0 equiv) and triethyl orthoformate (34.38 mL, 207.6 mmol, 10.0 equiv) were added and stirred under the same conditions for 4 days. After cooling to room temperature, water and saturated aq. Na_2_CO_3_ were added, and the mixture was extracted with ethyl acetate, dried over Na_2_SO_4_, filtered, and concentrated in vacuo. The crude product was purified by column chromatography (cyclohexane/ethyl acetate 3:1) to obtain **X6** as a yellow solid (2.76 g, 40% yield). ^1^H NMR (400 MHz, CDCl_3_): δ 8.09 (s, 1H), 7.89 (d, *J* = 1.9 Hz, 1H), 7.71 (dd, *J* = 8.0, 1.9 Hz, 1H), 7.35 (d, *J* = 8.0 Hz, 1H), 4.38 (q, *J* = 7.2 Hz, 2H), 4.14 (q, *J* = 7.1 Hz, 2H), 2.43 (s, 3H), 1.46 (t, *J* = 7.1 Hz, 3H), 1.19 (t, *J* = 7.1 Hz, 3H) ppm. ^13^C­{^1^H} NMR (101 MHz, CDCl_3_): δ 168.6, 160.4, 141.9, 140.4, 134.8, 131.1, 129.0, 126.5, 113.6, 74.8, 61.2, 20.5, 15.4, 14.0 ppm. HR-MS (ESI) calc. for [M + H]^+^: 333.0558, found: 333.0554.

#### 5-(3-Chloro-4-methylbenzenesulfonyl)-2-sulfanyl-3,4-dihydropyrimidin-4-one (**X7**)

Based on ref 35, **X6** (4.65 g, 13.98 mmol, 1.0 equiv) was dissolved in 120 mL of dry EtOH, and thiourea (1.17 g, 15.38 mmol, 1.1 equiv) and NaOEt (15,38 mL, 15.38 mmol, 1.1 equiv, 1 M in EtOH) were added. The solution was stirred at RT under an argon atmosphere for 16 h. The reaction mixture was filtered to obtain **X7** as its sodium salt as a yellow solid (2.34 g, 49% yield). The filtrate was concentrated *in vacuo*. The crude product was dissolved in water and precipitated with 1 M aq. HCl and filtered to obtain the product **X7** as a brown solid (2.02 g, 45% yield). ^1^H NMR (400 MHz, DMSO-*d*_6_): δ 11.42 (br s, 1H), 8.10 (s, 1H), 7.90 (d, *J* = 1.9 Hz, 1H), 7.72 (dd, *J* = 8.0, 1.9 Hz, 1H), 7.51 (d, *J* = 8.1 Hz, 1H), 2.37 (s, 3H) ppm. ^13^C­{^1^H} NMR (101 MHz, DMSO-*d*_6_): δ 187.1, 158.6, 157.0, 142.1, 141.0, 133.6, 131.9, 127.9, 126.2, 113.2, 20.1 ppm. HR-MS (ESI) calc. for [M + H]^+^: 316.9816, found: 316.9831.

#### 2-{[5-(3-Chloro-4-methylbenzenesulfonyl)-4-oxo-1,4-dihydropyrimidin-2-yl]­sulfanyl}-*N*-(2-methoxy-5-methyl-phenyl)­acetamide (Compound **1**, **VU0514009**)

Based on ref 35, **X7** (120 mg, 0.38 mmol, 1.0 equiv) was dissolved in 10 mL of a 1:1 mixture of ACN/THF. **X8a** (98 mg, 0.38 mmol, 1.0 equiv) and Na_2_CO_3_ (30 mg, 0.28 mmol, 0.75 equiv) were added, and the reaction was stirred at 35 °C for 16 h. The solution was concentrated in vacuo. The crude product was purified by column chromatography (dichloromethane/methanole 95:5) to obtain compound **1** as a yellow solid (55 mg, 29% yield). ^1^H NMR (300 MHz, DMSO-*d*_6_): δ 9.53 (br s, 1H), 8.54 (s, 1H), 7.97 (d, *J* = 1.9 Hz, 1H), 7.82 (d, *J* = 1.9 Hz, 1H), 7.64–7.55 (m, 1H), 6.91 (d, *J* = 8.4 Hz, 1H), 6.86 (dd, *J* = 8.4, 2.0 Hz, 1H), 4.18 (s, 2H), 3.77 (s, 3H), 2.40 (s, 3H), 2.20 (s, 3H) ppm. ^13^C­{^1^H} NMR (75 MHz, DMSO-*d*_6_): δ 168.5, 165.4, 157.0, 147.0, 142.2, 139.1, 133.6, 131.8, 131.8, 129.1, 128.2, 126.8, 126.7, 124.6, 121.7, 121.1, 111.0, 55.7, 35.0, 20.5, 19.8 ppm. HR-MS (ESI) calc. for [M + H]^+^: 494.0606, found: 494.0599.

#### 2-{[5-(3-Chloro-4-methylbenzenesulfonyl)-4-oxo-1,4-dihydropyrimidin-2-yl]­sulfanyl}-*N*-(2,5-dimethoxyphenyl)­acetamide (Compound **2**)

Compound **2** was synthesized from **X7** and **X8b** following the procedure described for compound **1** as a gray-green solid (79% yield). ^1^H NMR (400 MHz, DMSO-*d*_6_): δ 9.96 (br s, 1H), 8.31 (s, 1H), 8.00 (d, *J* = 1.8 Hz, 1H), 7.87 (d, *J* = 3.0 Hz, 1H), 7.77 (dd, *J* = 8.0, 1.9 Hz, 1H), 7.50 (d, *J* = 8.0 Hz, 1H), 6.84 (d, *J* = 8.9 Hz, 1H), 6.55 (dd, *J* = 8.9, 3.1 Hz, 1H), 3.74 (s, 2H), 3.66 (s, 3H), 3.52 (s, 3H), 2.38 (s, 3H) ppm. ^13^C­{^1^H} NMR (101 MHz, DMSO-*d*_6_): δ 173.1, 168.6, 164.8, 156.1, 152.9, 142.3, 141.4, 140.6, 133.0, 131.3, 128.4, 128.1, 126.2, 117.6, 111.1, 107.2, 106.4, 55.6, 55.3, 34.7, 19.7 ppm. HR-MS (ESI) calc. for [M + H]^+^: 510.0555, found: 510.0545.

#### 2-{[5-(3-Chloro-4-methylbenzenesulfonyl)-4-oxo-1,4-dihydropyrimidin-2-yl]­sulfanyl}-*N*-(5-fluoro-2-methylphenyl)­acetamide (Compound **3**)

Compound **3** was synthesized from **X7** and **X8c** following the procedure described for compound **1** as a white solid (98% yield). ^1^H NMR (300 MHz, DMSO-*d*_6_): δ 9.81 (br s, 1H), 8.26 (d, *J* = 1.7 Hz, 1H), 7.96 (d, *J* = 1.8 Hz, 1H), 7.74 (dd, *J* = 8.0, 1.9 Hz, 1H), 7.57 (dd, *J* = 11.4, 2.8 Hz, 1H), 7.50 (d, *J* = 8.0 Hz, 1H), 7.15 (dd, *J* = 8.5, 6.6 Hz, 1H), 6.84 (td, *J* = 8.4, 2.8 Hz, 1H), 3.80 (s, 2H), 2.38 (s, 3H), 2.03 (s, 3H) ppm. ^13^C­{^1^H} NMR (75 MHz, DMSO-*d*_6_): δ 173.5, 168.4, 164.8, 160.1 (d, *J* = 239.4 Hz), 156.2, 141.4, 140.5, 137.6 (d, *J* = 10.9 Hz), 133.0, 131.3, 131.3 (d, *J* = 9.1 Hz), 127.8, 126.0, 124.9, 117.5, 110.6 (d, *J* = 21.0 Hz), 109.2 (d, *J* = 25.8 Hz), 34.7, 19.7, 16.9 ppm. ^19^F NMR (282 MHz, DMSO-*d*_6_): δ −116.34 (q, *J* = 8.7 Hz) ppm. HR-MS (ESI) calc. for [M + H]^+^: 482.0406, found: 482.0400.

#### 2-{[5-(3-Chloro-4-methylbenzenesulfonyl)-4-oxo-1,4-dihydropyrimidin-2-yl]­sulfanyl}-*N*-(2-methoxyphenyl)­acetamide (Compound **4**)

Compound **4** was synthesized from **X7** and **X8d** following the procedure described for compound **1** as a white solid (77% yield). ^1^H NMR (300 MHz, DMSO-*d*_6_): δ 9.96 (br s, 1H), 8.31 (s, 1H), 8.15 (dd, *J* = 8.0, 1.6 Hz, 1H), 8.01 (d, *J* = 1.8 Hz, 1H), 7.78 (dd, *J* = 8.0, 1.9 Hz, 1H), 7.51 (d, *J* = 8.0 Hz, 1H), 7.05–6.93 (m, 1H), 6.95–6.89 (m, 1H), 6.89–6.82 (m, 1H), 3.74 (s, 2H), 3.57 (s, 3H), 2.38 (s, 3H) ppm. ^13^C­{^1^H} NMR (75 MHz, DMSO-*d*_6_): δ 173.1, 168.4, 164.7, 156.1, 148.1, 141.4, 140.6, 133.0, 131.3, 128.1, 127.7, 126.2, 123.6, 120.2, 119.4, 117.6, 110.6, 55.1, 34.6, 19.7 ppm. HR-MS (ESI) calc. for [M + H]^+^: 480.0450, found: 518.0441.

#### 2-{[5-(3-Chloro-4-methylbenzenesulfonyl)-4-oxo-1,4-dihydropyrimidin-2-yl]­sulfanyl}-*N*-(4-methoxyphenyl)­acetamide (Compound **5**)

Compound **5** was synthesized from **X7** and **X8e** following the procedure described for compound **1** as a white solid (54% yield). ^1^H NMR (400 MHz, DMSO-*d*_6_): δ 10.57 (br s, 1H), 8.23 (s, 1H), 7.99 (d, *J* = 1.9 Hz, 1H), 7.75 (dd, *J* = 8.0, 1.9 Hz, 1H), 7.50 (d, *J* = 8.1 Hz, 1H), 7.46 (d, *J* = 9.0 Hz, 1H), 6.84 (d, *J* = 9.0 Hz, 2H), 3.75 (s, 2H), 3.70 (s, 3H), 2.38 (s, 3H) ppm. ^13^C­{^1^H} NMR (101 MHz, DMSO-*d*_6_): δ 173.7, 166.9, 164.8, 156.1, 155.1, 141.5, 140.5, 133.0, 132.3, 131.3, 127.8, 126.0, 120.3 (2C), 117.3, 113.9 (2C), 55.2, 35.1, 19.7 ppm. HR-MS (ESI) calc. for [M + H]^+^: 480.0449, found: 480.0445.

#### 2-{[5-(3-Chloro-4-methylbenzenesulfonyl)-4-oxo-1,4-dihydropyrimidin-2-yl]­sulfanyl}-*N*-(3-methylphenyl)­acetamide (Compound **6**)

Compound **6** was synthesized from **X7** and **X8f** following the procedure described for compound **1** as a white solid (89% yield). ^1^H NMR (400 MHz, DMSO-*d*_6_): δ 10.66 (br s, 1H), 8.25 (s, 1H), 7.99 (d, *J* = 1.8 Hz, 1H), 7.76 (dd, *J* = 8.0, 1.9 Hz, 1H), 7.50 (d, *J* = 8.0 Hz, 1H), 7.36 (t, *J* = 1.9 Hz, 1H), 7.32 (dd, *J* = 8.0, 2.3 Hz, 1H), 7.13 (t, *J* = 7.8 Hz, 1H), 6.87–6.80 (m, 1H), 3.76 (s, 2H), 2.37 (s, 3H), 2.22 (s, 3H) ppm. ^13^C­{^1^H} NMR (101 MHz, DMSO-*d*_6_): δ 173.7, 167.5, 164.8, 156.2, 141.4, 140.5, 138.9, 137.9, 133.0, 131.3, 128.6, 127.9, 126.1, 123.9, 119.4, 117.3, 116.1, 35.2, 21.1, 19.7 ppm. HR-MS (ESI) calc. for [M + H]^+^: 464.0501, found: 518.0483.

#### 2-{[5-(3-Chloro-4-methylbenzenesulfonyl)-4-oxo-1,4-dihydropyrimidin-2-yl]­sulfanyl}-*N*-[2-(propan-2-yl)­phenyl]­acetamide (Compound **7**)

Compound **7** was synthesized from **X7** and **X8g** following the procedure described for compound **1** as a green-yellow solid (92% yield). ^1^H NMR (300 MHz, DMSO-*d*_6_): δ 9.70 (br s, 1H), 8.28 (s, 1H), 7.96 (d, *J* = 1.8 Hz, 1H), 7.75 (dd, *J* = 8.0, 1.9 Hz, 1H), 7.54–7.41 (m, 2H), 7.21 (dd, *J* = 5.8, 3.6 Hz, 1H), 7.17–7.05 (m, 2H), 3.79 (s, 2H), 2.98 (hept, *J* = 6.7 Hz, 1H), 2.37 (s, 3H), 0.89 (d, *J* = 6.8 Hz, 6H) ppm. ^13^C­{^1^H} NMR (75 MHz, DMSO-*d*_6_): δ 173.5, 168.4, 164.6, 156.1, 141.3, 141.2, 140.6, 134.8, 133.1, 131.3, 128.0, 126.2, 125.7, 125.5, 125.3, 125.0, 117.6, 34.5, 26.9, 22.7 (2C), 19.7 ppm. HR-MS (ESI) calc. for [M + H]^+^: 492.0814, found: 492.0819.

#### 2-{[5-(3-Chloro-4-methylbenzenesulfonyl)-4-oxo-1,4-dihydropyrimidin-2-yl]­sulfanyl}-*N*-[3-(propan-2-yl)­phenyl]­acetamide (Compound **8**)

Compound **8** was synthesized from **X7** and **X8h** following the procedure described for compound **1** as a yellow solid (96% yield). ^1^H NMR (400 MHz, DMSO-*d*_6_): δ 10.72 (br s, 1H), 8.27 (s, 1H), 7.98 (d, *J* = 1.8 Hz, 1H), 7.76 (dd, *J* = 8.0, 1.9 Hz, 1H), 7.50 (d, *J* = 8.1 Hz, 1H), 7.44–7.36 (m, 2H), 7.16 (dd, *J* = 8.6, 7.7 Hz, 1H), 6.89 (dt, *J* = 7.8, 1.4 Hz, 1H), 3.79 (s, 2H), 2.78 (h, *J* = 6.9 Hz, 1H), 2.37 (s, 3H), 1.13 (d, *J* = 6.9 Hz, 6H) ppm. ^13^C­{^1^H} NMR (101 MHz, DMSO-*d*_6_): δ 173.3, 167.2, 164.2, 156.1, 149.1, 141.3, 140.6, 139.0, 133.1, 131.3, 128.6, 127.9, 126.1, 121.4, 117.7, 116.9, 116.4, 35.2, 33.5, 23.8 (2C), 19.7 ppm. HR-MS (ESI) calc. for [M + H]^+^: 492.0814, found: 492.0805.

#### 2-{[5-(3-Chloro-4-methylbenzenesulfonyl)-4-oxo-1,4-dihydropyrimidin-2-yl]­sulfanyl}-*N*-[4-(propan-2-yl)­phenyl]­acetamide (Compound **9**)

Compound **9** was synthesized from **X7** and **X8i** following the procedure described for compound **1** as a brown solid (95% yield). ^1^H NMR (400 MHz, DMSO-*d*_6_): δ 10.62 (br s, 1H), 8.26 (s, 1H), 7.99 (d, *J* = 1.8 Hz, 1H), 7.76 (dd, *J* = 8.0, 1.9 Hz, 1H), 7.50 (d, *J* = 8.1 Hz, 1H), 7.44 (d, *J* = 8.5 Hz, 2H), 7.11 (d, *J* = 8.5 Hz, 2H), 3.77 (s, 2H), 2.81 (hept, *J* = 6.9 Hz, 1H), 2.38 (s, 3H), 1.15 (d, *J* = 6.9 Hz, 6H) ppm. ^13^C­{^1^H} NMR (101 MHz, DMSO-*d*_6_): δ 173.6, 167.2, 164.7, 156.2, 143.3, 141.4, 140.6, 136.8, 133.1, 131.3, 127.9, 126.4 (2C), 126.1, 119.0 (2C), 117.4, 35.1, 32.9, 23.9 (2C), 19.7 ppm. HR-MS (ESI) calc. for [M + H]^+^: 492.0814, found: 492.0815.

#### *N*-{[1,1′-Biphenyl]-4-yl}-2-{[5-(3-chloro-4-methylbenzenesulfonyl)-4-oxo-1,4-dihydro-pyrimidin-2-yl]­sulfanyl}­acetamide (Compound **10**)

Compound **10** was synthesized from **X7** and **X8j** following the procedure described for compound **1** as a yellow solid (48% yield). ^1^H NMR (400 MHz, DMSO-*d*_6_): δ 10.66 (br s, 1H), 8.39 (d, *J* = 1.6 Hz, 1H), 7.98 (d, *J* = 1.9 Hz, 1H), 7.79 (dd, *J* = 8.0, 1.9 Hz, 1H), 7.69–7.58 (m, 6H), 7.54 (d, *J* = 8.5 Hz, 1H), 7.47–7.39 (m, 2H), 7.35–7.29 (m, 1H), 4.02 (s, 2H), 2.38 (s, 3H) ppm. ^13^C­{^1^H} NMR (101 MHz, DMSO-*d*_6_): δ 171.2, 166.3, 161.1, 155.7, 141.4, 140.3, 139.7, 138.4, 135.1, 133.3, 131.6, 128.9 (2C), 128.0, 127.1, 127.0 (2C), 126.4, 126.3 (2C), 119.5 (2C), 119.2, 35.4, 19.8. HR-MS (ESI) calc. for [M + H]^+^: 526.0657, found: 496.0660.

#### 2-{[5-(3-Chloro-4-methylbenzenesulfonyl)-4-oxo-1,4-dihydropyrimidin-2-yl]­sulfanyl}-*N*-(2-fluorophenyl)­acetamide (Compound **11**)

Compound **11** was synthesized from **X7** and **X8k** following the procedure described for compound **1** as a yellow solid (19% yield). ^1^H NMR (400 MHz, DMSO-*d*_6_): δ 10.23 (br s, 1H), 8.38 (s, 1H), 7.96 (d, *J* = 1.9 Hz, 1H), 7.94–7.86 (m, 1H), 7.78 (dd, *J* = 8.0, 1.9 Hz, 1H), 7.54 (d, *J* = 8.1 Hz, 1H), 7.30–7.19 (m, 1H), 7.17–7.10 (m, 2H), 4.05 (s, 2H), 2.39 (s, 3H) ppm. ^13^C­{^1^H} NMR (101 MHz, DMSO-*d*_6_): δ 170.9, 166.8, 160.9, 155.5, 153.2 (d, *J* = 245.2 Hz), 141.3, 140.2, 133.3, 131.5, 128.0, 126.4, 126.1 (d, *J* = 11.5 Hz), 125.2 (d, *J* = 7.6 Hz), 124.4 (d, *J* = 3.5 Hz), 123.4, 119.3, 115.4 (d, *J* = 19.3 Hz), 34.8, 19.7 ppm. ^19^F NMR (377 MHz, DMSO-*d*_6_): δ −125.37 ppm. HR-MS (ESI) calc. for [M-H]^−^: 466.0103, found: 466.0087.

#### 2-{[5-(3-Chloro-4-methylbenzenesulfonyl)-4-oxo-1,4-dihydropyrimidin-2-yl]­sulfanyl}-*N*-(3-fluorophenyl)­acetamide (Compound **12**)

Compound **12** was synthesized from **X7** and **X8l** following the procedure described for compound **1** as a yellow solid (32% yield).^8^ ^1^H NMR (300 MHz, DMSO-*d*_6_): δ 11.03 (br s, 1H), 8.25 (s, 1H), 7.98 (d, *J* = 1.9 Hz, 1H), 7.76 (dd, *J* = 8.0, 1.9 Hz, 1H), 7.58–7.52 (m, 1H), 7.50 (d, *J* = 8.1 Hz, 1H), 7.35–7.23 (m, 2H), 6.91–6.78 (m, 1H), 3.80 (s, 2H), 2.37 (s, 3H) ppm. ^13^C­{^1^H} NMR (75 MHz, DMSO-*d*_6_): δ 173.4, 167.8, 164.6, 162.2 (d, *J* = 241.3 Hz), 156.1, 141.4, 140.7 (d, *J* = 11.2 Hz), 140.6, 133.0, 131.3, 130.4 (d, *J* = 9.4 Hz), 127.8, 126.1, 117.5, 114.7 (d, *J* = 2.4 Hz), 109.7 (d, *J* = 21.2 Hz), 105.7 (d, *J* = 26.3 Hz), 35.2, 19.7 ppm. ^19^F NMR (282 MHz, DMSO-*d*_6_): δ −111.95 (dt, *J* = 15.4, 7.3 Hz) ppm. HR-MS (ESI) calc. for [M + H]^+^: 468.0250, found: 468.0258.

#### 2-{[5-(3-Chloro-4-methylbenzenesulfonyl)-4-oxo-1,4-dihydropyrimidin-2-yl]­sulfanyl}-*N*-(4-fluorophenyl)­acetamide (Compound **13**)

Compound **13** was synthesized from **X7** and **X8m** following the procedure described for compound **1** as a white solid (19% yield). ^1^H NMR (400 MHz, DMSO-*d*_6_): δ 10.83 (br s, 1H), 8.23 (s, 1H), 7.98 (d, *J* = 1.9 Hz, 1H), 7.75 (dd, *J* = 8.0, 1.9 Hz, 1H), 7.57 (dd, *J* = 8.9, 5.1 Hz, 2H), 7.50 (d, *J* = 8.1 Hz, 1H), 7.10 (t, *J* = 8.9 Hz, 2H), 3.77 (s, 2H), 2.38 (s, 3H) ppm. ^13^C­{^1^H} NMR (101 MHz, DMSO-*d*_6_): δ 173.6, 167.4, 164.8, 157.9 (d, *J* = 239.5 Hz), 156.1, 141.5, 140.5, 135.5 (d, *J* = 2.4 Hz), 133.0, 131.3, 127.8, 126.0, 120.5 (d, *J* = 7.8 Hz, 2C), 117.3, 115.3 (d, *J* = 22.2 Hz, 2C), 35.1, 19.7 ppm. ^19^F NMR (376 MHz, DMSO-*d*_6_): δ −119.44 (dq, *J* = 9.2, 5.4, 4.6 Hz) ppm. HR-MS (ESI) calc. for [M-H]^−^: 466.0103, found: 466.0090.

#### 2-{[5-(3-Chloro-4-methylbenzenesulfonyl)-4-oxo-1,4-dihydropyrimidin-2-yl]­sulfanyl}-*N*-(4-nitrophenyl)­acetamide (Compound **14**)

Compound **14** was synthesized from **X7** and **X8n** following the procedure described for compound **1** as a yellow solid (98% yield). ^1^H NMR (300 MHz, DMSO-*d*_6_): δ 11.45 (br s, 1H), 8.23 (s, 1H), 8.17 (d, *J* = 9.2 Hz, 1H), 7.99 (d, *J* = 1.9 Hz, 1H), 7.79 (d, *J* = 9.3 Hz, 2H), 7.75 (dd, *J* = 8.0, 1.9 Hz, 1H), 7.50 (d, *J* = 8.1 Hz, 1H), 3.85 (s, 2H), 2.37 (s, 3H) ppm. ^13^C­{^1^H} NMR (75 MHz, DMSO-*d*_6_): δ 173.5, 168.4, 164.8, 156.2, 145.2, 142.1, 141.4, 140.5, 133.0, 131.3, 127.9, 126.0, 125.0 (2C), 118.6 (2C), 117.4, 35.3, 19.7 ppm. HR-MS (ESI) calc. for [M + H]^+^: 495.0195, found: 495.0206.

#### 2-{[5-(3-Chloro-4-methylbenzenesulfonyl)-4-oxo-1,4-dihydropyrimidin-2-yl]­sulfanyl}-*N*-[3-(trifluoromethyl)­phenyl]­acetamide (Compound **15**)

Compound **15** was synthesized from **X7** and **X8o** following the procedure described for compound **1** as a white solid (94% yield). ^1^H NMR (400 MHz, DMSO-*d*_6_): δ 11.14 (br s, 1H), 8.23 (s, 1H), 8.07 (s, 1H), 7.98 (s, 1H), 7.76 (d, *J* = 8.0 Hz, 1H), 7.72 (d, *J* = 8.1 Hz, 1H), 7.54–7.50 (m, 1H), 7.50–7.47 (m, 1H), 7.37 (d, *J* = 7.8 Hz, 1H), 3.82 (s, 2H), 2.37 (s, 3H) ppm. ^13^C­{^1^H} NMR (101 MHz, DMSO-*d*_6_): δ 173.6, 168.1, 164.8, 156.2, 141.5, 140.5, 139.8, 133.0, 131.2, 130.0, 129.5 (d, *J* = 31.4 Hz), 127.8, 126.0, 124.0 (d, *J* = 272.5 Hz), 122.4, 119.5, 117.3, 114.9, 35.2, 19.7. ^19^F NMR (376 MHz, DMSO-*d*_6_): δ −61.42 ppm. HR-MS (ESI) calc. for [M + H]^+^: 518.0218, found: 518.0231.

#### 2-{[5-(3-Chloro-4-methylbenzenesulfonyl)-4-oxo-1,4-dihydropyrimidin-2-yl]­sulfanyl}-*N*-[4-(trifluoromethyl)­phenyl]­acetamide (Compound **16**)

Compound **16** was synthesized from **X7** and **X8p** following the procedure described for compound **1** as a white solid (98% yield). ^1^H NMR (400 MHz, DMSO-*d*_6_): δ 11.15 (br s, 1H), 8.23 (s, 1H), 7.99 (d, *J* = 1.8 Hz, 1H), 7.76 (d, *J* = 8.1 Hz, 1H), 7.76 (d, *J* = 8.0 Hz, 1H), 7.62 (d, *J* = 8.6 Hz, 2H), 7.50 (d, *J* = 8.0 Hz, 1H), 3.82 (s, 2H), 2.37 (s, 3H) ppm. ^13^C­{^1^H} NMR (101 MHz, DMSO-*d*_6_): δ 173.5, 168.1, 164.7, 156.1, 142.5, 141.5, 140.5, 133.0, 131.3, 127.9, 126.0 (3C), 125.7 (d, *J* = 268.5 Hz), 123.2 (d, *J* = 32.4 Hz), 118.8 (2C), 117.4, 35.2, 19.6 ppm. ^19^F NMR (376 MHz, DMSO-*d*_6_): δ −60.33 ppm. HR-MS (ESI) calc. for [M + H]^+^: 518.0218, found: 518.0232.

#### 2-{[5-(3-Chloro-4-methylbenzenesulfonyl)-4-oxo-1,4-dihydropyrimidin-2-yl]­sulfanyl}-*N*-[2-(difluoromethoxy)­phenyl]­acetamide (Compound **17**)

Compound **17** was synthesized from **X7** and **X8q** following the procedure described for compound **1** as a white solid (94% yield). ^1^H NMR (300 MHz, DMSO-*d*_6_): δ 9.98 (br s, 1H), 8.37–8.27 (m, 1H), 8.16 (dd, *J* = 8.4, 1.8 Hz, 1H), 7.96 (d, *J* = 1.9 Hz, 1H), 7.76 (dd, *J* = 8.0, 1.9 Hz, 1H), 7.50 (d, *J* = 8.1 Hz, 1H), 7.23–7.17 (m, 2H), 7.10 (dd, *J* = 7.2, 2.0 Hz, 1H), 7.17–6.68 (m, 1H), 3.80 (s, 1H), 2.38 (s, 3H) ppm. ^13^C­{^1^H} NMR (75 MHz, DMSO-*d*_6_): δ 173.3, 168.5, 164.8, 156.1, 141.3, 140.6, 139.8, 133.0, 131.2, 130.3, 127.9, 126.2, 125.5, 124.1, 121.7, 119.4, 117.7, 115.9, 34.7, 19.7 ppm. ^19^F NMR (282 MHz, DMSO-*d*_6_): δ −82.33 (dd, *J* = 73.0, 6.7 Hz) ppm. HR-MS (ESI) calc. for [M + H]^+^: 516.0261, found: 516.0263.

#### 2-{[5-(3-Chloro-4-methylbenzenesulfonyl)-4-oxo-1,4-dihydropyrimidin-2-yl]­sulfanyl}-*N*-[4-(difluoromethoxy)­phenyl]­acetamide (Compound **18**)

Compound **18** was synthesized from **X7** and **X8r** following the procedure described for compound **1** as a pink solid (79% yield). ^1^H NMR (400 MHz, DMSO-*d*_6_): δ 10.84 (br s, 1H), 8.24 (d, *J* = 3.1 Hz, 1H), 7.99 (d, *J* = 1.8 Hz, 1H), 7.76 (dd, *J* = 8.0, 1.9 Hz, 1H), 7.63–7.54 (m, 2H), 7.50 (d, *J* = 8.1 Hz, 1H), 7.12 (t, *J* = 148.7 Hz, 1H), 7.12–7.05 (m, 2H), 3.78 (s, 1H), 2.37 (s, 3H) ppm. ^13^C­{^1^H} NMR (101 MHz, DMSO-*d*_6_): δ 173.6, 167.4, 164.8, 156.2, 146.4–146.1 (m), 141.5, 140.5, 136.4, 133.0, 131.3, 127.8, 126.0, 120.2 (2C), 119.6 (2C), 117.3, 116.5 (t, *J* = 257.6 Hz), 35.1, 19.7. ^19^F NMR (376 MHz, DMSO-*d*_6_): δ −81.45 (d, *J* = 74.2 Hz) ppm. HR-MS (ESI) calc. for [M + H]^+^: 516.0261, found: 516.0267.

#### 2-{[5-(3-Chloro-4-methylbenzenesulfonyl)-4-oxo-1,4-dihydropyrimidin-2-yl]­sulfanyl}-*N*-[2-(trifluoromethoxy)­phenyl]­acetamide (Compound **19**)

Compound **19** was synthesized from **X7** and **X8s** following the procedure described for compound **1** as a white solid (91% yield). ^1^H NMR (400 MHz, DMSO-*d*_6_): δ 10.27 (br s, 1H), 8.29 (s, 1H), 7.99 (dd, *J* = 8.4, 1.6 Hz, 1H), 7.94 (d, *J* = 1.9 Hz, 1H), 7.74 (dd, *J* = 8.0, 1.9 Hz, 1H), 7.50 (d, *J* = 8.0 Hz, 1H), 7.39–7.30 (m, 2H), 7.21 (td, *J* = 7.7, 1.7 Hz, 1H), 3.86 (s, 2H), 2.37 (s, 3H) ppm. ^13^C­{^1^H} NMR (75 MHz, DMSO-*d*_6_): δ 173.0, 168.3, 164.4, 156.0, 141.3, 140.6, 139.1, 133.1, 131.3, 130.9, 127.9, 127.7, 126.1, 125.2, 124.1, 121.4, 120.0 (d, *J* = 257.8 Hz), 117.7, 34.7, 19.7 ppm. ^19^F NMR (377 MHz, DMSO-*d*_6_): δ −56.99 ppm. HR-MS (ESI) calc. for [M + H]^+^: 534.0167, found: 534.0168.

#### 2-{[5-(3-Chloro-4-methylbenzenesulfonyl)-4-oxo-1,4-dihydropyrimidin-2-yl]­sulfanyl}-*N*-[4-(methylsulfanyl)­phenyl]­acetamide (Compound **20**)

Compound **20** was synthesized from **X7** and **X8t** following the procedure described for compound **1** as a white solid (82% yield). ^1^H NMR (400 MHz, DMSO-*d*_6_): δ 10.74 (br s, 1H), 8.25 (s, 1H), 7.99 (d, *J* = 1.9 Hz, 1H), 7.76 (dd, *J* = 7.9, 1.9 Hz, 1H), 7.55–7.47 (m, 3H), 7.22–7.14 (m, 2H), 3.78 (s, 2H), 2.42 (s, 3H), 2.37 (s, 3H) ppm. ^13^C­{^1^H} NMR (101 MHz, DMSO-*d*_6_): δ 173.6, 167.4, 164.8, 156.2, 141.4, 140.6, 136.6, 133.1, 131.8, 131.3, 127.9, 127.2 (2C), 126.1, 119.6 (2C), 117.4, 35.1, 19.7, 15.6 ppm. HR-MS (ESI) calc. for [M + H]^+^: 496.0221, found: 496.0219.

#### 3-{[5-(3-Chloro-4-methylbenzenesulfonyl)-4-oxo-1,4-dihydropyrimidin-2-yl]­sulfanyl}-*N*-(4-fluorophenyl)­propenamide (Compound **21**)

Compound **21** was synthesized from **X7** and **X8u** following the procedure described for compound **1** as a yellow solid (30% yield). ^1^H NMR (400 MHz, DMSO-*d*_6_): δ 10.04 (br s, 1H), 8.44 (s, 1H), 7.97 (d, *J* = 2.0 Hz, 1H), 7.80 (dd, *J* = 8.0, 2.0 Hz, 1H), 7.62–7.55 (m, 3H), 7.16–7.08 (m, 2H), 3.34 (t, *J* = 6.7 Hz, 2H), 2.76 (t, *J* = 6.7 Hz, 2H), 2.39 (s, 3H) ppm. ^13^C­{^1^H} NMR (101 MHz, DMSO-*d*_6_): δ 170.3, 169.1, 159.1, 156.7, 155.4, 141.6, 139.9, 135.4 (d, *J* = 2.7 Hz), 133.4, 131.6, 128.1, 126.5, 120.8 (d, *J* = 7.7 Hz, 2C), 119.6, 115.2 (d, *J* = 21.9 Hz, 2C), 35.7, 25.8, 19.8 ppm. ^19^F NMR (376 MHz, DMSO-*d*_6_): δ −119.48 (h, *J* = 9.7, 8.2 Hz) ppm. HR-MS (ESI) calc. for [M + H]^+^: 482.0406, found: 482.0404.

#### 3-{[5-(3-Chloro-4-methylbenzenesulfonyl)-4-oxo-1,4-dihydropyrimidin-2-yl]­sulfanyl}-*N*-[4-(trifluoromethyl)­phenyl]­propenamide (Compound **22**)

Compound **22** was synthesized from **X7** and **X8v** following the procedure described for compound **1** as a gray-green solid (50% yield). ^1^H NMR (300 MHz, DMSO-*d*_6_): δ 10.34 (br d, *J* = 5.3 Hz, 1H), 8.48–8.41 (m, 1H), 7.97 (d, *J* = 1.9 Hz, 1H), 7.80 (ddd, *J* = 9.2, 4.7, 2.9 Hz, 3H), 7.65 (dd, *J* = 9.0, 2.7 Hz, 2H), 7.56 (dd, *J* = 8.2, 3.6 Hz, 1H), 3.35 (td, *J* = 6.6, 2.1 Hz, 2H), 2.87–2.77 (m, 2H), 2.39 (d, *J* = 3.7 Hz, 3H) ppm. ^13^C­{^1^H} NMR (75 MHz, DMSO-*d*_6_): δ 170.3, 170.0, 159.7, 155.4, 142.5, 141.6, 139.9, 133.4, 131.6, 128.1, 126.5, 126.1 (q, *J* = 5.8, 4.1 Hz, 2C), 123.2 (d, *J* = 32.0 Hz), 121.1 (d, *J* = 220.5 Hz, 2C), 118.9 (2C), 35.9, 25.7, 19.7 ppm. ^19^F NMR (282 MHz, DMSO-*d*_6_): δ −60.33 (d, *J* = 9.1 Hz) ppm. HR-MS (ESI) calc. for [M + H]^+^: 532.0374, found: 532.0384.

#### 2-{[5-(3-Chloro-4-methylbenzenesulfonyl)-4-oxo-1,4-dihydropyrimidin-2-yl]­sulfanyl}-*N*-[4-(trifluoromethyl)­phenyl]­propenamide (Compound **23**)

Compound **23** was synthesized from **X7** and **X8w** following the procedure described for compound **1** as a yellow solid (80% yield). ^1^H NMR (300 MHz, DMSO-*d*_6_): δ 11.23 (br s, 1H), 8.31 (d, *J* = 2.1 Hz, 1H), 7.98 (d, *J* = 2.1 Hz, 1H), 7.80–7.77 (m, 1H), 7.77–7.72 (m, 2H), 7.61 (d, *J* = 8.9 Hz, 2H), 7.52 (d, *J* = 8.1 Hz, 1H), 4.49 (q, *J* = 7.1 Hz, 1H), 2.38 (s, 4H), 1.46 (d, *J* = 7.2 Hz, 3H) ppm. ^13^C­{^1^H} NMR (75 MHz, DMSO-*d*_6_): δ 172.3, 170.7, 163.0, 155.9, 142.5, 141.0, 140.9, 133.2, 131.6 (d, *J* = 281.6 Hz), 131.5, 128.0, 126.3, 126.2–126.0 (m, 2C), 123.4 (q, *J* = 32.0 Hz), 119.0 (2C), 118.4, 43.0, 19.7, 17.2 ppm. ^19^F NMR (282 MHz, DMSO-*d*_6_): δ −60.37 ppm. HR-MS (ESI) calc. for [M + H]^+^: 532.0374, found: 532.0388.

#### 2-{[5-(3-Chloro-4-methylbenzenesulfonyl)-4-oxo-1,4-dihydropyrimidin-2-yl]­sulfanyl}-*N*-[4-(trifluoromethyl)­phenyl]­butanamide (Compound **24**)

Compound **24** was synthesized from **X7** and **X8x** following the procedure described for compound **1** as a white solid (42% yield). ^1^H NMR (400 MHz, DMSO-*d*_6_): δ 10.78 (br s, 1H), 8.51 (s, 1H), 7.96 (d, *J* = 1.9 Hz, 1H), 7.86–7.77 (m, 3H), 7.68 (d, *J* = 8.6 Hz, 2H), 7.58 (d, *J* = 8.1 Hz, 1H), 4.72 (t, *J* = 6.9 Hz, 1H), 2.39 (s, 3H), 2.09–1.86 (m, 2H), 0.98 (t, *J* = 7.3 Hz, 3H) ppm. ^13^C­{^1^H} NMR (101 MHz, DMSO-*d*_6_): δ 168.7, 167.9, 157.4, 155.2, 142.1, 142.0, 139.1, 133.6, 131.8, 128.1, 126.7, 126.1 (2C), 128.8–120.4 (m), 123.8 (q, *J* = 32.9, 32.4 Hz), 121.1, 119.4 (2C), 51.3, 25.8, 19.8, 11.1 ppm. ^19^F NMR (377 MHz, DMSO-*d*_6_): δ −60.44 ppm. HR-MS (ESI) calc. for [M + H]^+^: 546.0531, found: 546.0543.

#### 2-{[5-(3-Chloro-4-methylbenzenesulfonyl)-4-oxo-1,4-dihydropyrimidin-2-yl]­sulfanyl}-3-methyl-*N*-[4-(trifluoromethyl)­phenyl]­butanamide (Compound **25**)

Compound **25** was synthesized from **X7** and **X8y** following the procedure described for compound **1** as a yellow solid (49% yield). ^1^H NMR (400 MHz, DMSO-*d*_6_): δ 10.90 (br s, 2H), 8.34 (d, *J* = 14.6 Hz, 0H), 7.98 (d, *J* = 1.9 Hz, 2H), 7.79 (dd, *J* = 8.0, 1.7 Hz, 7H), 7.64 (d, *J* = 8.6 Hz, 5H), 7.53 (d, *J* = 8.1 Hz, 3H), 4.45 (d, *J* = 7.3 Hz, 2H), 2.38 (s, 7H), 2.23 (tt, *J* = 14.1, 7.0 Hz, 2H), 1.03 (d, *J* = 6.7 Hz, 6H) ppm. ^13^C­{^1^H} NMR (101 MHz, DMSO-*d*_6_): δ 171.0, 169.6, 161.2, 155.4, 142.3, 141.3, 140.3, 133.3, 131.5, 128.0, 126.4, 126.1 (q, *J* = 3.7 Hz, 2C), 124.3 (d, *J* = 271.4 Hz), 123.5 (d, *J* = 31.8 Hz), 119.2 (3C), 55.4, 30.3, 20.2, 19.7 (d, *J* = 8.1 Hz, 2C) ppm. ^19^F NMR (377 MHz, DMSO-*d*_6_): δ −60.38 ppm. HR-MS (ESI) calc. for [M + H]^+^: 560.0687, found: 560.0705.

#### 2-{[5-(3-Chloro-4-methylbenzenesulfonyl)-4-oxo-1,4-dihydropyrimidin-2-yl]­sulfanyl}-2-phenyl-*N*-[4-(trifluoromethyl)­phenyl]­acetamide (Compound **26**)

Compound **26** was synthesized from **X7** and **X8z** following the procedure described for compound **1** as a yellow solid (74% yield). ^1^H NMR (300 MHz, DMSO-*d*_6_): δ 11.02 (br s, 1H), 8.35 (s, 1H), 7.96 (d, *J* = 2.0 Hz, 1H), 7.82–7.73 (m, 3H), 7.64 (d, *J* = 8.9 Hz, 2H), 7.59–7.48 (m, 3H), 7.43–7.25 (m, 3H), 5.79 (s, 1H), 2.37 (s, 3H) ppm. ^13^C­{^1^H} NMR (75 MHz, DMSO-*d*_6_): δ 170.7, 168.5, 161.6, 155.8, 142.8, 141.7, 140.7, 136.4, 133.7, 131.9, 129.2 (2C), 128.9 (2C), 128.7, 128.5, 126.7 (d, *J* = 19.3 Hz, 2C), 126.5, 124.1 (q, *J* = 31.8 Hz), 122.9 (d, *J* = 269.3 Hz), 119.7 (2C), 53.7, 20.2 ppm. ^19^F NMR (282 MHz, DMSO-*d*_6_): δ −60.43 ppm. HR-MS (ESI) calc. for [M + H]^+^: 594.0531, found: 594.0547.

#### Sodium 4-Methylbenzene-1-sulfinate (**X10a**)

Based on Wang et al.,^37^ 4-methylbenzene-1-sulfonyl chloride (**X9a**, 2.86 g, 15.0 mmol, 1.0 equiv) was dissolved in 70 mL of H_2_O. NaHCO_3_ (2.52 g, 30.0 mmol, 2.0 equiv) and Na_2_SO_3_ (3.78 g, 30.0 mmol, 2.0 equiv) were added, and the solution was stirred at 80 °C for 16 h. The solution was concentrated in vacuo, and the crude product was suspended in 150 mL of EtOH. The filtrate was concentrated in vacuo to obtain **X10a** as a white solid (2.64 g, 99% yield).

#### Sodium 3-Chlorobenzene-1-sulfinate (**X10b**)

**X10b** was synthesized from 3-chlorobenzene-1-sulfonyl chloride (**X9b**) following the procedure described for **X10a** as a white solid (70% yield).

#### Sodium 4-Fluorobenzene-1-sulfinate (**X10c**)

**X10c** was synthesized from 4-fluorobenzene-1-sulfonyl chloride (**X9c**) following the procedure described for **X10a** as a white solid (99% yield).

#### Sodium 4-Chlorobenzene-1-sulfinate (**X10d**)

**X10d** was synthesized from 4-chlorobenzene-1-sulfonyl chloride (**X9d**) following the procedure described for **X10a** as a white solid (97% yield).

#### Sodium 4-Bromobenzene-1-sulfinate (**X10e**)

**X10e** was synthesized from 4-bromobenzene-1-sulfonyl chloride (**X9e**) following the procedure described for **X10a** as a white solid (58% yield).

#### Sodium 4-Iodobenzene-1-sulfinate (**X10f**)

**X10f** was synthesized from 4-iodobenzene-1-sulfonyl chloride (**X9f**) following the procedure described for **X10a** as a white solid (76% yield).

#### Sodium 4-(Trifluoromethyl)­benzene-1-sulfinate (**X10g**)

**X10g** was synthesized from 4-(trifluoromethyl)­benzene-1-sulfonyl chloride (**X9g**) following the procedure described for **X10a** as a white solid (94% yield).

#### Sodium 4-Nitrobenzene-1-sulfinate (**X10h**)

**X10h** was synthesized from 4-nitrobenzene-1-sulfonyl chloride (**X9h**) following the procedure described for **X10a** as a white solid (58% yield).

#### Sodium 4-Methoxybenzene-1-sulfinate (**X10i**)

**X10i** was synthesized from 4-methoxybenzene-1-sulfonyl chloride (**X9i**) following the procedure described for **X10a** as a white solid (63% yield).

#### Sodium Benzenesulfinate (**X10j**)

**X10j** was synthesized from sodium benzenesulfonyl chloride (**X9j**) following the procedure described for **X10a** as a white solid (82% yield).

#### Sodium 2-Chlorobenzene-1-sulfinate (**X10k**)

**X10k** was synthesized from 2-chlorobenzene-1-sulfonyl chloride (**X9k**) following the procedure described for **X10a** as a white solid (58% yield).

#### Sodium 3-(Trifluoromethyl)­benzene-1-sulfinate (**X10l**)

**X10l** was synthesized from 3-(trifluoromethyl)­benzene-1-sulfonyl chloride (**X9l**) following the procedure described for **X10a** as a white solid (99% yield).

#### Sodium [1,1′-Biphenyl]-4-sulfinate (**X10m**)

**X10m** was synthesized from [1,1′-biphenyl]-4-sulfonyl chloride (**X9m**) following the procedure described for **X10a** as a white solid (76% yield).

#### Sodium 4-Benzylbenzene-1-sulfinate (**X10n**)

**X10n** was synthesized from 4-benzylbenzene-1-sulfonyl chloride (**X9n**) following the procedure described for **X10a** as a white solid (95% yield).

#### Sodium Pyridine-2-sulfinate (**X10o**)

Based on Wei et al., pyridine-2-thiol (1.0 g, 8.94 mmol, 1.0 equiv) was dissolved in 100 mL of EtOH.^36^ Aq. NaOH (6 mL, 11.6 mmol, 2 M, 1.3 equiv) and aq. H_2_O_2_ (1.0 mL, 9.83 mmol, 30%, 1.1 equiv) were added, and the solution was stirred at RT for 21 h. The solution was concentrated in vacuo, and the crude product was dissolved in H_2_O and extracted with dichloromethane. The aqueous phase was concentrated in vacuo to obtain **X10o** as a white solid (1.69 g, 95% yield).

#### Sodium Naphthalene-2-sulfinate (**X10p**)

**X10p** was synthesized from naphthalene-2-sulfonyl chloride (**X9p**) following the procedure described for **X10a** as a white solid (56% yield).

#### Sodium Quinoline-8-sulfinate (**X10q**)

**X10q** was synthesized from quinoline-8-sulfonyl chloride (**X9q**) following the procedure described for **X10a** as a white solid (95% yield).

#### Ethyl 2-(4-Methylbenzenesulfonyl)­acetate (**X11a**)

Based on ref 35, **X10a** (2.37 g, 10.0 mmol, 1.0 equiv), Na_2_CO_3_ (794 mg, 7.5 mmol, 0.75 equiv), and ethyl bromoacetate (1.31 mL, 20.0 mmol, 2.0 equiv) were dissolved in 50 mL of a 1:1 mixture of ACN/THF. The solution was stirred at 35 °C for 16 h. The solution was concentrated in vacuo. The crude product was purified by column chromatography (cyclohexane/ethyl acetate 9:1) to obtain **X11a** as a yellow liquid (36% yield). ^1^H NMR (400 MHz, CDCl_3_): δ 7.85–7.78 (m, 2H), 7.39–7.33 (m, 2H), 4.14 (q, *J* = 7.1 Hz, 2H), 4.08 (s, 2H), 2.45 (s, 3H), 1.19 (t, *J* = 7.1 Hz, 3H) ppm. ^13^C­{^1^H} NMR (101 MHz, CDCl_3_): δ 162.6, 145.5, 135.9, 129.9 (C2), 128.7 (C2), 62.4, 61.2, 21.8, 14.0 ppm. LR-MS (EI) calc. for (M^+•^): 242.1, found: 242.1.

#### Ethyl 2-(3-Chlorobenzenesulfonyl)­acetate (**X11b**)

**X11b** was synthesized from **X10b** following the procedure described for **X11a** as a yellow liquid (41% yield). ^1^H NMR (400 MHz, CDCl_3_): δ 7.94 (t, *J* = 1.9 Hz, 1H), 7.87–7.82 (m, 1H), 7.68–7.63 (m, 1H), 7.53 (t, *J* = 7.9 Hz, 1H), 4.16 (q, *J* = 7.1 Hz, 2H), 4.12 (s, 2H), 1.21 (t, *J* = 7.1 Hz, 3H). ^13^C­{^1^H} NMR (101 MHz, CDCl_3_): δ 162.1, 151.3, 144.3, 130.4 (2C), 124.5 (2C), 62.9, 60.7, 14.0 ppm. LR-MS (EI) calc. for (M^+•^): 262.0, found: 262.0.

#### Ethyl 2-(4-Fluorobenzenesulfonyl)­acetate (**X11c**)

**X11c** was synthesized from **X10c** following the procedure described for **X11a** as a yellow liquid (43% yield). ^1^H NMR (300 MHz, CDCl_3_): δ 8.08–7.90 (m, 2H), 7.31–7.19 (m, 2H), 4.15 (q, *J* = 7.2 Hz, 2H), 4.11 (s, 2H), 1.21 (t, *J* = 7.1 Hz, 3H) ppm. ^13^C­{^1^H} NMR (75 MHz, CDCl_3_): δ 166.2 (d, *J* = 257.3 Hz), 162.4, 134.9 (d, *J* = 3.4 Hz), 131.8 (d, *J* = 9.8 Hz, 2C), 116.7 (d, *J* = 22.8 Hz, 2C), 62.6, 61.1, 14.0 ppm. ^19^F NMR (282 MHz, CDCl_3_): δ −102.37 (td, *J* = 1.84, 4.3 Hz) ppm. LR-MS (EI) calc. for (M^+•^): 246.0, found: 246.1.

#### Ethyl 2-(4-Chlorobenzenesulfonyl)­acetate (**X11d**)

**X11d** was synthesized from **X10d** following the procedure described for **X11a** as a yellow liquid (51% yield). ^1^H NMR (400 MHz, CDCl_3_): δ 7.89 (d, *J* = 8.6 Hz, 2H), 7.55 (d, *J* = 8.6 Hz, 2H), 4.15 (q, *J* = 7.1 Hz, 2H), 4.11 (s, 2H), 1.21 (t, *J* = 7.1 Hz, 3H) ppm. ^13^C­{^1^H} NMR (101 MHz, CDCl_3_): δ 162.4, 141.3, 137.3, 130.3 (2C), 129.7 (2C), 62.7, 61.1, 14.0 ppm. LR-MS (EI) calc. for (M^+•^): 262.0, found: 262.0.

#### Ethyl 2-(4-Bromobenzenesulfonyl)­acetate (**X11e**)

**X11e** was synthesized from **X10e** following the procedure described for **X11a** as a yellow liquid (53% yield). ^1^H NMR (400 MHz, CDCl_3_): δ 7.82 (d, *J* = 8.7 Hz, 2H), 7.75–7.71 (m, 2H), 4.17 (q, *J* = 7.1 Hz, 2H), 4.10 (s, 2H), 1.23 (t, *J* = 7.1 Hz, 3H) ppm. ^13^C­{^1^H} NMR (101 MHz, CDCl_3_): δ 162.2, 137.7, 132.5 (2C), 130.2 (2C), 129.8, 62.5, 60.9, 13.9 ppm. LR-MS (EI) calc. for (M^+•^): 308.0, found: 308.0.

#### Ethyl 2-(4-Iodobenzenesulfonyl)­acetate (**X11f**)

**X11f** was synthesized from **X10f** following the procedure described for **X11a** as a yellow liquid (54% yield). ^1^H NMR (400 MHz, CDCl_3_): δ 7.97–7.93 (m, 2H), 7.67–7.63 (m, 2H), 4.17 (q, *J* = 7.1 Hz, 2H), 4.10 (s, 2H), 1.23 (t, *J* = 7.1 Hz, 3H) ppm. ^13^C­{^1^H} NMR (101 MHz, CDCl_3_): δ 162.4, 138.7 (2C), 130.1 (2C), 102.7, 62.7, 61.0, 14.0 ppm. LR-MS (EI) calc. for (M^+•^): 353.9, found: 354.0.

#### Ethyl 2-[4-(Trifluoromethyl)­benzenesulfonyl]­acetate (**X11g**)

**X11g** was synthesized from **X10g** following the procedure described for **X11a** as a yellow liquid (52% yield). ^1^H NMR (400 MHz, CDCl_3_): δ 8.14–8.07 (m, 2H), 7.93–7.81 (m, 2H), 4.16 (d, *J* = 7.3 Hz, 4H), 1.21 (t, *J* = 7.2 Hz, 3H) ppm. ^13^C­{^1^H} NMR (101 MHz, CDCl_3_): δ 162.2, 142.3, 136.1 (q, *J* = 33.2 Hz), 129.5, 126.5 (q, *J* = 3.8 Hz), 123.2 (q, *J* = 273.2 Hz), 62.8, 60.9, 14.0 ppm. ^19^F NMR (376 MHz, CDCl_3_): δ −63.32 (s) ppm. LR-MS (EI) calc. for (M^+•^): 297.0, found: 297.0.

#### Ethyl 2-(4-Nitrobenzenesulfonyl)­acetate (**X11h**)

**X11h** was synthesized from **X10h** following the procedure described for **X11a** as a yellow liquid (28% yield). ^1^H NMR (400 MHz, CDCl_3_): δ 8.45–8.40 (m, 2H), 8.19–8.14 (m, 2H), 4.17 (q, *J* = 6.7 Hz, 4H), 1.23 (t, *J* = 7.2 Hz, 3H) ppm. ^13^C­{^1^H} NMR (101 MHz, CDCl_3_): δ 162.2, 136.4, 135.3, 132.8, 132.2, 132.0, 127.5, 62.5, 58.9, 13.9 ppm. LR-MS (EI) calc. for (M^+•^): 273.0, found: 273.1.

#### Ethyl 2-(4-Methoxybenzenesulfonyl)­acetate (**X11i**)

**X11i** was synthesized from **X10i** following the procedure described for **X11a** as a yellow liquid (14% yield). ^1^H NMR (400 MHz, CDCl_3_): δ 7.88–7.80 (m, 2H), 7.21–7.13 (m, 2H), 4.52 (s, 2H), 4.03 (q, *J* = 7.1 Hz, 2H), 3.86 (s, 3H), 1.07 (t, *J* = 7.1 Hz, 3H) ppm. ^13^C­{^1^H} NMR (101 MHz, CDCl_3_): δ 163.6, 162.8, 130.5 (3C), 114.4 (2C), 61.4, 60.2, 55.8, 13.7 ppm. LR-MS (EI) calc. for (M^+•^): 258.1, found: 258.1.

#### Ethyl 2-(Benzenesulfonyl)­acetate (**X11j**)

**X11j** was synthesized from **X10j** following the procedure described for **X11a** as a yellow liquid (95% yield). ^1^H NMR (400 MHz, DMSO-*d*_6_): δ 7.97–7.89 (m, 2H), 7.82–7.73 (m, 1H), 7.72–7.62 (m, 2H), 4.62 (s, 2H), 4.02 (q, *J* = 7.11 Hz, 2H), 1.04 (t, *J* = 7.10 Hz, 3H) ppm. ^13^C­{^1^H} NMR (101 MHz, DMSO-*d*_6_): δ 162.6, 139.0, 134.2, 129.3 (2C), 128.1 (2C), 61.5, 59.8, 13.6 ppm. HR-MS (ESI) calc. for [M + H]^+^: 229.0530, found: 229.0534.

#### Ethyl 2-(2-Chlorobenzenesulfonyl)­acetate (**X11k**)

**X11k** was synthesized from **X10k** following the procedure described for **X11a** as a yellow liquid (48% yield). ^1^H NMR (400 MHz, CDCl_3_): δ 8.16–8.10 (m, 1H), 7.65–7.54 (m, 2H), 7.53–7.44 (m, 1H), 4.44 (s, 2H), 4.10 (q, *J* = 7.2 Hz, 2H), 1.13 (td, *J* = 7.1, 0.7 Hz, 3H) ppm. ^13^C­{^1^H} NMR (101 MHz, CDCl_3_): δ 162.2, 136.4, 135.3, 132.8, 132.2, 132.0, 127.5, 62.5, 58.9, 13.9 ppm. LR-MS (EI) calc. for (M^+•^): 262.0, found: 262.0.

#### Ethyl 2-[3-(Trifluoromethyl)­benzenesulfonyl]­acetate (**X11l**)

**X11l** was synthesized from **X10l** following the procedure described for **X11a** as a yellow liquid (64% yield). ^1^H NMR (400 MHz, CDCl_3_): δ 8.22 (s, 1H), 8.16 (d, *J* = 8.0 Hz, 1H), 7.95 (d, *J* = 8.0 Hz, 1H), 7.75 (t, *J* = 8.2 Hz, 1H), 4.20–4.10 (m, 4H), 1.20 (t, *J* = 7.1 Hz, 3H) ppm. ^13^C­{^1^H} NMR (101 MHz, CDCl_3_): δ 162.0, 139.9, 132.0, 132.0 (q, *J* = 33.8 Hz), 130.9 (q, *J* = 3.6 Hz), 130.0, 125.9 (q, *J* = 3.9 Hz), 124.4 (d, *J* = 272.7 Hz), 62.6, 60.8, 13.8 ppm. ^19^F NMR (376 MHz, CDCl_3_): δ −62.73 (d, *J* = 4.8 Hz) ppm. LR-MS (EI) calc. for (M^+•^): 297.0, found: 297.1.

#### Ethyl 2-{[1,1′-Biphenyl]-4-sulfonyl}­acetate (**X11m**)

**X11m** was synthesized from **X10m** following the procedure described for **X11a** as a yellow liquid (35% yield). ^1^H NMR (300 MHz, CDCl_3_): δ 8.08–7.96 (m, 2H), 7.84–7.73 (m, 2H), 7.67–7.58 (m, 2H), 7.53–7.40 (m, 3H), 4.17 (q, *J* = 7.1 Hz, 2H), 4.15 (s, 2H), 1.21 (t, *J* = 7.1 Hz, 3H) ppm. ^13^C­{^1^H} NMR (75 MHz, CDCl_3_): δ 162.6, 147.4, 139.1, 137.4, 129.3 (4C), 128.9, 127.9 (2C), 127.6 (2C), 62.6, 61.3, 14.0 ppm. LR-MS (EI) calc. for (M^+•^): 304.1, found: 304.1.

#### Ethyl 2-(4-Benzylbenzenesulfonyl)­acetate (**X11n**)

**X11n** was synthesized from **X10n** following the procedure described for **X11a** as a yellow liquid (45% yield). ^1^H NMR (400 MHz, CDCl_3_): δ 7.85 (d, *J* = 8.3 Hz, 2H), 7.39 (d, *J* = 8.6 Hz, 2H), 7.31 (t, *J* = 7.5 Hz, 2H), 7.25 (d, *J* = 5.9 Hz, 1H), 7.17 (d, *J* = 7.4 Hz, 1H), 4.13 (q, *J* = 7.1 Hz, 3H), 4.09 (s, 2H), 4.07 (s, 2H), 1.17 (t, *J* = 7.1 Hz, 3H) ppm. ^13^C­{^1^H} NMR (101 MHz, CDCl_3_): δ 162.5, 148.5, 139.4, 136.7, 129.8 (2C), 129.1 (2C), 128.9 (2C), 128.9 (2C), 126.8, 62.5, 61.2, 42.0, 14.0 ppm. LR-MS (ESI) calc. for [M + Na]^+^: 341.1, found: 341.2.

#### Ethyl 2-(Pyridine-2-sulfonyl)­acetate (**X11o**)

**X11o** was synthesized from **X10o** following the procedure described for **X11a** as a yellow liquid (16% yield). ^1^H NMR (300 MHz, DMSO-*d*_6_): δ 8.82–8.79 (m, 1H), 8.18 (td, *J* = 7.7, 1.7 Hz, 1H), 8.06 (dt, *J* = 7.9, 1.1 Hz, 1H), 7.81–7.75 (m, 1H), 4.72 (s, 2H), 4.00 (q, *J* = 7.1 Hz, 2H), 1.01 (t, *J* = 7.1 Hz, 3H) ppm. ^13^C­{^1^H} NMR (75 MHz, DMSO-*d*_6_): δ 162.5, 156.2, 150.3, 139.1, 128.3, 122.0, 61.5, 56.1, 13.6 ppm. LR-MS (EI) calc. for (M^+•^): 229.0, found: 229.1.

#### Ethyl 2-(Naphthalene-2-sulfonyl)­acetate (**X11p**)

**X11p** was synthesized from **X10p** following the procedure described for **X11a** as a yellow liquid (67% yield). ^1^H NMR (400 MHz, CDCl_3_): δ 8.56–8.51 (m, 1H), 8.05–7.98 (m, 3H), 7.98–7.88 (m, 3H), 7.74–7.60 (m, 2H), 4.19 (s, 2H), 4.13 (q, *J* = 7.2 Hz, 2H), 1.14 (t, *J* = 7.2 Hz, 3H) ppm. ^13^C­{^1^H} NMR (101 MHz, CDCl_3_): δ 162.5, 135.8, 135.7, 132.2, 130.8, 129.8–129.6 (m, 3C), 128.2, 128.0, 123.1, 62.5, 61.3, 14.0 ppm. LR-MS (EI) calc. for (M^+•^): 278.1, found: 278.1.

#### Ethyl 2-(Quinoline-8-sulfonyl)­acetate (**X11q**)

**X11q** was synthesized from **X10q** following the procedure described for **X11a** as a yellow liquid (41% yield). ^1^H NMR (400 MHz, DMSO-*d*_6_): δ 9.12 (dd, *J* = 4.3, 1.8 Hz, 1H), 8.62 (dd, *J* = 8.3, 1.8 Hz, 1H), 8.41 (ddd, *J* = 11.8, 7.8, 1.5 Hz, 2H), 7.84 (t, *J* = 7.8 Hz, 1H), 7.76 (dd, *J* = 8.4, 4.2 Hz, 1H), 5.09 (s, 2H), 3.90 (q, *J* = 7.1 Hz, 2H), 0.85 (t, *J* = 7.1 Hz, 3H) ppm. ^13^C­{^1^H} NMR (101 MHz, DMSO-*d*_6_): δ 162.7, 151.7, 143.0, 137.3, 135.3, 135.2, 131.4, 128.6, 125.8, 122.7, 61.2, 59.9, 13.4 ppm. LR-MS (EI) calc. for (M^+•^): 279.1, found: 279.2.

#### Ethyl (2*E*)-3-Ethoxy-2-(4-methylbenzenesulfonyl)­prop-2-enoate (**X12a**)

**X12a** was synthesized from **X11a** following the procedure described for **X6** as a yellow solid (42% yield). ^1^H NMR (400 MHz, DMSO-*d*_6_): δ 8.18 (s, 1H), 7.79–7.76 (m, 2H), 7.42–7.37 (m, 2H), 4.45 (q, *J* = 7.1 Hz, 2H), 4.00 (q, *J* = 7.2 Hz, 1H), 2.39 (s, 3H), 1.31 (t, *J* = 7.1 Hz, 3H), 1.06 (t, *J* = 7.1 Hz, 3H) ppm. ^13^C­{^1^H} NMR (101 MHz, DMSO-*d*_6_): δ 169.2, 160.2, 143.4, 138.8, 129.3 (2C), 127.7 (2C), 112.2, 74.0, 60.4, 21.0, 15.1, 13.8 ppm. LR-MS (EI) calc. for (M^+•^): 298.1, found: 298.1.

#### Ethyl (2*E*)-2-(3-Chlorobenzenesulfonyl)-3-ethoxyprop-2-enoate (**X12b**)

**X12b** was synthesized from **X11b** following the procedure described for **X6** as a yellow solid (83% yield). ^1^H NMR (400 MHz, DMSO-*d*_6_): δ 8.23 (s, 1H), 7.95 (t, *J* = 1.9 Hz, 1H), 7.90–7.87 (m, 1H), 7.78–7.74 (m, 1H), 7.64 (t, *J* = 7.9 Hz, 1H), 4.49 (q, *J* = 7.1 Hz, 2H), 4.02 (q, *J* = 7.1 Hz, 2H), 1.32 (t, *J* = 7.1 Hz, 3H), 1.06 (t, *J* = 7.1 Hz, 3H) ppm. ^13^C­{^1^H} NMR (101 MHz, DMSO-*d*_6_): δ 170.6, 159.9, 143.6, 133.4, 133.0, 130.9, 127.3, 126.4, 110.9, 74.4, 60.5, 15.0, 13.7 ppm. LR-MS (EI) calc. for (M^+•^): 318.0, found: 318.0.

#### Ethyl (2*E*)-3-Ethoxy-2-(4-fluorobenzenesulfonyl)­prop-2-enoate (**X12c**)

**X12c** was synthesized from **X11c** following the procedure described for **X6** as a yellow solid (72% yield). ^1^H NMR (400 MHz, DMSO-*d*_6_): δ 8.22 (s, 1H), 8.01–7.95 (m, 2H), 7.48–7.41 (m, 2H), 4.47 (q, *J* = 7.1 Hz, 2H), 4.02 (q, *J* = 7.1 Hz, 2H), 1.32 (t, *J* = 7.1 Hz, 3H), 1.06 (t, *J* = 7.1 Hz, 3H) ppm. ^13^C­{^1^H} NMR (101 MHz, DMSO-*d*_6_): δ 170.3, 165.0 (d, *J* = 251.8 Hz), 160.5, 138.5 (d, *J* = 2.9 Hz), 131.3 (2C), 116.5 (d, *J* = 22.9 Hz, 2C), 112.1, 74.6, 60.9, 15.5, 14.2 ppm. ^19^F NMR (376 MHz, DMSO-*d*_6_): δ −106.05 (tt, *J* = 8.7, 5.1 Hz) ppm. LR-MS (EI) calc. (M^+•^): 302.1, found: 302.1.

#### Ethyl (2*E*)-2-(4-Chlorobenzenesulfonyl)-3-ethoxyprop-2-enoate (**X12d**)

**X12d** was synthesized from **X11d** following the procedure described for **X6** as a yellow solid (61% yield). ^1^H NMR (400 MHz, DMSO-*d*_6_): δ 8.22 (s, 1H), 7.97–7.86 (m, 2H), 7.72–7.64 (m, 2H), 4.47 (q, *J* = 7.1 Hz, 2H), 4.02 (q, *J* = 7.1 Hz, 2H), 1.32 (t, *J* = 7.1 Hz, 3H), 1.06 (t, *J* = 7.1 Hz, 3H) ppm. ^13^C­{^1^H} NMR (101 MHz, DMSO-*d*_6_): δ 170.6, 160.5, 141.0, 138.4, 130.1 (2C), 129.5 (2C), 111.8, 74.8, 61.0, 15.5, 14.2 ppm. LR-MS (EI) calc. for (M^+•^): 318.0, found: 318.0.

#### Ethyl (2*E*)-2-(4-Bromobenzenesulfonyl)-3-ethoxyprop-2-enoate (**X12e**)

**X12e** was synthesized from **X11e** following the procedure described for **X6** as a yellow solid (31% yield). ^1^H NMR (400 MHz, DMSO-*d*_6_): δ 8.22 (s, 1H), 7.93–7.77 (m, 4H), 4.47 (q, *J* = 7.1 Hz, 2H), 4.02 (q, *J* = 7.1 Hz, 2H), 1.32 (t, *J* = 7.1 Hz, 3H), 1.06 (t, *J* = 7.1 Hz, 3H) ppm. ^13^C­{^1^H} NMR (101 MHz, DMSO-*d*_6_): δ 170.1, 160.0, 141.0, 131.9 (2C), 129.7 (2C), 127.0, 111.3, 74.3, 60.5, 15.0, 13.7 ppm. LR-MS (EI) calc. for (M^+•^): 364.0, found: 364.0.

#### Ethyl (2*E*)-3-Ethoxy-2-(4-iodobenzenesulfonyl)­prop-2-enoate (**X12f**)

**X12f** was synthesized from **X11f** following the procedure described for **X6** as a yellow solid (65% yield). ^1^H NMR (400 MHz, DMSO-*d*_6_): δ 8.21 (s, 1H), 8.04–7.95 (m, 2H), 7.70–7.62 (m, 2H), 4.47 (q, *J* = 7.1 Hz, 2H), 4.02 (q, *J* = 7.1 Hz, 2H), 1.31 (t, *J* = 7.1 Hz, 3H), 1.06 (t, *J* = 7.1 Hz, 3H) ppm. ^13^C­{^1^H} NMR (101 MHz, DMSO-*d*_6_): δ 170.0, 160.0, 141.3, 137.8 (2C), 129.3 (2C), 111.3, 101.4, 74.2, 60.5, 15.1, 13.7 ppm. LR-MS (EI) calc. for (M^+•^): 410.0, found: 410.0.

#### Ethyl (2*E*)-3-Ethoxy-2-[4-(trifluoromethyl)­benzenesulfonyl]­prop-2-enoate (**X12g**)

**X12g** was synthesized from **X11g** following the procedure described for **X6** as a yellow solid (62% yield). ^1^H NMR (400 MHz, DMSO-*d*_6_): δ 8.28 (s, 1H), 8.20–8.11 (m, 2H), 8.03–7.96 (m, 2H), 4.50 (q, *J* = 7.1 Hz, 2H), 4.02 (q, *J* = 7.1 Hz, 2H), 1.33 (t, *J* = 7.1 Hz, 3H), 1.04 (t, *J* = 7.1 Hz, 3H) ppm. ^13^C­{^1^H} NMR (101 MHz, DMSO-*d*_6_): δ 170.7, 159.9, 145.6 (d, *J* = 1.5 Hz), 132.6 (q, *J* = 32.1 Hz), 128.6 (2C), 126.1 (q, *J* = 3.7 Hz), 123.5 (q, *J* = 273.0 Hz, 2C), 110.7, 74.5, 60.5, 15.0, 13.7 ppm. ^19^F NMR (376 MHz, DMSO-*d*_6_): δ −61.63 (s) ppm. LR-MS (EI) calc. for (M^+•^): 352.1, found: 352.1.

#### Ethyl (2*E*)-3-Ethoxy-2-(4-nitrobenzenesulfonyl)­prop-2-enoate (**X12h**)

**X12h** was synthesized from **X11h** following the procedure described for **X6** as a yellow solid (68% yield). ^1^H NMR (400 MHz, DMSO-*d*_6_): δ 8.43–8.40 (m, 2H), 8.30 (s, 1H), 8.20–8.16 (m, 2H), 4.52 (q, *J* = 7.1 Hz, 2H), 4.02 (q, *J* = 7.1 Hz, 2H), 1.33 (t, *J* = 7.1 Hz, 3H), 1.06 (t, *J* = 7.1 Hz, 3H) ppm. ^13^C­{^1^H} NMR (101 MHz, DMSO-*d*_6_): δ 171.1, 159.9, 149.9, 147.1, 129.2 (2C), 124.4 (2C), 110.4, 74.6, 60.6, 15.0, 13.7 ppm. LR-MS (EI) calc. for (M^+•^): 329.1, found: 329.0.

#### Ethyl (2*E*)-3-Ethoxy-2-(4-methoxybenzenesulfonyl)­prop-2-enoate (**X12i**)

**X12i** was synthesized from **X11i** following the procedure described for **X6** as a yellow solid (24% yield). ^1^H NMR (400 MHz, DMSO-*d*_6_): δ 8.16 (s, 1H), 7.87–7.77 (m, 2H), 7.14–7.06 (m, 2H), 4.43 (q, *J* = 7.1 Hz, 2H), 4.02 (q, *J* = 7.1 Hz, 2H), 3.84 (s, 3H), 1.30 (t, *J* = 7.1 Hz, 3H), 1.08 (t, *J* = 7.1 Hz, 3H) ppm. ^13^C­{^1^H} NMR (101 MHz, DMSO-*d*_6_): δ 168.7, 162.6, 160.2, 133.2, 130.0 (2C), 114.0 (2C), 112.6, 73.8, 60.3, 55.7, 15.1, 13.8 ppm. LR-MS (EI) calc. for (M^+•^): 314.1, found: 314.1.

#### Ethyl (2*E*)-2-(Benzenesulfonyl)-3-ethoxyprop-2-enoate (**X12j**)

**X12j** was synthesized from **X11j** following the procedure described for **X6** as a yellow solid (79% yield). ^1^H NMR (400 MHz, DMSO-*d*_6_): δ 8.21 (s, 1H), 7.97–7.85 (m, 2H), 7.70–7.64 (m, 1H), 7.63–7.56 (m, 2H), 4.47 (q, *J* = 7.1 Hz, 2H), 4.00 (q, *J* = 7.1 Hz, 2H), 1.32 (t, *J* = 7.1 Hz, 3H), 1.04 (t, *J* = 7.1 Hz, 3H) ppm. ^13^C­{^1^H} NMR (101 MHz, DMSO-*d*_6_): δ 169.5, 160.1, 141.6, 133.0, 128.8, 127.5, 111.8, 74.1, 60.3, 15.1, 13.7 ppm. HR-MS (ESI) calc. for [M + H]^+^: 285.0792, found: 285.0799.

#### Ethyl (2*E*)-2-(2-Chlorobenzenesulfonyl)-3-ethoxyprop-2-enoate (**X12k**)

**X12k** was synthesized from **X11k** following the procedure described for **X6** as a yellow solid (68% yield). ^1^H NMR (400 MHz, DMSO-*d*_6_): δ 8.26 (s, 1H), 8.12 (dd, *J* = 7.9, 1.7 Hz, 1H), 7.72–7.58 (m, 3H), 4.53 (q, *J* = 7.1 Hz, 2H), 3.96 (q, *J* = 7.1 Hz, 2H), 1.32 (t, *J* = 7.1 Hz, 3H), 0.98 (t, *J* = 7.1 Hz, 3H) ppm. ^13^C­{^1^H} NMR (101 MHz, DMSO-*d*_6_): δ 171.9, 159.7, 137.7, 134.9, 132.0, 131.7, 130.6, 127.6, 110.1, 74.4, 60.4, 15.3, 13.6 ppm. LR-MS (EI) calc. for (M^+•^): 318.0, found: 318.0.

#### Ethyl (2*E*)-3-Ethoxy-2-[3-(trifluoromethyl)­benzenesulfonyl)­prop-2-enoate (**X12l**)

**X12l** was synthesized as a crude product from **X11l** following the procedure described for **X6** as a yellow solid (19% yield) and used without further purification. LR-MS (EI) calc. (M^+•^): 352.1, found: 352.1.

#### Ethyl (2*E*)-2-{[1,1′-Biphenyl]-4-sulfonyl}-3-ethoxyprop-2-enoate (**X12m**)

**X12m** was synthesized from **X11m** following the procedure described for **X6** as a yellow solid (42% yield). ^1^H NMR (300 MHz, CDCl_3_): δ 8.15 (s, 1H), 8.06–7.94 (m, 2H), 7.75–7.65 (m, 2H), 7.65–7.55 (m, 2H), 7.53–7.37 (m, 3H), 4.38 (q, *J* = 7.1 Hz, 2H), 4.15 (q, *J* = 7.1 Hz, 2H), 1.47 (t, *J* = 7.1 Hz, 3H), 1.18 (t, *J* = 7.1 Hz, 3H) ppm. ^13^C­{^1^H} NMR (75 MHz, CDCl_3_): δ 168.3, 160.6, 146.0, 140.1, 139.5, 129.1 (2C), 128.8 (2C), 128.6, 127.5 (2C), 127.4 (2C), 113.8, 74.7, 61.1, 27.0, 15.4, 14.0. HR-MS (ESI) calc. for [M + H]^+^: 361.1105, found: 361.1104.

#### Ethyl (2*E*)-2-(4-Benzylbenzenesulfonyl)-3-ethoxyprop-2-enoate (**X12n**)

**X12n** was synthesized from **X11n** following the procedure described for **X6** as a yellow solid (19% yield). ^1^H NMR (400 MHz, CDCl_3_): δ 8.09 (s, 1H), 7.84 (d, *J* = 8.4 Hz, 2H), 7.33–7.27 (m, 2H), 7.22 (t, *J* = 7.3 Hz, 1H), 7.15 (dd, *J* = 18.1, 6.6 Hz, 1H), 4.35 (q, *J* = 7.1 Hz, 2H), 4.12 (q, *J* = 7.1 Hz, 2H), 4.04 (s, 2H), 1.45 (t, *J* = 7.1 Hz, 3H), 1.13 (t, *J* = 7.1 Hz, 3H) ppm. ^13^C­{^1^H} NMR (101 MHz, CDCl_3_): δ 168.1, 160.6, 146.9, 139.8, 139.2, 129.2 (2C), 129.1 (2C), 128.8 (2C), 128.6 (2C), 126.7, 113.9, 74.6, 61.1, 41.9, 15.5, 14.0 ppm. LR-MS (EI) calc. for (M^+•^): 374.1, found: 374.1.

#### Ethyl (2*E*)-3-Ethoxy-2-(pyridine-2-sulfonyl)­prop-2-enoate (**X12o**)

**X12o** was synthesized from **X11o** following the procedure described for **X6** as a yellow solid (43% yield). ^1^H NMR (400 MHz, DMSO-*d*_6_): δ 8.72–8.69 (m, 1H), 8.20 (s, 1H), 8.16–8.06 (m, 2H), 7.71–7.66 (m, 1H), 4.52 (q, *J* = 7.1 Hz, 2H), 3.94 (q, *J* = 7.1 Hz, 2H), 1.33 (t, *J* = 7.1 Hz, 3H), 0.93 (t, *J* = 7.1 Hz, 3H) ppm. ^13^C­{^1^H} NMR (101 MHz, DMSO-*d*_6_): δ 170.5, 159.9, 158.1, 149.8, 138.5, 127.3, 122.0, 109.2, 74.4, 60.3, 15.2, 13.6 ppm. LR-MS (EI) calc. for (M^+•^): 286.1, found: 286.1.

#### Ethyl (2*E*)-3-Ethoxy-2-(naphthalene-2-sulfonyl)­prop-2-enoate (**X12p**)

**X12p** was synthesized from **X11p** following the procedure described for **X6** as a yellow solid (17% yield). ^1^H NMR (400 MHz, CDCl_3_): δ 8.54 (d, *J* = 2.1 Hz, 1H), 8.19 (s, 1H), 7.99–7.94 (m, 2H), 7.93–7.88 (m, 2H), 7.86 (dd, *J* = 8.7, 1.9 Hz, 1H), 7.66–7.57 (m, 2H) 4.40 (q, *J* = 7.1 Hz, 2H), 4.09 (q, *J* = 7.1 Hz, 2H), 1.48 (t, *J* = 7.1 Hz, 3H), 1.11 (t, *J* = 7.1 Hz, 3H) ppm. ^13^C­{^1^H} NMR (101 MHz, CDCl_3_): δ 168.4, 160.6, 138.3, 135.1, 132.2, 130.1, 129.5, 129.0, 128.9, 128.0, 127.5, 123.2, 113.8, 74.7, 61.1, 15.5, 14.0 ppm. LR-MS (EI) calc. for (M^+•^): 334.1, found: 334.1.

#### Ethyl (2*E*)-3-Ethoxy-2-(quinoline-8-sulfonyl)­prop-2-enoate (**X12q**)

**X12q** was synthesized from **X11q** following the procedure described for **X6** as a yellow solid (21% yield). ^1^H NMR (400 MHz, DMSO-*d*_6_): δ 9.02 (dd, *J* = 4.3, 1.8 Hz, 1H), 8.53 (dd, *J* = 8.4, 1.8 Hz, 1H), 8.47 (d, *J* = 1.5 Hz, 0H), 8.45 (d, *J* = 1.5 Hz, 1H), 8.45 (s, 1H), 4.54 (q, *J* = 7.1 Hz, 2H), 3.89 (q, *J* = 7.1 Hz, 2H), 1.38 (t, *J* = 7.1 Hz, 3H), 0.93 (t, *J* = 7.1 Hz, 3H). ^13^C­{^1^H} NMR (101 MHz, DMSO-*d*_6_): δ 172.2, 160.4, 151.2, 142.6, 137.0, 136.3, 134.6, 132.3, 128.5, 125.6, 122.3, 111.9, 73.9, 60.1, 15.3, 13.6 ppm. HR-MS (ESI) calc. for [M + H]^+^: 336.0901, found: 336.0895.

#### 5-(4-Methylbenzenesulfonyl)-2-sulfanyl-1,4-dihydropyrimidin-4-one (**X13a**)

**X13a** was synthesized from **X12a** following the procedure described for **X7** as a yellow solid (96% yield). ^1^H NMR (400 MHz, DMSO-*d*_6_): δ 13.14 (br s, 1H), 12.98 (br s, 1H), 8.06 (s, 1H), 7.85 (d, *J* = 8.4 Hz, 2H), 7.40 (d, *J* = 8.1 Hz, 2H), 2.38 (s, 3H) ppm. ^13^C­{^1^H} NMR (101 MHz, DMSO-*d*_6_): δ 177.2, 155.7, 146.4, 144.4, 136.9, 129.5 (2C), 128.2 (2C), 116.8, 21.1 ppm. HR-MS (ESI) calc. for [M + H]^+^: 283.0206, found: 283.0215.

#### 5-(3-Chlorobenzenesulfonyl)-2-sulfanyl-1,4-dihydropyrimidin-4-one (**X13b**)

**X13b** was synthesized from **X12b** following the procedure described for **X7** as a yellow solid (95% yield). ^1^H NMR (400 MHz, DMSO-*d*_6_): δ 13.20 (br s, 1H), 13.00 (br s, 1H), 8.09 (s, 1H), 8.01 (t, *J* = 1.9 Hz, 1H), 7.98–7.90 (m, 1H), 7.83–7.75 (m, 1H), 7.65 (t, *J* = 8.0 Hz, 1H) ppm. ^13^C­{^1^H} NMR (101 MHz, DMSO-*d*_6_): δ 177.2, 155.7, 147.3, 141.7, 133.7, 133.6, 131.0, 127.6, 126.8, 115.5 ppm. HR-MS (ESI) calc. for [M + H]^+^: 302.9660, found: 302.9671.

#### 5-(4-Fluorobenzenesulfonyl)-2-sulfanyl-1,4-dihydropyrimidin-4-one (**X13c**)

**X13c** was synthesized from **X12c** following the procedure described for **X7** as a yellow solid (91% yield). ^1^H NMR (400 MHz, DMSO-*d*_6_): δ 13.16 (br s, 1H), 12.99 (br s, 1H), 8.10–8.00 (m, 3H), 7.50–7.40 (m, 2H) ppm. ^13^C­{^1^H} NMR (101 MHz, DMSO-*d*_6_): δ 177.2, 165.0 (d, *J* = 252.8 Hz), 155.6, 146.7, 136.1 (d, *J* = 2.9 Hz), 131.4 (d, *J* = 9.9 Hz, 2C), 116.3, 116.2 (d, *J* = 22.8 Hz, 2C) ppm. ^19^F NMR (376 MHz, DMSO-*d*_6_): δ −104.78 (tt, *J* = 8.1, 5.3 Hz) ppm. HR-MS (ESI) calc. for [M + H]^+^: 286.9955, found: 286.9961.

#### 5-(4-Chlorobenzenesulfonyl)-2-sulfanyl-1,4-dihydropyrimidin-4-one (**X13d**)

**X13d** was synthesized from **X12d** following the procedure described for **X7** as a yellow solid (97% yield). ^1^H NMR (400 MHz, DMSO-*d*_6_): δ 13.17 (br s, 1H), 13.00 (br s, 1H), 8.08 (s, 1H), 8.01–7.95 (m, 2H), 7.72–7.67 (m, 2H) ppm. ^13^C­{^1^H} NMR (101 MHz, DMSO-*d*_6_): δ 177.2, 155.6, 146.9, 138.8, 138.6, 130.1 (2C), 129.1 (2C), 116.0 ppm. HR-MS (ESI) calc. for [M + H]^+^: 302.9660, found: 302.9674.

#### 5-(4-Bromobenzenesulfonyl)-2-sulfanyl-1,4-dihydropyrimidin-4-one (**X13e**)

**X13e** was synthesized from **X12e** following the procedure described for **X7** as a yellow solid (89% yield). ^1^H NMR (400 MHz, DMSO-*d*_6_): δ 13.17 (br s, 1H), 13.00 (br s, 1H), 8.07 (s, 1H), 7.92–7.87 (m, 2H), 7.85–7.80 (m, 2H) ppm. ^13^C­{^1^H} NMR (101 MHz, DMSO-*d*_6_): δ 177.2, 155.6, 146.9, 139.0, 132.1 (2C), 130.1 (2C), 127.9, 115.9 ppm. HR-MS (ESI) calc. for [M + Na]^+^: 368.8974, found: 368.8996.

#### 5-(4-Iodobenzenesulfonyl)-2-sulfanyl-1,4-dihydropyrimidin-4-one (**X13f**)

**X13f** was synthesized from **X12f** following the procedure described for **X7** as a yellow solid (93% yield). ^1^H NMR (400 MHz, DMSO-*d*_6_): δ 13.16 (br s, 1H), 12.99 (br s, 1H), 8.06 (s, 1H), 8.02–7.98 (m, 2H), 7.75–7.69 (m, 2H) ppm. ^13^C­{^1^H} NMR (101 MHz, DMSO-*d*_6_): δ 177.2, 155.6, 146.8, 139.4, 137.9 (2C), 129.7 (2C), 116.0, 102.5 ppm. HR-MS (ESI) calc. for [M + H]^+^: 394.9016, found: 394.9000.

#### 2-Sulfanyl-5-[4-(trifluoromethyl)­benzenesulfonyl]-1,4-dihydropyrimidin-4-one (**X13g**)

**X13g** was synthesized from **X12g** following the procedure described for **X7** as a yellow solid (91% yield). ^1^H NMR (400 MHz, DMSO-*d*_6_): δ 13.20 (br s, 1H), 13.02 (br s, 1H), 8.22–8.18 (m, 2H), 8.12 (s, 1H), 8.00 (d, *J* = 8.4 Hz, 2H) ppm. ^13^C­{^1^H} NMR (101 MHz, DMSO-*d*_6_): δ 177.3, 155.7, 147.4, 143.7, 133.2 (q, *J* = 32.3 Hz), 129.1 (2C), 126.2 (q, *J* = 3.8 Hz, 2C), 123.4 (q, *J* = 273.2 Hz), 115.4 ppm. ^19^F NMR (376 MHz, DMSO-*d*_6_): δ −61.70 (s) ppm. HR-MS (ESI) calc. for [M + H]^+^: 336.9923, found: 336.9915.

#### 5-(4-Nitrobenzenesulfonyl)-2-sulfanyl-1,4-dihydropyrimidin-4-one (**X13h**)

**X13h** was synthesized from **X12h** following the procedure described for **X7** as a yellow solid (89% yield). ^1^H NMR (400 MHz, DMSO-*d*_6_): δ 13.26 (br s, 1H), 13.04 (br s, 1H), 8.43–8.38 (m, 2H), 8.26–8.22 (m, 2H), 8.14 (s, 1H) ppm. ^13^C­{^1^H} NMR (101 MHz, DMSO-*d*_6_): δ 177.3, 155.7, 150.4, 147.6, 145.1, 129.7 (2C), 124.2 (2C), 115.1 ppm. HR-MS (ESI) calc. for [M + Na]^+^: 335.9720, found: 335.9727.

#### 5-(4-Methoxybenzenesulfonyl)-2-sulfanyl-1,4-dihydropyrimidin-4-one (**X13i**)

**X13i** was synthesized from **X12i** following the procedure described for **X7** as a yellow solid (93% yield). ^1^H NMR (400 MHz, DMSO-*d*_6_): δ 13.08 (br s, 1H), 12.94 (br s, 1H), 8.04 (s, 1H), 7.93–7.85 (m, 2H), 7.17–7.07 (m, 2H), 3.84 (s, 3H) ppm. ^13^C­{^1^H} NMR (101 MHz, DMSO-*d*_6_): δ 177.1, 163.3, 155.6, 145.8, 131.2, 130.5 (2C), 117.3, 114.2 (2C), 55.8 ppm. HR-MS (ESI) calc. for [M + H]^+^: 299.0155, found: 299.0151.

#### 5-(Benzenesulfonyl)-2-sulfanyl-1,4-dihydropyrimidin-4-one (**X13j**)

**X13j** was synthesized from **X12j** following the procedure described for **X7** as a yellow solid (93% yield). ^1^H NMR (400 MHz, DMSO-*d*_6_): δ 13.14 (br s, 1H), 12.97 (br s, 1H), 8.08 (s, 1H), 7.97 (d, *J* = 7.4 Hz, 2H), 7.71 (t, *J* = 7.4 Hz, 1H), 7.61 (t, *J* = 7.7 Hz, 2H) ppm. ^13^C­{^1^H} NMR (101 MHz, DMSO-*d*_6_): δ 177.2, 155.6, 146.6, 139.8, 133.7, 129.0 (2C), 128.0 (2C), 116.4 ppm. HR-MS (ESI) calc. for [M + H]^+^: 269.0050, found: 269.0066.

#### 5-(2-Chlorobenzenesulfonyl)-2-sulfanyl-1,4-dihydropyrimidin-4-one (**X13k**)

**X13k** was synthesized from **X12k** following the procedure described for **X7** as a yellow solid (97% yield). ^1^H NMR (400 MHz, DMSO-*d*_6_): δ 13.31 (br s, 1H), 13.08 (br s, 1H), 8.21–8.14 (m, 2H), 7.77–7.68 (m, 1H), 7.67–7.61 (m, 3H) ppm. ^13^C­{^1^H} NMR (101 MHz, DMSO-*d*_6_): δ 177.2, 155.4, 148.2, 136.2, 135.6, 132.4, 131.9, 131.0, 127.9, 114.8 ppm. HR-MS (ESI) calc. for [M + H]^+^: 302.9660, found: 302. 9659.

#### 2-Sulfanyl-5-[3-(trifluoromethyl)­benzenesulfonyl]-1,4-dihydropyrimidin-4-one (**X13l**)

**X13l** was synthesized from **X12l** following the procedure described for **X7** as a yellow solid (92% yield). ^1^H NMR (400 MHz, DMSO-*d*_6_): δ 13.22 (br s, 1H), 13.01 (s, 1H), 8.31–8.26 (m, 2H), 8.17–8.07 (m, 2H), 7.87 (t, *J* = 8.1 Hz, 1H) ppm. ^13^C­{^1^H} NMR (101 MHz, DMSO-*d*_6_): δ 177.3, 155.7, 147.4, 141.0, 132.3, 130.6, 130.5 (d, *J* = 4.6 Hz), 129.6 (q, *J* = 32.9 Hz), 124.9 (d, *J* = 4.1 Hz), 123.4 (d, *J* = 272.8 Hz), 115.4 ppm. ^19^F NMR (376 MHz, DMSO-*d*_6_): δ −61.30 (s) ppm. HR-MS (ESI) calc. for [M + H]^+^: 336.9923, found: 336.9915.

#### 5-{[1,1′-Biphenyl]-4-sulfonyl}-2-sulfanyl-1,4-dihydropyrimidin-4-one (**X13m**)

**X13m** was synthesized from **X12m** following the procedure described for **X7** as a yellow solid (35% yield). ^1^H NMR (400 MHz, DMSO-*d*_6_): δ 12.99 (br s, 1H), 8.12 (s, 1H), 8.04 (d, *J* = 8.6 Hz, 2H), 7.89 (d, *J* = 8.5 Hz, 2H), 7.73 (d, *J* = 7.3 Hz, 2H), 7.51 (t, *J* = 7.4 Hz, 2H), 7.44 (t, *J* = 7.3 Hz, 1H) ppm. ^13^C­{^1^H} NMR (101 MHz, DMSO-*d*_6_): δ 177.3, 155.8, 146.7, 145.3, 138.5, 138.5, 129.2 (2C), 128.9 (2C), 128.8, 127.3 (4C), 116.6 ppm. HR-MS (ESI) calc. for [M + H]^+^: 345.0363, found: 345.0358.

#### 5-(4-Benzylbenzenesulfonyl)-2-sulfanyl-1,4-dihydropyrimidin-4-one (**X13n**)

**X13n** was synthesized as a crude product from **X12n** following the procedure described for **X7** as a yellow solid (33% yield) and used without further purification. LR-MS (ESI) calc. for [M + H]^+^: 357.0, found: 357.2.

#### 5-(Pyridine-2-sulfonyl)-2-sulfanyl-1,4-dihydropyrimidin-4-one (**X13o**)

**X13o** was synthesized from **X12o** following the procedure described for **X7** as a yellow solid (91% yield). ^1^H NMR (400 MHz, DMSO-*d*_6_): δ 13.24 (br s, 1H), 13.03 (br s, 1H), 8.70 (dt, *J* = 4.7, 1.3 Hz, 1H), 8.17–8.15 (m, 2H), 8.13 (s, 1H), 7.76–7.68 (m, 1H) ppm. ^13^C­{^1^H} NMR (101 MHz, DMSO-*d*_6_): δ 177.2, 156.7, 155.7, 150.1, 147.6, 138.8, 128.0, 122.6, 114.3 ppm. HR-MS (ESI) calc. for [M + H]^+^: 270.0002, found: 270.0003.

#### 5-(Naphthalene-2-sulfonyl)-2-sulfanyl-1,4-dihydropyrimidin-4-one (**X13p**)

**X13p** was synthesized as a crude product from **X12p** following the procedure described for **X7** as a yellow solid (16% yield) and used without further purification. LR-MS (ESI) calc. for [M-H]^−^: 317.0, found: 316.9.

#### 5-(Quinoline-8-sulfonyl)-2-sulfanyl-1,4-dihydropyrimidin-4-one (**X13q**)

**X13q** was synthesized from **X12q** following the procedure described for **X7** as a yellow solid (53% yield). ^1^H NMR (400 MHz, DMSO-*d*_6_): δ 13.20 (br s, 1H), 12.86 (br s, 1H), 8.94 (dd, *J* = 4.2, 1.8 Hz, 1H), 8.54 (t, *J* = 2.0 Hz, 1H), 8.52 (t, *J* = 1.5 Hz, 1H), 8.38 (dd, *J* = 8.3, 1.5 Hz, 1H), 8.33 (s, 1H), 7.85 (t, *J* = 7.8 Hz, 1H), 7.66 (dd, *J* = 8.3, 4.2 Hz, 1H) ppm. ^13^C­{^1^H} NMR (101 MHz, DMSO-*d*_6_): δ 176.8, 155.6, 151.5, 148.3, 142.7, 137.1, 135.2, 134.9, 132.7, 128.5, 125.7, 122.5, 117.1 ppm. HR-MS (ESI) calc. for [M + H]^+^: 320.0159, found: 320.0163.

#### 2-{[5-(4-Methylbenzenesulfonyl)-4-oxo-1,4-dihydropyrimidin-2-yl]­sulfanyl}-*N*-[4-(trifluoromethyl)­phenyl]­acetamide (Compound **27**)

Compound **27** was synthesized from **X13a** and **X8e** following the procedure described for compound **1** as a yellow solid (94% yield). ^1^H NMR (400 MHz, DMSO-*d*_6_): δ 11.18 (br s, 1H), 8.27 (s, 1H), 7.86–7.79 (m, 2H), 7.75 (d, *J* = 8.1 Hz, 2H), 7.62 (d, *J* = 8.6 Hz, 2H), 7.36–7.28 (m, 2H), 3.86 (s, 2H), 2.35 (s, 3H) ppm. ^13^C­{^1^H} NMR (101 MHz, DMSO-*d*_6_): δ 172.6, 168.0, 164.1, 155.7, 142.8, 142.5, 139.1, 128.9 (2C), 127.7 (2C), 126.1 (q, *J* = 3.6 Hz, 2C), 124.4 (d, *J* = 271.3 Hz), 123.2 (q, *J* = 32.0 Hz), 118.8 (2C), 118.5, 35.2, 21.0 ppm. ^19^F NMR (376 MHz, DMSO-*d*_6_): δ −60.32 (s) ppm. HR-MS (ESI) calc. for [M + H]^+^: 484.0608, found: 484.0616.

#### 2-{[5-(3-Chlorobenzenesulfonyl)-4-oxo-1,4-dihydropyrimidin-2-yl]­sulfanyl}-*N*-[4-(trifluoromethyl)­phenyl]­acetamide (Compound **28**)

Compound **28** was synthesized from **X13b** and **X8e** following the procedure described for compound **1** as a yellow solid (57% yield). ^1^H NMR (400 MHz, DMSO-*d*_6_): δ 11.13 (br s, 1H), 8.25 (s, 1H), 8.01 (t, *J* = 1.9 Hz, 1H), 7.87 (dt, *J* = 7.8, 1.4 Hz, 1H), 7.76 (d, *J* = 8.5 Hz, 2H), 7.68 (ddd, *J* = 8.0, 2.2, 1.1 Hz, 1H), 7.63 (d, *J* = 8.6 Hz, 2H), 7.56 (t, *J* = 7.9 Hz, 1H), 3.85 (s, 2H) ppm. ^13^C­{^1^H} NMR (101 MHz, DMSO-*d*_6_): δ 173.5, 168.0, 164.3, 156.3, 144.0, 142.5, 133.0, 132.4, 130.6, 127.3, 126.1, 126.1 (2C), 124.3 (d, *J* = 271.1 Hz), 123.2 (d, *J* = 32.0 Hz), 118.8 (2C), 117.1, 35.3 ppm. ^19^F NMR (377 MHz, DMSO-*d*_6_): δ −60.30 (s) ppm. HR-MS (ESI) calc. for [M + H]^+^: 504.0061, found: 504.0060.

#### 2-{[5-(4-Fluorobenzenesulfonyl)-4-oxo-1,4-dihydropyrimidin-2-yl]­sulfanyl}-*N*-[4-(trifluoromethyl)­phenyl]­acetamide (Compound **29**)

Compound **29** was synthesized from **X13c** and **X8e** following the procedure described for compound **1** as a yellow solid (58% yield). ^1^H NMR (400 MHz, DMSO-*d*_6_): δ 13.76 (br s, 1H), 10.70 (br s, 1H), 8.51 (s, 1H), 8.09–7.99 (m, 2H), 7.78 (d, *J* = 8.5 Hz, 2H), 7.68 (d, *J* = 8.6 Hz, 2H), 7.49–7.38 (m, 2H), 4.25 (s, 2H) ppm. ^13^C­{^1^H} NMR (101 MHz, DMSO): δ 168.4, 165.7, 165.0 (d, *J* = 252.7 Hz), 157.3, 155.3, 142.3 (d, *J* = 0.9 Hz), 136.2 (d, *J* = 2.8 Hz), 131.4 (d, *J* = 9.9 Hz, 2C), 126.2 (q, *J* = 3.9 Hz, 2C), 124.3 (q, *J* = 271.4 Hz), 123.6 (q, *J* = 32.1 Hz), 121.4, 119.1 (2C), 116.2 (d, *J* = 22.8 Hz, 2C), 35.5 ppm. ^19^F NMR (377 MHz, DMSO-*d*_6_): δ −60.39 (s), −104.82 (tt, *J* = 8.8, 5.1 Hz) ppm. HR-MS (ESI) calc. for [M + H]^+^: 488.0357, found: 488.0359.

#### 2-{[5-(4-Chlorobenzenesulfonyl)-4-oxo-1,4-dihydropyrimidin-2-yl]­sulfanyl}-*N*-[4-(trifluoromethyl)­phenyl]­acetamide (Compound **30**)

Compound **30** was synthesized from **X13d** and **X8e** following the procedure described for compound **1** as a yellow solid (69% yield). ^1^H NMR (400 MHz, DMSO-*d*_6_): δ 11.15 (br s, 1H), 8.25 (s, 1H), 7.99–7.90 (m, 2H), 7.74 (d, *J* = 8.5 Hz, 2H), 7.66–7.55 (m, 4H), 3.84 (s, 2H) ppm. ^13^C­{^1^H} NMR (101 MHz, DMSO-*d*_6_): δ 173.4, 168.0, 164.4, 156.0, 142.5, 140.9, 137.3, 129.6 (2C), 128.5 (2C), 126.1 (q, *J* = 3.8 Hz, 2C), 124.3 (d, *J* = 271.3 Hz), 123.2 (q, *J* = 32.1 Hz), 118.8 (2C), 117.5, 35.2 ppm. ^19^F NMR (377 MHz, DMSO-*d*_6_): δ −60.29 (s) ppm. HR-MS (ESI) calc. for [M + H]^+^: 504.0061, found: 504.0055.

#### 2-{[5-(4-Bromobenzenesulfonyl)-4-oxo-1,4-dihydropyrimidin-2-yl]­sulfanyl}-*N*-[4-(trifluoromethyl)­phenyl]­acetamide (Compound **31**)

Compound **31** was synthesized from **X13e** and **X8e** following the procedure described for compound **1** as a yellow solid (72% yield). ^1^H NMR (400 MHz, DMSO-*d*_6_): δ 11.10 (br s, 1H), 8.28 (s, 1H), 7.95–7.80 (m, 2H), 7.77–7.72 (m, 4H), 7.63 (d, *J* = 8.6 Hz, 2H), 3.89 (s, 2H) ppm. ^13^C­{^1^H} NMR (101 MHz, DMSO-*d*_6_): δ 172.8, 167.7, 155.9, 142.5, 141.1, 131.5 (2C), 129.8 (2C), 126.5, 126.1 (q, *J* = 3.6 Hz, 2C), 124.3 (d, *J* = 271.2 Hz), 123.3 (q, *J* = 31.9 Hz), 118.8 (2C), 117.9, 35.3 ppm. ^19^F NMR (377 MHz, DMSO-*d*_6_): δ −60.29 (s) ppm. HR-MS (ESI) calc. for [M + H]^+^: 547.9556, found: 547.9561.

#### 2-{[5-(4-Iodobenzenesulfonyl)-4-oxo-1,4-dihydropyrimidin-2-yl]­sulfanyl}-*N*-[4-(trifluoromethyl)­phenyl]­acetamide (Compound **32**)

Compound **32** was synthesized from **X13f** and **X8e** following the procedure described for compound **1** as a yellow solid (87% yield). ^1^H NMR (400 MHz, DMSO-*d*_6_): δ 11.15 (br s, 1H), 8.25 (s, 1H), 7.93–7.89 (m, 2H), 7.75 (d, *J* = 8.5 Hz, 2H), 7.72–7.67 (m, 2H), 7.63 (d, *J* = 8.6 Hz, 2H), 3.85 (s, 2H) ppm. ^13^C­{^1^H} NMR (101 MHz, DMSO-*d*_6_): δ 173.1, 167.9, 156.0, 142.5, 142.5, 141.6, 137.3 (2C), 129.4 (2C), 126.1 (q, *J* = 3.8 Hz, 2C), 124.3 (d, *J* = 271.2 Hz), 123.2 (d, *J* = 32.1 Hz), 118.8 (2C), 117.7, 100.6, 35.3 ppm. ^19^F NMR (377 MHz, DMSO-*d*_6_): δ −60.24 (s) ppm. HR-MS (ESI) calc. for [M + H]^+^: 595.9418, found: 595.9423.

#### 2-({4-Oxo-5-[4-(trifluoromethyl)­benzenesulfonyl]-1,4-dihydropyrimidin-2-yl}­sulfanyl)-*N*-[4-(trifluoromethyl)­phenyl]­acetamide (Compound **33**)

Compound **33** was synthesized from **X13g** and **X8e** following the procedure described for compound **1** as a yellow solid (81% yield). ^1^H NMR (400 MHz, DMSO-*d*_6_): δ 10.90 (br s, 0H), 8.34 (s, 1H), 8.12 (d, *J* = 8.2 Hz, 2H), 7.90 (d, *J* = 8.3 Hz, 2H), 7.70 (d, *J* = 8.6 Hz, 2H), 7.58 (d, *J* = 8.6 Hz, 2H), 3.91 (s, 2H) ppm. ^13^C­{^1^H} NMR (101 MHz, DMSO-*d*_6_): δ 172.7 (q, *J* = 32.3 Hz), 167.9, 163.1, 156.6, 145.5, 142.7, 133.0 (q, *J* = 32.1 Hz), 129.2 (2C), 126.5 (d, *J* = 4.2 Hz, 2C), 126.3 (q, *J* = 3.9 Hz, 2C), 124.7 (q, *J* = 271.4 Hz), 123.9 (d, *J* = 272.8 Hz), 123.9 (d, *J* = 32.0 Hz), 119.5 (2C), 118.2, 35.6 ppm. ^19^F NMR (376 MHz, DMSO-*d*_6_): δ −60.43 (s), −61.68 (s) ppm. HR-MS (ESI) calc. for [M + H]^+^: 538.0325, found: 538.0317.

#### 2-{[5-(4-Nitrobenzenesulfonyl)-4-oxo-1,4-dihydropyrimidin-2-yl]­sulfanyl}-*N*-[4-(trifluoromethyl)­phenyl]­acetamide (Compound **34**)

Compound **34** was synthesized from **X13h** and **X8e** following the procedure described for compound **1** as a yellow solid (97% yield). ^1^H NMR (400 MHz, DMSO-*d*_6_): δ 11.05 (br s, 1H), 8.38–8.29 (m, 2H), 8.30 (s, 1H), 8.23–8.15 (m, 2H), 7.75 (d, *J* = 8.2 Hz, 2H), 7.62 (d, *J* = 8.3 Hz, 1H), 3.86 (s, 2H) ppm. ^13^C­{^1^H} NMR (101 MHz, DMSO-*d*_6_): δ 173.9, 167.9, 164.5, 156.5, 149.6, 147.5, 142.5 (q, *J* = 1.3 Hz), 129.2 (2C), 126.1 (q, *J* = 3.8 Hz, 2C), 125.7, 124.3 (d, *J* = 271.3 Hz), 123.8 (2C), 123.2 (q, *J* = 32.0 Hz), 118.8 (2C), 116.5, 35.3 ppm. ^19^F NMR (376 MHz, DMSO-*d*_6_): δ −60.35 (s) ppm. HR-MS (ESI) calc. for [M + H]^+^: 515.0302, found: 515.0307.

#### 2-{[5-(4-Methoxybenzenesulfonyl)-4-oxo-1,4-dihydropyrimidin-2-yl]­sulfanyl}-*N*-[4-(trifluoromethyl)­phenyl]­acetamide (Compound **35**)

Compound **35** was synthesized from **X13i** and **X8e** following the procedure described for compound **1** as a yellow solid (98% yield). ^1^H NMR (400 MHz, DMSO-*d*_6_): δ 11.20 (br s, 1H), 8.26 (s, 1H), 7.93–7.84 (m, 2H), 7.75 (d, *J* = 8.5 Hz, 2H), 7.62 (d, *J* = 8.5 Hz, 2H), 7.08–7.00 (m, 2H), 3.86 (s, 2H), 3.81 (s, 3H) ppm. ^13^C­{^1^H} NMR (101 MHz, DMSO-*d*_6_): δ 172.4, 168.0, 164.0, 162.4, 155.4, 142.5, 133.6, 130.0, 126.1 (q, *J* = 3.8 Hz), 124.4 (q, *J* = 271.3 Hz), 123.3 (q, *J* = 32.1 Hz), 119.1, 118.8, 113.6, 55.6, 35.2 ppm. ^19^F NMR (377 MHz, DMSO-*d*_6_): δ −60.31 (s) ppm. HR-MS (ESI) calc. for [M + H]^+^: 500.0557, found: 500.0556.

#### 2-{[5-(Benzenesulfonyl)-4-oxo-1,4-dihydropyrimidin-2-yl]­sulfanyl}-*N*-[4-(trifluoromethyl)­phenyl]­acetamide (Compound **36**)

Compound **36** was synthesized from **X13j** and **X8e** following the procedure described for compound **1** as a yellow solid (70% yield). ^1^H NMR (400 MHz, DMSO-*d*_6_): δ 11.20 (br s, 1H), 8.25 (s, 1H), 7.99–7.91 (m, 2H), 7.76 (d, *J* = 8.4 Hz, 2H), 7.63 (d, *J* = 8.7 Hz, 2H), 7.58 (d, *J* = 7.2 Hz, 1H), 7.52 (t, *J* = 7.5 Hz, 2H), 3.82 (s, 2H) ppm. ^13^C­{^1^H} NMR (101 MHz, DMSO-*d*_6_): δ 173.4, 168.2, 164.9, 156.1, 142.6, 142.2, 132.3, 128.4 (2C), 127.5 (2C), 126.1 (q, *J* = 3.9 Hz, 2C), 124.4 (d, *J* = 271.4 Hz),123.2 (q, *J* = 32.0 Hz), 118.8 (2C), 117.7, 35.2 ppm. ^19^F NMR (376 MHz, DMSO-*d*_6_): δ −60.30 (s) ppm. HR-MS (ESI) calc. for [M + H]^+^: 470.0451, found: 470.0430.

#### 2-{[5-(2-Chlorobenzenesulfonyl)-4-oxo-1,4-dihydropyrimidin-2-yl]­sulfanyl}-*N*-[4-(trifluoromethyl)­phenyl]­acetamide (Compound **37**)

Compound **37** was synthesized from **X13k** and **X8e** following the procedure described for compound **1** as a yellow solid (96% yield). ^1^H NMR (400 MHz, DMSO-*d*_6_): δ 11.09 (br s, 1H), 8.33 (s, 1H), 8.18 (dd, *J* = 7.7, 2.0 Hz, 1H), 7.75 (d, *J* = 8.5 Hz, 2H), 7.66–7.59 (m, 2H), 7.60–7.56 (m, 1H), 7.51 (dd, *J* = 7.6, 1.6 Hz, 1H), 3.88 (s, 2H) ppm. ^13^C­{^1^H} NMR (101 MHz, DMSO-*d*_6_): δ 173.0, 167.9, 157.8, 142.5, 138.3, 134.1, 132.5, 131.2, 130.6, 128.4, 127.1, 126.1 (q, *J* = 3.3 Hz, 2C), 124.4 (d, *J* = 271.4 Hz) 123.3 (d, *J* = 32.1 Hz), 118.8 (2C), 116.0, 35.3 ppm. ^19^F NMR (377 MHz, DMSO-*d*_6_): δ −60.29 (s) ppm. HR-MS (ESI) calc. for [M + H]^+^: 504.0061, found: 504.0064.

#### 2-({4-Oxo-5-[3-(trifluoromethyl)­benzenesulfonyl]-1,4-dihydropyrimidin-2-yl}­sulfanyl)-*N*-[4-(trifluoromethyl)­phenyl]­acetamide (Compound **38**)

Compound **38** was synthesized from **X13l** and **X8e** following the procedure described for compound **1** as a white solid (84% yield). ^1^H NMR (400 MHz, DMSO-*d*_6_): δ 10.68 (br s, 1H), 8.53 (s, 1H), 8.29–8.23 (m, 2H), 8.08 (d, *J* = 8.6 Hz, 1H), 7.84 (t, *J* = 7.7 Hz, 1H), 7.76 (d, *J* = 8.5 Hz, 2H), 7.66 (d, *J* = 8.6 Hz, 2H), 4.24 (s, 2H) ppm. ^13^C­{^1^H} NMR (101 MHz, DMSO-*d*_6_): δ 169.2, 166.1, 157.9, 155.9, 142.7, 141.5, 132.7, 131.1, 130.9 (q, *J* = 3.4 Hz), 130.0 (q, *J* = 32.7 Hz), 126.6 (q, *J* = 3.9 Hz, 2C), 125.4 (q, *J* = 4.1 Hz), 124.8 (q, *J* = 271.4 Hz), 124.0 (q, *J* = 32.0 Hz), 123.8 (d, *J* = 272.8 Hz), 121.0, 119.5 (2C), 36.0 ppm. ^19^F NMR (376 MHz, DMSO-*d*_6_): δ −60.42 (s), −61.37 (s) ppm. HR-MS (ESI) calc. for [M + H]^+^: 538.0325, found: 538.0320.

#### 2-[(5-{[1,1′-Biphenyl]-4-sulfonyl}-4-oxo-1,4-dihydropyrimidin-2-yl)­sulfanyl]-*N*-[4-(trifluoromethyl)­phenyl]­acetamide (Compound **39**)

Compound **39** was synthesized from **X13m** and **X8e** following the procedure described for compound **1** as a yellow solid (46% yield). ^1^H NMR (400 MHz, DMSO-*d*_6_): δ 11.23 (br s, 1H), 8.28 (s, 1H), 8.05–7.99 (m, 2H), 7.83–7.78 (m, 2H), 7.76 (d, *J* = 8.5 Hz, 2H), 7.72–7.68 (m, 2H), 7.61 (d, *J* = 8.6 Hz, 2H), 7.53–7.46 (m, 2H), 7.45–7.40 (m, 1H), 3.82 (s, 2H) ppm. ^13^C­{^1^H} NMR (101 MHz, DMSO-*d*_6_): δ 173.4, 168.3, 165.0, 156.1, 144.0, 142.6, 141.0, 138.9, 129.1 (3C), 128.4, 128.3, 127.1 (3C), 126.7, 126.1 (q, *J* = 3.8 Hz, 2C), 124.3 (d, *J* = 271.4 Hz), 123.2 (q, *J* = 32.1 Hz), 118.8 (2C), 117.8, 35.2 ppm. ^19^F NMR (377 MHz, DMSO-*d*_6_): δ −60.31 (s) ppm. HR-MS (ESI) calc. for [M + H]^+^: 546.0764, found: 546.0743.

#### 2-{[5-(4-Benzylbenzenesulfonyl)-4-oxo-1,4-dihydropyrimidin-2-yl]­sulfanyl}-*N*-[4-(trifluoromethyl)­phenyl]­acetamide (Compound **40**)

Compound **40** was synthesized from **X13n** and **X8e** following the procedure described for compound **1** as a yellow solid (37% yield). ^1^H NMR (400 MHz, DMSO-*d*_6_): δ 10.74 (br s, 1H), 8.46 (s, 1H), 7.87 (d, *J* = 8.4 Hz, 2H), 7.78 (d, *J* = 8.5 Hz, 2H), 7.67 (d, *J* = 8.7 Hz, 2H), 7.43 (d, *J* = 8.4 Hz, 2H), 7.34–7.14 (m, 5H), 4.20 (s, 2H), 4.02 (s, 2H) ppm. ^13^C­{^1^H} NMR (101 MHz, DMSO-*d*_6_): δ 168.5, 165.9, 157.9, 154.9, 147.6, 142.3, 140.1, 137.8, 129.1 (2C), 128.8 (2C), 128.6 (2C), 128.4 (2C), 126.3, 126.2 (d, *J* = 4.1 Hz, 2C), 124.3 (d, *J* = 271.3 Hz), 123.5 (d, *J* = 32.1 Hz, 2C), 121.4, 119.1 (2C), 40.8, 35.5 ppm. ^19^F NMR (376 MHz, DMSO-*d*_6_): δ −60.38 (s) ppm. HR-MS (ESI) calc. for [M + H]^+^: 560.0921, found: 560.0929.

#### 2-{[4-Oxo-5-(pyridine-2-sulfonyl)-1,4-dihydropyrimidin-2-yl]­sulfanyl}-*N*-[4-(trifluoromethyl)­phenyl]­acetamide (Compound **41**)

Compound **41** was synthesized from **X13o** and **X8e** following the procedure described for compound **1** as a yellow solid (91% yield). ^1^H NMR (400 MHz, DMSO-*d*_6_): δ 11.16 (br s, 1H), 8.58 (d, *J* = 4.1 Hz, 1H), 8.25 (s, 1H), 8.12 (d, *J* = 7.8 Hz, 1H), 8.07 (td, *J* = 7.6, 1.7 Hz, 1H), 7.75 (d, *J* = 8.5 Hz, 2H), 7.62 (d, *J* = 8.8 Hz, 2H), 7.60–7.57 (m, 1H), 3.83 (s, 2H) ppm. ^13^C­{^1^H} NMR (101 MHz, DMSO-*d*_6_): δ 173.6, 168.2, 165.1, 158.7, 157.1, 149.4, 142.5, 137.9, 126.6, 126.1 (q, *J* = 3.8 Hz, 2C), 124.3 (d, *J* = 271.4 Hz), 123.2 (d, *J* = 32.0 Hz), 122.6, 118.8 (2C), 115.4, 35.2 ppm. ^19^F NMR (377 MHz, DMSO-*d*_6_): δ −60.28 (s) ppm. HR-MS (ESI) calc. for [M + H]^+^: 471.0404, found: 471.0390.

#### 2-{[5-(Naphthalene-2-sulfonyl)-4-oxo-1,4-dihydropyrimidin-2-yl]­sulfanyl}-*N*-[4-(trifluoromethyl)­phenyl]­acetamide (Compound **42**)

Compound **42** was synthesized from **X13p** and **X8e** following the procedure described for compound **1** as a yellow solid (96% yield). ^1^H NMR (400 MHz, DMSO-*d*_6_): δ 11.20 (br s, 1H), 8.59 (d, *J* = 1.8 Hz, 1H), 8.33 (s, 1H), 8.15 (d, *J* = 7.8 Hz, 1H), 8.01 (t, *J* = 8.6 Hz, 2H), 7.92 (dd, *J* = 8.7, 2.0 Hz, 1H), 7.76–7.60 (m, 4H), 7.52 (d, *J* = 8.8 Hz, 2H), 3.80 (s, 2H) ppm. ^13^C­{^1^H} NMR (101 MHz, DMSO-*d*_6_): δ 173.3, 168.1, 164.6, 156.1, 142.5, 139.2, 134.3, 131.5, 129.3, 128.6, 128.6, 128.3, 127.7, 127.2, 126.0 (q, *J* = 3.8 Hz, 2C), 124.3 (d, *J* = 271.3 Hz), 123.1 (d, *J* = 31.5 Hz), 123.3, 118.7 (2C), 117.8, 35.2 ppm. ^19^F NMR (377 MHz, DMSO-*d*_6_): δ −60.28 (s) ppm. HR-MS (ESI) calc. for [M + Na]^+^: 542.0427, found: 540.0424.

#### 2-{[4-Oxo-5-(quinoline-8-sulfonyl)-1,4-dihydropyrimidin-2-yl]­sulfanyl}-*N*-[4-(trifluoromethyl)­phenyl]­acetamide (Compound **43**)

Compound **43** was synthesized from **X13q** and **X8e** following the procedure described for compound **1** as a yellow solid (99% yield). ^1^H NMR (400 MHz, DMSO-*d*_6_): δ 11.26 (br s, 1H), 8.85 (dd, *J* = 4.2, 1.8 Hz, 1H), 8.53 (d, *J* = 1.5 Hz, 1H), 8.51 (s, 1H), 8.43 (dd, *J* = 8.4, 1.8 Hz, 1H), 8.25 (dd, *J* = 8.2, 1.5 Hz, 1H), 7.82–7.76 (m, 1H), 7.71 (d, *J* = 8.5 Hz, 2H), 7.59 (d, *J* = 8.6 Hz, 2H), 7.54 (dd, *J* = 8.3, 4.2 Hz, 1H), 3.77 (s, 2H) ppm. ^13^C­{^1^H} NMR (101 MHz, DMSO-*d*_6_): δ 172.4, 168.3, 164.9, 157.9, 150.9, 143.2, 142.5, 137.4, 136.5, 133.6, 132.5, 128.3, 126.0 (q, *J* = 3.9 Hz, 2C), 125.4, 124.3 (d, *J* = 271.3 Hz), 123.1 (q, *J* = 32.0 Hz), 121.9, 118.7 (2C), 118.3, 35.2 ppm. ^19^F NMR (377 MHz, DMSO-*d*_6_): δ −60.28 (s) ppm. HR-MS (ESI) calc. for [M + H]^+^: 521.0560, found: 521.0547.

### Solid-Phase Peptide Synthesis

Solid-phase peptide synthesis was performed using the fluorenylmethoxycarbonyl (Fmoc)/*tert*-butyl (*t*Bu) strategy. A preloaded Wang resin (Fmoc-l-Ser­(*t*Bu)-Wang TG, IRIS Biotech GmbH) with a loading capacity of 0.2–0.25 mmol/g was elongated with automated synthesis accomplished on a SYRO I synthesis robot (Multisyntech/Biotage) using a scale of 15 μmol per peptide for the synthesis of chemerin-9 and derivatives. Porcine neuropeptide Y was synthesized using a TG R RAM resin (loading capacity of 0.15–0.25 mmol/g, IRIS Biotech GmbH) for its amidated C terminus. All amino acids (ORPEGEN) were dissolved in 0.1 M 1-hydroxybenzotriazole (HOBt, Novabiochem) in *N*,*N*-dimethylformamide (DMF, BioSolve) to a final concentration of 0.3 M each. First, the resins were swollen in DMF for 10 min. Fmoc was cleaved from the preloaded resin with 40% piperidine (Fluka) in DMF for 3 min and 20% piperidine in DMF for 10 min. The amino acid coupling process was performed with 8 equiv of amino acid, *N*,*N*-diisopropylcarbodiimide (DIC, IRIS Biotech GmbH), and ethyl cyanohydroxyiminoacetate (Oxyma, IRIS Biotech GmbH) for 42 min. After washing with DMF, coupling was repeated, followed by Fmoc deprotection and the next amino acid coupling until the peptide sequence was reached. TAMRA coupling was performed manually with 2 equiv of 6-carboxytetramethylrhodamine, 1.9 equiv of *O*-(7-azabenzotriazol-1-yl)-*N*,*N*,*N*,*N*-tetramethyluronium hexafluorophosphate (HATU, Sigma-Aldrich), and 2 equiv of *N*,*N*-diisopropylethylamine (DIPEA, Sigma-Aldrich) at room temperature (22 °C) in the dark within 3 h. For the cleavage of the peptide from the resin, TFA/EDT/TA (90:3:7, v:v:v, Merck, Merck, Sigma-Aldrich) was added and incubated under shaking at room temperature for 3 h. The cleaved peptide was precipitated from ice-cold diethyl ether at −20 °C for at least 1 h or overnight, was washed with diethyl ether, and was collected by centrifugation. After drying the peptide, it was dissolved in 20% of ACN/H_2_O (VWR). RP-HPLC was applied to control the purity and MALDI-MS (matrix-assisted laser desorption/ionization-mass spectrometry) and ESI-MS (electrospray ionization-mass spectrometry) to determine the molecular mass and identity of the peptides, respectively (Table S1).

### Protein Expression

For the expression of His_10_-tagged full-length chemerinS157, the plasmid DNA was transformed into competent Escherichia coli BL21 (DE3).^54,55^ After addition of 1 mM isopropyl β-d-thiogalactopyranoside (IPTG) for the induction of expression, the bacteria were incubated at 37 °C for 6 h, and the cells were harvested and resuspended in base buffer (0.5 M NaCl, 25 mM Tris/HCl, pH 7.8). After cell lysis by using zirconium beads and a high-speed homogenizer, the cell lysates were digested by DNaseI (AppliChem, PanReac A3778,0100 activity 3895.8 U/mg, lot 8C013081). The cell pellet was washed with 0.5% w/v Triton X-100 containing base buffer and base buffer only. The inclusion bodies were solubilized in 8 M urea-containing base buffer. After purification using immobilized Ni-NTA agarose ion chromatography, the protein was eluted from the column with 500 mM imidazole. The protein was refolded stepwise by dialysis, using decreasing urea concentrations and a cysteine/cystamine redox pair. Identity and purity were confirmed via RP-HPLC (Phenomenex Proteo 300 Å C18) and MALDI-TOF-MS (Ultraflex III, Bruker). In a semisynthetic approach, TAMRA-labeled ChemS157 was produced as published recently.^16^ Shortly, [K^141^(TAMRA)]­ChemS157­(135–157), a TAMRA-labeled peptide, and a ChemS157(21–134)-thioester were fused by using native chemical ligation to produce the protein [K^141^(TAMRA)]­ChemS157.

### Cell Culture

HEK293 cells were obtained from Deutsche Sammlung von Mikroorganismen and Zellkulturen (DSMZ) and were cultured in Dulbecco’s modified Eagle’s medium (DMEM)/Ham’s F12 (1:1, v/v, Lonza) supplemented with 15% heat-inactivated fetal bovine serum (FBS, Biochrom). The stable HEK293 cell line containing hCMKLR1b-eYFP and the chimeric G protein Gα_Δ6qi4myr_ were cultured in DMEM/Ham’s F12 (1:1) supplemented with 15% FBS and 100 μg/mL hygromycin (Invivogen). The vector for the chimeric G protein was kindly provided by E. Kostenis (Rheinische Friedrich-Wilhelms-Universität, Bonn, Germany) for research.^56^ All cell lines were split at least twice a week and kept at 37 °C and 5% CO_2_ in a humidified atmosphere (standard conditions). For detaching or splitting, the cells were washed with Dulbecco's phosphate-buffered saline (DPBS, Lonza) twice, detached with trypsin/EDTA (Lonza), and resuspended in fresh medium according to further requirements.

### High-Throughput Screening Ca^2+^ Flux Assay

For a high-throughput screening, the Ca^2+^ flux assay setup was performed in a 384-well format using Panoptic (WaveFront Biosciences) with stably transfected HEK293-hCMKLR1b-eYFP-Gα_Δ6qi4myr_ cells. The chimeric G protein redirects the endogenous G_i_-coupled pathway to a Gq pathway and activates the phospholipase C instead of the adenylate cyclase. Gα_Δ6qi4myr_ was kindly provided by E. Kostenis (Rheinische Friedrich-Wilhelms-Universität) Bonn, Germany.^56^ Here, 10,000 cells/well were seeded into a BioCoat amine, black-wall, clear-bottom 384-well plate (Corning) and incubated overnight at standard conditions. On the next day, cells were loaded with a Ca^2+^ dye Fluo8-AM (AAT Bioquest, 2.5 μg/mL, final 0.05 μg/well) and Pluronic-F127 (0.3% (v/v), final 0.02%) diluted in assay buffer (HBSS (Lonza), 20 mM HEPES (Sigma-Aldrich), and 2.5 mM probenecid (Sigma-Aldrich), 0.00125% BSA, pH 7.4) for 1 h. The BSA prevents the small peptide from sticking to the wells. Plates were placed into the plate reader. After 10 s of baseline recording, the respective compound (∼10 μM) was added. Two minutes afterward, chemerin-9 (1 nM) was added. Data were collected with 1 Hz and with an excitation of 477 nm and an emission at 536 nm with a 40 nm bandwidth. Eleven hits out of 9280 tested compounds showed receptor modulation. The activity of two compounds with high similarity was reproduced in a 96-well plate assay setup described in the Ca^2+^ Flux Assay section.

### Ca^2+^ Flux Assay

For detecting the released Ca^2+^ concentration, a Ca^2+^ flux G protein activation assay was applied with stably transfected HEK293-hCMKLR1b-eYFP-Gα_Δ6qi4myr_ cells (100,000 cells per well), which were seeded into a poly-d-lysine (0.001% in DPBS, v/v) coated black, transparent-bottom 96-well plate and incubated overnight at standard conditions. For receptor variant investigations, empty HEK293 cells were cultivated to 80% confluence in a T25 cell culture flask and transfected with 3000 ng of wild-type CMKLR1 or the variant plasmid in addition to 1000 ng of chimeric G protein Gα_Δ6qi4myr_ overnight using Metafectene Pro (Biontex) according to the manufacturer’s protocol. On the following day, the cell culture medium was aspirated and the cells were incubated at 37 °C in 50 μL/well assay buffer (HBSS (Lonza), 20 mM HEPES (Sigma-Aldrich), 2.5 mM probenecid (Sigma-Aldrich, pH 7.4) containing Fluo2-AM (Abcam, 0.3% (v/v) of 2.5 μg/mL, final 0.125 μg/well), and Pluronic-F127 (final 0.06% (v/v)) for 1 h. For one-addition assays, the buffer was removed and replaced with 100 μL/well assay buffer, and for two-addition assays with 50 μL/well. The cell plate was set into a FlexStation III device (Molecular Devices, λ_ex_ = 485 nm, λ_em_ = 525 nm). The basal Ca^2+^ level was recorded for 20 s, followed by the addition of 50 μL of a 2-fold concentrated compound or DMSO (negative control) and 20 μL of 6-fold concentrated peptide solution, or only 20 μL of peptide solution for the one-addition assay. Ca^2+^ signal responses were analyzed as the resulting maximum *x*-fold over basal and normalized to the maximum response after the chemerin-9 (C9) addition. All experiments were performed in duplicates, and each experiment was repeated at least three times. The results were analyzed by using log­(agonist) vs response (three parameters) calculation in Prism version 5.03. For submaximal Ca^2+^ experiments, compound or DMSO was added as mentioned above and followed by an EC_80/50/20_ of C9 (15, 3, and 0.5 nM) or EC_80_ values of C9 appropriate to the CMKLR1 variant. The results were examined by log­(inhibitor) vs response (three parameters).

### BRET-Based Arrestin-3 Recruitment

By using a bioluminescence resonance energy transfer (BRET) technique, the arrestin-3 (arr-3) recruitment to the CMKLR1 or GPR1 was investigated. HEK293 cells grown to 80% confluence in a T75 cell flask were transient transfected with 300 ng of Nluc-arr-3-pcDNA3.1 (bioluminescence signal) fusion proteins and 11,700 ng of CMKLR1-eYFP-pVitro2 or GPR1-eYFP-pVitro2 (fluorescence signal), thus in a ratio of 1:40, using Metafectene Pro (Biontex) according to the manufacturer’s protocol. One day after transfection, 75,000 cells/well were seeded into a white, solid 96-well plate using a phenol red-free culture medium. Two days after transfection, the assay was carried out in assay buffer (HBSS (Sigma) and 25 mM HEPES (Sigma-Aldrich), pH 7.3) and at 37 °C. First, the medium was removed and replaced by 80 μL of 1.25-fold concentrated compound solution or DMSO (negative control, w/o compounds); then, 10 μL of a serial dilution of a 10-fold concentrated peptide was added to the cells as well, and 10 μL of coelenterazine h (concentration 2.4 mM, final 2.64 μM). For submaximal analysis, different compound concentrations were investigated while a constant EC_80/50/20_ of C9 (300, 250, and 40 nM) was applied to the cells. Concentration–response curves were measured 15 min after coelenterazine h addition in a microplate reader (Tecan Spark). The BRET ratio was calculated by dividing the fluorescence signal (λ_em_ = 400–440 nm) by bioluminescence (λ_em_ = 505–590 nm), and netBRET signals were determined by subtracting the ratio of unstimulated controls. Data analysis was performed with GraphPad Prism 5.03 containing data from at least two independent experiments as triplicates. The concentration responses were fitted to log­(agonist/inhibitor) vs response (three parameters).

### Displacement BRET

To observe the displacement in the binding pocket of C9 at CMKLR1 or GPR1 with the compound, HEK293 cells were transfected with 3000 ng of Nluc-CMKLR1-eYFP-pVitro2 or Nluc-GPR1-eYFP-pVitro2 in a T75 (80% confluence). After overnight incubation at standard conditions, 75,000 cells/well were seeded into a black, solid 96-well plate using phenol red-free culture medium. On the next day, 80 μL of a 1.25-fold concentrated compound solution, DMSO (negative control, w/o compounds), or peptide or chemerinS157 (positive control) was added to the cells. Then, 10 μL of a 10-fold concentrated, constant concentration of [K^141^-TAMRA]-ChemS157 (final 40 nM) or TAMRA-C9 (final 10 nM) was added to the cells.^16^ To reach equilibrium, cells were incubated on top of a rotating platform on ice in the dark for 1 h, followed by adding 10 μL of coelenterazine h (concentration 2.4 mM, final 8.04 μM). The readout was performed directly in a microplate reader (Tecan Spark) with a filter for fluorescence (λ_em_ = 550–700 nm) and bioluminescence (λ_em_ = 430–470 nm). Data studies were performed as mentioned for arr-3 BRET.

### Live-Cell Microscopy

Live-cell imaging was recorded using an inverted light microscope equipped with an apotome (Carl Zeiss Microscopy), a 63× oil objective, and Zen 2 blue edition software version 2.0. In poly-d-lysine-coated eight-chamber coverslips (μ-slides Ibidi), HEK293 cells (150,000 cells/200 μL) were seeded and grown overnight at 37 °C (standard conditions). On the next day, cells were transfected at 80% confluence with 1000 ng of CMKLR1-eYFP variants in Opti-MEM Lipofectamine 2000 (Invitrogen) according to the manufacturer’s protocol. One day after transfection, cells were starved in Opti-MEM and nuclei were labeled with Hoechst 33342 (final 0.5 μg/well) for 30 min at standard conditions. The receptor localization was determined by recording the fluorescence of YFP (λ_ex_ = 500/20 nm, λ_em_ = 535/40 nm) with an exposure time of 3000 ms and nuclei localization with Hoechst 33342 (λ_ex_ = 365 nm, λ_em_ = 420 nm, and exposure time = 30 ms).

### Receptor-Mediated Peptide Internalization: A High-Content Imaging System

Receptor-mediated peptide uptake was determined by observing the accumulation of TAMRA fluorescence within cells. For this purpose, HEK293-hCMKLR1b-eYFP-Gα_Δ6qi4myr_ were seeded into black, clear-bottom, 96-well plates (100,000 cells/well) coated before with poly-d-lysine and grown overnight at standard conditions. Prior to stimulation, 30 min of incubation with Hoechst 33342 (0.5 mg/mL, final 0.25 μg/well) in Opti-MEM (Gibco) labeled the nuclei and starved the cells. The solution was removed, and CMKLR1 was stimulated with a 2-fold concentrated compound solution or DMSO as a negative control, continued by the addition of 2-fold concentrated TAMRA-(EG)_4_-C9 peptide serial dilution. After 1 h of incubation at standard conditions, the extracellular TAMRA background was removed by several washing steps with acidic wash (50 mM glycine and 100 mM NaCl, pH 3.0), Hank’s balanced salt solution (HBSS, Lonza), and Opti-MEM. An ImageXpress microconfocal high-content imaging system (HCI, Molecular Devices) detected a microscopic picture for each condition with a filter setup as described previously.^15^ The granularity module processed the intracellular internalized TAMRA-labeled peptide with the following settings for nuclei: 5–30 μm in diameter and 10 gray levels above the background; for granules by TAMRA-peptide fluorescence: 2–5 μm in diameter and 100 gray levels above the background. The average fluorescence intensity per cell was plotted against the logarithm of TAMRA-peptide concentration using GraphPad Prism 5.03. Data were derived from at least four independent experiments performed as duplicates.

### Mutagenesis of Receptors

The receptor construct hCMKLR1b-eYFP-Gα_Δ6qi4myr_-pVitro2 has been described before^18,57^ and was modified to hCMKLR1b-eYFP-pVitro2. According to the manufacturer’s protocol, single amino acid exchanges were created for site-directed mutagenesis experiments into the hCMKLR1b-eYFP-pVitro2 plasmid using a Q5 site-directed mutagenesis kit (New England Biolabs). Primers were designed with NEBaseChanger. All constructs were generally cloned by a polymerase chain reaction (PCR) with a hot start high-fidelity DNA polymerase. After PCR, kinase, ligase, and *Dpn*I enzymes were added for KLD reaction (1 h, 22 °C) to phosphorylate linear plasmids, ligate them, and digest the template. The identity was evaluated using Sanger sequencing performed by Microsynth SeqLab GmbH. The receptor positions are named based on the Ballesteros–Weinstein nomenclature.^53^

### Molecular Modeling of the Inactive Structure of CMKLR1 and GPR1 and Docking of Compound **16**

#### Molecular Modeling of Inactive Structures in AlphaFold2

Models of the inactive conformation of both CMKLR1 and GPR1 were generated by using the adapted version of AlphaFold2 proposed by Heo and Feig.^58^ This multistate GPCR modeling version of AlphaFold uses state-specific templates to bias the selection.^58^ The models were taken from the GPCRdb (the model was generated on 16 August 2022)^59^ and cut to the transmembrane area, including helix 8.^60^ Using the Rosetta relax protocol within Rosetta3 (version 3.13), the models underwent energetic minimization.^61^ Then, 500 structures were created for each receptor, the 50 lowest-scoring ones were chosen and grouped, and their structures were visualized using PyMOL (version 2.5.4). Three different models were selected per structure.

#### Docking of Compound **16** with RosettaLigand

To explore the binding modes of the initial hit and its derivatives, especially compound **16**, a range of docking algorithms was employed. First, RosettaLigand was chosen for its flexibility in accommodating both side-chain and ligand movement.^49,50^ Using the BioChemical Library (version 4.3.0), we generated 100 conformers for each compound.^62,63^ The initial placement of compound **16** within the orthosteric binding sites of CMKLR1 and GPR1 was influenced by its antagonist behavior and preliminary mutagenesis studies. Each docking round involved the creation of 1000 decoys with the interface_delta_X, representing the predicted binding energy between the ligand and receptor, serving as the primary evaluation metric. The docked models were assessed and ranked according to their binding energy, as calculated by Rosetta, and their proximity to key residues, which was highlighted in mutagenesis research. The compounds were grouped based on root-mean-square deviation (RMSD). The most promising docking positions underwent further refinement, where the docking settings were fine-tuned to allow for slight modifications in binding orientations. To enhance the selection process, an experimental filter was applied to the docking models. This filter prioritized ligand poses that interact with residues proven critical for antagonistic activity, as indicated by mutagenesis data. For each receptor, three refinement rounds were conducted, until a final binding pose was determined and validated by mutagenesis data. The final binding pose was evaluated based on the per residue contribution to the total docking score (energy breakdown). Through this comprehensive approach, a definitive binding pose was identified.

#### Docking of Compound **16** with DiffDock and DynamicBind and Refinement with RosettaLigand

To investigate the predicted binding pose in more detail, we employed both DiffDock^51^ and DynamicBind,^52^ cutting-edge generative learning algorithms designed for small-molecule docking. DiffDock employs a novel approach to molecular docking by treating it as a generative modeling problem, rather than the traditional physics-based methods. It starts by generating an initial seed conformation of the ligand using RDKit (open-source cheminformatics software),^64^ which serves as the starting point for the docking process. The core of DiffDock is a diffusion generative model that operates over the non-Euclidean manifold of ligand poses, encompassing translational, rotational, and torsional degrees of freedom. This model iteratively refines the ligand pose through a reverse diffusion process, where the ligand’s translations, rotations, and torsion angles are progressively adjusted toward a likely bound state. DiffDock samples diverse ligand poses and ranks them accordingly.^51^ The respective protein structure and the SMILES code of ligands were used to predict the binding pose with small-molecule conformation. Subsequently, we applied side-chain refinement within RosettaLigand to the results with the highest confidence level. As the results lacked good binding properties, an additional refinement of the receptor structure with a bound ligand with the relax application of Rosetta was conducted. The top-scoring refined receptor was used in an iterative DiffDock process, resulting in a higher confidence level and more realistic binding poses.

At its core, DynamicBind uses a similar deep equivariant geometric diffusion network like DiffDock. However, this algorithm facilitates the efficient transition between different protein equilibrium states by dynamically adjusting the protein conformation from its initial state to a conformation more akin to a ligand-bound state. This capacity allows it to handle substantial protein conformational changes effectively. During its operation, DynamicBind accepts input structures in the form of apo-like conformations for proteins and various formats for ligands. It performs iterative prediction steps, involving gradual translations and rotations of the ligand and concurrent adjustments of the protein residues and side-chain chi angles. Throughout this process, the model simultaneously predicts updates for the ligand and protein, leveraging a scoring module (contact-LDDT) to select the most suitable complex structure from its outputs.^52^ The respective UniProt accession code for generating the protein structure and the SMILES code of ligands were inputted into the algorithm. Again, we applied side-chain refinement within RosettaLigand.

The most promising structures from each docking approach were meticulously selected for further investigation. An energetic analysis of the interactions between the small molecule and individual residues was conducted, comparing these findings to experimental data.

## Supplementary Material









## Notes

Behavior of fragments, which build up compound **16**; Ca^2+^ flux assay and receptor localization of CMKLR1 variants not expressed in the cell membrane; comparison of modeled inactive conformation of CMKLR1 to experimentally determined active conformation; NMR spectra of all synthesized compounds; HPLC traces of *in vitro* tested compounds (PDF)
Molecular formula strings (CSV)
PDB file for CMKLR1 (PDB)
PDB file for GPR1 (PDB)

## §

Present address: Biohaven, 2100 Wharton Street, Suite 615, Pittsburgh, Pennsylvania 15203, United States

## Abbreviations

α-NETA: 2-(α-naphthoyl)-ethyl-trimethylammonium iodide
arr-3: arrestin-3
C9: chemerin-9
BRET: bioluminescence resonance energy transfer
CCRL2: CC-motif chemokine receptor-like 2
C terminus: carboxyl terminus
ChemS157: chemerinS157
CMKLR1: chemokine-like receptor 1
EG: ethylene glycol
GPR1: G protein-coupled receptor 1
Nluc: Nanoluciferase
N terminus: amino terminus
RMSD: root-mean-square deviation
Rarres2: retinoic acid receptor responder 2 gene
TAMRA: 5-carboxytetramethylrhodamine
YFP: yellow fluorescent protein

## References

[1] Sriram K., Insel P. A. (2018). G Protein-coupled receptors as targets for approved
drugs: how many targets and how many drugs?. Mol. Pharmacol..

[2] Hauser A. S., Attwood M. M., Rask-Andersen M., Schiöth H. B., Gloriam D. E. (2017). Trends in GPCR drug discovery: new agents, targets
and indications. Nat. Rev. Drug Discovery.

[3] Lipinski C. A., Lombardo F., Dominy B. W., Feeney P. J. (1997). Experimental and
computational approaches to estimate solubility and permeability in
drug discovery and development settings. Adv.
Drug Delivery Rev..

[4] La
Manna S., Di Natale C., Florio D., Marasco D. (2018). Peptides as
therapeutic agents for inflammatory-related diseases. Int. J. Mol. Sci..

[5] Lewis, R. J. ; Vetter, I. ; Cardoso, F. C. ; Inserra, M. ; King, G. Does nature do ion channel drug discovery better than us? In Ion channel drug discovery, edited by Cox, B. ; Gosling, M. The Royal Society of Chemistry. 2014, 297–319.

[6] Kennedy A.
J., Davenport A. P. (2018). International
union of basic and clinical pharmacology
CIII: chemerin Receptors CMKLR1 (Chemerin1) and GPR1 (Chemerin2) nomenclature,
pharmacology, and function. Pharmacol Rev..

[7] Zabel B. A., Allen S. J., Kulig P., Allen J. A., Cichy J., Handel T. M., Butcher E. C. (2005). Chemerin
activation by serine proteases
of the coagulation, fibrinolytic, and inflammatory cascades. J. Biol. Chem..

[8] Goralski K. B., McCarthy T. C., Hanniman E. A., Zabel B. A., Butcher E. C., Parlee S. D., Muruganandan S., Sinal C. J. (2007). Chemerin, a novel
adipokine that regulates adipogenesis and adipocyte metabolism. J. Biol. Chem..

[9] Wittamer V., Franssen J.-D., Vulcano M., Mirjolet J.-F., Le Poul E., Migeotte I., Brézillon S., Tyldesley R., Blanpain C., Detheux M., Mantovani A., Sozzani S., Vassart G., Parmentier M., Communi D. (2003). Specific recruitment of antigen-presenting cells by
chemerin, a novel processed ligand from human inflammatory fluids. J. Exp. Med..

[10] Zabel B. A., Silverio A. M., Butcher E. C. (2005). Chemokine-like receptor 1 expression
and chemerin-directed chemotaxis distinguish plasmacytoid from myeloid
dendritic cells in human blood. J. Immunol..

[11] Bozaoglu K., Bolton K., McMillan J., Zimmet P., Jowett J. B. M., Collier G., Walder K., Segal D. (2007). Chemerin is a novel
adipokine associated with obesity and metabolic syndrome. Endocrinology..

[12] Chang S.-S., Eisenberg D., Zhao L., Adams C., Leib R., Morser J., Leung L. L. K. (2016). Chemerin activation in human obesity. Obesity (Silver Spring)..

[13] Treeck O., Buechler C. (2020). Chemerin Signaling
in Cancer. Cancers (Basel)..

[14] Wittamer V., Grégoire F., Robberecht P., Vassart G., Communi D., Parmentier M. (2004). The C-terminal nonapeptide of mature chemerin activates
the chemerin receptor with low nanomolar potency. J. Biol. Chem..

[15] Fischer T. F., Czerniak A. S., Weiß T., Zellmann T., Zielke L., Els-Heindl S., Beck-Sickinger A. G. (2021). Cyclic derivatives of the chemerin
C-Terminus as metabolically stable agonists at the chemokine-like
receptor 1 for cancer treatment. Cancers (Basel)..

[16] Czerniak A. S., Kretschmer K., Weiß T., Beck-Sickinger A. G. (2022). The chemerin
receptor CMKLR1 requires full-length chemerin for high affinity in
contrast to GPR1 as demonstrated by a new nanoluciferase-based binding
assay. ChemMedChem.

[17] Wang J., Chen G., Liao Q., Lyu W., Liu A., Zhu L., Du Y., Ye R. D. (2023). Cryo-EM structure of the human chemerin
receptor 1-Gi protein complex bound to the C-terminal nonapeptide
of chemerin. Proc. Natl. Acad. Sci. U.S.A..

[18] Zhang X., Weiß T., Cheng M. H., Chen S., Ambrosius C. K., Czerniak A. S., Li K., Feng M., Bahar I., Beck-Sickinger A. G., Zhang C. (2023). Structural basis of
G protein-coupled
receptor CMKLR1 activation and signaling induced by a chemerin-derived
agonist. PLoS Biol..

[19] Graham K. L., Zhang J. V., Lewén S., Burke T. M., Dang T., Zoudilova M., Sobel R. A., Butcher E. C., Zabel B. A. (2014). A novel
CMKLR1 small molecule antagonist suppresses CNS autoimmune inflammatory
disease. PLoS One.

[20] Yu M., Yang Y., Zhao H., Li M., Chen J., Wang B.-B., Xiao T., Huang C., Zhao H., Zhou W., Zhang J. V. (2022). Targeting the chemerin/CMKLR1
axis
by small molecule antagonist α-NETA mitigates endometriosis
progression. Front. Pharmacol..

[21] Zheng C., Zheng Y., Chen X., Zhong X., Zheng X., Yang S., Zheng Z. (2023). α-NETA
down-regulates CMKLR1
mRNA expression in ileum and prevents body weight gains collaborating
with ERK inhibitor PD98059 in turn to alleviate hepatic steatosis
in HFD-induced obese mice but no impact on ileal mucosal integrity
and steatohepatitis progression. BMC Endocr.
Disord..

[22] Kumar J. D., Holmberg C., Kandola S., Steele I., Hegyi P., Tiszlavicz L., Jenkins R., Beynon R. J., Peeney D., Giger O. T., Alqahtani A., Wang T. C., Charvat T. T., Penfold M., Dockray G. J., Varro A. (2014). Increased expression
of chemerin in squamous esophageal cancer myofibroblasts and role
in recruitment of mesenchymal stromal cells. PLoS One.

[23] Kumar J. D., Kandola S., Tiszlavicz L., Reisz Z., Dockray G. J., Varro A. (2016). The role of chemerin
and ChemR23 in stimulating the invasion of squamous
oesophageal cancer cells. Br. J. Cancer..

[24] Kennedy A. J., Yang P., Read C., Kuc R. E., Yang L., Taylor E. J. A., Taylor C. W., Maguire J. J., Davenport A. P. (2016). Chemerin
elicits potent constrictor actions via chemokine-like receptor 1 (CMKLR1),
not G-protein-coupled receptor 1 (GPR1), in human and rat vasculature. J. Am. Heart Assoc..

[25] Neves K. B., Nguyen Dinh Cat A., Alves-Lopes R., Harvey K. Y., Da Costa R. M., Lobato N. S., Montezano A. C., de Oliveira A. M., Touyz R. M., Tostes R. C. (2018). Chemerin
receptor blockade improves
vascular function in diabetic obese mice via redox-sensitive and Akt-dependent
pathways. Am. J. Physiol.: Heart Circ. Physiol..

[26] Macvanin M. T., Rizzo M., Radovanovic J., Sonmez A., Paneni F., Isenovic E. R. (2022). Role of Chemerin
in Cardiovascular Diseases. Biomedicines..

[27] Imaizumi T., Kobayashi A., Otsubo S., Komai M., Magara M., Otsubo N. (2019). The discovery
and optimization of a series of 2-aminobenzoxazole
derivatives as ChemR23 inhibitors. Bioorg. Med.
Chem..

[28] Imaizumi T., Otsubo S., Komai M., Takada H., Maemoto M., Kobayashi A., Otsubo N. (2020). The design, synthesis and evaluation
of 2-aminobenzoxazole analogues as potent and orally efficacious ChemR23
inhibitors. Bioorg. Med. Chem..

[29] Imaizumi T., Otsubo S., Maemoto M., Kobayashi A., Komai M., Takada H., Sakaida Y., Otsubo N. (2022). Discovery
and mechanistic study of thiazole-4-acylsulfonamide derivatives as
potent and orally active ChemR23 inhibitors with a long-acting effect
in cynomolgus monkeys. Bioorg. Med. Chem..

[30] Ko B., Jang Y., Kwak S.-H., You H., Kim J.-H., Lee J.-E., Park H. D., Kim S.-K., Goddard W. A., Han J. H., Kim Y.-C. (2023). Discovery of 3-phenyl
indazole-based
novel chemokine-like receptor 1 antagonists for the treatment of psoriasis. J. Med. Chem..

[31] Baell J. B., Holloway G. A. (2010). New substructure filters for removal
of pan assay interference
compounds (PAINS) from screening libraries and for their exclusion
in bioassays. J. Med. Chem..

[32] https://zinc15.docking.org/patterns/home/ (accessed July 4, 2024) 2024.

[33] Sterling T., Irwin J. J. (2015). ZINC 15--Ligand Discovery for Everyone. J. Chem. Inf. Model..

[34] Skjæret, T. ; Fraser, D. A. ; Steineger, H. H. Patent: Romatic compounds and pharmaceutical uses thereof. 2019, BASF AS [NO] WO2020074964A1.

[35] von
Bredow L., Fürll A., Tretbar M. (2024). Development of a Divergent
Synthesis Strategy for 5-Sulfonyl-Substituted Uracil Derivatives. J. Org. Chem..

[36] Wei J., Liang H., Ni C., Sheng R., Hu J. (2019). Transition-metal-free
desulfinative cross-coupling of heteroaryl sulfinates with Grignard
reagents. Org. Lett..

[37] Wang Y., Zhang F., Wang Y., Pan Y. (2022). Electrochemistry enabled
nickel-catalyzed selective C–S bond coupling reaction. Eur. J. Org. Chem..

[38] Schild H. O. (1947). pA, a new
scale for the measurement of drug antagonism. Br. J. Pharmacol. Chemother..

[39] Kenakin T. P. (1982). The Schild
regression in the process of receptor classification. Can. J. Physiol. Pharmacol..

[40] de
Henau O., Degroot G.-N., Imbault V., Robert V., de Poorter C., Mcheik S., Galés C., Parmentier M., Springael J.-Y. (2016). Signaling properties of chemerin
receptors CMKLR1, GPR1 and CCRL2. PLoS One.

[41] Barnea G., Strapps W., Herrada G., Berman Y., Ong J., Kloss B., Axel R., Lee K. J. (2008). The genetic design
of signaling cascades to record receptor activation. PNAS..

[42] Mundell S. J., Orsini M. J., Benovic J. L. (2002). Characterization of arrestin expression
and function. Methods Enzymol..

[43] Degroot G.-N., Lepage V., Parmentier M., Springael J.-Y. (2022). The atypical
chemerin receptor GPR1 displays different modes of interaction with
β-arrestins in humans and mice with important consequences on
subcellular localization and trafficking. Cells.

[44] Fischer T. F., Czerniak A. S., Weiß T., Schoeder C. T., Wolf P., Seitz O., Meiler J., Beck-Sickinger A. G. (2021). Ligand-binding
and -scavenging of the chemerin receptor GPR1. Cell. Mol. Life Sci..

[45] von
Moo E., van Senten J. R., Bräuner-Osborne H., Mo̷ller T. C. (2021). Arrestin-dependent and -independent internalization
of G protein-coupled receptors: Methods, mechanisms, and implications
on cell signaling. Mol. Pharmacol..

[46] Kretschmer K., Zellmann T., Mörl K., Beck-Sickinger A. G. (2023). Stable
binding of full-length chemerin is driven by negative charges in the
CMKLR1 N terminus. ChemBioChem.

[47] https://rowansci.com/tools/tautomers (accessed March 20, 2025) 2025.

[48] Stanovnik, B. ; Tišler, M. ; Katritxky, A. R. ; Denisko, O. V. The Tautomerism of Heterocycles. Six-Membered Heterocycles: Part 1, Annular Tautomerism, In: Adv. Heterocycl. Chem.; Elsevier, Ed.: Katritzky, A. R. 2001, 81, 253–303.

[49] Meiler J., Baker D. (2006). ROSETTALIGAND: protein-small
molecule docking with full side-chain
flexibility. Proteins..

[50] Lemmon G., Meiler J. (2012). Rosetta Ligand docking
with flexible XML protocols. Methods Mol. Biol..

[51] Corso, G. ; Stärk, H. ; Jing, B. ; Barzilay, R. ; Jaakkola, T. DiffDock: Diffusion steps, twists, and turns for molecular docking. In Conference paper at ICLR 2023. 2022, arXiv:2210.01776.

[52] Lu W., Zhang J., Huang W., Zhang Z., Jia X., Wang Z., Shi L., Li C., Wolynes P. G., Zheng S. (2024). DynamicBind: predicting ligand-specific protein-ligand complex structure
with a deep equivariant generative model. Nat.
Commun..

[53] Ballesteros J. A., Weinstein H. (1995). [19] Integrated
methods for the construction of three-dimensional
models and computational probing of structure-function relations in
G protein-coupled receptors. Methods Neurosci..

[54] Schultz S., Saalbach A., Heiker J. T., Meier R., Zellmann T., Simon J. C., Beck-Sickinger A. G. (2013). Proteolytic
activation of prochemerin
by kallikrein 7 breaks an ionic linkage and results in C-terminal
rearrangement. Biochem. J..

[55] Mattern, A. Chemical modification of adipokines: adiponectin and chemerin Dissertation Universität Leipzig 15, 11, 2016

[56] Kostenis E. (2001). Is Gαα16
the optimal tool for fishing ligands of orphan G-protein-coupled receptors?. Trends Pharmacol. Sci..

[57] Schermeng T., Liessmann F., Ambrosius C. K., Meiler J., Beck-Sickinger A. G. (2024). Binding
Mode of Cyclic Chemerin-9 Peptide and ChemerinS157 Protein at CMKLR1. ChemBioChem.

[58] Heo L., Feig M. (2022). Multi-state
modeling of G-protein coupled receptors at experimental
accuracy. Proteins..

[59] https://gpcrdb.org (accessed Feb 23, 2024) 2024.

[60] Pándy-Szekeres G., Caroli J., Mamyrbekov A., Kermani A. A., Keserű G. M., Kooistra A. J., Gloriam D. E. (2023). GPCRdb in 2023: state-specific structure
models using AlphaFold2 and new ligand resources. Nucleic Acids Res..

[61] Leman J. K., Weitzner B. D., Lewis S. M., Adolf-Bryfogle J., Alam N., Alford R. F., Aprahamian M., Baker D., Barlow K. A., Barth P., Basanta B., Bender B. J., Blacklock K., Bonet J., Boyken S. E., Bradley P., Bystroff C., Conway P., Cooper S., Correia B. E., Coventry B., Das R., de Jong R. M., DiMaio F., Dsilva L., Dunbrack R., Ford A. S., Frenz B., Fu D. Y., Geniesse C., Goldschmidt L., Gowthaman R., Gray J. J., Gront D., Guffy S., Horowitz S., Huang P.-S., Huber T., Jacobs T. M., Jeliazkov J. R., Johnson D. K., Kappel K., Karanicolas J., Khakzad H., Khar K. R., Khare S. D., Khatib F., Khramushin A., King I. C., Kleffner R., Koepnick B., Kortemme T., Kuenze G., Kuhlman B., Kuroda D., Labonte J. W., Lai J. K., Lapidoth G., Leaver-Fay A., Lindert S., Linsky T., London N., Lubin J. H., Lyskov S., Maguire J., Malmström L., Marcos E., Marcu O., Marze N. A., Meiler J., Moretti R., Mulligan V. K., Nerli S., Norn C., Ó’Conchúir S., Ollikainen N., Ovchinnikov S., Pacella M. S., Pan X., Park H., Pavlovicz R. E., Pethe M., Pierce B. G., Pilla K. B., Raveh B., Renfrew P. D., Burman S. S. R., Rubenstein A., Sauer M. F., Scheck A., Schief W., Schueler-Furman O., Sedan Y., Sevy A. M., Sgourakis N. G., Shi L., Siegel J. B., Silva D.-A., Smith S., Song Y., Stein A., Szegedy M., Teets F. D., Thyme S. B., Wang R. Y.-R., Watkins A., Zimmerman L., Bonneau R. (2020). Macromolecular modeling and design in Rosetta: recent
methods and frameworks. Nat. methods..

[62] Kothiwale S., Mendenhall J. L., Meiler J. (2015). BCL::Conf: small molecule conformational
sampling using a knowledge based rotamer library. J. Cheminf..

[63] Mendenhall J., Brown B. P., Kothiwale S., Meiler J. (2021). BCL::Conf: Improved
open-source knowledge-based conformation sampling using the crystallography
open database. J. Chem. Inf. Model..

[64] Landrum, G. ; Tosco, P. ; Kelley, B. ; Sriniker; Ric; Gedeck; Vianello, R. ; Schneider, N. ; Dalke, A. ; Dan, N ; Kawashima, E. ; Cole, B. ; Turk, S. ; Swain, M. ; Savelyev, A. ; Cosgrove, D. ; Vaucher, A. ; Wójcikowski, M. ; Jones, G. ; Probst, D. ; Godin, G. ; Scalfani, V. F. ; Pahl, A. ; Berenger, F. ; Lehtivarjo, J. ; strets123; JP; Gavid, D. ; Sforna, G. ; Jensen, J. H. rdkit/rdkit: 2021_03_1 (Q1 2021) Release, DOI:10.5281/zenodo.4639764. 2021.

