# Supplementary material for: Similar Binding Mode of a 5‑Sulfonylthiouracil Derivative Antagonist at Chemerin Receptors CMKLR1 and GPR1
Source: J Med Chem. 2025 May 16;68(11):11149–73. doi: 10.1021/acs.jmedchem.5c00135 (PMC12169613; doi:10.1021/acs.jmedchem.5c00135)
Supplement: Supplementary file 1 [file jm5c00135_si_001.pdf]

Supporting Information

Similar Binding Mode of a 5-Sulfonylthiouracil Derivative Antagonist at Chemerin Receptors CMKLR1 and GPR1

Tina Schermeng[a, ‡], Alexander Fürll[b, ‡], Fabian Liessmann[b,c], Lukas von Bredow[b], Jan Stichel[a], C. David Weaver[d], Maik Tretbar[b], Jens Meiler[b,c,d,*] and Annette G. Beck-Sickinger[a,*]

[a] Institute of Biochemistry, Leipzig University, Leipzig 04103, Germany

[b] Institute for Drug Discovery, Leipzig University, Leipzig 04103, Germany

[c] Center for Scalable Data Analytics and Artificial Intelligence ScaDS.AI and School of Embedded Composite Artificial Intelligence SECAI, Dresden/Leipzig 01069/04105, Germany Department of Chemistry,

[d] Department of Chemistry, Department of Pharmacology and Institute of Chemical Biology, Vanderbilt University, Nashville, Tennessee 37235, United States

[*] corresponding author Email: abeck-sickinger@uni-leipzig.de, jens@meilerlab.org

[‡] These authors contributed equally to this work.

Table of Contents

1. HPLC traces 3

2. NMR spectra 26

Figure S96: Selectivity of VU0514009 at two different class A GPCRs. 122

Figure S97: Behavior of fragments, which build up compound 16 123

Figure S98: Comparison of the inhibitory effect of α-NETA and 16 without pre-incubation. 123

Figure S99: Ca^{2+} flux data and microscopic images of CMKLR1 variants with impaired membrane localization 124

Figure S100: Comparison of modeled inactive conformation of CMKLR1 to experimentally determined active conformation 125

Table S1. Analytical data and sequence of peptides 125

3. Generation of energetical minimized CMKLR1 and GPR1 structures 126

3.1 Sequence for CMKLR1 and GPR1 models 126

3.2 Options for Energy Minimization (500 generated structures in 50 runs): 126
4. Molecular Docking with RosettaLigand 128
4.1 Conformer generation with BCL 128
4.2 Docking with RosettaLigand 128
5. Molecular Docking with DiffDock 131
5.1 Preparing the ranked sdfs for refinement 131
5.2 Refinement with RosettaLigand 131
5.3 Refinement with Rosetta Energy Minimization 132
6. Molecular Docking with DynamicBind 132
7. Energy Breakdown 132
8. Bibliography 134

1. HPLC traces

The purity of all compounds (**1-43**) to be > 95% was determined prior to biological testing by HPLC analysis using RP-HPLC Thermo Fisher Scientific ULTIMATE 3000 with a Macherey-Nagel 100-5 C18ec column (5 µm, 250 x 4.6 mm), eluted with a linear gradient solvent system (CH_3CN/H_2O).

2-{[5-(3-Chloro-4-methylbenzenesulfonyl)-4-oxo-1,4-dihydropyrimidin-2-yl]sulfanyl}-*N*-(2-methoxy-5-methylphenyl) acetamide (**compound 1, VU0514009**)

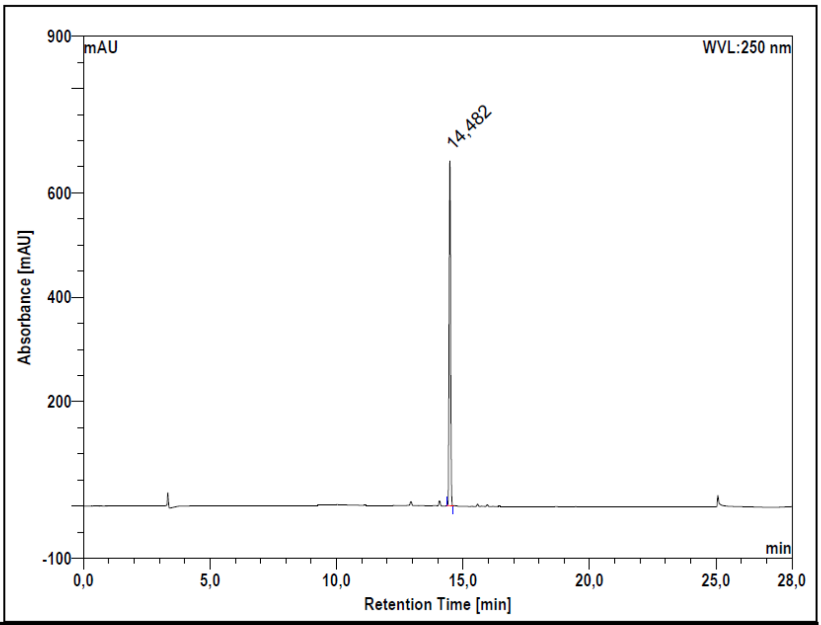

No.	Ret.Time	Peak Name	Height	Area	Rel.Area	Amount	Type
V_VIS_	UV_VIS_1	UV_VIS_1	UV_VIS_1	UV_VIS_1	UV_VIS_1	UV_VIS_1	UV_VIS_1
	min		mAU	mAU*min	%		
1	14,48	n.a.	660,471	45,117	100,00	n.a.	BMB
Total:			660,471	45,117	100,00	0,000	

2-{[5-(3-Chloro-4-methylbenzenesulfonyl)-4-oxo-1,4-dihydropyrimidin-2-yl]sulfanyl}-*N*-(2,5-dimethoxyphenyl)acetamide (**compound 2**)

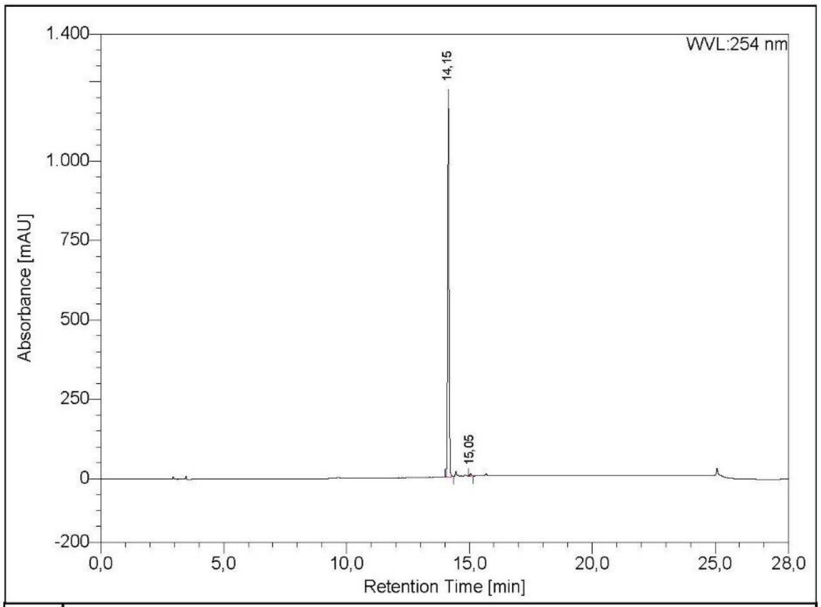

No.	Ret.Time	Peak Name	Height	Area	Rel.Area	Amount	Type
	min		mAU	mAU*min	%		
1	14,15	n.a.	1218,936	83,665	99,31	n.a.	BMB
2	15,05	n.a.	8,238	0,578	0,69	n.a.	BMB*
Total:			1227,174	84,243	100,00	0,000	

2-{[5-(3-Chloro-4-methylbenzenesulfonyl)-4-oxo-1,4-dihydropyrimidin-2-yl]sulfanyl}-*N*-(5-fluoro-2-methylphenyl)acetamide (**compound 3**)

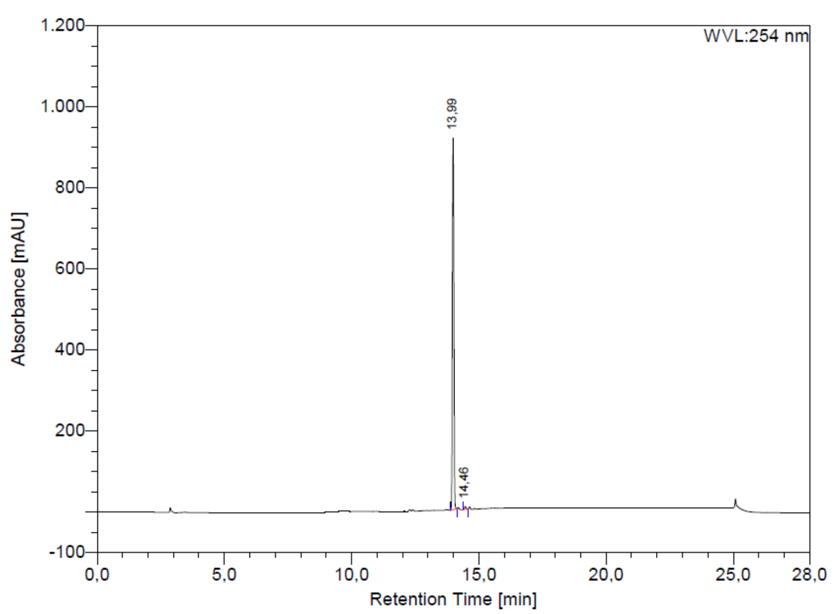

No.	Ret.Time min		Peak Name	Height mAU	Area mAU*min	Rel.Area %	Amount	Type
1	13,99	n.a.		916,812	62,018	99,19	n.a.	BMB
2	14,46	n.a.		6,960	0,504	0,81	n.a.	BMB
Total:				923,772	62,523	100,00	0,000	

2-{[5-(3-Chloro-4-methylbenzenesulfonyl)-4-oxo-1,4-dihydropyrimidin-2-yl]sulfanyl}-*N*-(2-methoxyphenyl)acetamide (**compound 4**)

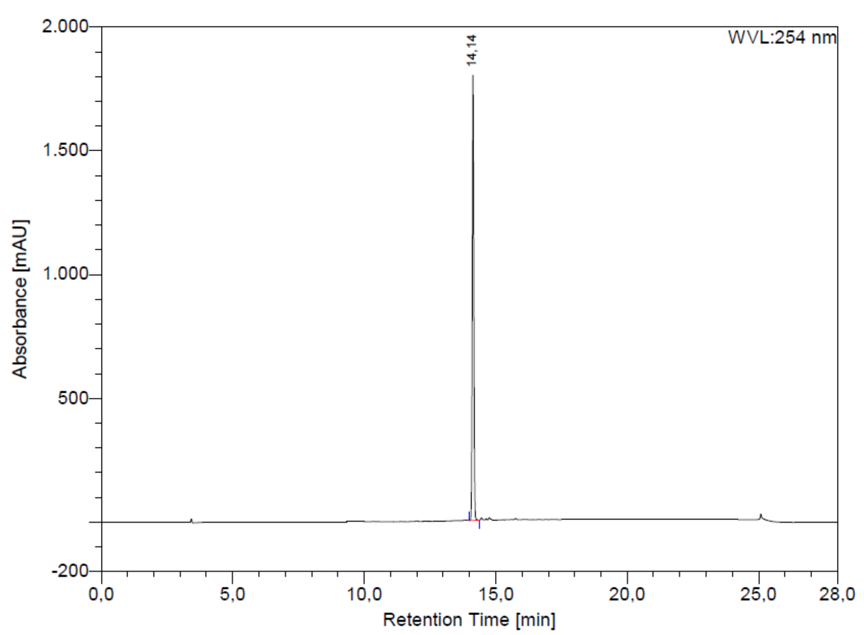

No.	Ret.Time min		Peak Name	Height mAU	Area mAU*min	Rel.Area %	Amount	Type
1	14,14	n.a.		1797,679	124,201	100,00	n.a.	BMB
Total:				1797,679	124,201	100,00	0,000	

2-{[5-(3-Chloro-4-methylbenzenesulfonyl)-4-oxo-1,4-dihydropyrimidin-2-yl]sulfanyl}-*N*-(4-methoxyphenyl)acetamide (**compound 5**)

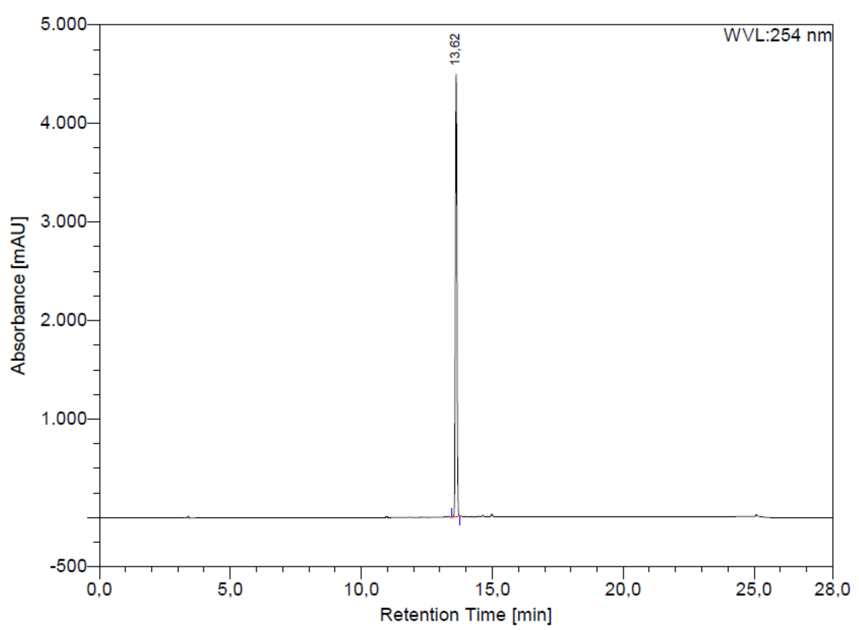

No.	Ret.Time min	Peak Name	Height mAU	Area mAU*min	Rel.Area %	Amount	Type
1	13,62	n.a.	4487,954	314,541	100,00	n.a.	BMB
Total:			4487,954	314,541	100,00	0,000	

2-{[5-(3-Chloro-4-methylbenzenesulfonyl)-4-oxo-1,4-dihydropyrimidin-2-yl]sulfanyl}-*N*-(3-methylphenyl)acetamide (**compound 6**)

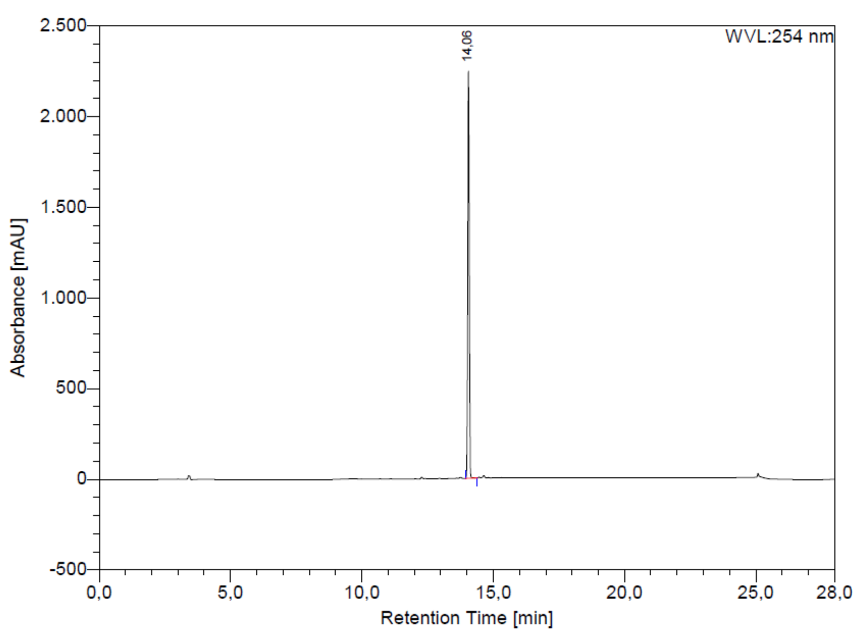

No.	Ret.Time min	Peak Name	Height mAU	Area mAU*min	Rel.Area %	Amount	Type
1	14,06	n.a.	2245,388	154,617	100,00	n.a.	BMB
Total:			2245,388	154,617	100,00	0,000	

2-{[5-(3-Chloro-4-methylbenzenesulfonyl)-4-oxo-1,4-dihydropyrimidin-2-yl]sulfanyl}-*N*-[2-(propan-2-yl)phenyl]acetamide (**compound 7**)

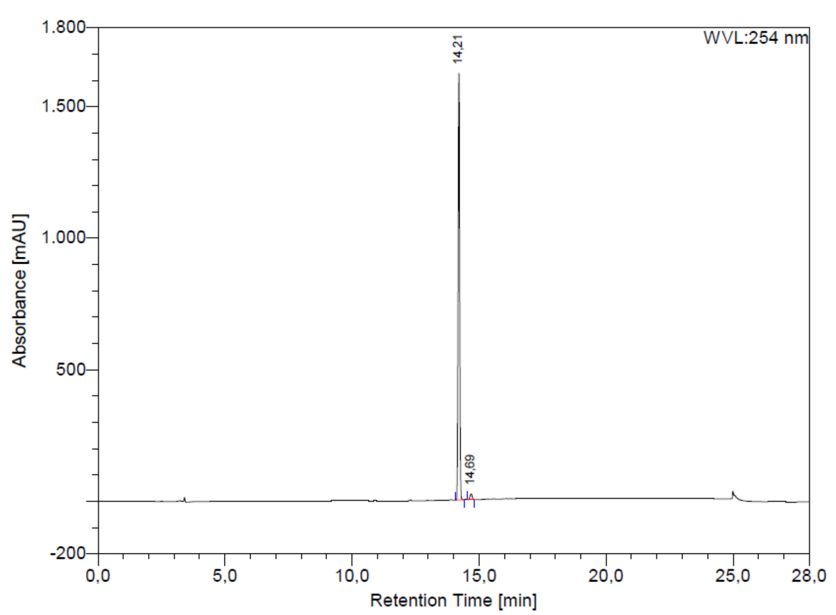

No.	Ret.Time min	Peak Name	Height mAU	Area mAU*min	Rel.Area %	Amount	Type
1	14,21	n.a.	1621,345	115,002	98,18	n.a.	BMB
2	14,69	n.a.	21,862	2,132	1,82	n.a.	BMB
Total:			1643,206	117,135	100,00	0,000	

2-{[5-(3-Chloro-4-methylbenzenesulfonyl)-4-oxo-1,4-dihydropyrimidin-2-yl]sulfanyl}-*N*-[3-(propan-2-yl)phenyl]acetamide (**compound 8**)

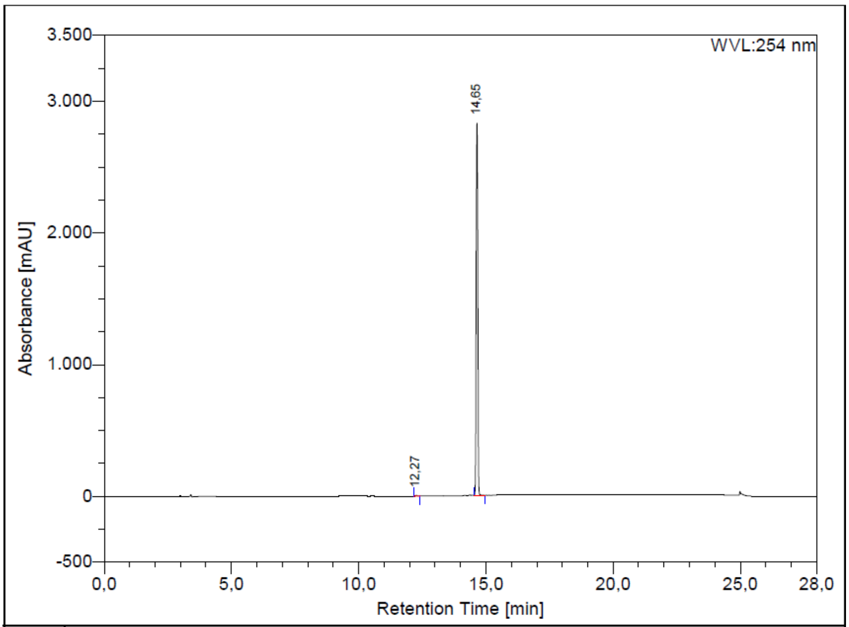

No.	Ret.Time min	Peak Name	Height mAU	Area mAU*min	Rel.Area %	Amount	Type
1	12,27	n.a.	6,717	0,504	0,24	n.a.	BMB
2	14,65	n.a.	2824,075	207,043	99,76	n.a.	BMB
Total:			2830,792	207,547	100,00	0,000	

2-{[5-(3-Chloro-4-methylbenzenesulfonyl)-4-oxo-1,4-dihydropyrimidin-2-yl]sulfanyl}-*N*-[4-(propan-2-yl)phenyl]acetamide (**compound 9**)

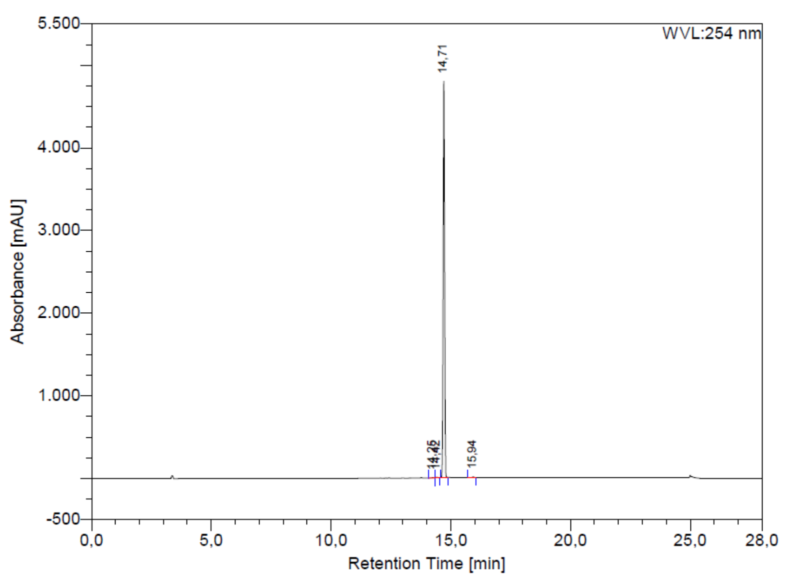

No.	Ret.Time min	Peak Name	Height mAU	Area mAU*min	Rel.Area %	Amount	Type
1	14,25	n.a.	5,971	0,507	0,14	n.a.	BMB
2	14,42	n.a.	8,823	0,680	0,19	n.a.	BMB
3	14,71	n.a.	4794,022	351,934	99,44	n.a.	BMB
4	15,94	n.a.	9,575	0,809	0,23	n.a.	BMB
Total:			4818,392	353,930	100,00	0,000	

N-{[1,1'-Biphenyl]-4-yl}-2-{[5-(3-chloro-4-methylbenzenesulfonyl)-4-oxo-1,4-dihydro-pyrimidin-2-yl]sulfanyl}acetamide (**compound 10**)

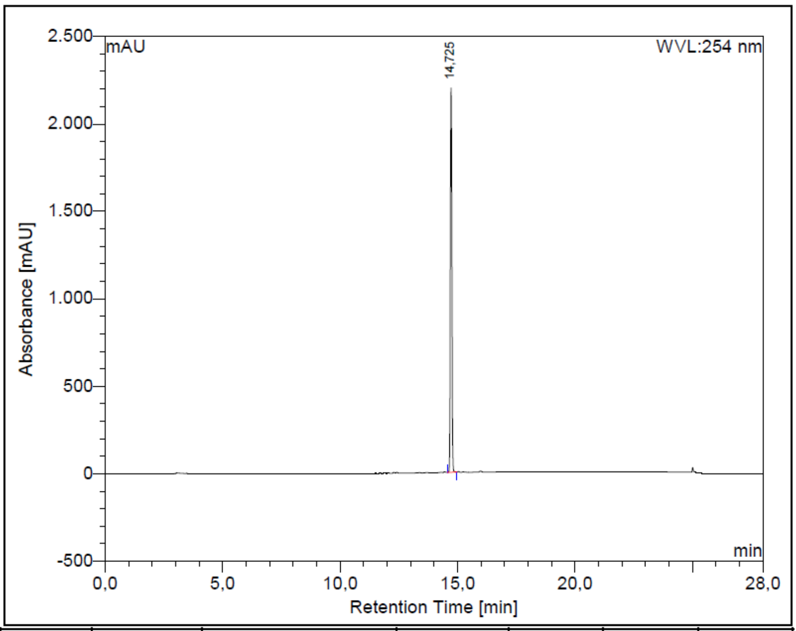

No.	Ret.Time	Height	Area	Rel.Area	Amount	Type
UV_VIS_1	UV_VIS_1	UV_VIS_1	UV_VIS_1	UV_VIS_1	UV_VIS_1	UV_VIS_1
	min	mAU	mAU*min	%		
1	14,73	2196,570	165,137	100,00	n.a.	BMB
Total:			2196,570	165,137	100,00	0,000

2-{[5-(3-Chloro-4-methylbenzenesulfonyl)-4-oxo-1,4-dihydropyrimidin-2-yl]sulfanyl}-*N*-(2-fluorophenyl)acetamide (**compound 11**)

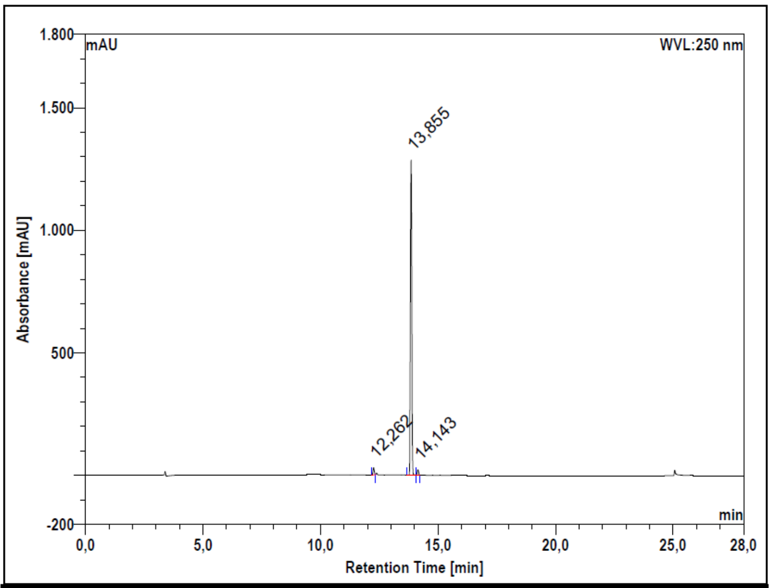

No. V_VIS	Ret.Time UV_VIS_1 min	Peak Name UV_VIS_1	Height UV_VIS_1 mAU	Area UV_VIS_1 mAU*min	Rel.Area UV_VIS_1 %	Amount UV_VIS_1	Type UV_VIS_1
1	12,26	n.a.	29,622	1,912	2,11	n.a.	BMB*
2	13,86	n.a.	1285,808	87,265	96,18	n.a.	BM
3	14,14	n.a.	22,613	1,552	1,71	n.a.	MB
Total:			1338,043	90,728	100,00	0,000	

2-{[5-(3-Chloro-4-methylbenzenesulfonyl)-4-oxo-1,4-dihydropyrimidin-2-yl]sulfanyl}-*N*-(3-fluorophenyl)acetamide (**compound 12**)

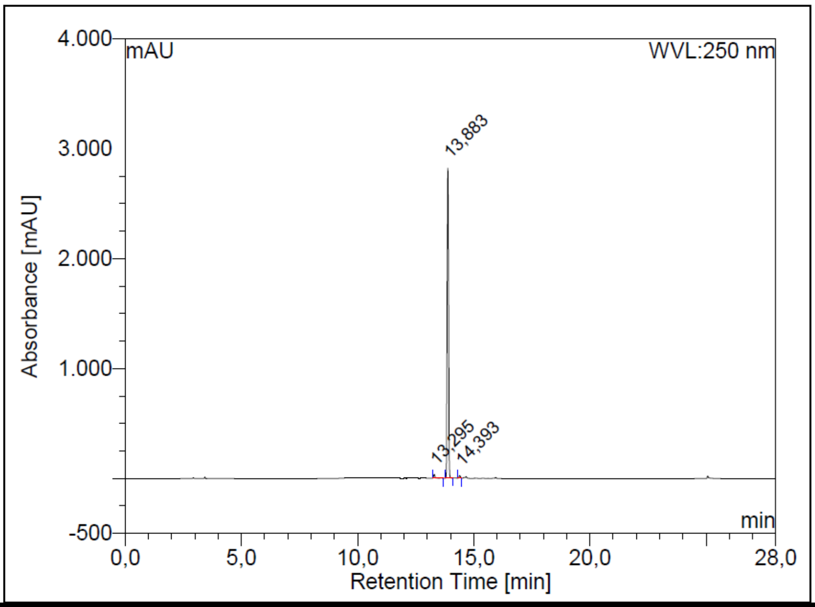

No.	Ret.Time min	Peak Name	Height mAU	Area mAU*min	Rel.Area %	Amount	Type
1	13,30	n.a.	33,724	2,742	1,39	n.a.	BMB*
2	13,88	n.a.	2818,159	192,710	97,78	n.a.	BMB*
3	14,39	n.a.	24,145	1,638	0,83	n.a.	BMB
Total:			2876,027	197,090	100,00	0,000	

2-{[5-(3-Chloro-4-methylbenzenesulfonyl)-4-oxo-1,4-dihydropyrimidin-2-yl]sulfanyl}-*N*-(4-fluorophenyl)acetamide (**compound 13**)

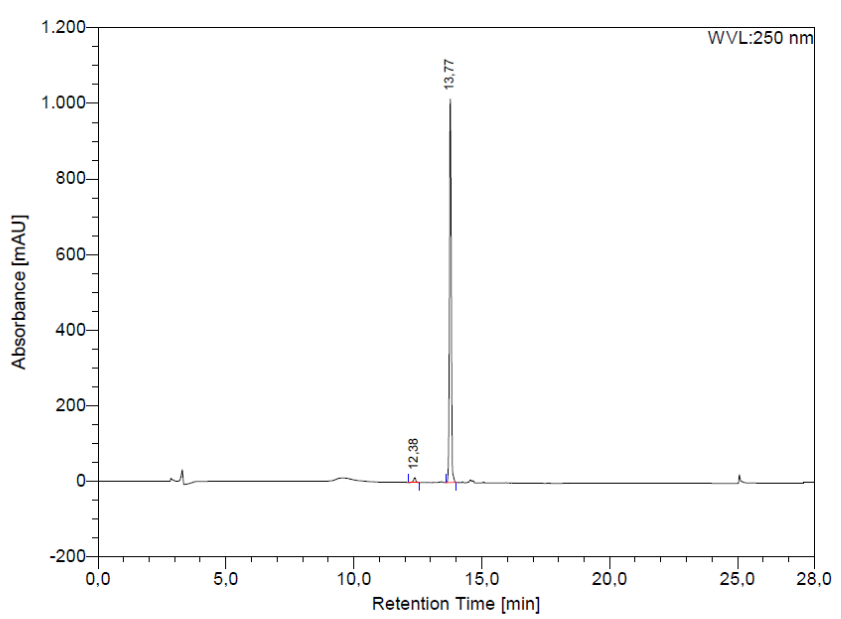

No.	Ret.Time min	Peak Name	Height mAU	Area mAU*min	Rel.Area %	Amount	Type
1	12,38	n.a.	12,694	1,286	1,52	n.a.	BMB
2	13,77	n.a.	1014,155	83,183	98,48	n.a.	BMB
Total:			1026,849	84,469	100,00	0,000	

2-{[5-(3-Chloro-4-methylbenzenesulfonyl)-4-oxo-1,4-dihydropyrimidin-2-yl]sulfanyl}-*N*-(4-nitrophenyl)acetamide (**compound 14**)

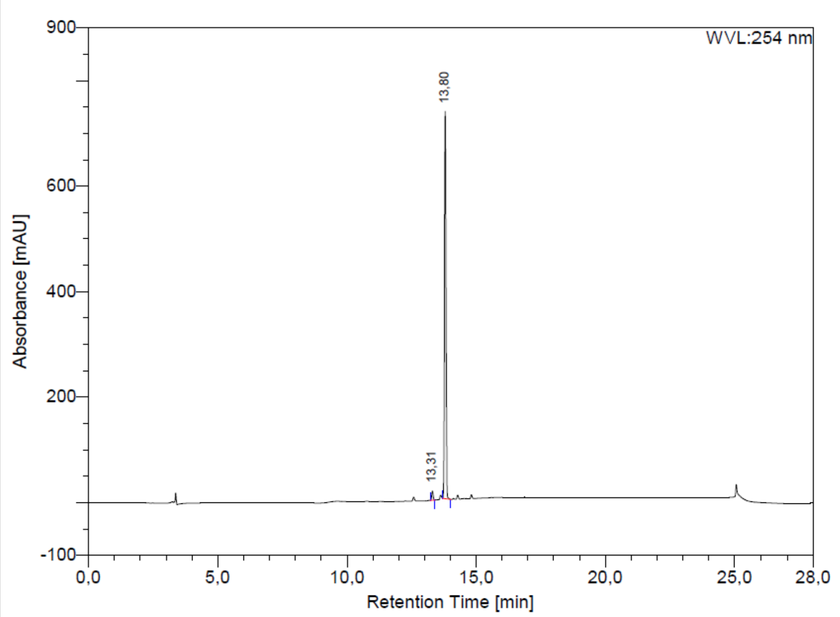

No.	Ret.Time min	Peak Name	Height mAU	Area mAU*min	Rel.Area %	Amount	Type
1	13,31	n.a.	17,768	1,142	2,22	n.a.	BMB
2	13,80	n.a.	733,903	50,405	97,78	n.a.	BMB
Total:			751,670	51,547	100,00	0,000	

2-{[5-(3-Chloro-4-methylbenzenesulfonyl)-4-oxo-1,4-dihydropyrimidin-2-yl]sulfanyl}-*N*-[3-(trifluoromethyl)phenyl]acetamide (**compound 15**)

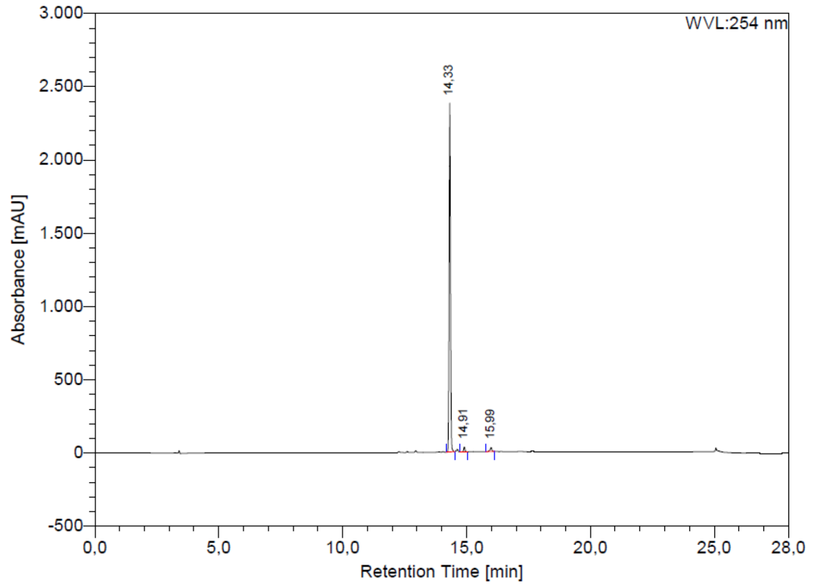

No.	Ret.Time min	Peak Name	Height mAU	Area mAU*min	Rel.Area %	Amount	Type
1	14,33	n.a.	2379,575	157,974	97,36	n.a.	BMB*
2	14,91	n.a.	33,075	2,035	1,25	n.a.	BMB*
3	15,99	n.a.	27,969	2,257	1,39	n.a.	BMB
Total:			2440,618	162,266	100,00	0,000	

2-{[5-(3-Chloro-4-methylbenzenesulfonyl)-4-oxo-1,4-dihydropyrimidin-2-yl]sulfanyl}-*N*-[4-(trifluoromethyl)phenyl]acetamide (**compound 16**)

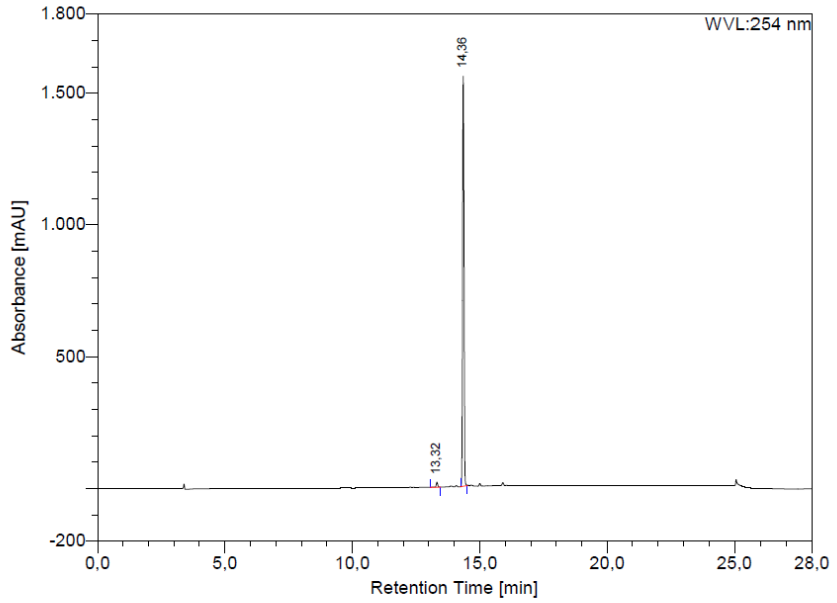

No.	Ret.Time min	Peak Name	Height mAU	Area mAU*min	Rel.Area %	Amount	Type
1	13,32	n.a.	18,426	1,200	1,15	n.a.	BMB
2	14,36	n.a.	1554,529	102,826	98,85	n.a.	BMB
Total:			1572,955	104,026	100,00	0,000	

2-{[5-(3-Chloro-4-methylbenzenesulfonyl)-4-oxo-1,4-dihydropyrimidin-2-yl]sulfanyl}-*N*-[2-(difluoromethoxy)phenyl]acetamide (**compound 17**)

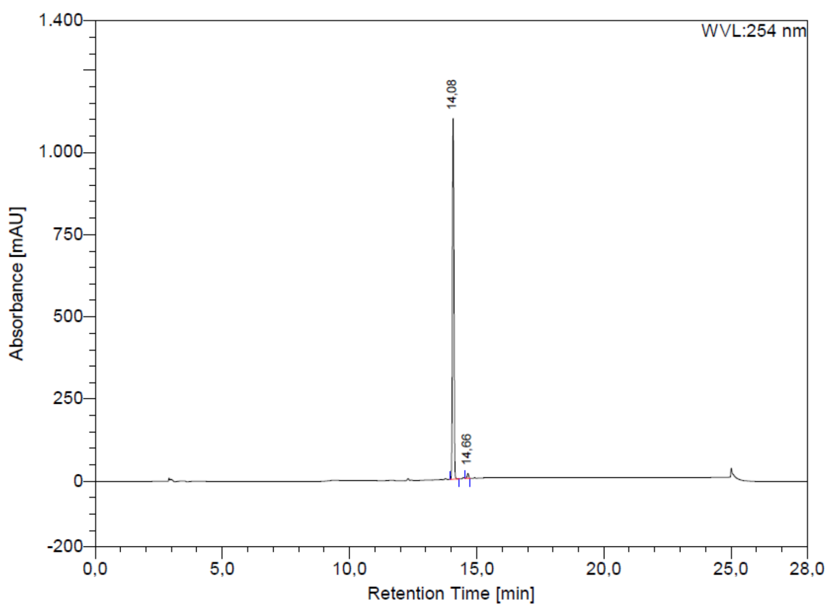

No.	Ret.Time min		Peak Name	Height mAU	Area mAU*min	Rel.Area %	Amount	Type
1	14,08	n.a.		1096,500	75,886	98,67	n.a.	BMB
2	14,66	n.a.		15,071	1,025	1,33	n.a.	BMB
Total:				1111,570	76,911	100,00	0,000	

2-{[5-(3-Chloro-4-methylbenzenesulfonyl)-4-oxo-1,4-dihydropyrimidin-2-yl]sulfanyl}-*N*-[4-(difluoromethoxy)phenyl]acetamide (**compound 18**)

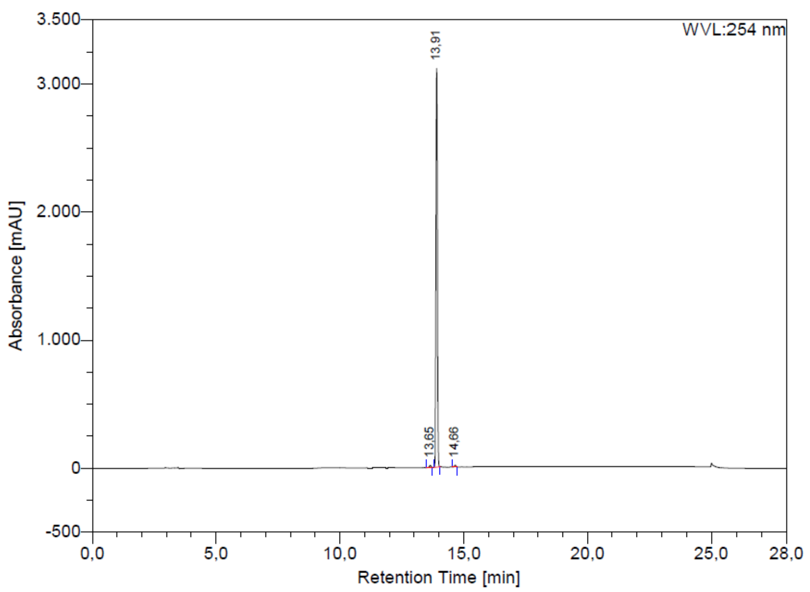

No.	Ret.Time min		Peak Name	Height mAU	Area mAU*min	Rel.Area %	Amount	Type
1	13,65	n.a.		17,581	1,294	0,59	n.a.	BMB
2	13,91	n.a.		3110,564	217,904	98,96	n.a.	BMB
3	14,66	n.a.		14,725	1,000	0,45	n.a.	BMB
Total:				3142,870	220,197	100,00	0,000	

2-{[5-(3-Chloro-4-methylbenzenesulfonyl)-4-oxo-1,4-dihydropyrimidin-2-yl]sulfanyl}-*N*-[2-(trifluoromethoxy)phenyl]acetamide (**compound 19**)

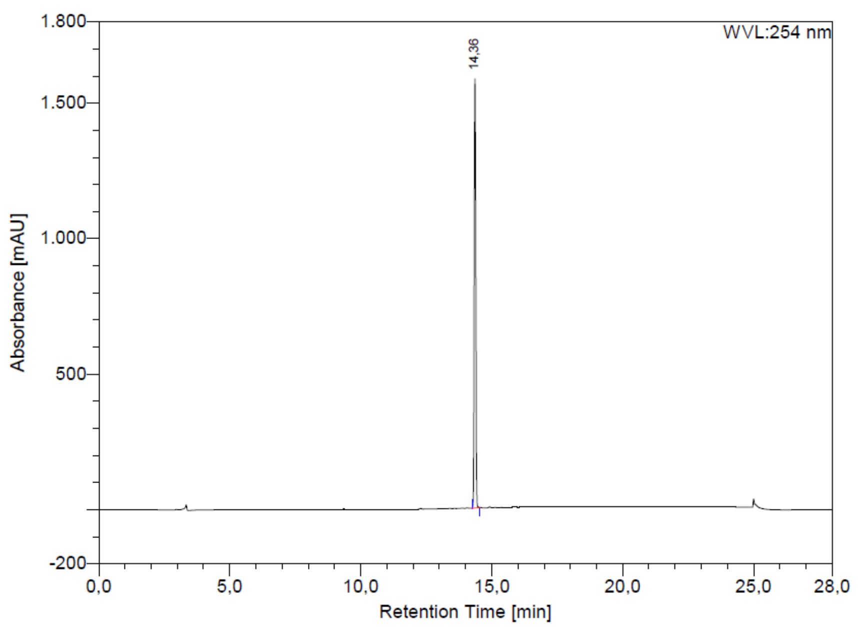

No.	Ret.Time min	Peak Name	Height mAU	Area mAU*min	Rel.Area %	Amount	Type
1	14,36	n.a.	1582,898	109,372	100,00	n.a.	BMB
Total:			1582,898	109,372	100,00	0,000	

2-{[5-(3-Chloro-4-methylbenzenesulfonyl)-4-oxo-1,4-dihydropyrimidin-2-yl]sulfanyl}-*N*-[4-(methylsulfanyl)phenyl]acetamide (**compound 20**)

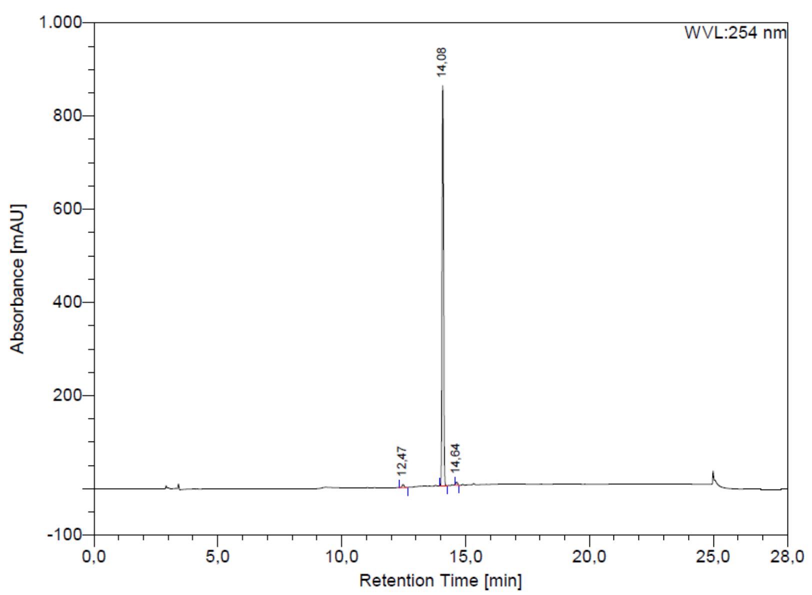

No.	Ret.Time min	Peak Name	Height mAU	Area mAU*min	Rel.Area %	Amount	Type
1	12,47	n.a.	6,674	0,622	0,98	n.a.	BMB
2	14,08	n.a.	859,063	62,587	98,13	n.a.	BMB
3	14,64	n.a.	6,715	0,571	0,89	n.a.	BMB
Total:			872,451	63,780	100,00	0,000	

3-{[5-(3-Chloro-4-methylbenzenesulfonyl)-4-oxo-1,4-dihydropyrimidin-2-yl]sulfanyl}-*N*-(4-fluorophenyl)propenamide (**compound 21**)

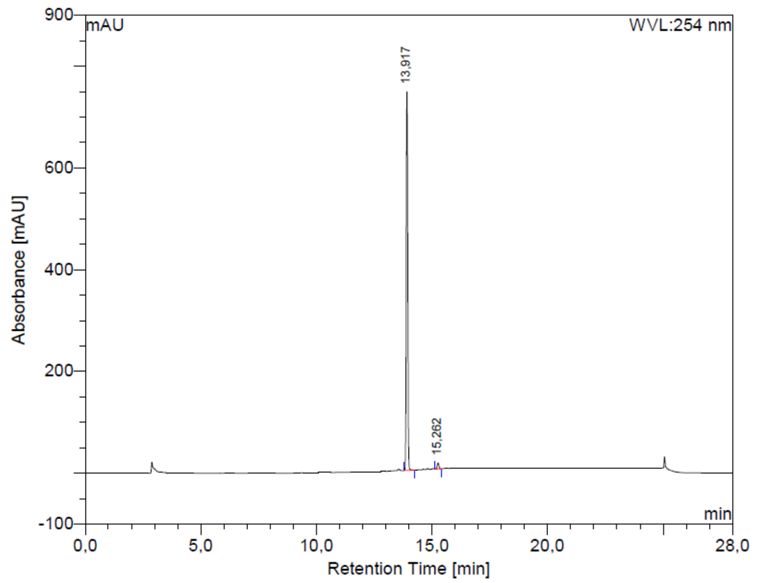

No.	Ret.Time	Height	Area	Rel.Area	Amount	Type
UV_VIS_1	UV_VIS_1	UV_VIS_1	UV_VIS_1	UV_VIS_1	UV_VIS_1	UV_VIS_1
	min	mAU	mAU*min	%		
1	13,92	744,347	57,427	98,27	n.a.	BMB
2	15,26	11,742	1,013	1,73	n.a.	BMB
Total:			756,089	58,439	100,00	0,000

3-{[5-(3-Chloro-4-methylbenzenesulfonyl)-4-oxo-1,4-dihydropyrimidin-2-yl]sulfanyl}-*N*-[4-(trifluoromethyl)phenyl]propenamide (**compound 22**)

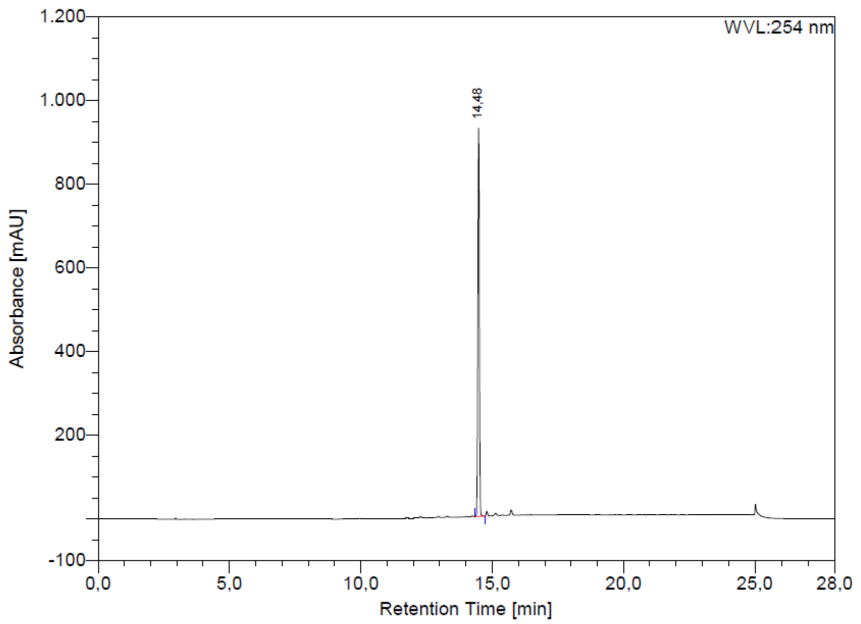

No.	Ret.Time min	Peak Name	Height mAU	Area mAU*min	Rel.Area %	Amount	Type
1	14,48	n.a.	927,860	62,594	100,00	n.a.	BMB*
Total:			927,860	62,594	100,00	0,000	

2-{[5-(3-Chloro-4-methylbenzenesulfonyl)-4-oxo-1,4-dihydropyrimidin-2-yl]sulfanyl}-*N*-[4-(trifluoromethyl)phenyl]propenamide (**compound 23**)

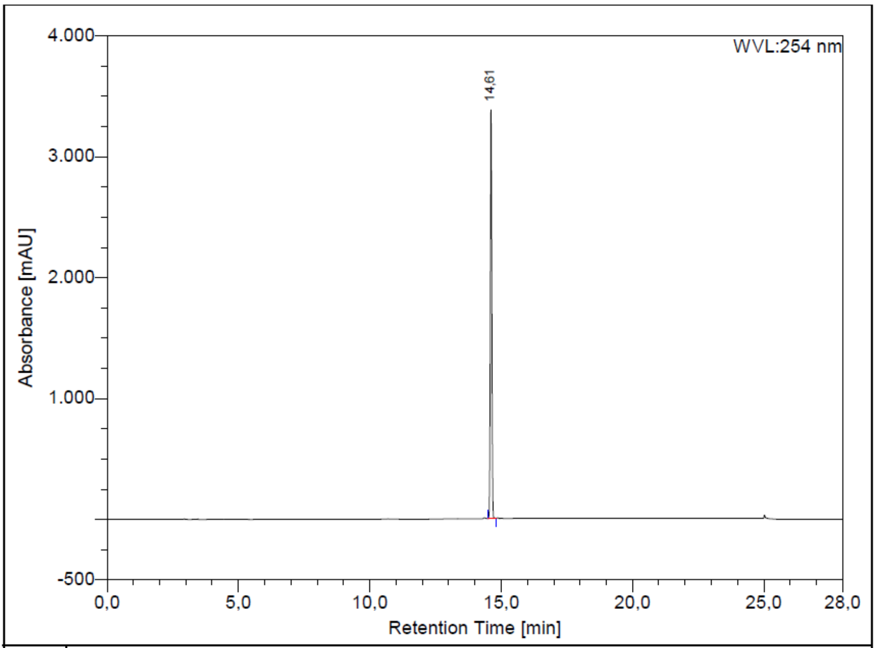

No.	Ret.Time min	Peak Name	Height mAU	Area mAU*min	Rel.Area %	Amount	Type
1	14,61	n.a.	3380,082	229,038	100,00	n.a.	BMB
Total:			3380,082	229,038	100,00	0,000	

2-{[5-(3-Chloro-4-methylbenzenesulfonyl)-4-oxo-1,4-dihydropyrimidin-2-yl]sulfanyl}-*N*-[4-(trifluoromethyl)phenyl]butanamide (**compound 24**)

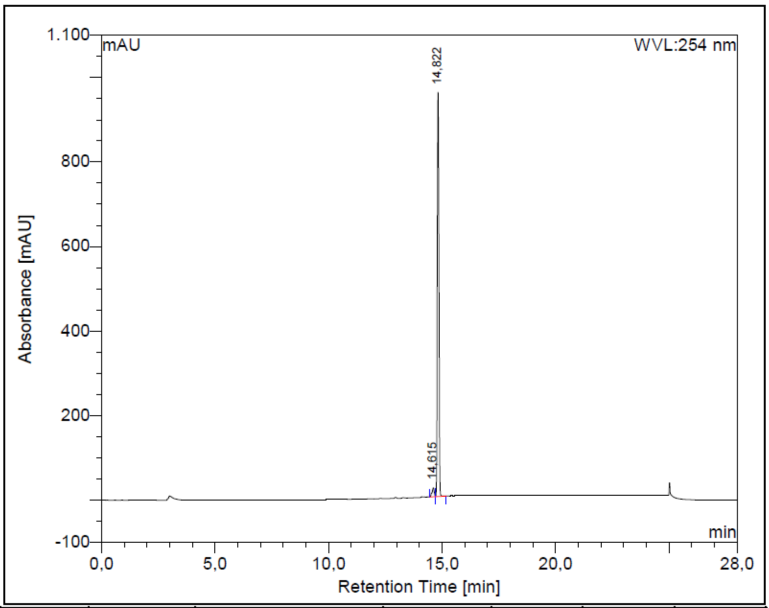

No. UV_VIS_1	Ret.Time UV_VIS_1 min	Height UV_VIS_1 mAU	Area UV_VIS_1 mAU*min	Rel.Area UV_VIS_1 %	Amount UV_VIS_1	Type UV_VIS_1
1	14,62	20,955	2,266	2,83	n.a.	BM
2	14,82	956,391	77,758	97,17	n.a.	MB
Total:			977,346	80,024	100,00	0,000

2-{[5-(3-Chloro-4-methylbenzenesulfonyl)-4-oxo-1,4-dihydropyrimidin-2-yl]sulfanyl}-3-methyl-*N*-[4-(trifluoromethyl)phenyl]butanamide (**compound 25**)

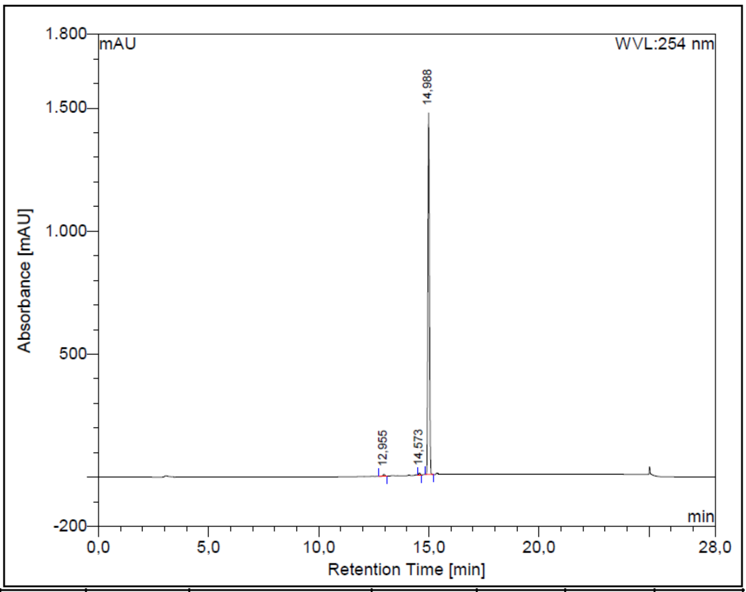

No.	Ret.Time	Height	Area	Rel.Area	Amount	Type
UV_VIS_1	UV_VIS_1	UV_VIS_1	UV_VIS_1	UV_VIS_1	UV_VIS_1	UV_VIS_1
	min	mAU	mAU*min	%		
1	12,96	7,560	0,729	0,59	n.a.	BMB
2	14,57	8,459	0,700	0,57	n.a.	BMB
3	14,99	1469,086	121,682	98,84	n.a.	BMB
**Total:**			1485,105	123,112	100,00	0,000

2-{[5-(3-Chloro-4-methylbenzenesulfonyl)-4-oxo-1,4-dihydropyrimidin-2-yl]sulfanyl}-2-phenyl-*N*-[4-(trifluoromethyl)phenyl]acetamide (**compound 26**)

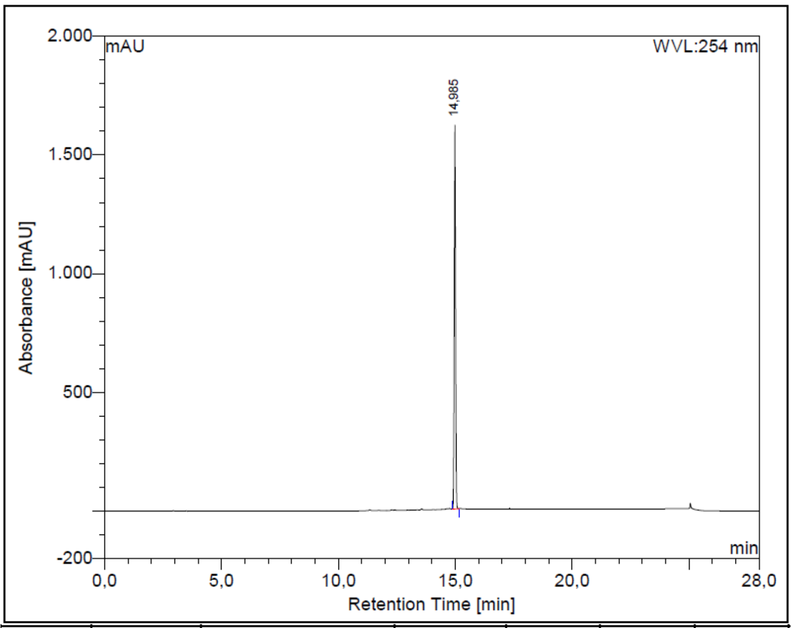

No.	Ret.Time	Height	Area	Rel.Area	Amount	Type
UV_VIS_1	UV_VIS_1	UV_VIS_1	UV_VIS_1	UV_VIS_1	UV_VIS_1	UV_VIS_1
	min	mAU	mAU*min	%		
1	14,99	1616,068	110,777	100,00	n.a.	BMB
**Total:**			1616,068	110,777	100,00	0,000

2-{[5-(4-Methylbenzenesulfonyl)-4-oxo-1,4-dihydropyrimidin-2-yl]sulfanyl}-*N*-[4-(trifluoromethyl)phenyl]acetamide (**compound 27**)

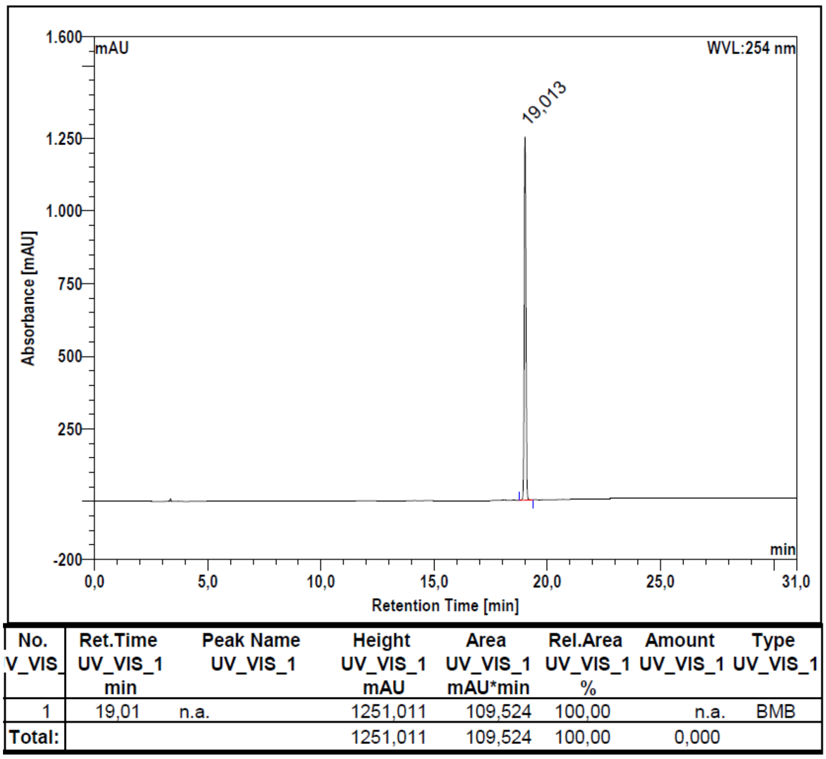

No. V_VIS_	Ret.Time UV_VIS_1 min	Peak Name UV_VIS_1	Height UV_VIS_1 mAU	Area UV_VIS_1 mAU*min	Rel.Area UV_VIS_1 %	Amount UV_VIS_1	Type UV_VIS_1
1	19,01	n.a.	1251,011	109,524	100,00	n.a.	BMB
Total:			1251,011	109,524	100,00	0,000	

2-{[5-(3-Chlorobenzenesulfonyl)-4-oxo-1,4-dihydropyrimidin-2-yl]sulfanyl}-*N*-[4-(trifluoromethyl)phenyl]acetamide (**compound 28**)

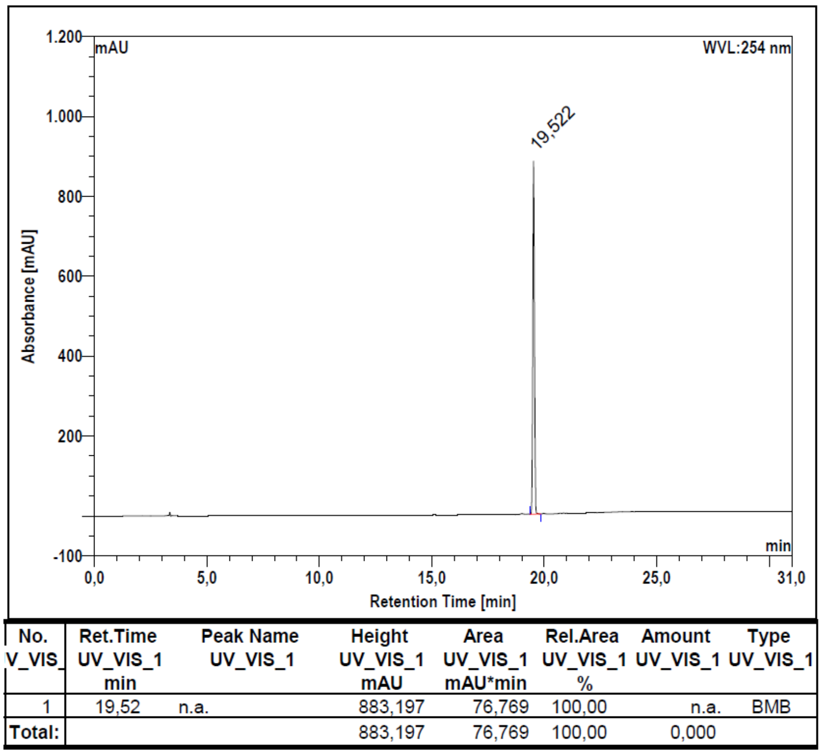

No. V_VIS_	Ret.Time UV_VIS_1 min	Peak Name UV_VIS_1	Height UV_VIS_1 mAU	Area UV_VIS_1 mAU*min	Rel.Area UV_VIS_1 %	Amount UV_VIS_1	Type UV_VIS_1
1	19,52	n.a.	883,197	76,769	100,00	n.a.	BMB
Total:			883,197	76,769	100,00	0,000	

2-{[5-(4-Fluorobenzenesulfonyl)-4-oxo-1,4-dihydropyrimidin-2-yl]sulfanyl}-*N*-[4-(trifluoromethyl)phenyl]acetamide (**compound 29**)

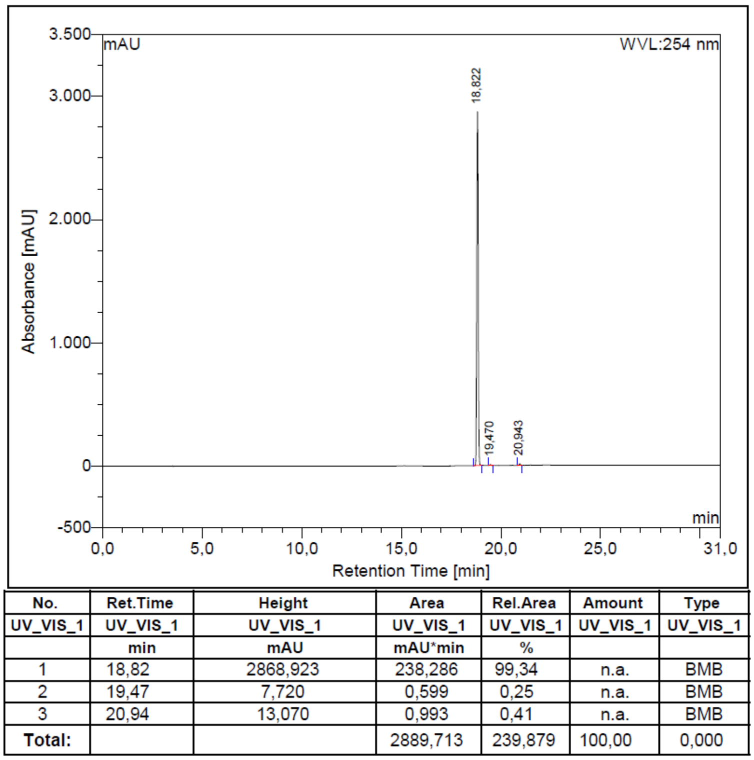

No.	Ret.Time	Height	Area	Rel.Area	Amount	Type
UV_VIS_1	UV_VIS_1	UV_VIS_1	UV_VIS_1	UV_VIS_1	UV_VIS_1	UV_VIS_1
	min	mAU	mAU*min	%		
1	18,82	2868,923	238,286	99,34	n.a.	BMB
2	19,47	7,720	0,599	0,25	n.a.	BMB
3	20,94	13,070	0,993	0,41	n.a.	BMB
Total:			2889,713	239,879	100,00	0,000

2-{[5-(4-Chlorobenzenesulfonyl)-4-oxo-1,4-dihydropyrimidin-2-yl]sulfanyl}-*N*-[4-(trifluoromethyl)phenyl]acetamide (**compound 30**)

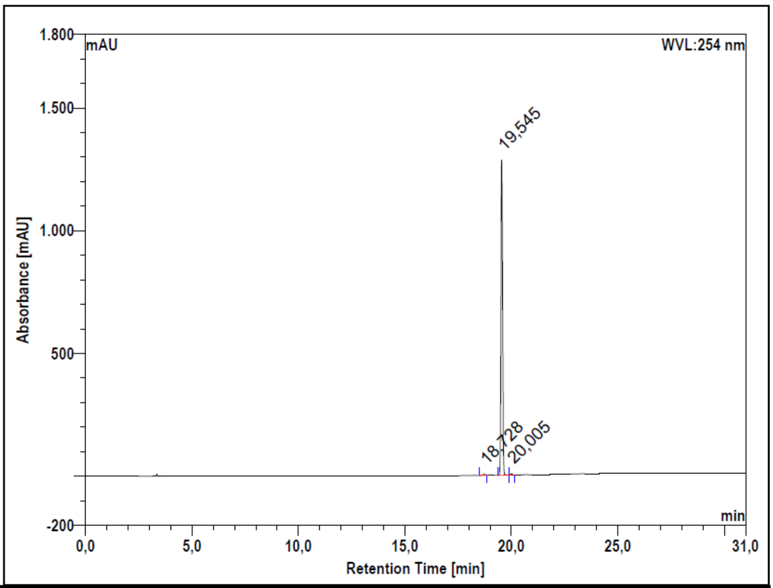

No.	Ret.Time	Peak Name	Height	Area	Rel.Area	Amount	Type
V_VIS_	UV_VIS_1	UV_VIS_1	UV_VIS_1	UV_VIS_1	UV_VIS_1	UV_VIS_1	UV_VIS_1
	min		mAU	mAU*min	%		
1	18,73	n.a.	4,409	0,510	0,44	n.a.	BMB
2	19,55	n.a.	1283,273	113,592	99,12	n.a.	BM
3	20,01	n.a.	5,474	0,502	0,44	n.a.	MB
Total:			1293,156	114,604	100,00	0,000	

2-{[5-(4-Bromobenzenesulfonyl)-4-oxo-1,4-dihydropyrimidin-2-yl]sulfanyl}-*N*-[4-(trifluoro-methyl)phenyl]acetamide (**compound 31**)

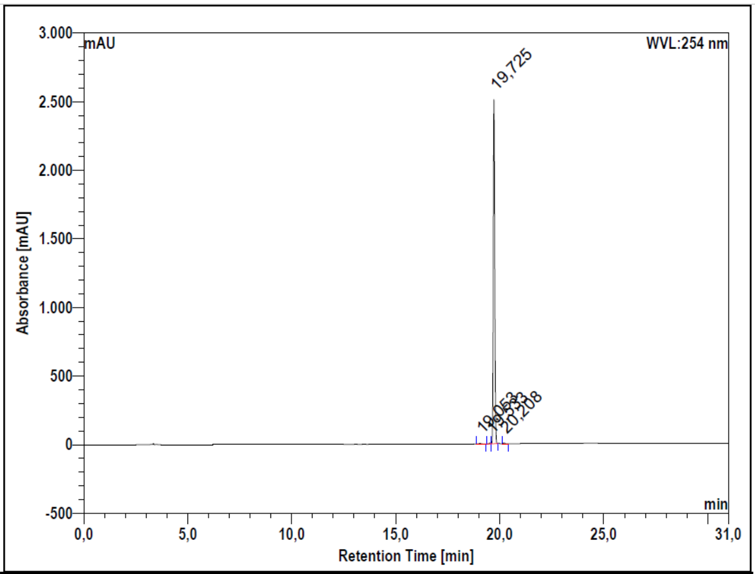

No. V_VIS_	Ret.Time UV_VIS_1 min	Peak Name UV_VIS_1	Height UV_VIS_1 mAU	Area UV_VIS_1 mAU*min	Rel.Area UV_VIS_1 %	Amount UV_VIS_1	Type UV_VIS_1
1	19,05	n.a.	5,391	0,752	0,34	n.a.	BMB
2	19,53	n.a.	7,093	0,553	0,25	n.a.	BM
3	19,73	n.a.	2507,470	216,400	99,08	n.a.	MB
4	20,21	n.a.	8,006	0,703	0,32	n.a.	BMB
Total:			2527,960	218,409	100,00	0,000	

2-{[5-(4-Iodobenzenesulfonyl)-4-oxo-1,4-dihydropyrimidin-2-yl]sulfanyl}-*N*-[4-(trifluoro-methyl)phenyl]acetamide (**compound 32**)

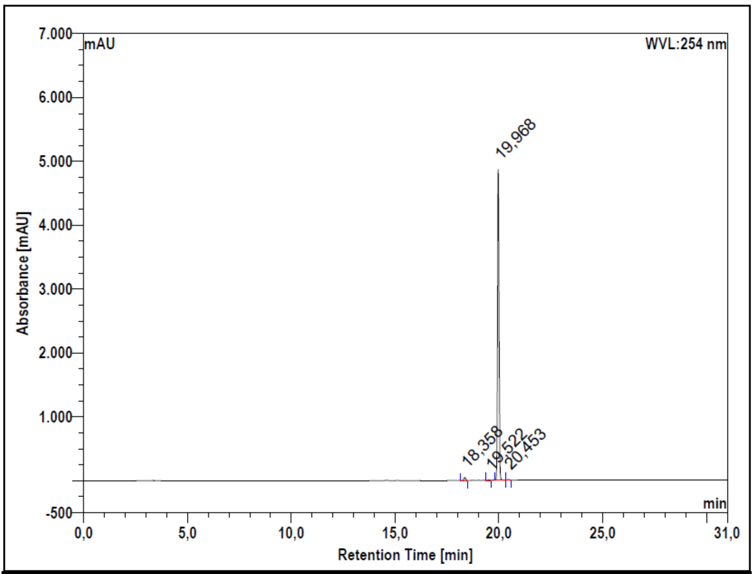

No. V_VIS_	Ret.Time UV_VIS_1 min	Peak Name UV_VIS_1	Height UV_VIS_1 mAU	Area UV_VIS_1 mAU*min	Rel.Area UV_VIS_1 %	Amount UV_VIS_1	Type UV_VIS_1
1	18,36	n.a.	50,658	4,819	1,11	n.a.	BMB
2	19,52	n.a.	10,758	1,096	0,25	n.a.	BMB
3	19,97	n.a.	4869,024	427,298	98,26	n.a.	BM
4	20,45	n.a.	13,277	1,659	0,38	n.a.	MB
Total:			4943,716	434,872	100,00	0,000	

2-({4-Oxo-5-[4-(trifluoromethyl)benzenesulfonyl]-1,4-dihydropyrimidin-2-yl}sulfanyl)-*N*-[4-(trifluoromethyl)phenyl]acetamide (**compound 33**)

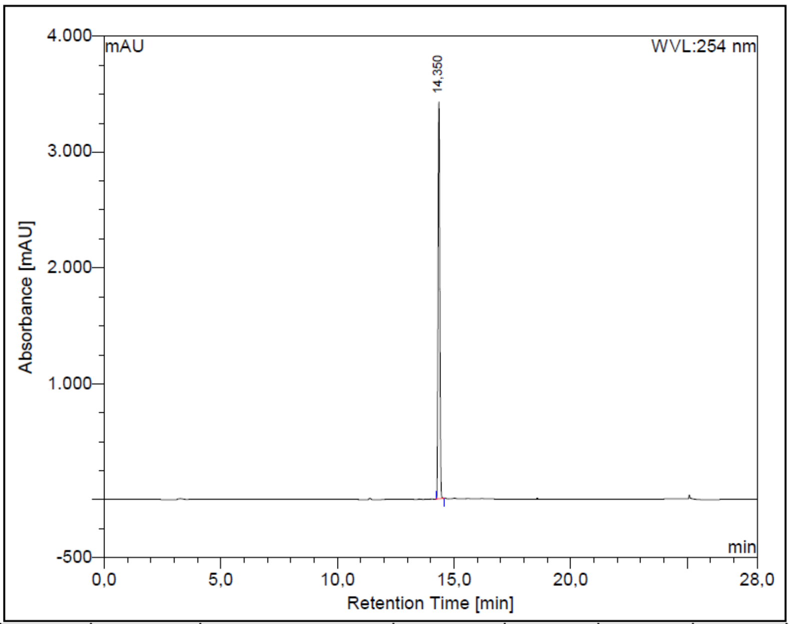

No.	Ret.Time	Height	Area	Rel.Area	Amount	Type
UV_VIS_1	UV_VIS_1	UV_VIS_1	UV_VIS_1	UV_VIS_1	UV_VIS_1	UV_VIS_1
	min	mAU	mAU*min	%		
1	14,35	3426,151	299,387	100,00	n.a.	BMB
Total:			3426,151	299,387	100,00	0,000

2-{[5-(4-Nitrobenzenesulfonyl)-4-oxo-1,4-dihydropyrimidin-2-yl]sulfanyl}-*N*-[4-(trifluoro-methyl)phenyl]acetamide (**compound 34**)

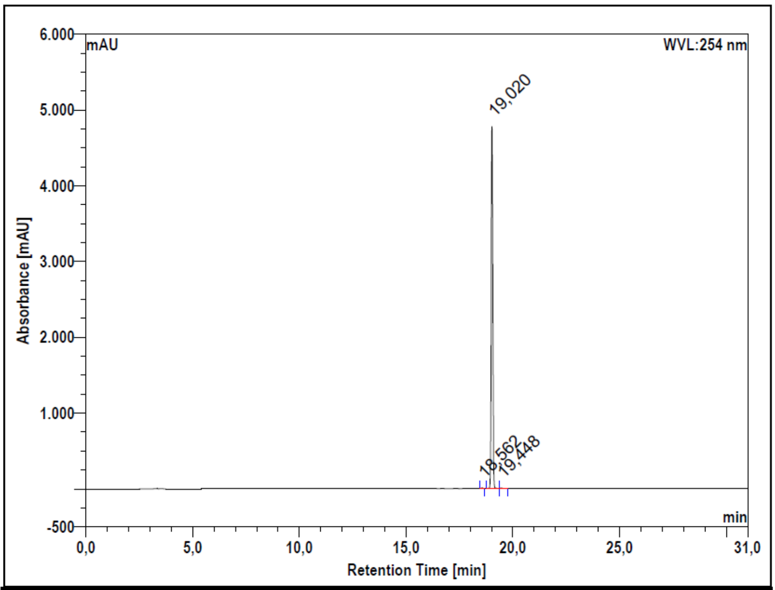

No.	Ret.Time	Peak Name	Height	Area	Rel.Area	Amount	Type
V_VIS_	UV_VIS_1	UV_VIS_1	UV_VIS_1	UV_VIS_1	UV_VIS_1	UV_VIS_1	UV_VIS_1
	min		mAU	mAU*min	%		
1	18,56	n.a.	10,453	0,873	0,21	n.a.	BMB
2	19,02	n.a.	4777,185	407,822	99,57	n.a.	BM
3	19,45	n.a.	7,719	0,881	0,22	n.a.	MB
Total:			4795,357	409,576	100,00	0,000	

2-{[5-(4-Methoxybenzenesulfonyl)-4-oxo-1,4-dihydropyrimidin-2-yl]sulfanyl}-*N*-[4-(trifluoromethyl)phenyl]acetamide (**compound 35**)

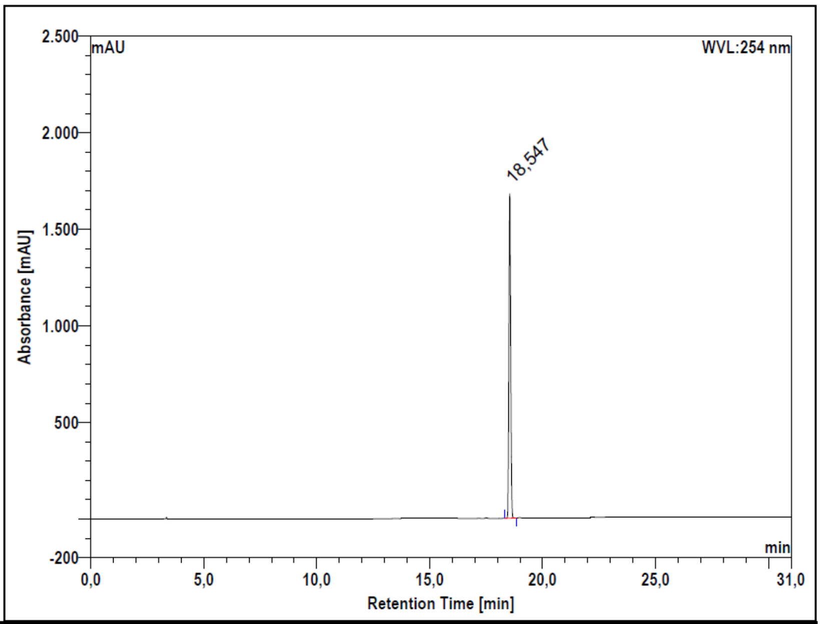

No. V_VIS	Ret.Time UV_VIS_1 min	Peak Name UV_VIS_1	Height UV_VIS_1 mAU	Area UV_VIS_1 mAU*min	Rel.Area UV_VIS_1 %	Amount UV_VIS_1	Type UV_VIS_1
1	18,55	n.a.	1679,784	153,087	100,00	n.a.	BMB*
Total:			1679,784	153,087	100,00	0,000	

2-{[5-(Benzenesulfonyl)-4-oxo-1,4-dihydropyrimidin-2-yl]sulfanyl}-*N*-[4-(trifluoro-methyl)phenyl]acetamide (**compound 36**)

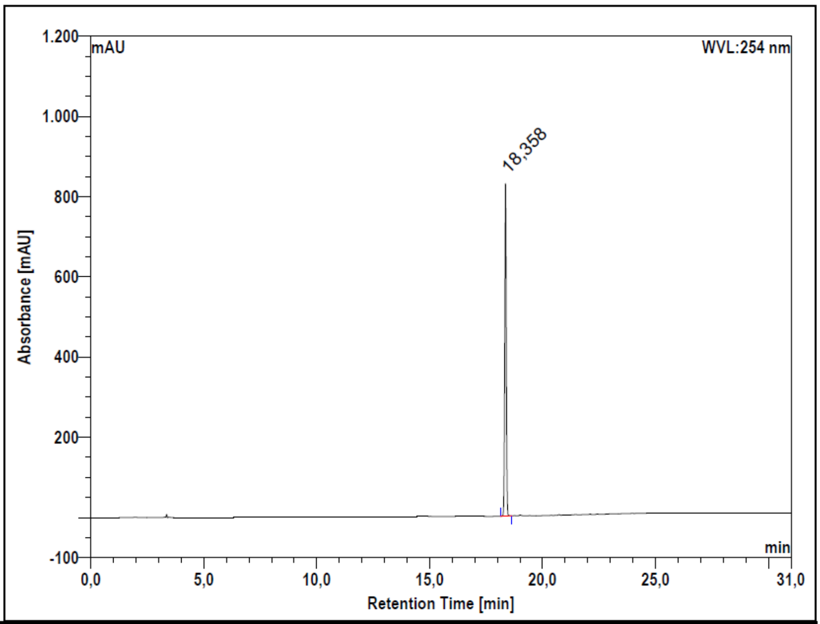

No. V_VIS	Ret.Time UV_VIS_1 min	Peak Name UV_VIS_1	Height UV_VIS_1 mAU	Area UV_VIS_1 mAU*min	Rel.Area UV_VIS_1 %	Amount UV_VIS_1	Type UV_VIS_1
1	18,36	n.a.	829,088	67,299	100,00	n.a.	BMB
Total:			829,088	67,299	100,00	0,000	

2-{[5-(2-Chlorobenzenesulfonyl)-4-oxo-1,4-dihydropyrimidin-2-yl]sulfanyl}-*N*-[4-(trifluoromethyl)phenyl]acetamide (**compound 37**)

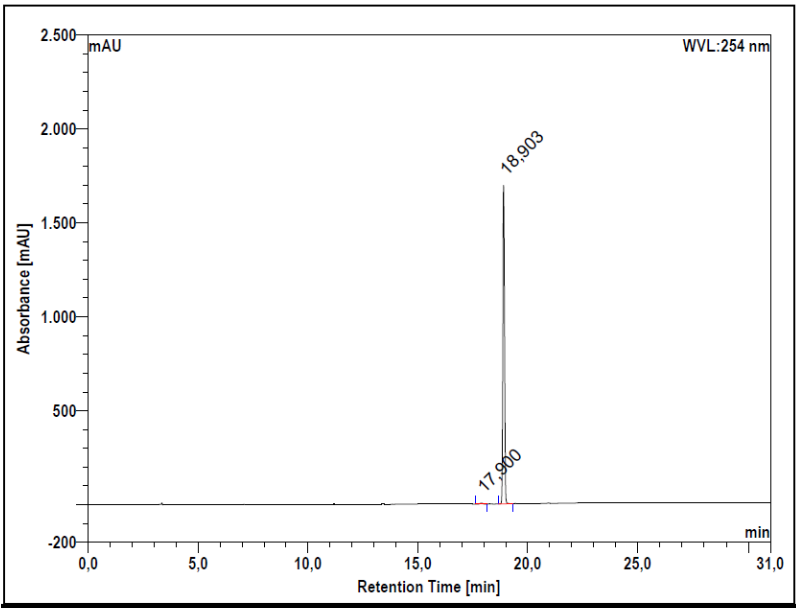

No.	Ret.Time	Peak Name	Height	Area	Rel.Area	Amount	Type
V_VIS_	UV_VIS_1	UV_VIS_1	UV_VIS_1	UV_VIS_1	UV_VIS_1	UV_VIS_1	UV_VIS_1
	min		mAU	mAU*min	%		
1	17,90	n.a.	4,696	0,720	0,48	n.a.	BMB
2	18,90	n.a.	1696,800	150,654	99,52	n.a.	BMB
**Total:**			1701,496	151,374	100,00	0,000	

2-({4-Oxo-5-[3-(trifluoromethyl)benzenesulfonyl]-1,4-dihydropyrimidin-2-yl}sulfanyl)-*N*-[4-(trifluoromethyl)phenyl]acetamide (**compound 38**)

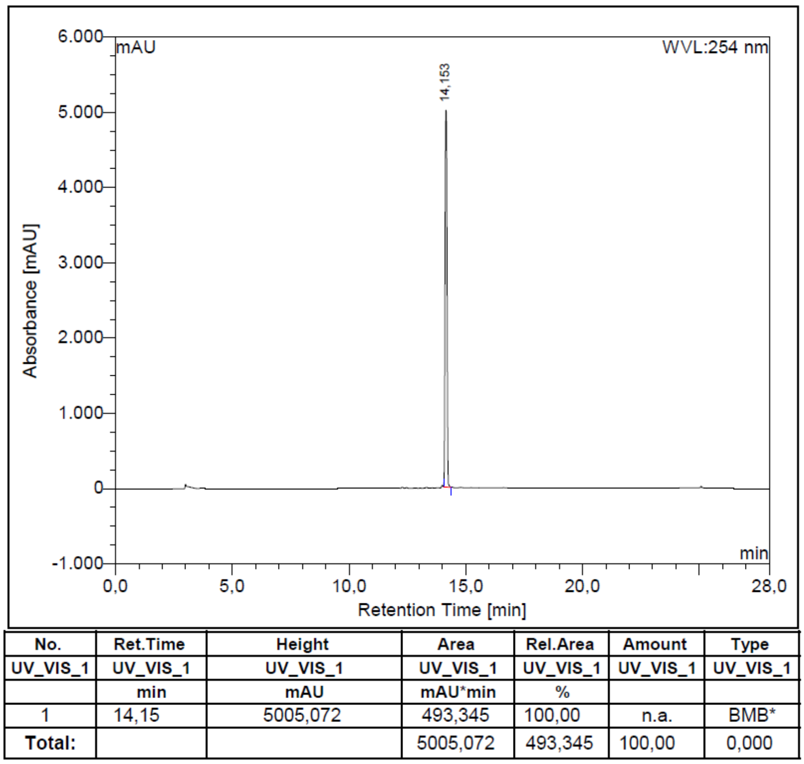

No.	Ret.Time	Height	Area	Rel.Area	Amount	Type
UV_VIS_1	UV_VIS_1	UV_VIS_1	UV_VIS_1	UV_VIS_1	UV_VIS_1	UV_VIS_1
	min	mAU	mAU*min	%		
1	14,15	5005,072	493,345	100,00	n.a.	BMB*
**Total:**			5005,072	493,345	100,00	0,000

2-[(5-{[1,1'-Biphenyl]-4-sulfonyl}-4-oxo-1,4-dihydropyrimidin-2-yl)sulfanyl]-*N*-[4-(trifluoromethyl)phenyl]acetamide (**compound 39**)

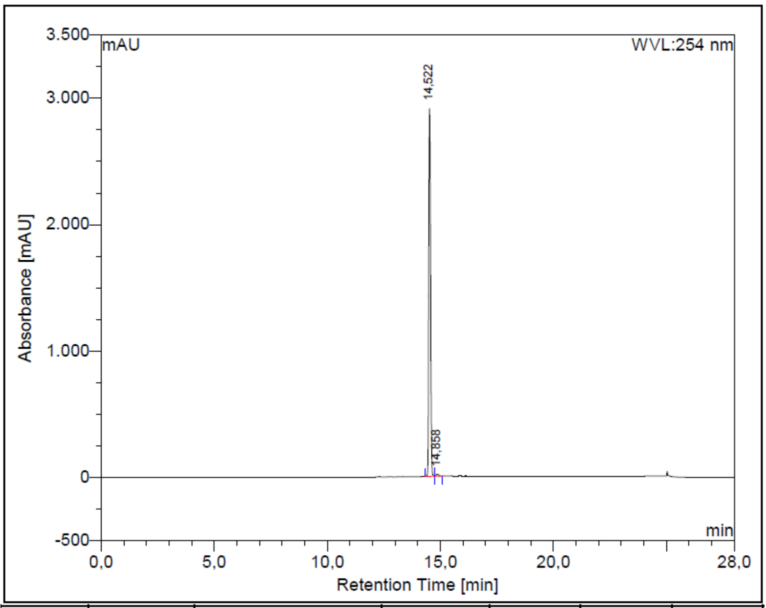

No.	Ret.Time	Height	Area	Rel.Area	Amount	Type
UV_VIS_1	UV_VIS_1	UV_VIS_1	UV_VIS_1	UV_VIS_1	UV_VIS_1	UV_VIS_1
	min	mAU	mAU*min	%		
1	14,52	2910,141	273,539	99,19	n.a.	BM
2	14,86	15,860	2,240	0,81	n.a.	MB
Total:			2926,001	275,779	100,00	0,000

2-{[5-(4-Benzylbenzenesulfonyl)-4-oxo-1,4-dihydropyrimidin-2-yl]sulfanyl}-*N*-[4-(trifluoromethyl)phenyl]acetamide (**compound 40**)

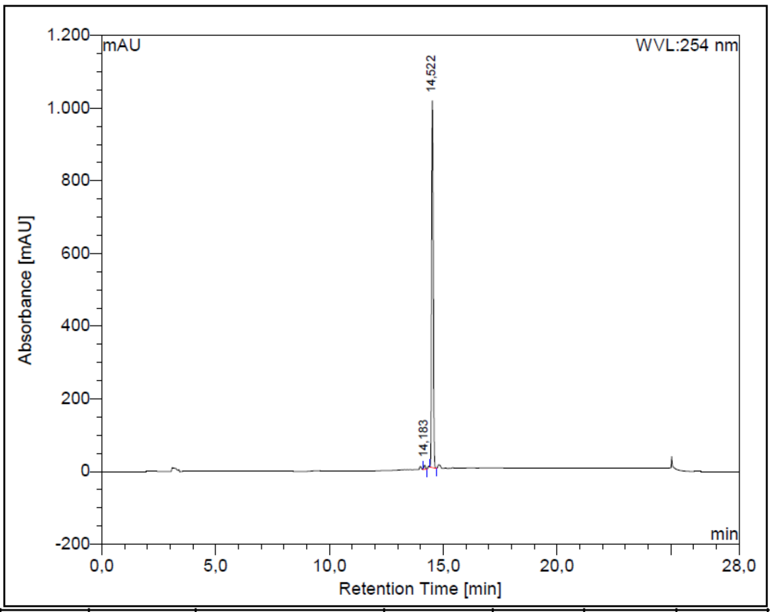

No.	Ret.Time	Height	Area	Rel.Area	Amount	Type
UV_VIS_1	UV_VIS_1	UV_VIS_1	UV_VIS_1	UV_VIS_1	UV_VIS_1	UV_VIS_1
	min	mAU	mAU*min	%		
1	14,18	11,107	0,905	1,03	n.a.	BMB*
2	14,52	1009,126	87,368	98,97	n.a.	BMB
Total:			1020,233	88,273	100,00	0,000

2-{[4-Oxo-5-(pyridine-2-sulfonyl)-1,4-dihydropyrimidin-2-yl]sulfanyl}-*N*-[4-(trifluoro-methyl)phenyl]acetamide (**compound 41**)

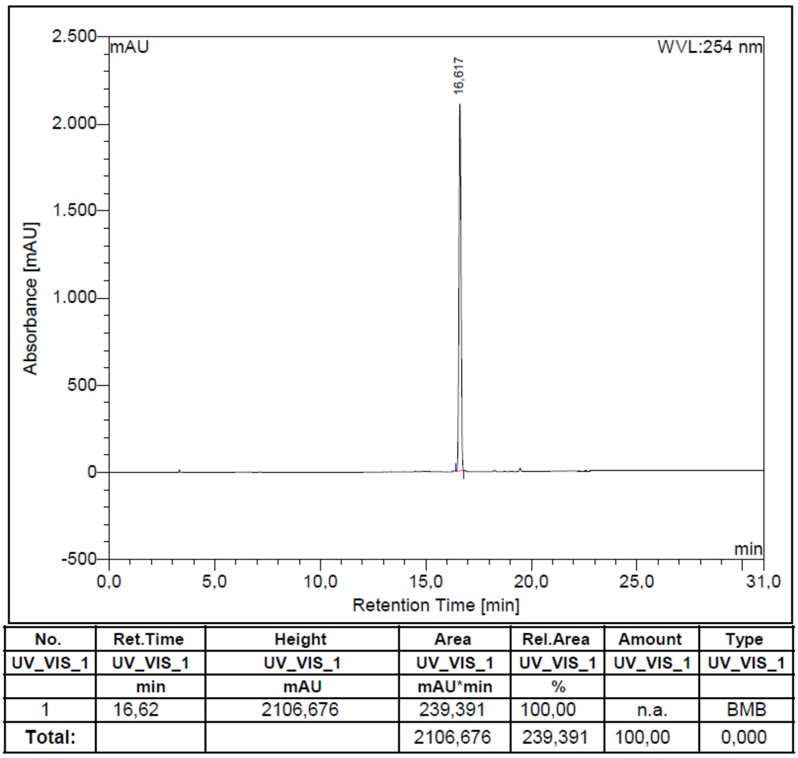

No.	Ret.Time	Height	Area	Rel.Area	Amount	Type
UV_VIS_1	UV_VIS_1	UV_VIS_1	UV_VIS_1	UV_VIS_1	UV_VIS_1	UV_VIS_1
	min	mAU	mAU*min	%		
1	16,62	2106,676	239,391	100,00	n.a.	BMB
**Total:**			2106,676	239,391	100,00	0,000

2-{[5-(Naphthalene-2-sulfonyl)-4-oxo-1,4-dihydropyrimidin-2-yl]sulfanyl}-*N*-[4-(trifluoro-methyl)phenyl]acetamide (**compound 42**)

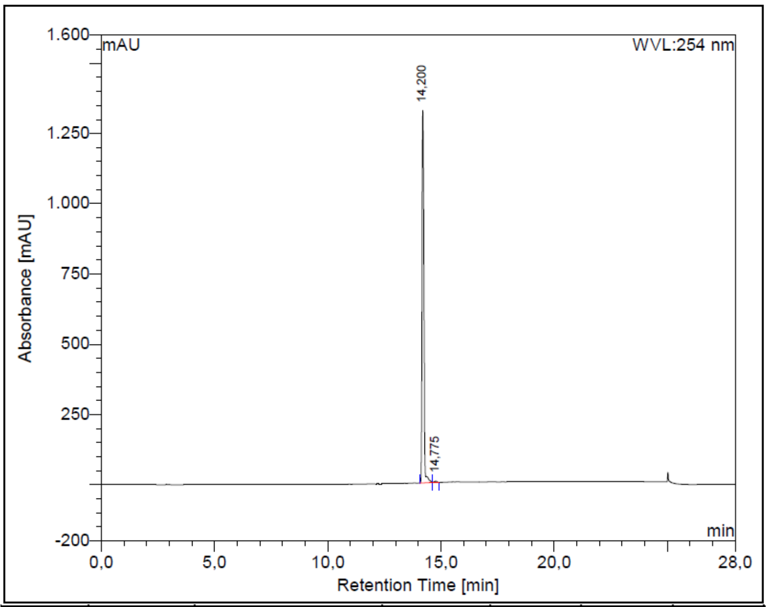

No.	Ret.Time	Height	Area	Rel.Area	Amount	Type
UV_VIS_1	UV_VIS_1	UV_VIS_1	UV_VIS_1	UV_VIS_1	UV_VIS_1	UV_VIS_1
	min	mAU	mAU*min	%		
1	14,20	1325,162	125,213	99,55	n.a.	BMB
2	14,78	4,624	0,571	0,45	n.a.	BMB
**Total:**			1329,785	125,785	100,00	0,000

2-{[4-Oxo-5-(quinoline-8-sulfonyl)-1,4-dihydropyrimidin-2-yl]sulfanyl}-*N*-[4-(trifluoromethyl)phenyl]acetamide (**compound 43**)

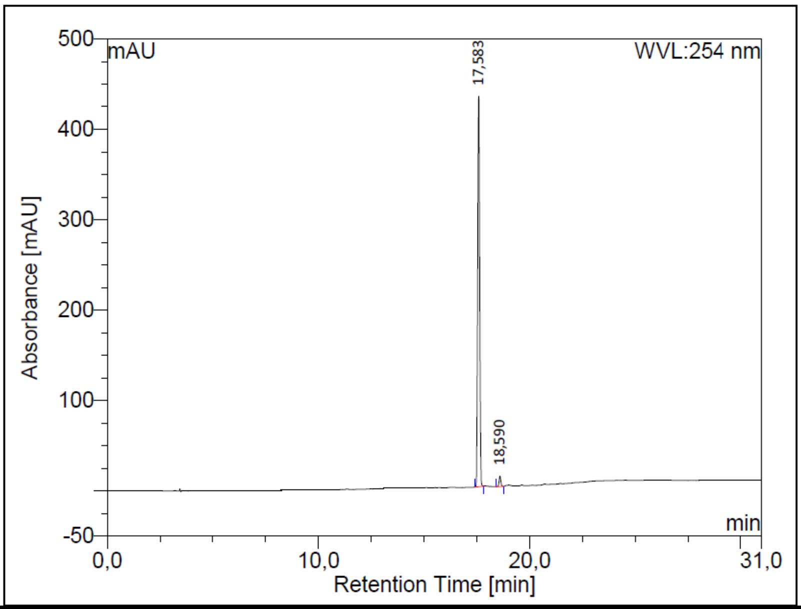

No. V_VIS_	Ret.Time UV_VIS_1 min	Peak Name UV_VIS_1	Height UV_VIS_1 mAU	Area UV_VIS_1 mAU*min	Rel.Area UV_VIS_1 %	Amount UV_VIS_1	Type UV_VIS_1
1	17,58	n.a.	431,918	46,872	97,77	n.a.	BMB
2	18,59	n.a.	11,490	1,070	2,23	n.a.	BMB
Total:			443,408	47,943	100,00	0,000	

2. NMR spectra

Proton, carbon, and fluorine NMR spectra were recorded using a Varian MERCURY PLUS or a Brucker ADVANCE III HD 400 (frequencies: ^{1}H: 400 MHz, 300 MHz; ^{19}F: 282 MHz, 376 MHz; ^{13}C{^{1}H}: 101 MHz, 75 MHz). Chemical shifts (δ) were reported in parts per million (ppm) and for proton and carbon NMR relative to the solvent residual signals $CDCl_3$ (^{1}H: 7.26 ppm, ^{13}C: 77.00 ppm) and DMSO-d_6 (^{1}H: 2.50 ppm, ^{13}C{^{1}H}: 39.52 ppm). NMR raw data were processed using Mestre Lab MESTRENOVA. NMR data are reported as follows: chemical shift, multiplicity (s = singlet, d = duplet, br s = broad singlet, t = triplet, q = quartet, hept = septet, m = multiplet, dd = duplet of duplets), coupling constants (Hz) and for 1H integrals.

NMR spectra of **X11a-k,o; X12a-k,o; X13a-k,o** were reported previously in *J. Org. Chem.* 2024, 89, 21, 15990–15994 (https://pubs.acs.org/doi/10.1021/acs.joc.4c01357).

The amides **X8a-z** were synthesized based on *J. Med. Chem.* 2014, 57, 18, 7590–7599 (dx.doi.org/10.1021/jm5006918) and are commercially available.

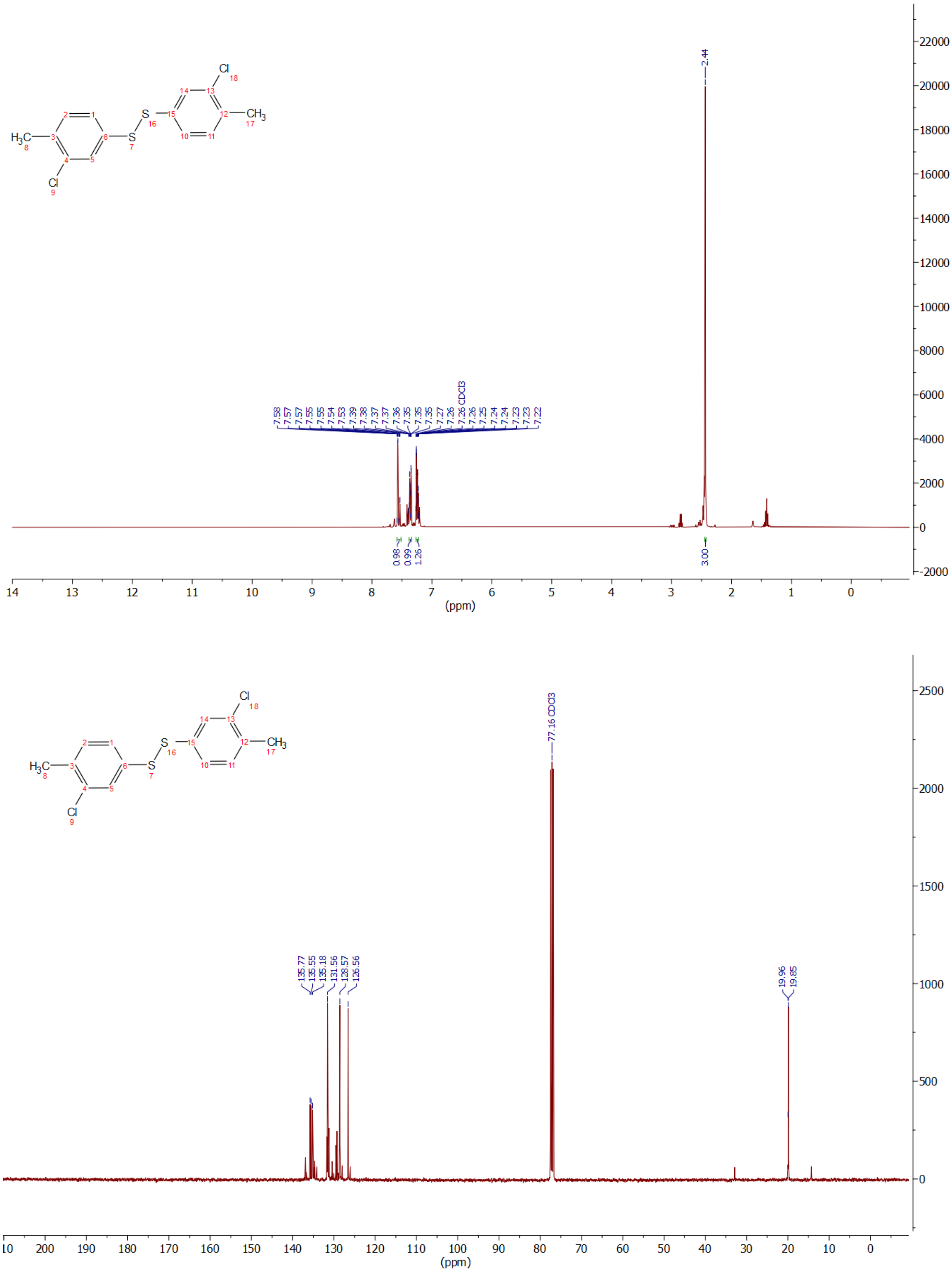

Figure S1. *^{1}H (400 MHz) and $^{13}C\{1H\}$ (101 MHz) NMR spectra in $CDCl_3$of* ***X2***

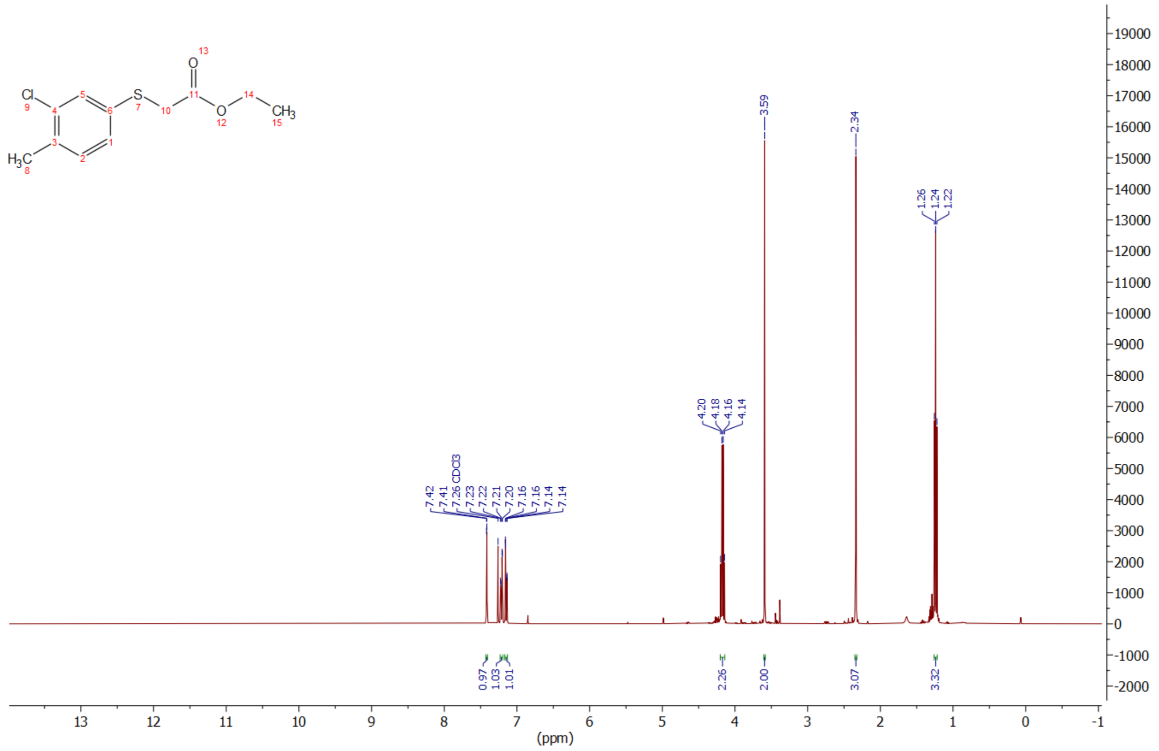

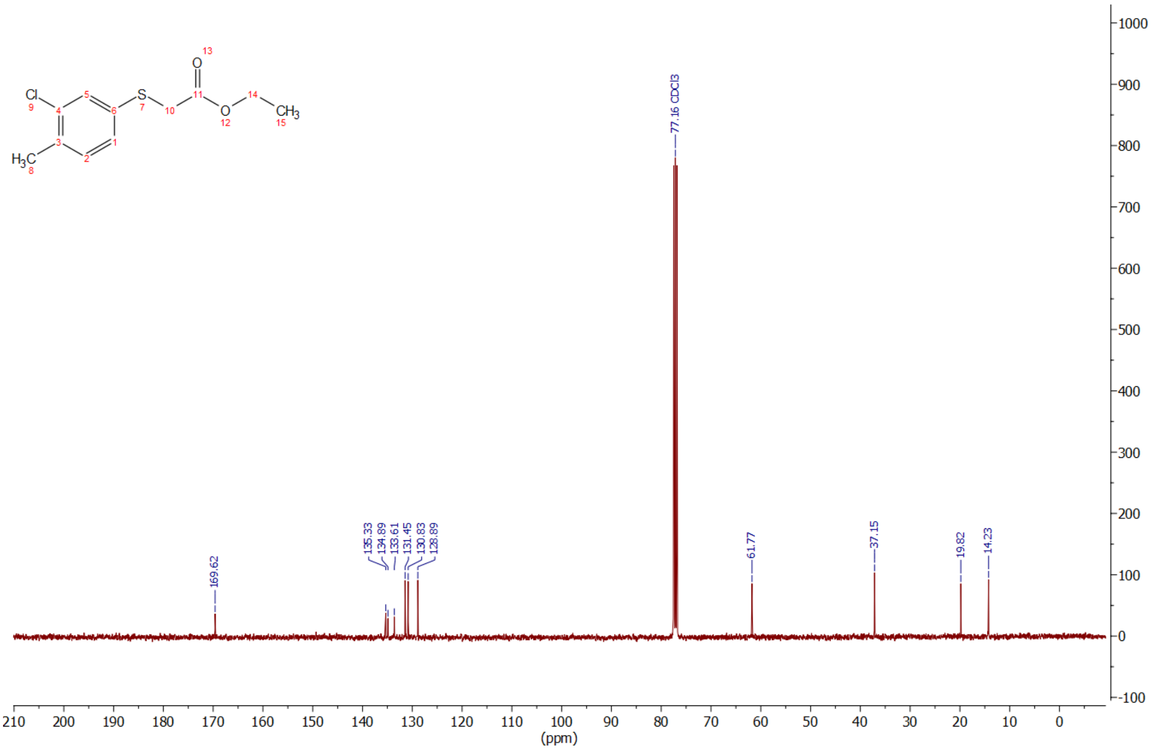

Figure S2. *^{1}H (400 MHz) and $^{13}C\{1H\}$ (101 MHz) NMR spectra in $CDCl_3$ of **X4***

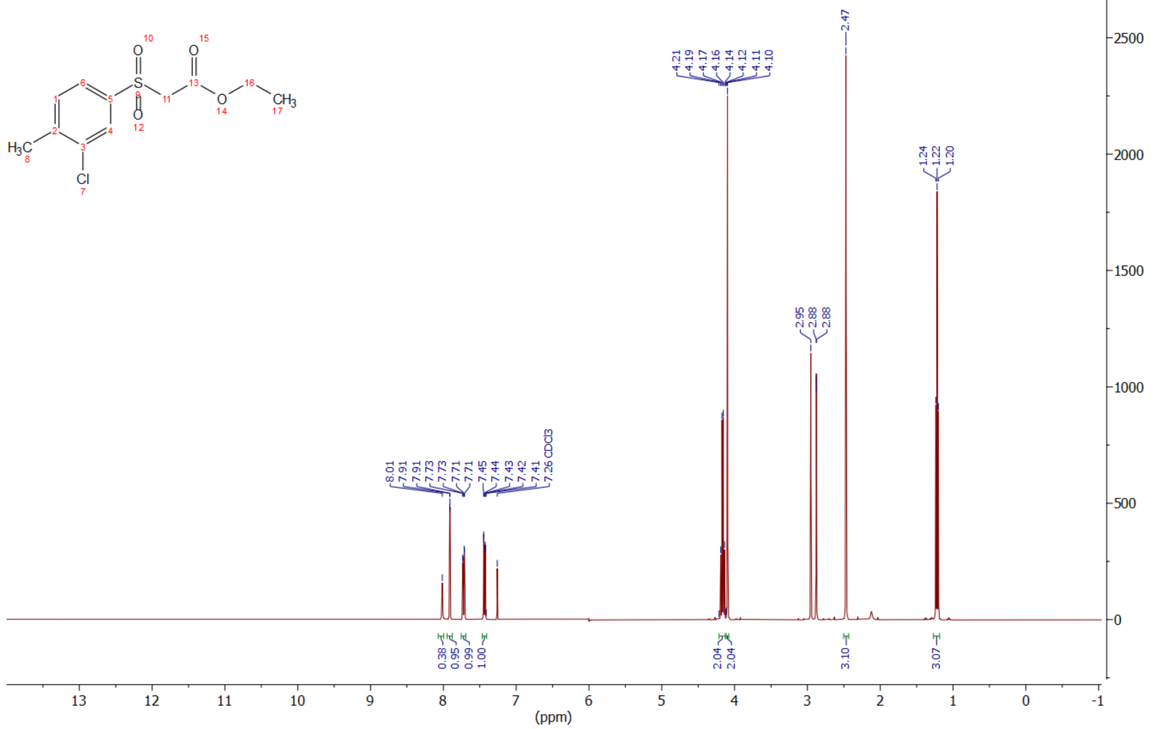

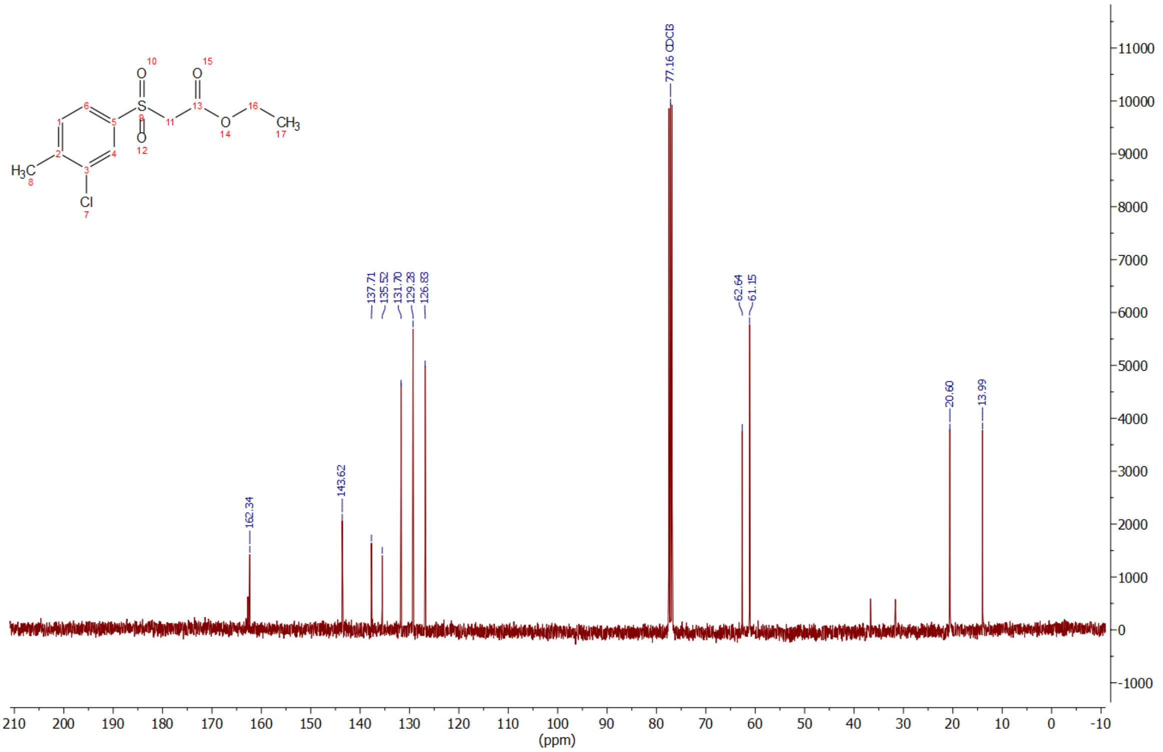

Figure S3. *^{1}H (400 MHz) and ^{13}C{1H} (101 MHz) NMR spectra in $CDCl_3$ of* ***X5***

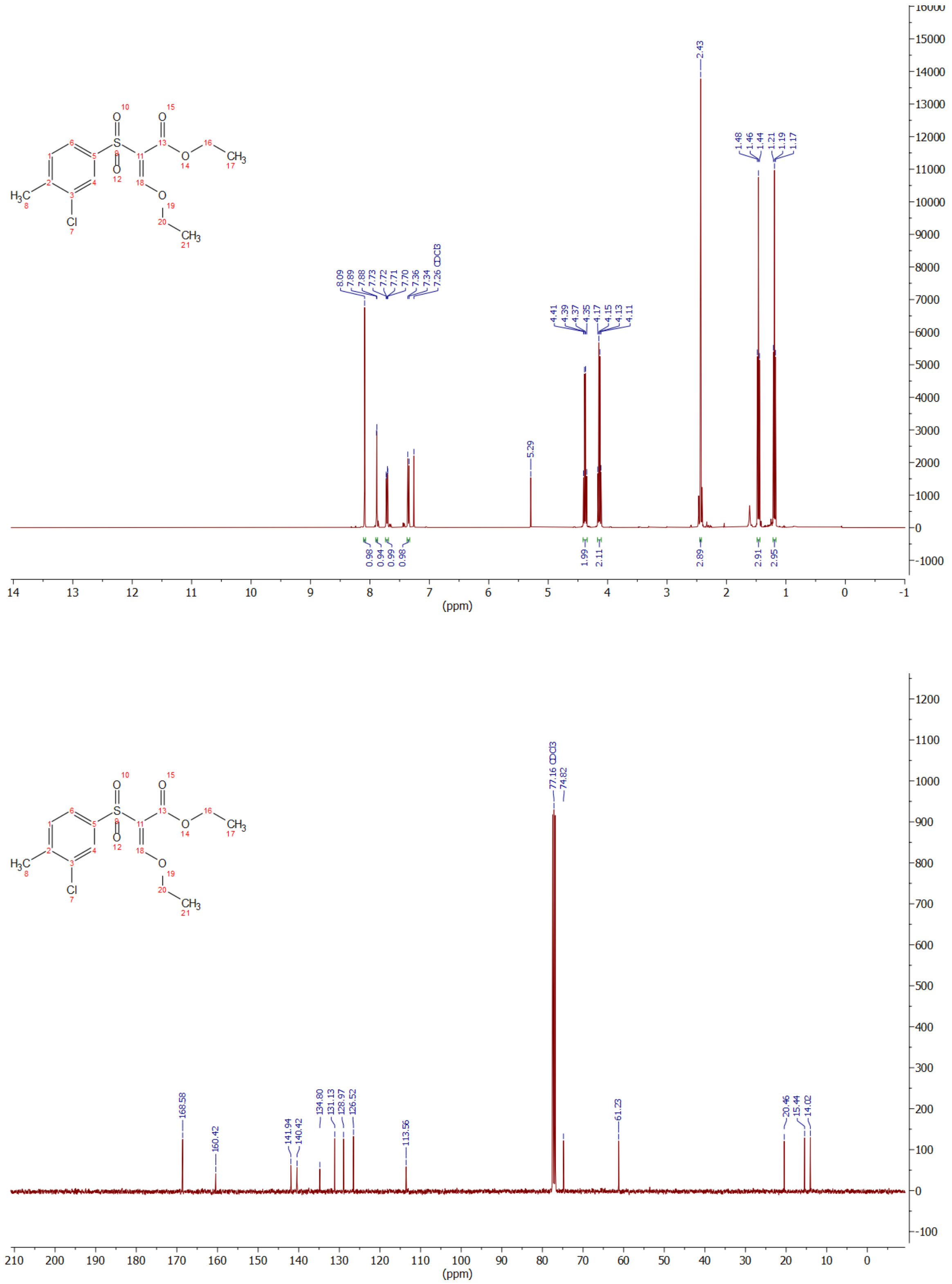

Figure S4. ^{1}H *(400 MHz) and* $^{13}C\{1H\}$ *(101 MHz) NMR spectra in* $CDCl_3$ *of* ***X6***

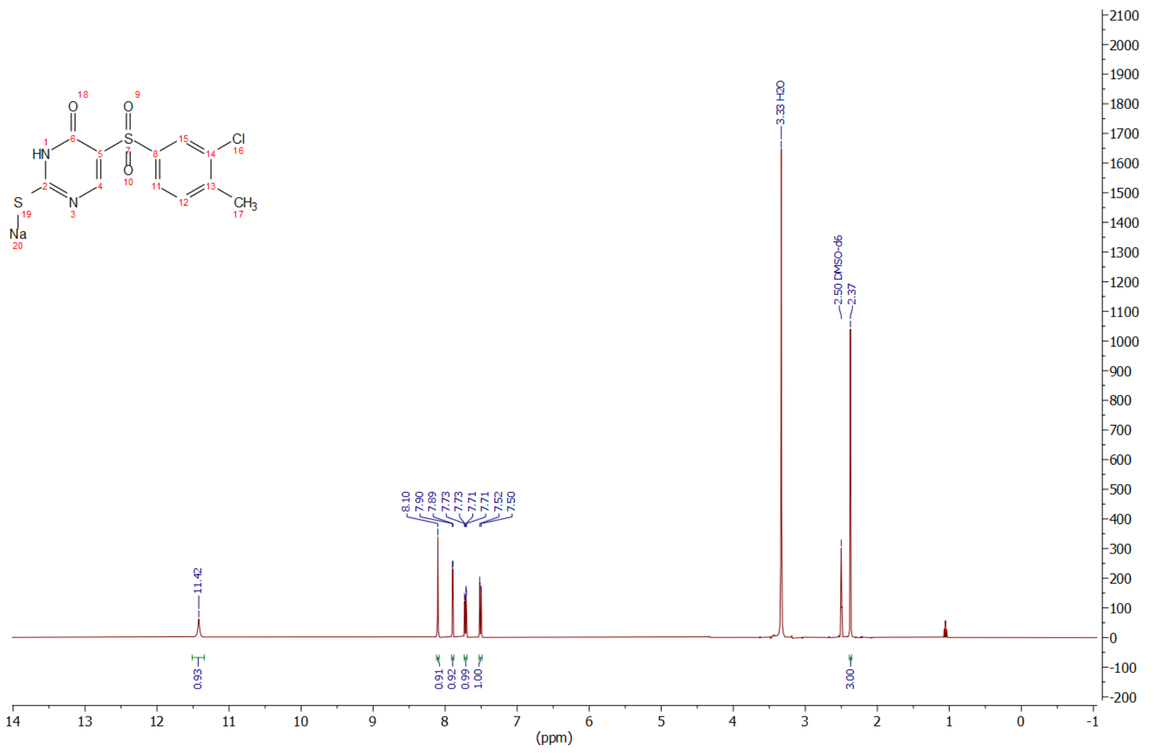

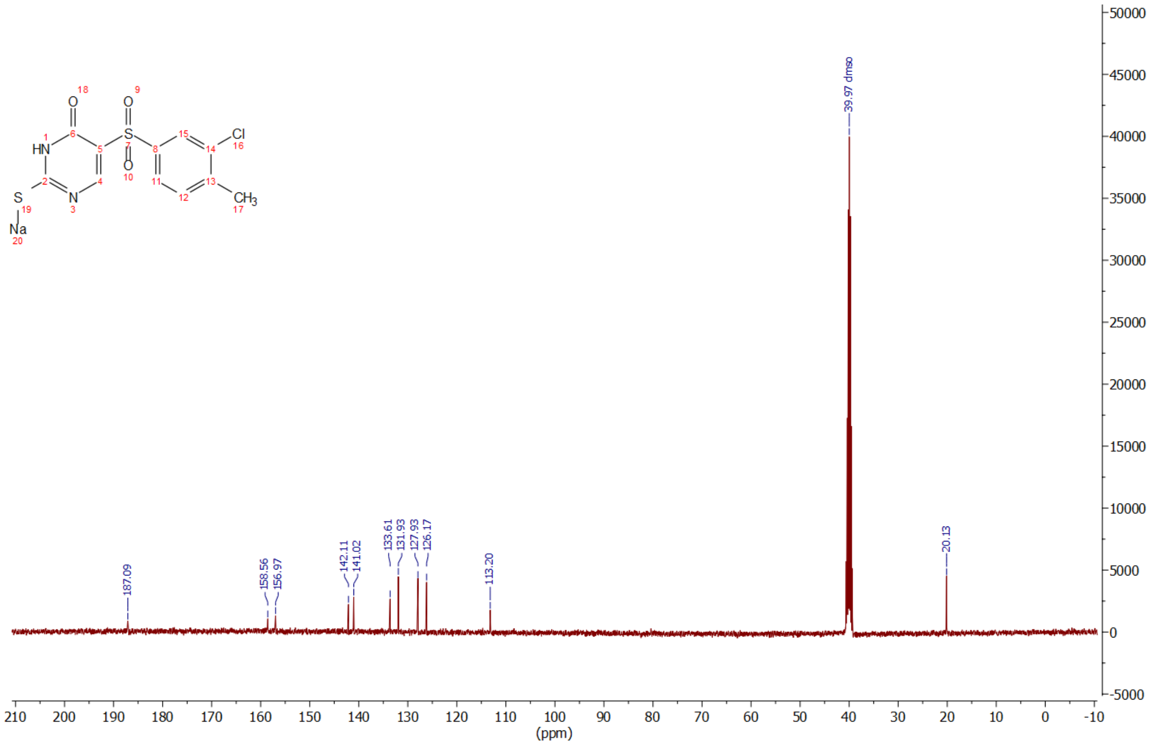

Figure S5. *^{1}H (400 MHz) and $^{13}C\{1H\}$ (101 MHz) NMR spectra in DMSO-d_6 of* ***X7***

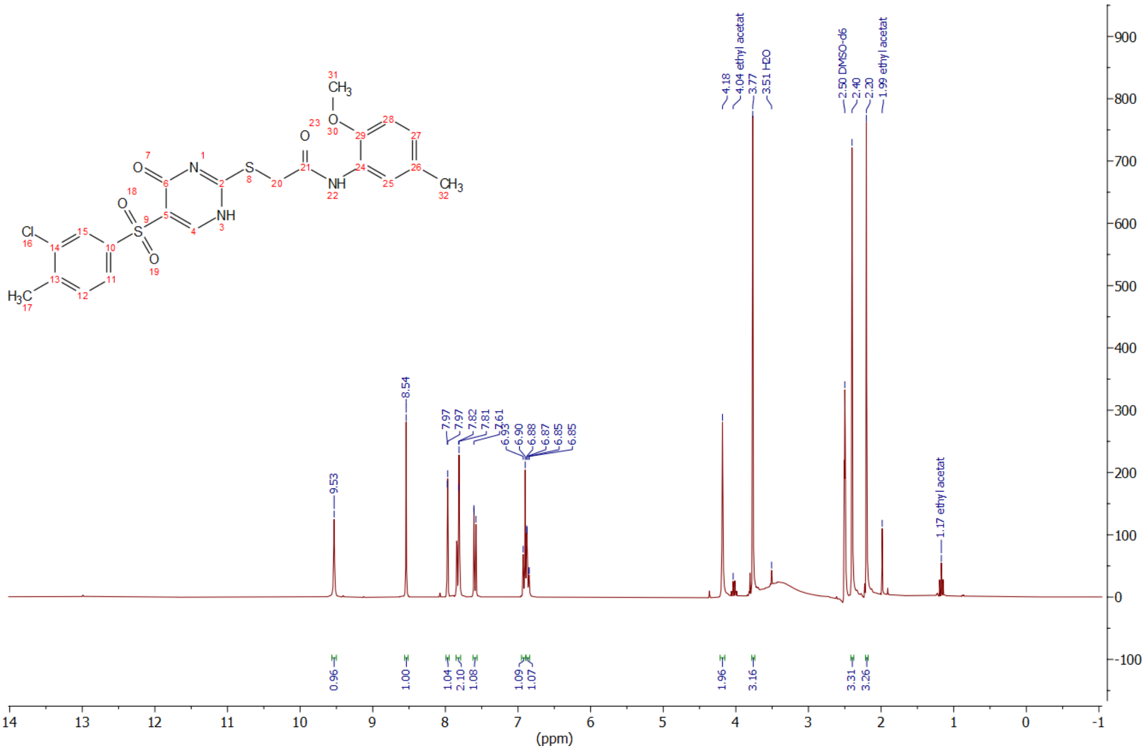

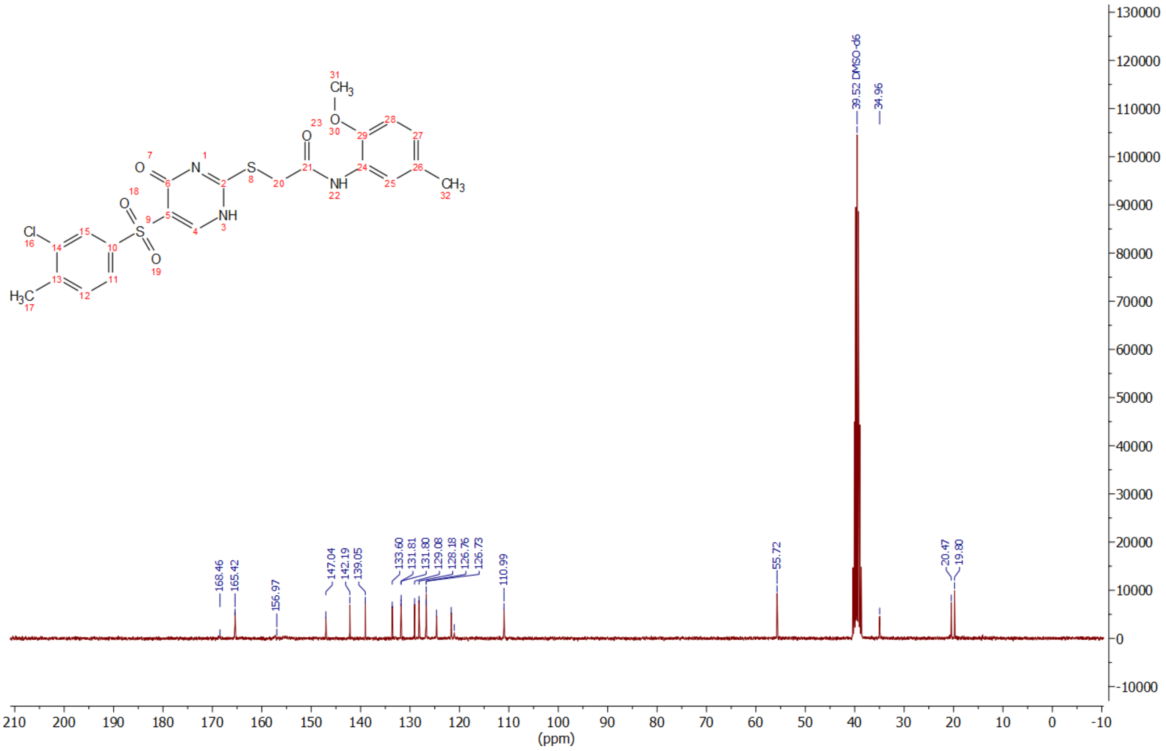

Figure S6. *^{1}H (300 MHz) and $^{13}C\{1H\}$ (75 MHz) NMR spectra in DMSO-d_6 of* ***compound 1***

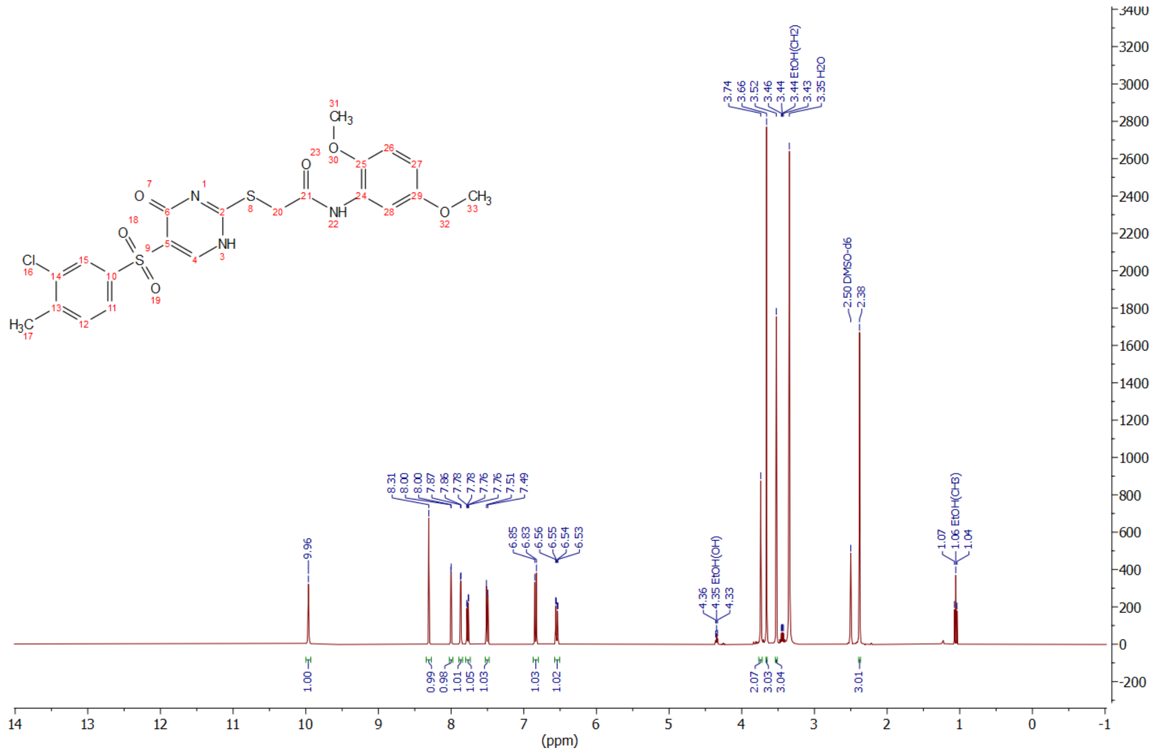

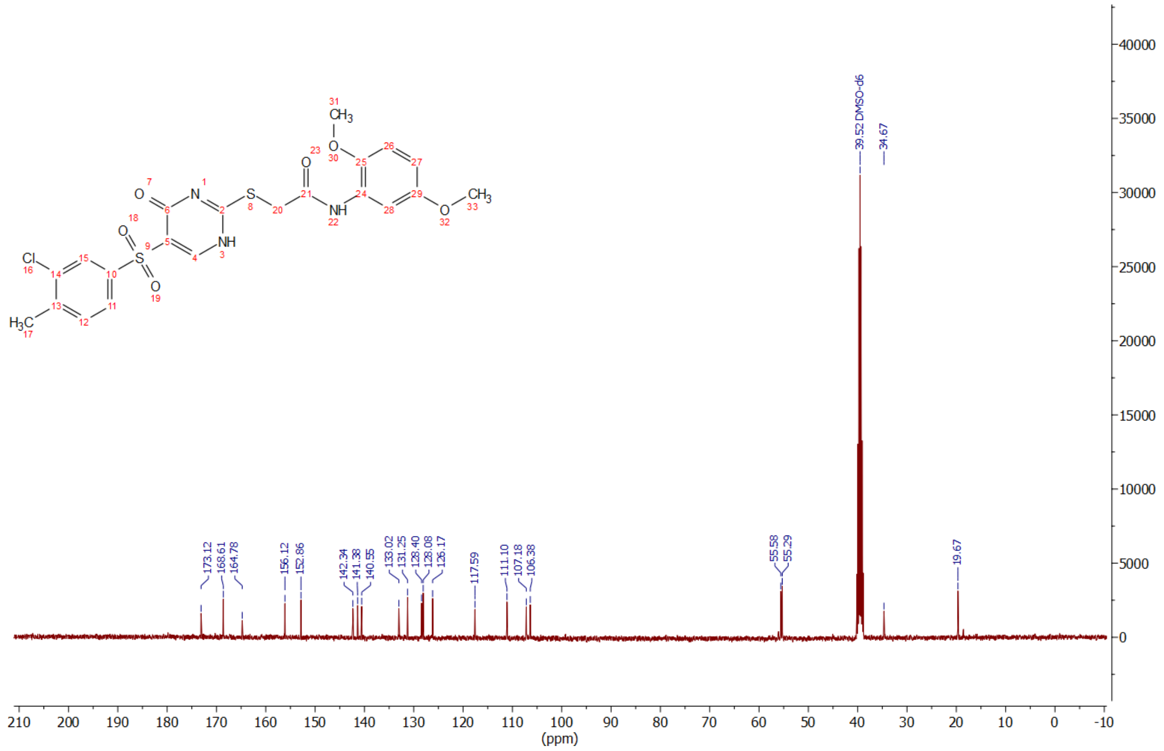

Figure S7. *^{1}H (400 MHz) and $^{13}C\{1H\}$ (101 MHz) NMR spectra in DMSO-d_6 of* ***compound 2***

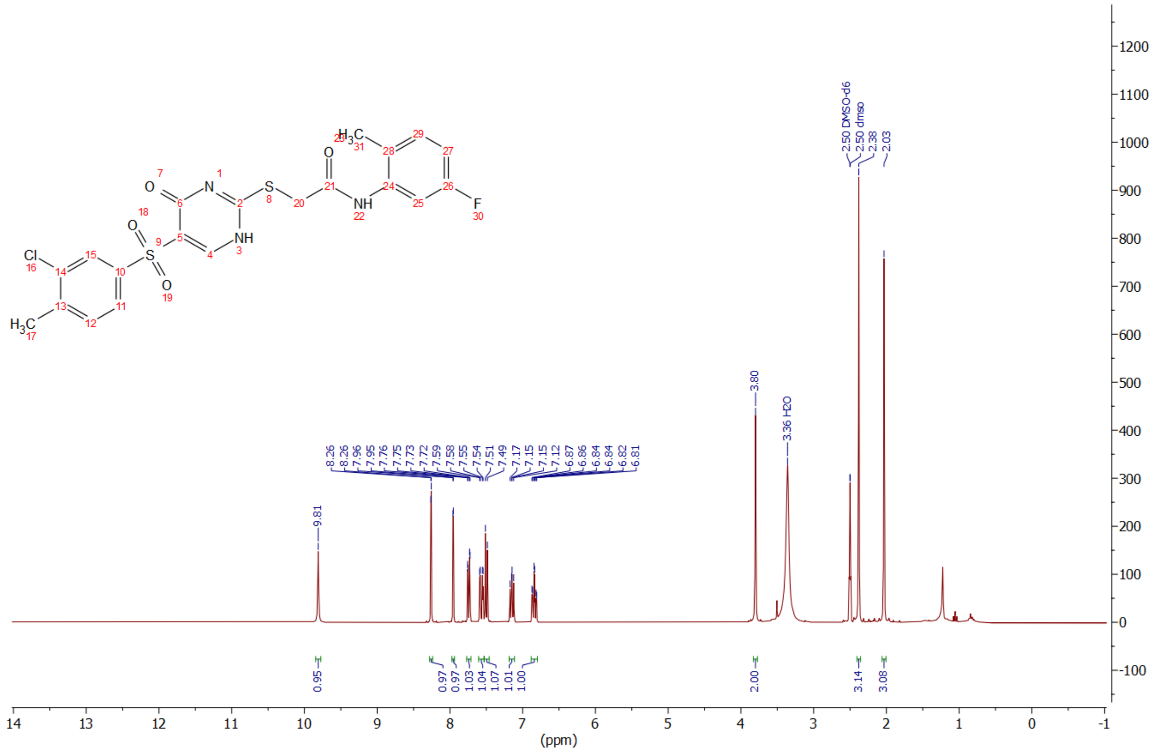

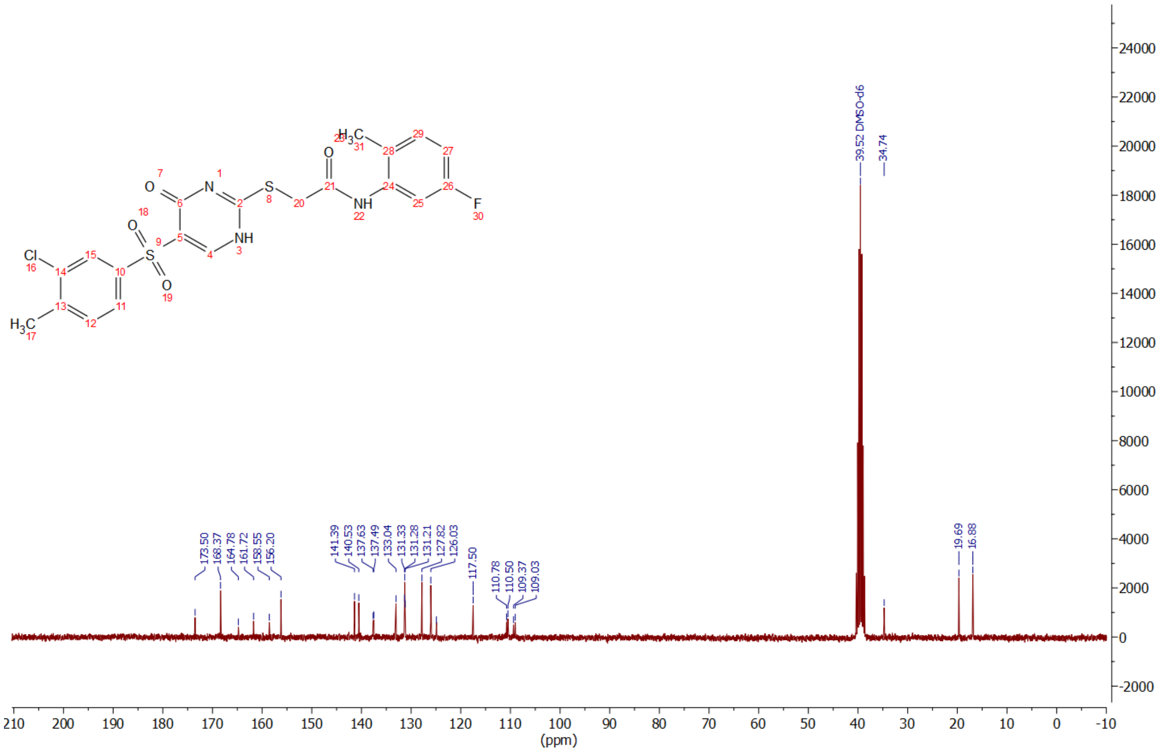

Figure S8. *^{1}H (300 MHz) and $^{13}C\{1H\}$ (75 MHz) NMR spectra in DMSO-d_6 of* ***compound 3***

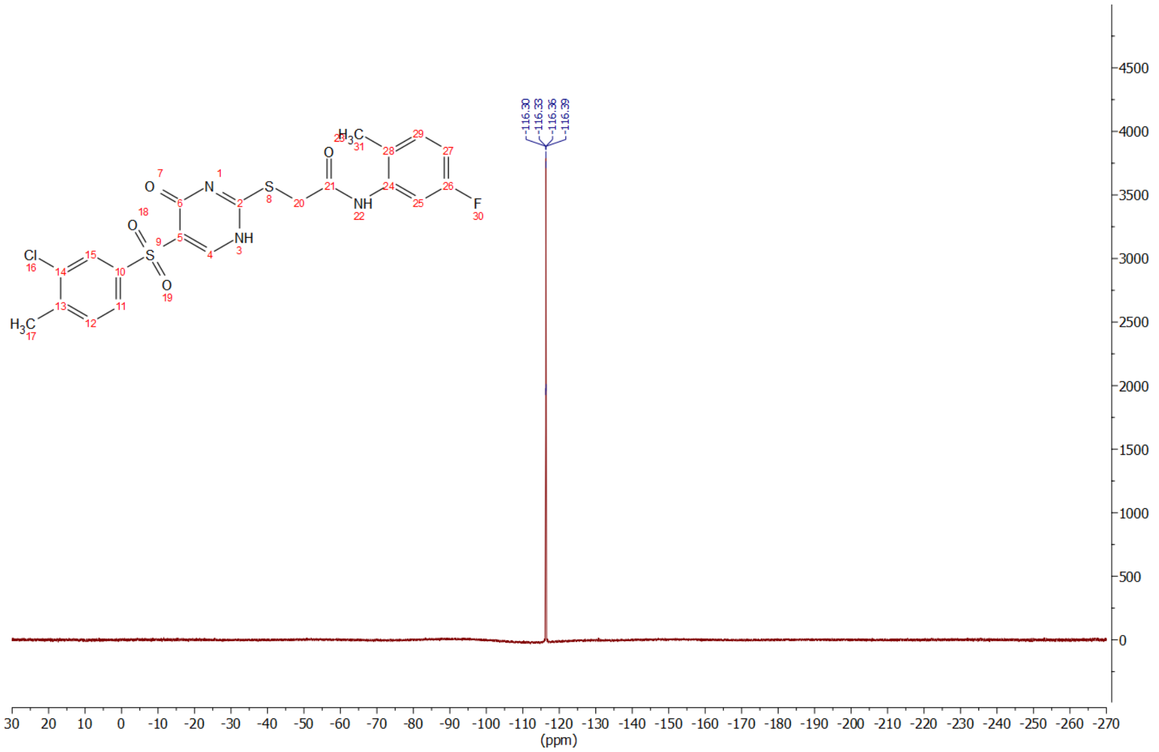

Figure S9. *^{19}F (282 MHz) NMR spectrum in DMSO-d$_6$ of* ***compound 3***

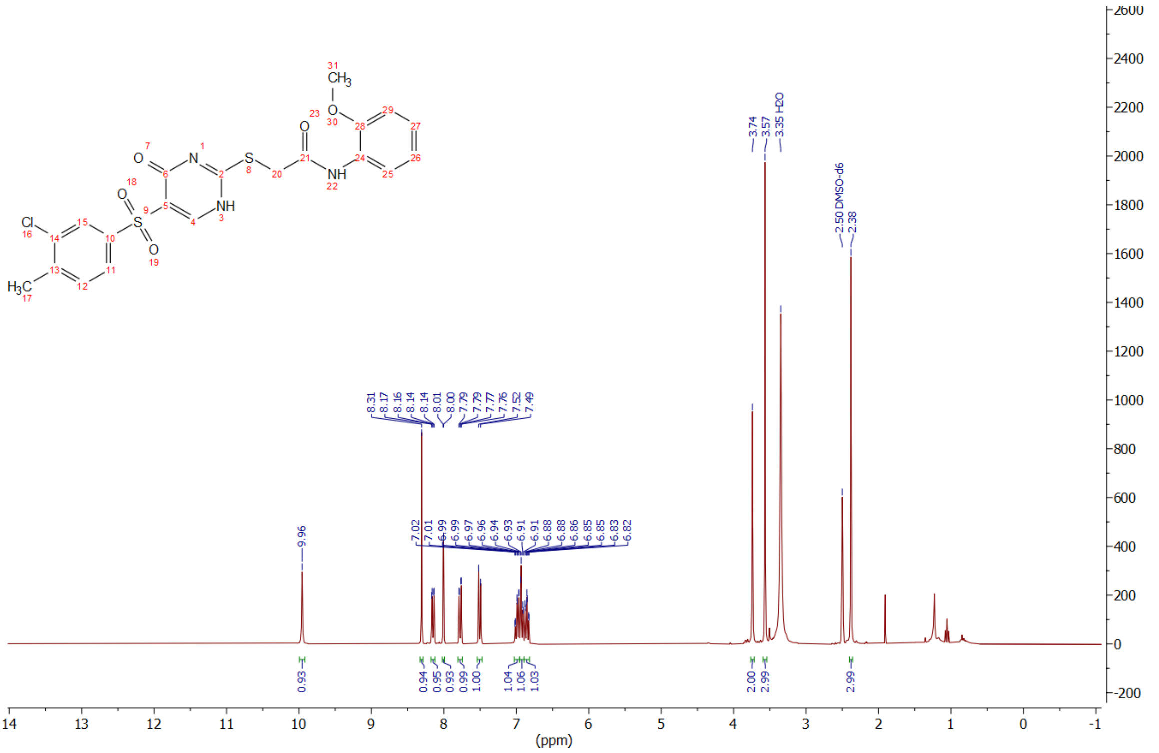

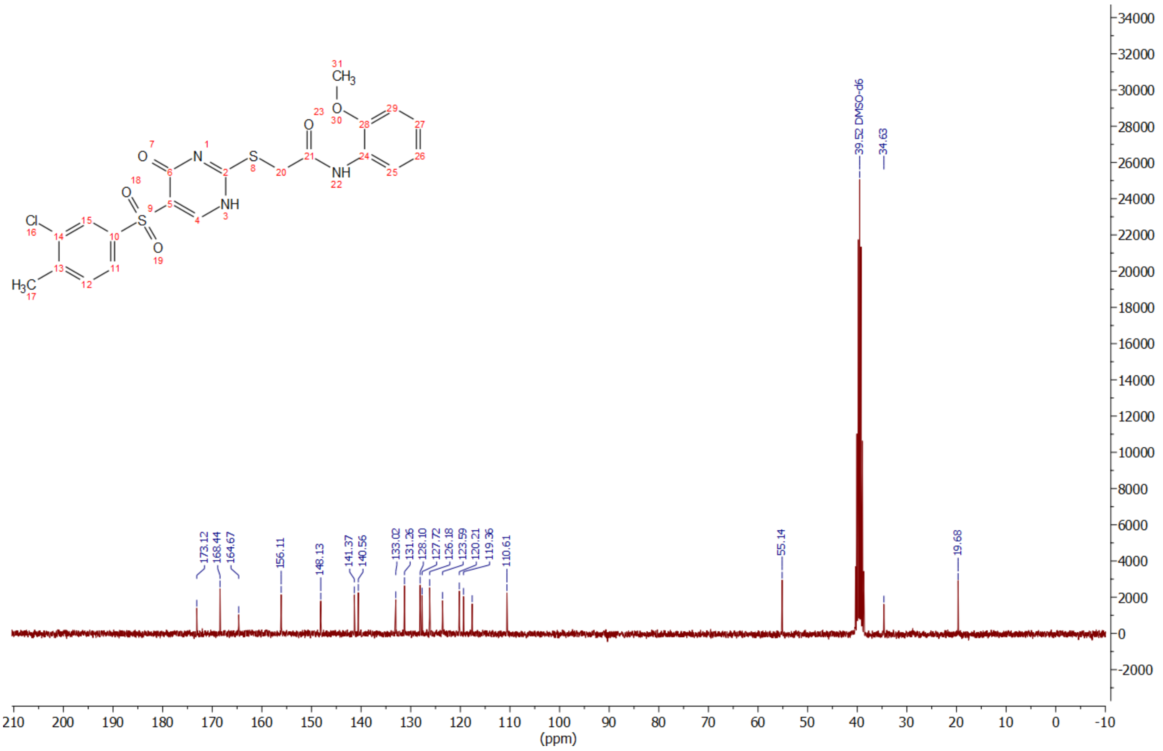

Figure S10. *^{1}H (300 MHz) and ^{13}C{1H} (75 MHz) NMR spectra in DMSO-d$_6$ of **compound 4***

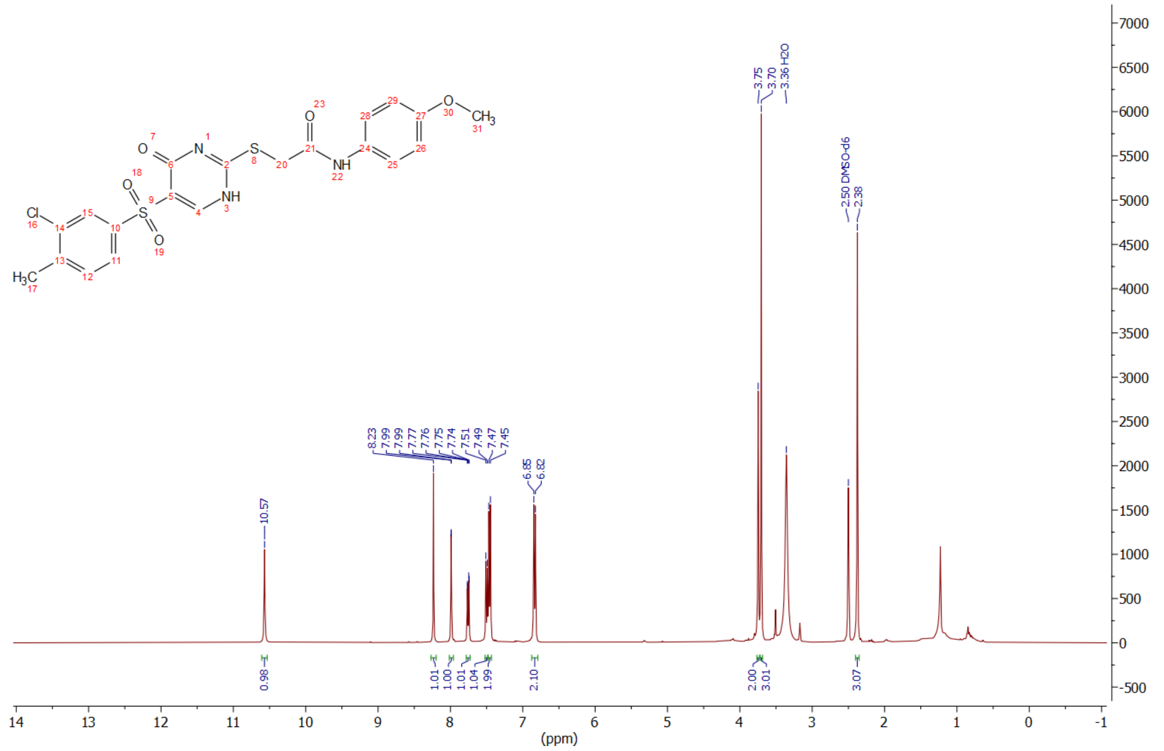

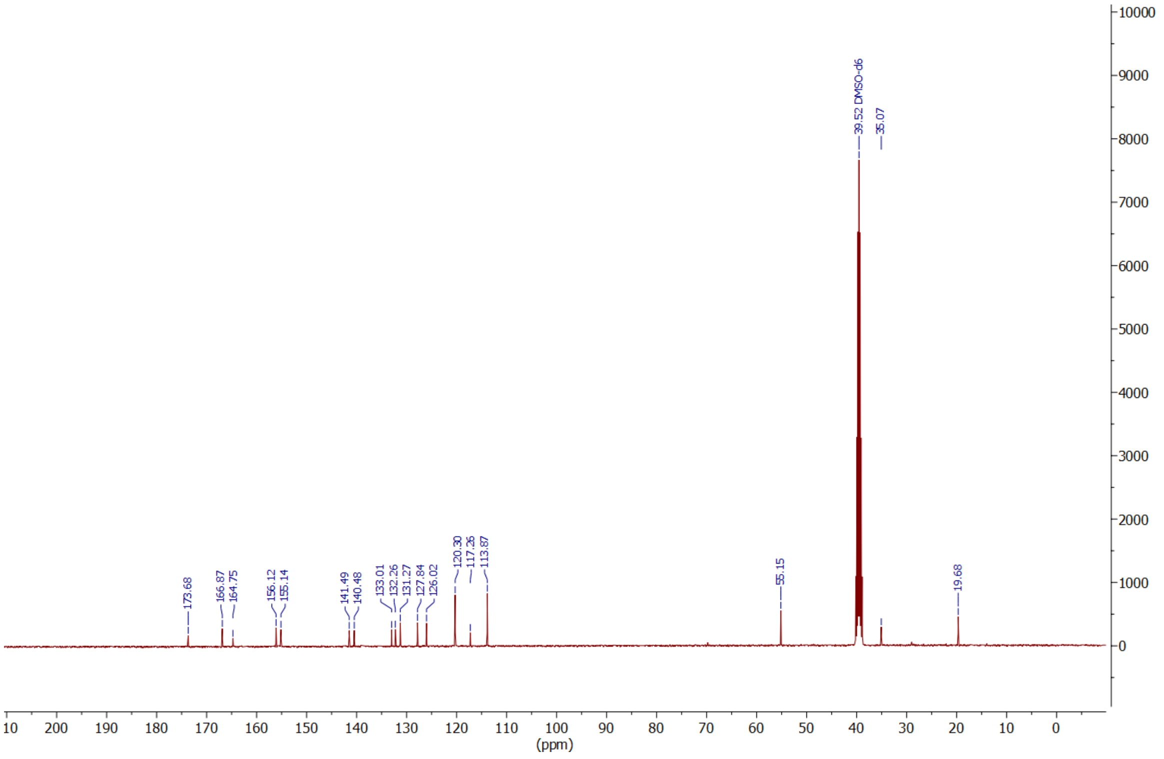

Figure S11. *^{1}H (400 MHz) and $^{13}C\{1H\}$ (101 MHz) NMR spectra in DMSO-d_6 of **compound 5***

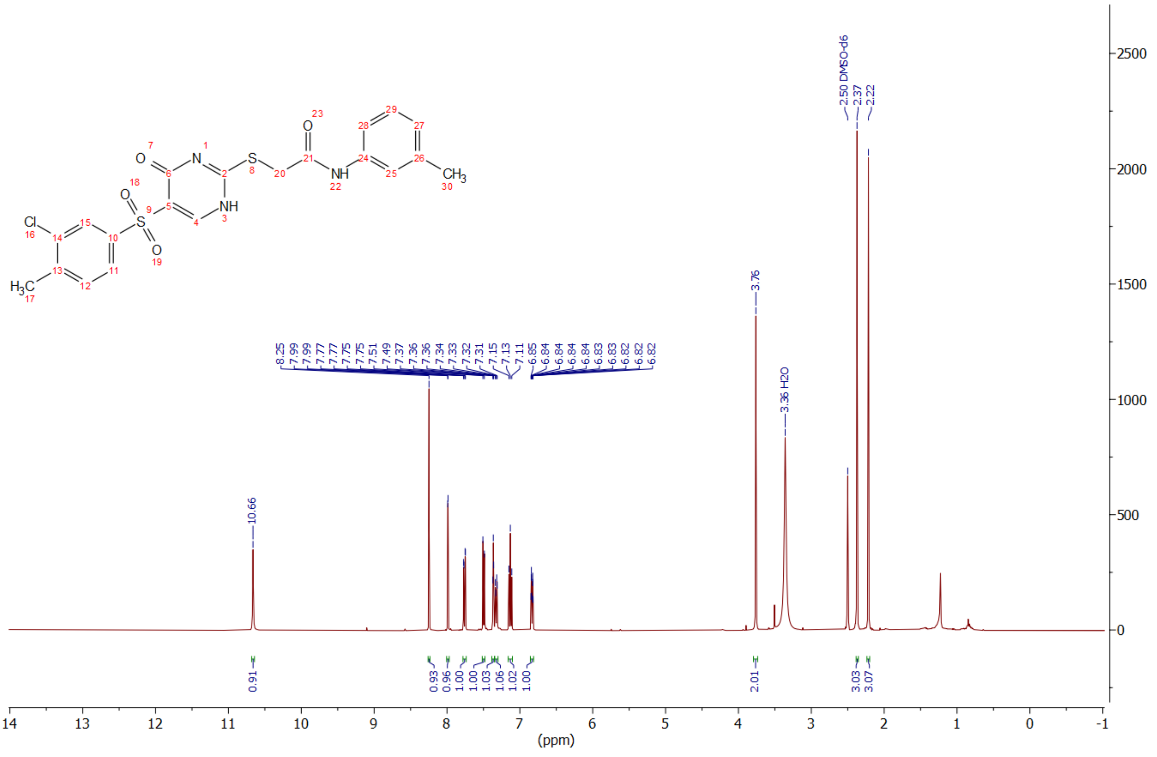

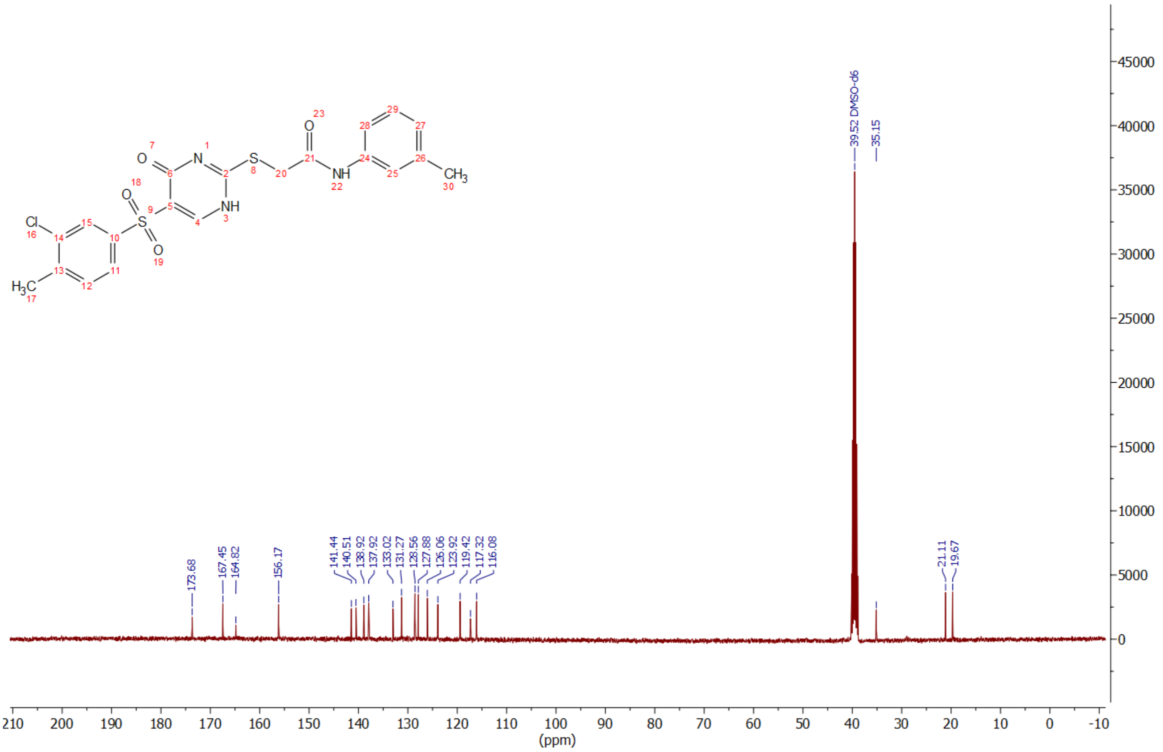

Figure S12. *^{1}H (400 MHz) and $^{13}C\{1H\}$ (101 MHz) NMR spectra in DMSO-d_6 of* ***compound 6***

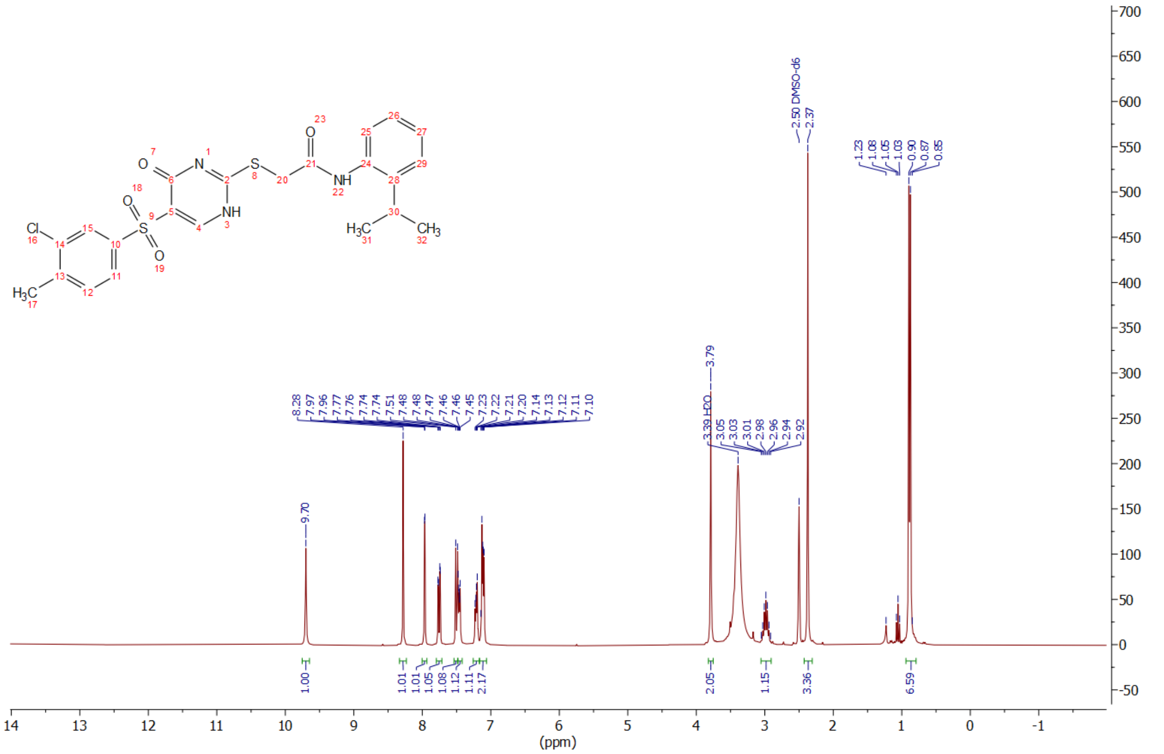

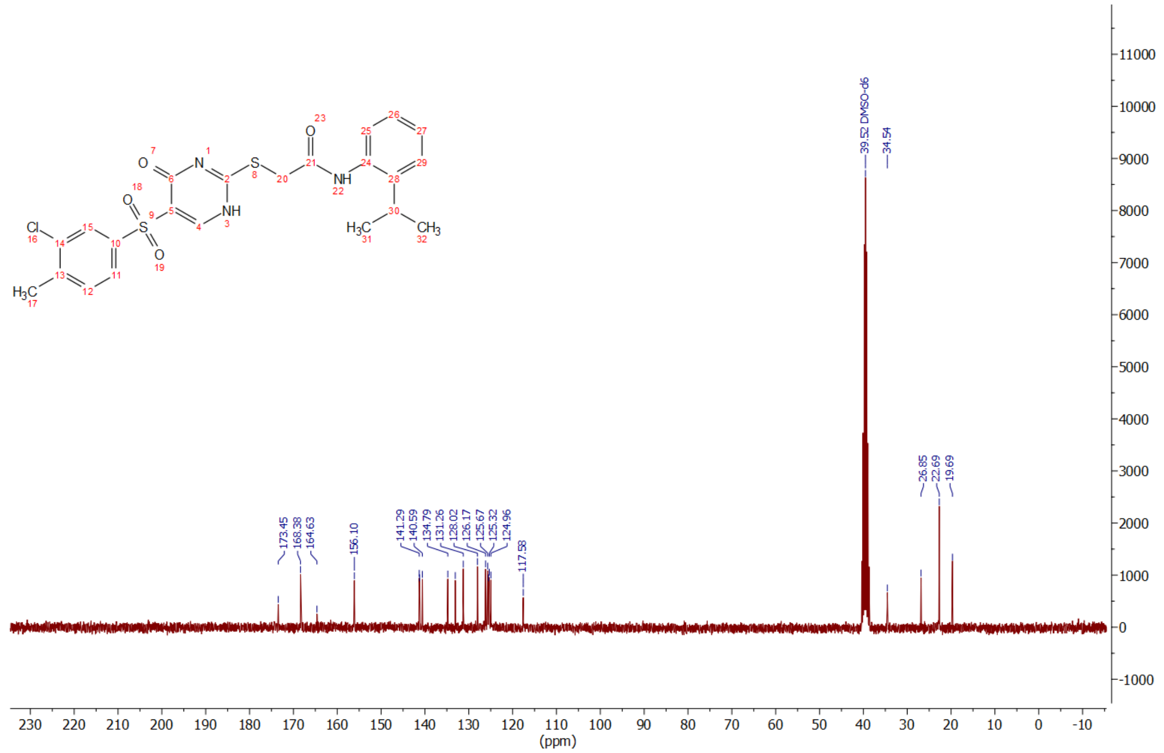

Figure S13. *^{1}H (300 MHz) and $^{13}C\{1H\}$ (75 MHz) NMR spectra in DMSO-d_6 of* ***compound 7***

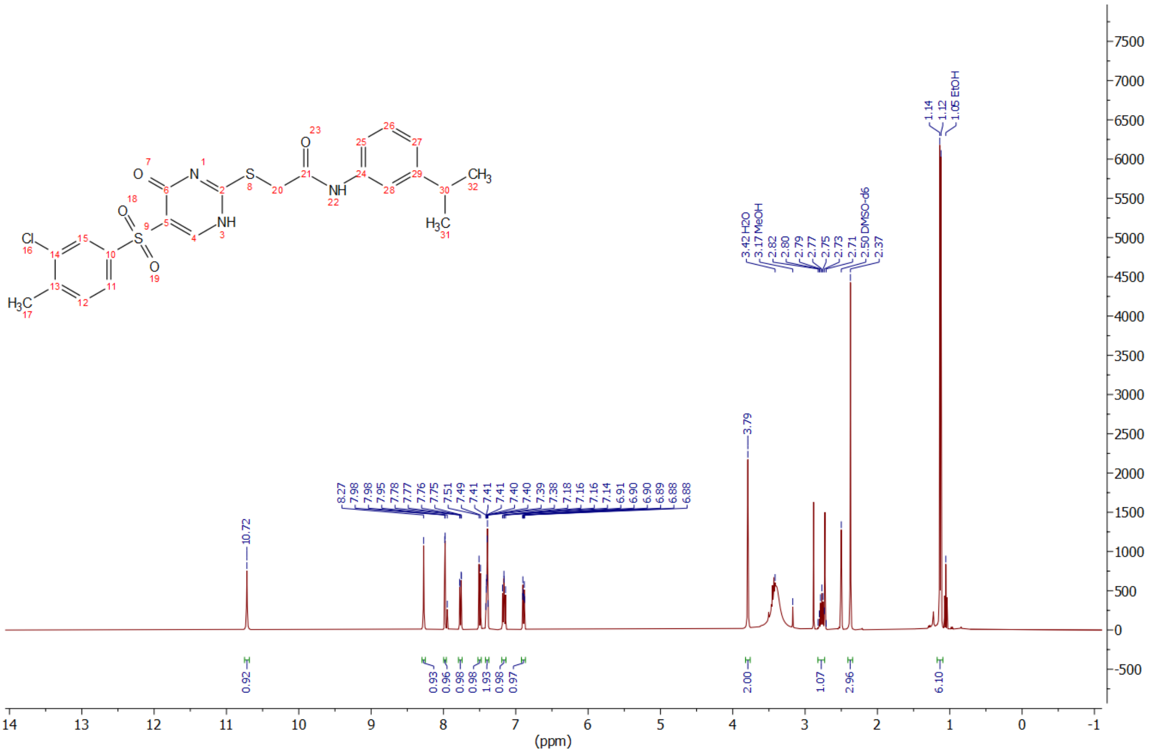

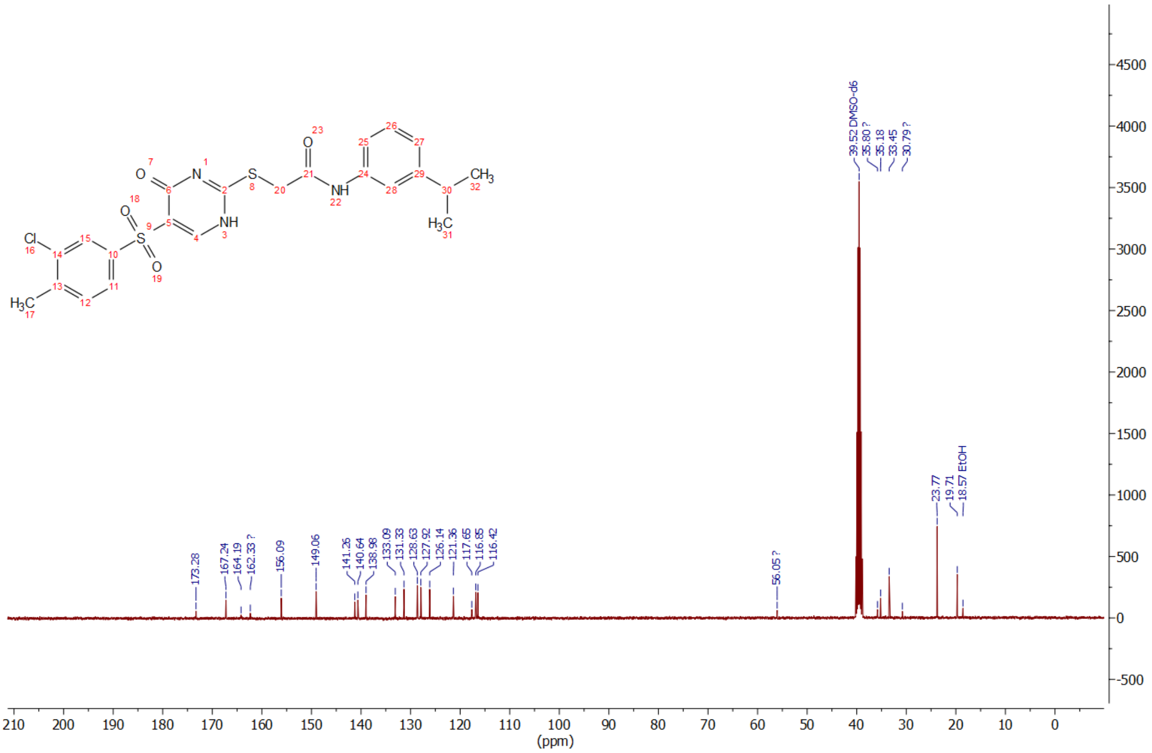

Figure S14. ^{1}H *(400 MHz) and* $^{13}C\{1H\}$ *(101 MHz) NMR spectra in DMSO-*d_6 *of* ***compound 8***

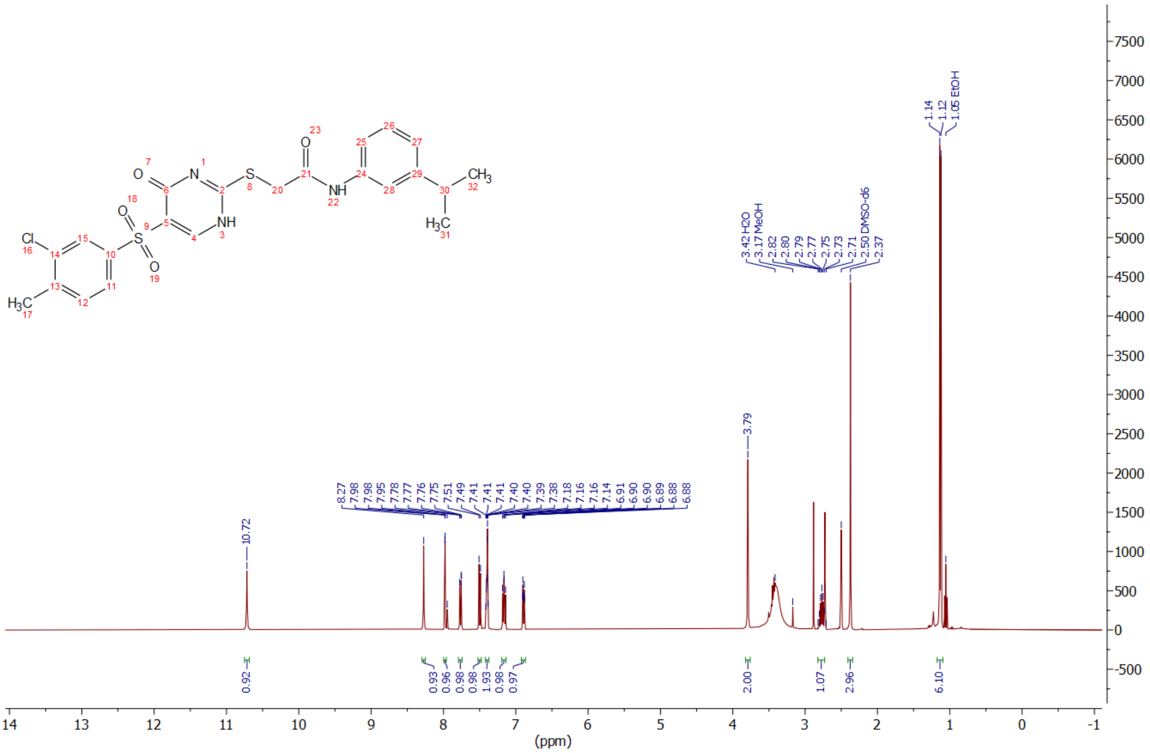

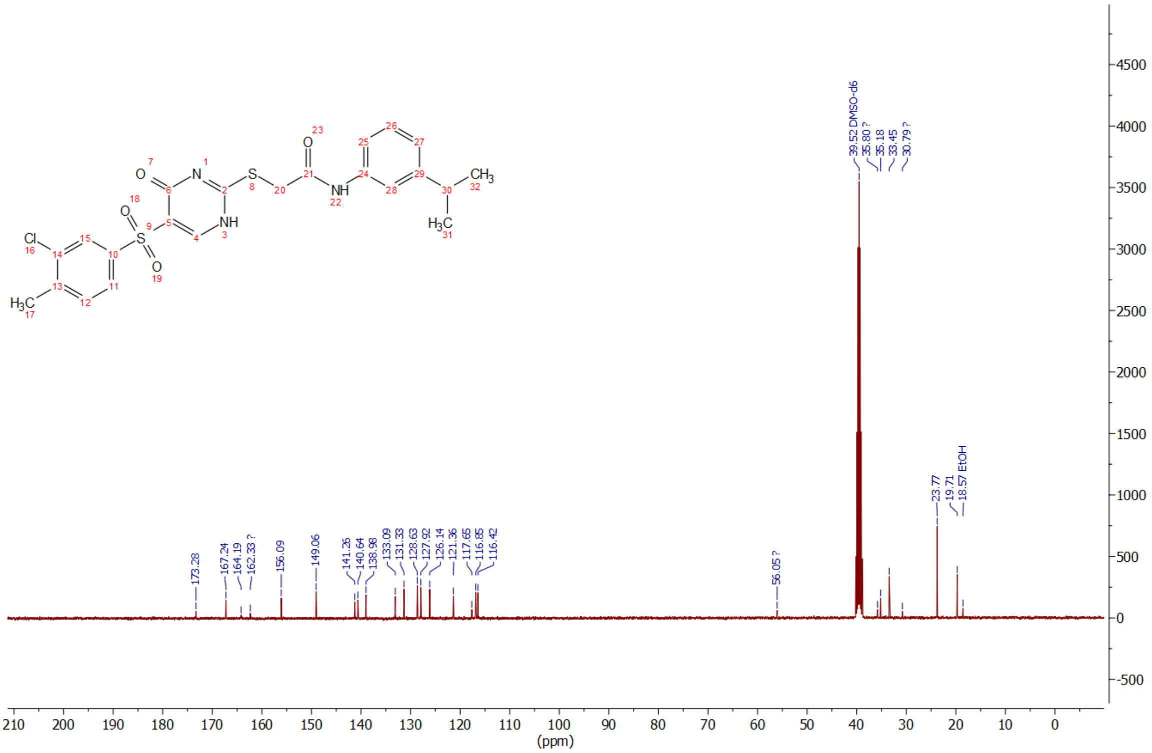

Figure S15. *^{1}H (400 MHz) and $^{13}C\{1H\}$ (101 MHz) NMR spectra in DMSO-d_6 of* ***compound 8***

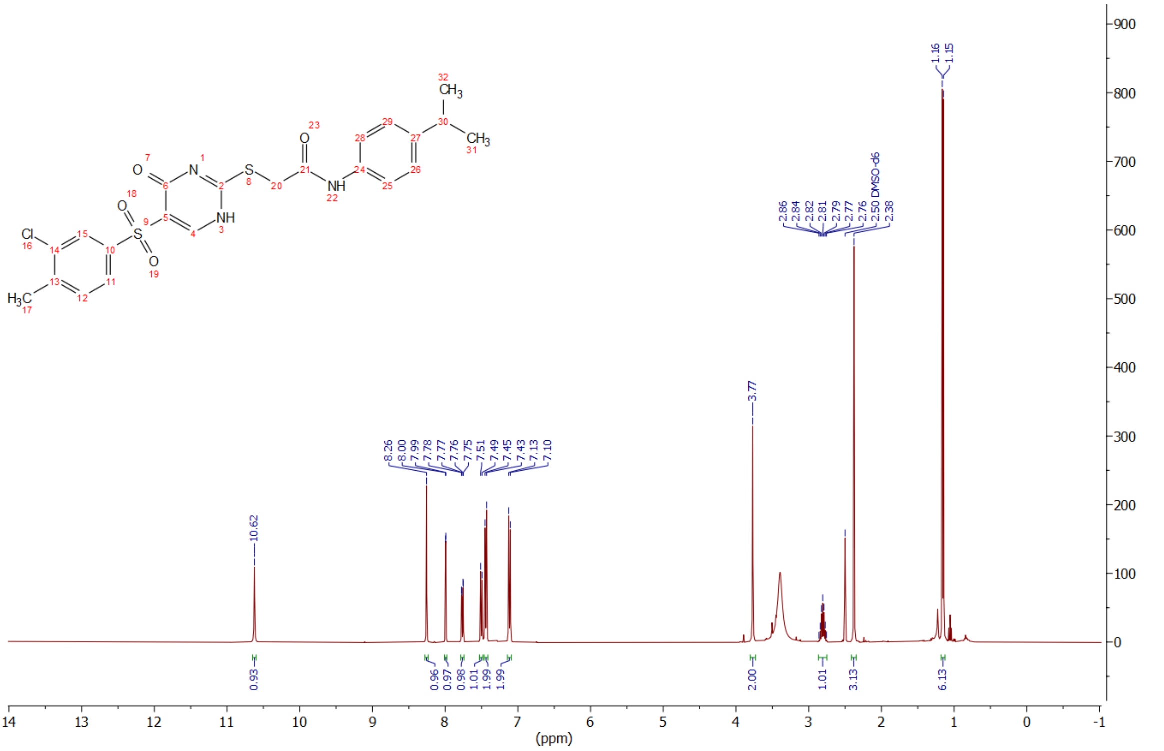

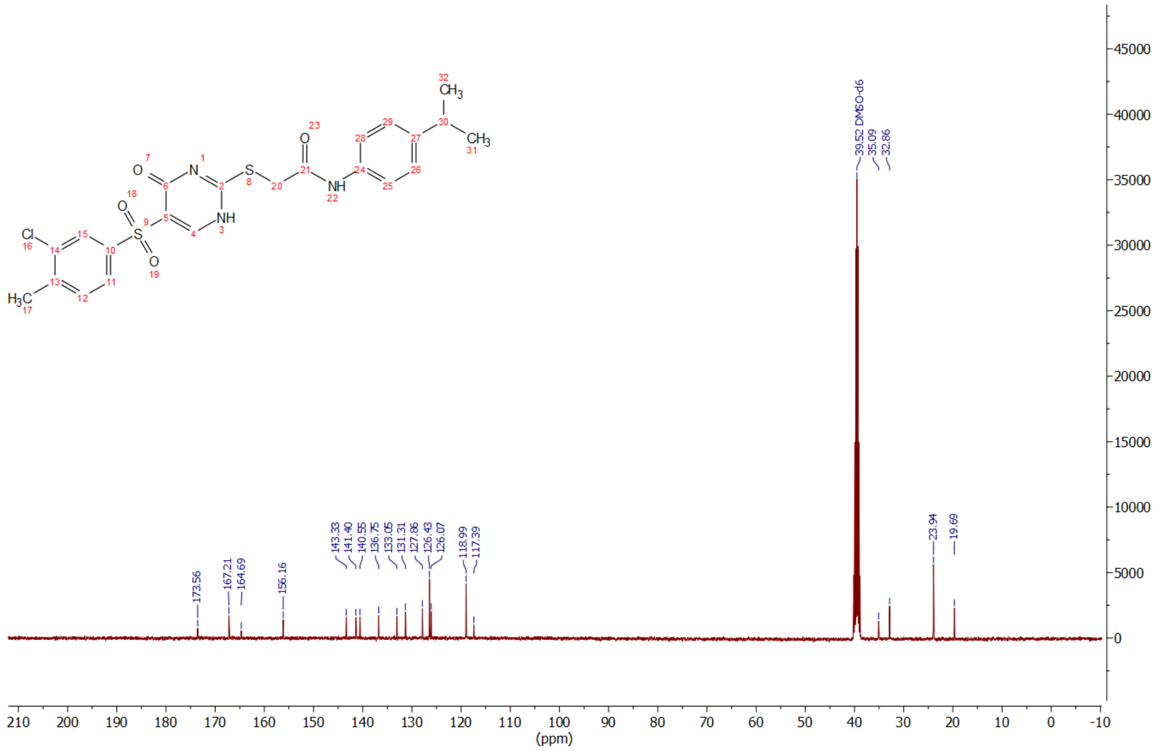

Figure S16. *^{1}H (400 MHz) and $^{13}C\{1H\}$ (101 MHz) NMR spectra in DMSO-d_6 of* ***compound 9***

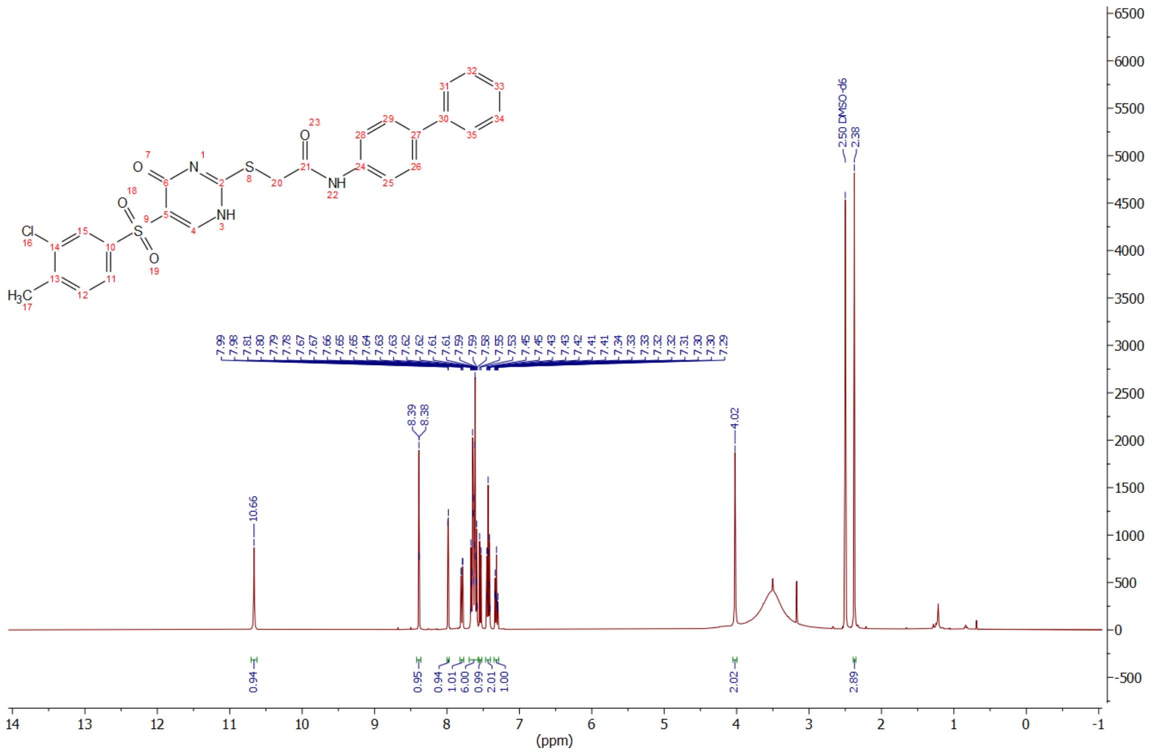

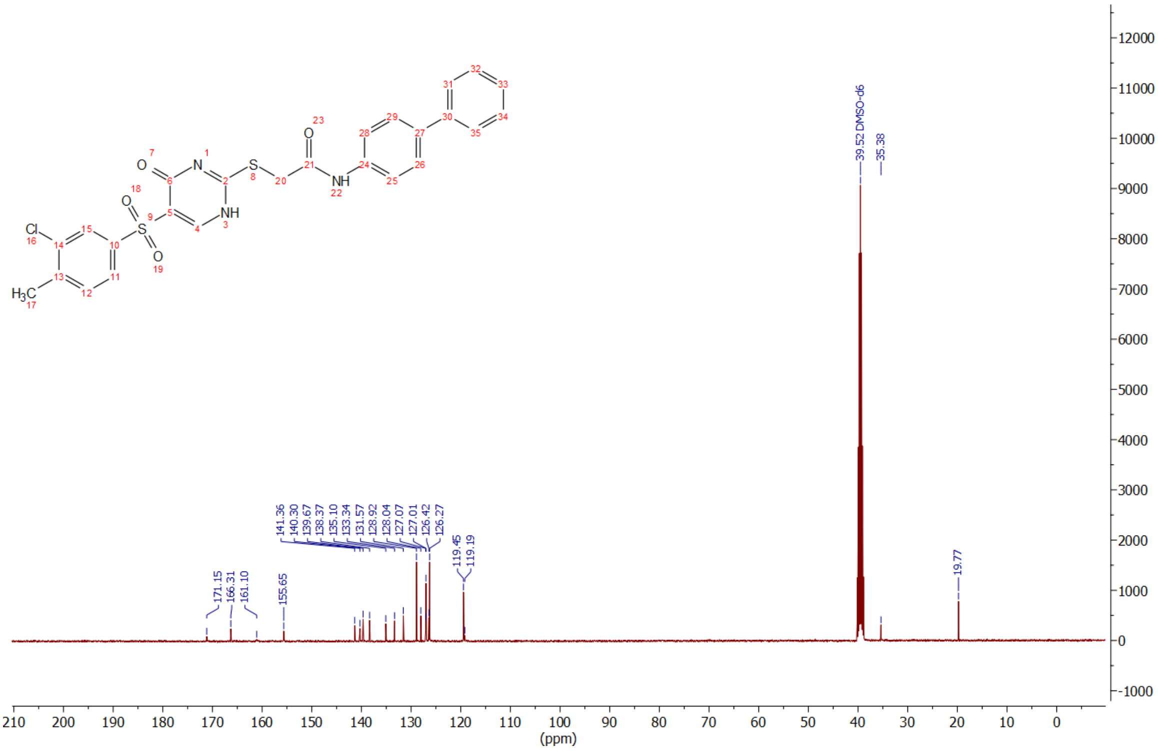

Figure S17. ^{1}H *(400 MHz) and* $^{13}C\{1H\}$ *(101 MHz) NMR spectra in DMSO-*d_6 *of* ***compound 10***

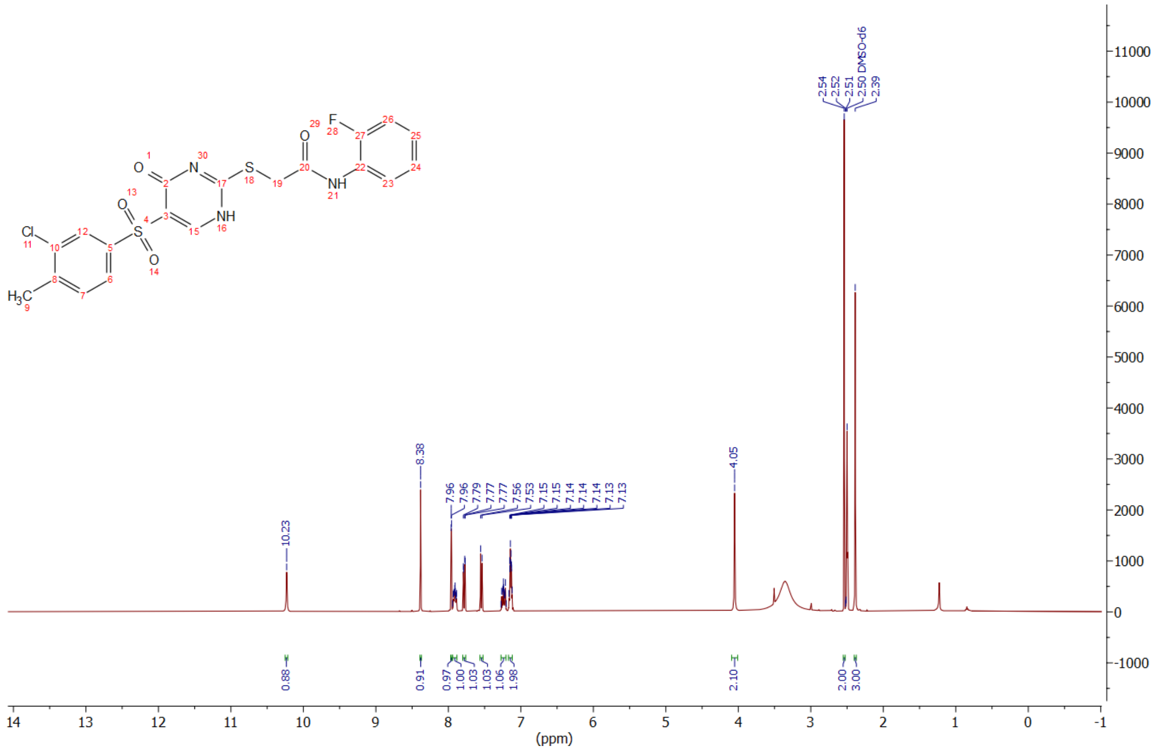

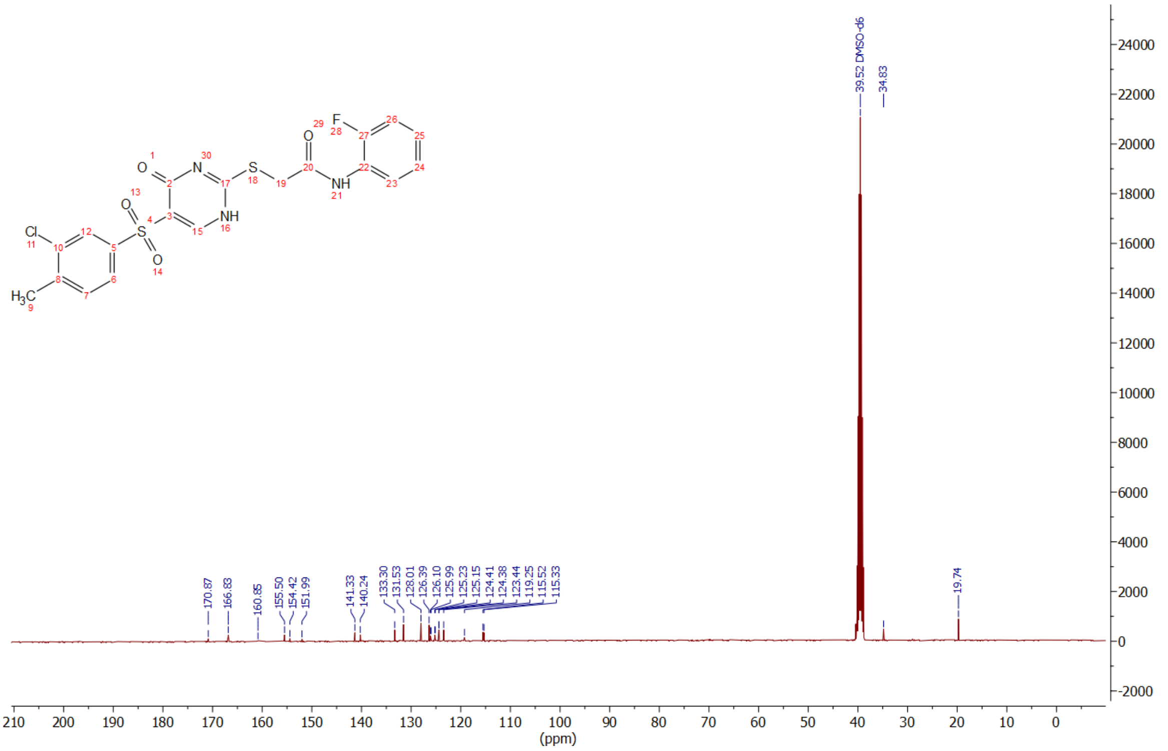

Figure S18. *^{1}H (400 MHz) and ^{13}C{1H} (101 MHz) NMR spectra in DMSO-d_6 of* ***compound 11***

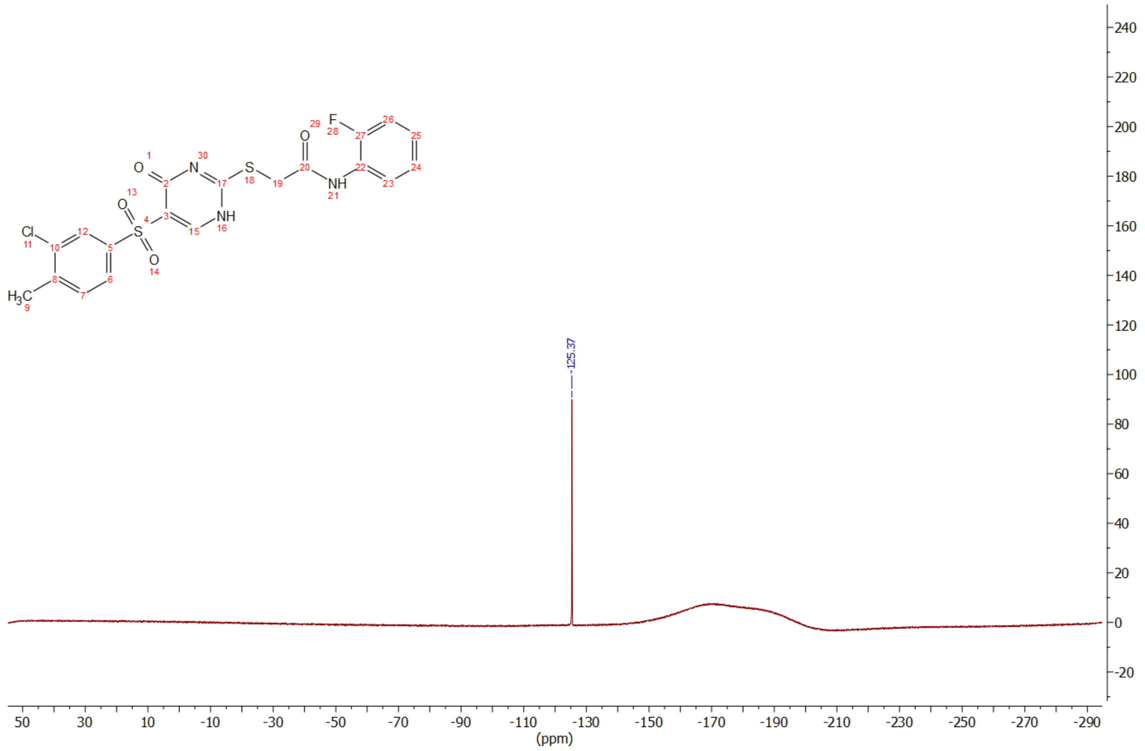

Figure S19. ^{19}*F (377 MHz) NMR spectrum in DMSO-d$_6$ of* ***compound 11***

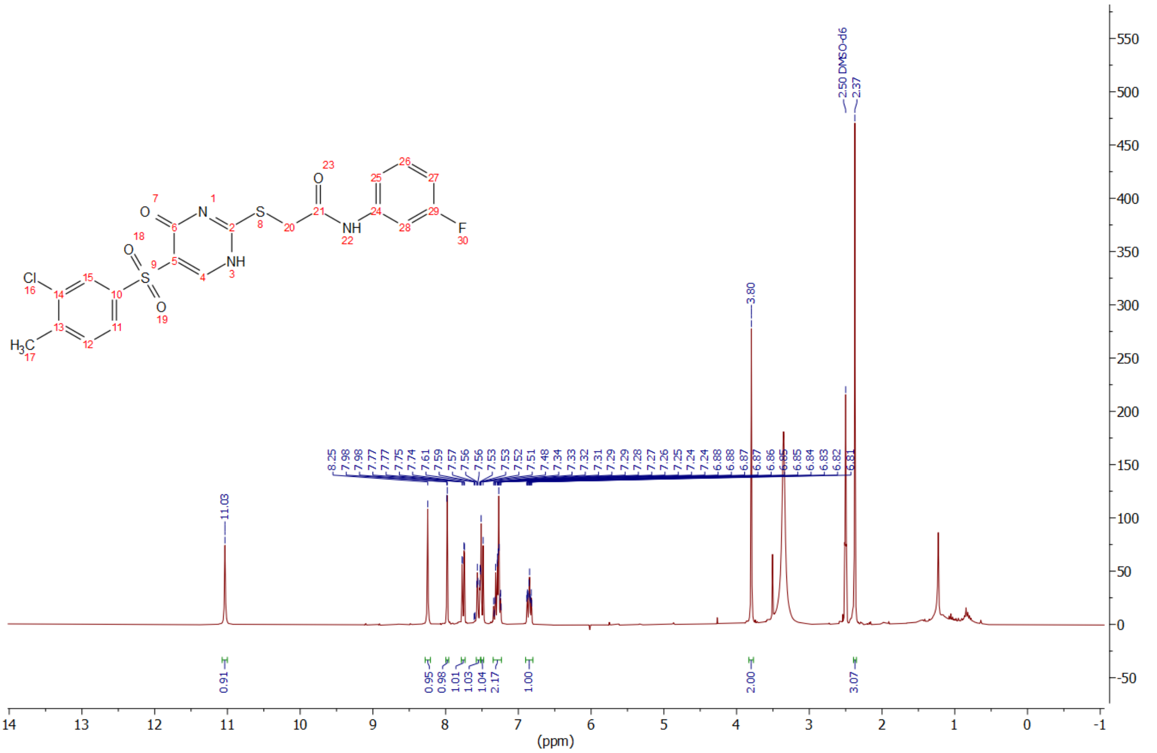

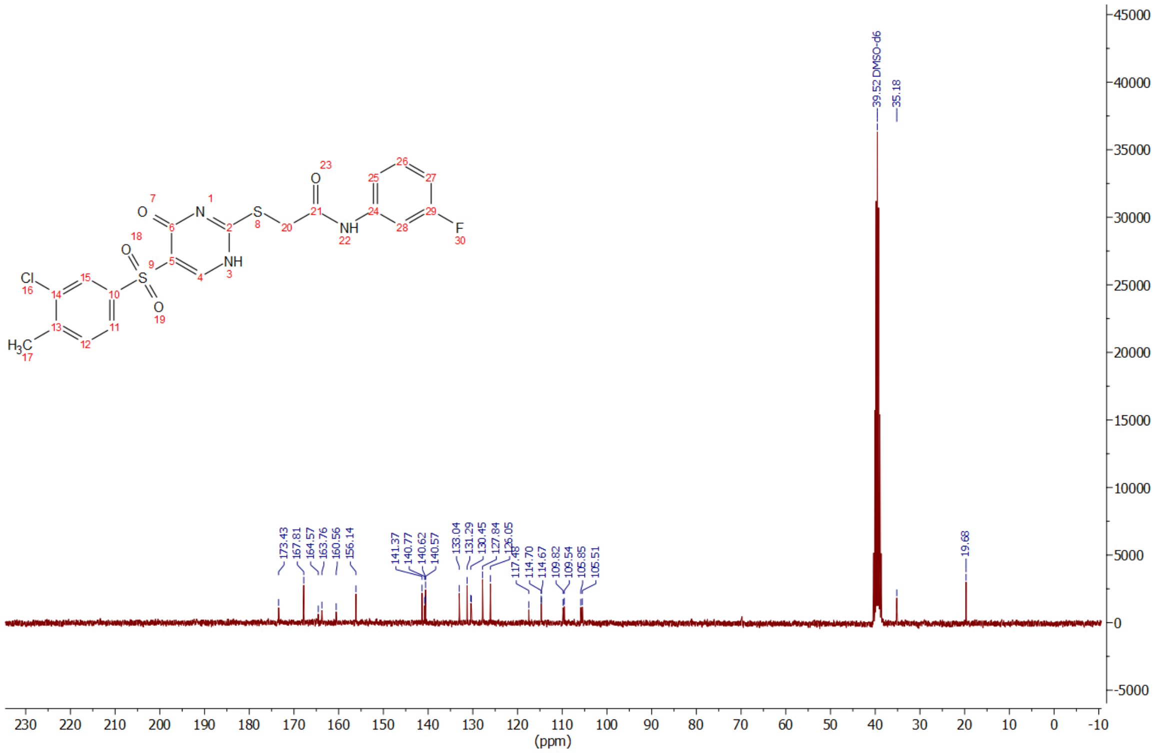

Figure S20. *^{1}H (300 MHz) and $^{13}C\{1H\}$ (75 MHz) NMR spectra in DMSO-d_6 of* ***compound 12***

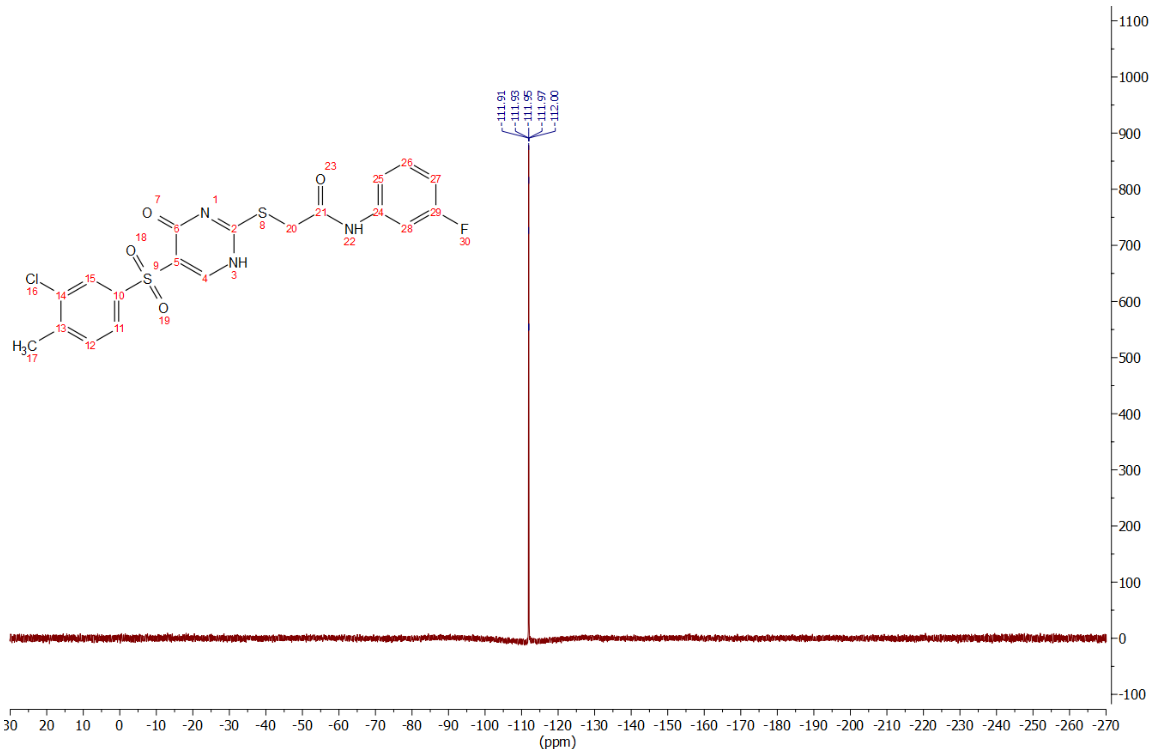

Figure S21. *^{19}F (282 MHz) NMR spectrum in DMSO-d_6 of* ***compound 12***

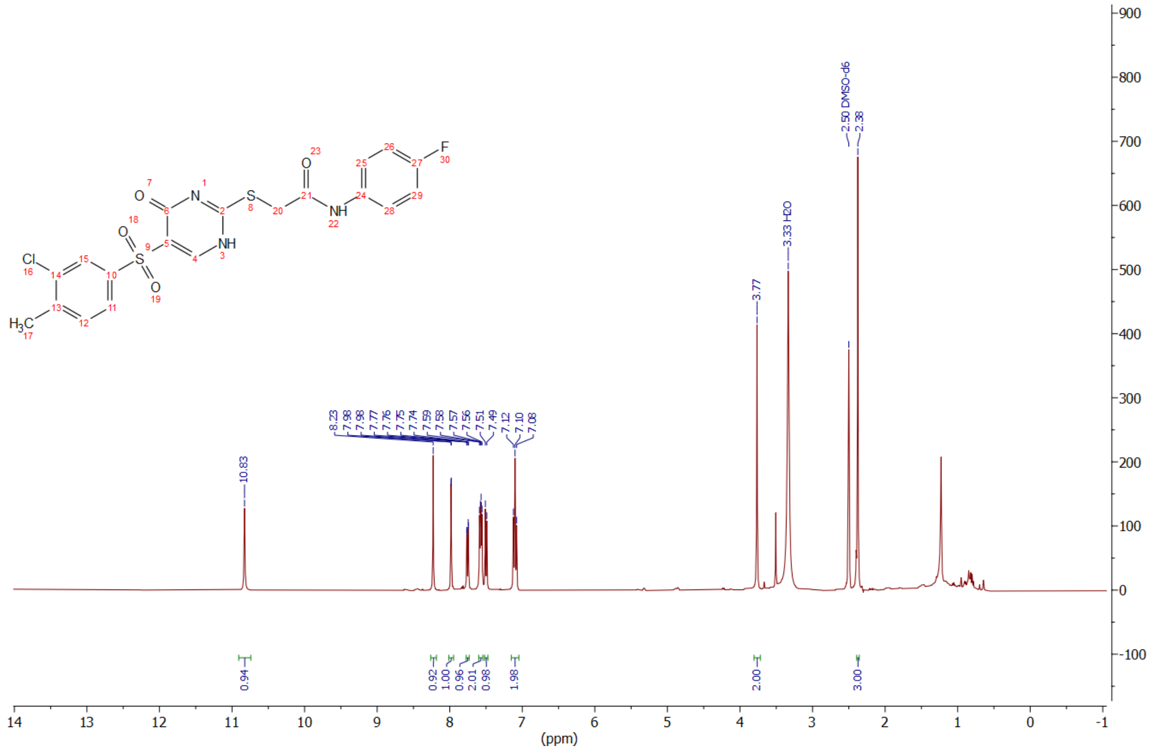

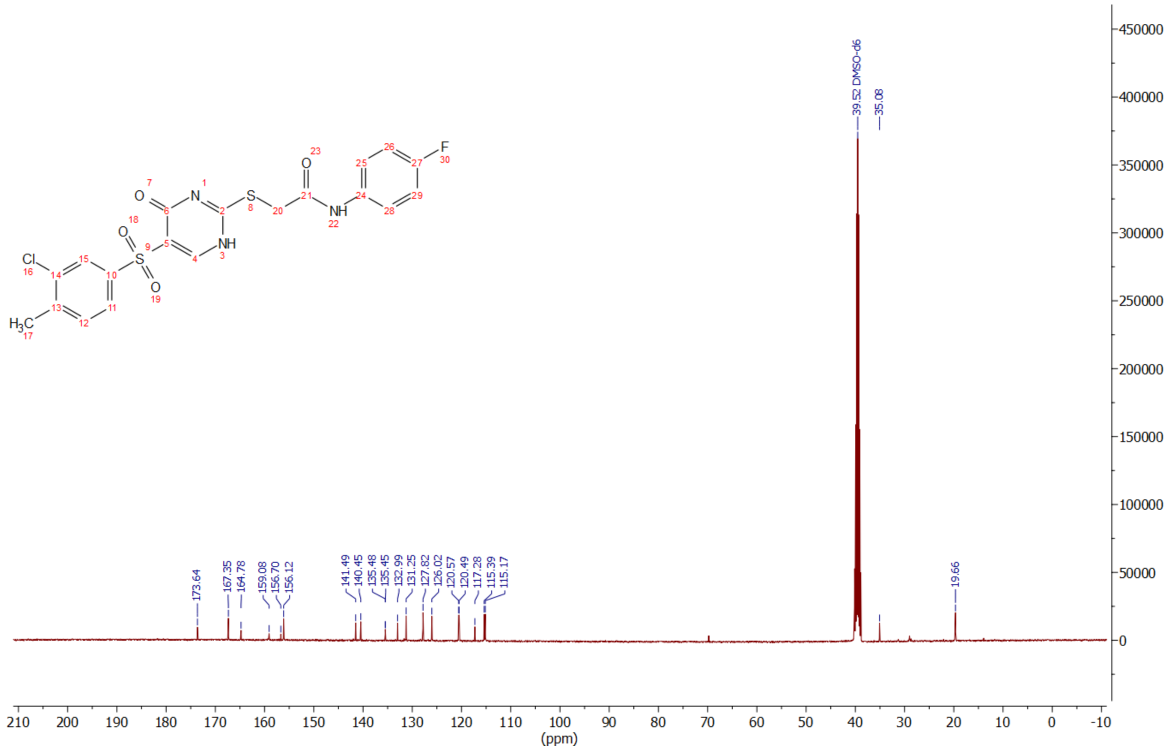

Figure S22. *^{1}H (400 MHz) and ^{13}C{1H} (101 MHz) NMR spectra in DMSO-d6 of* ***compound 13***

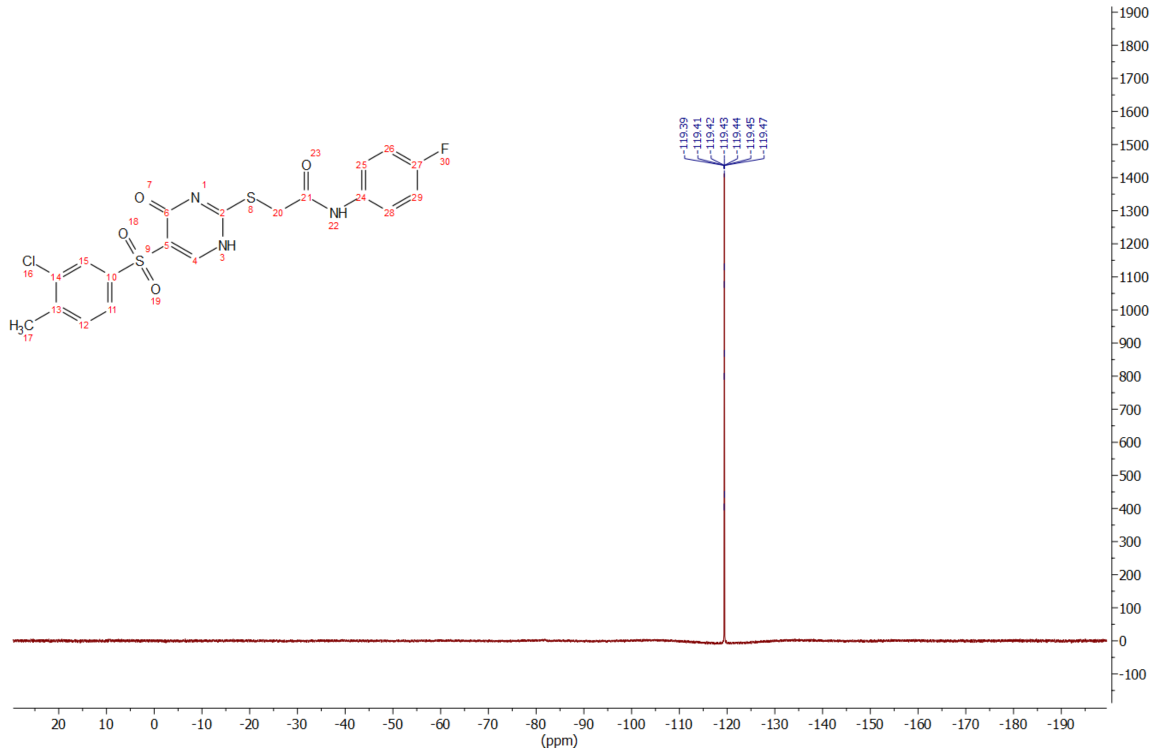

Figure S23. *^{19}F (376 MHz) NMR spectrum in DMSO-d_6 of* ***compound 13***

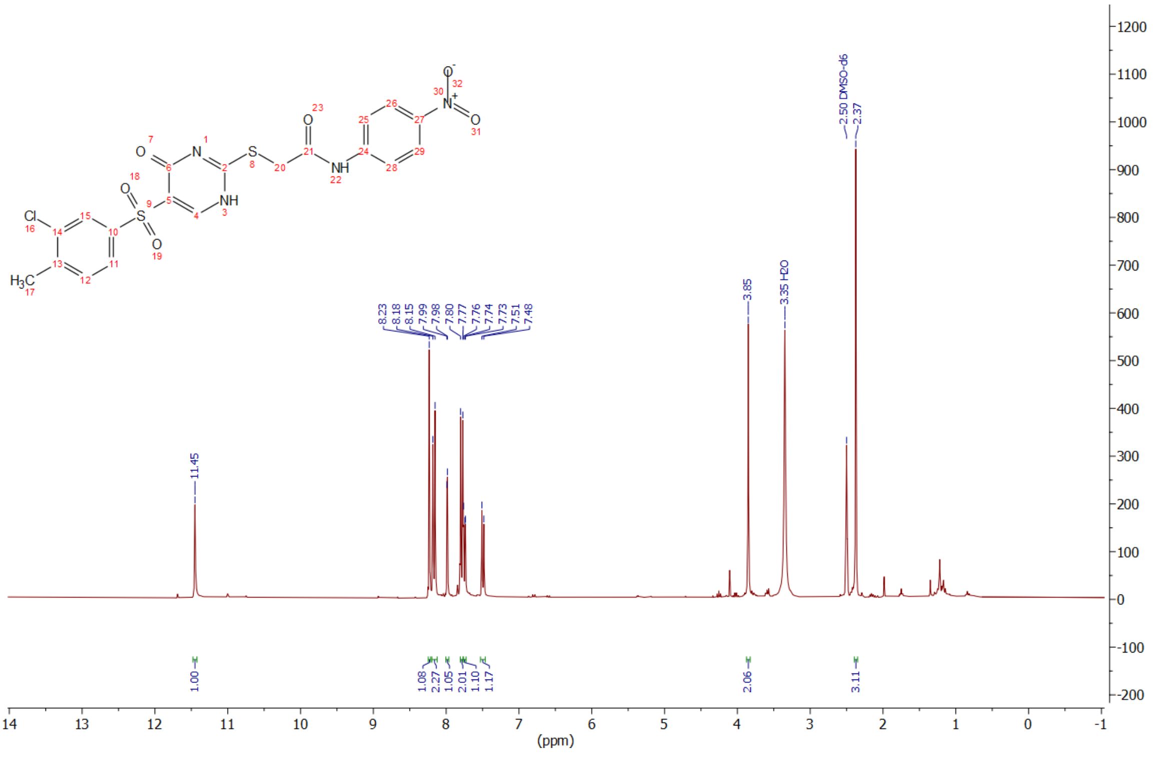

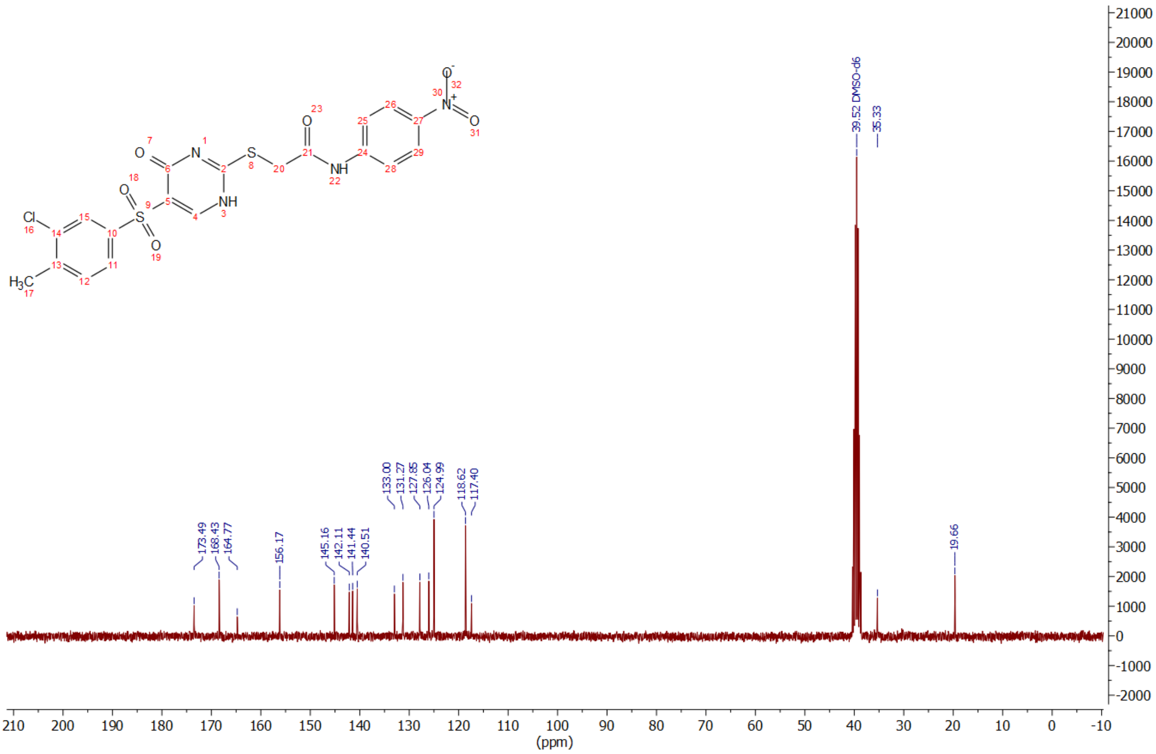

Figure S24. *^{1}H (300 MHz) and $^{13}C\{1H\}$ (75 MHz) NMR spectra in DMSO-d_6 of* ***compound 14***

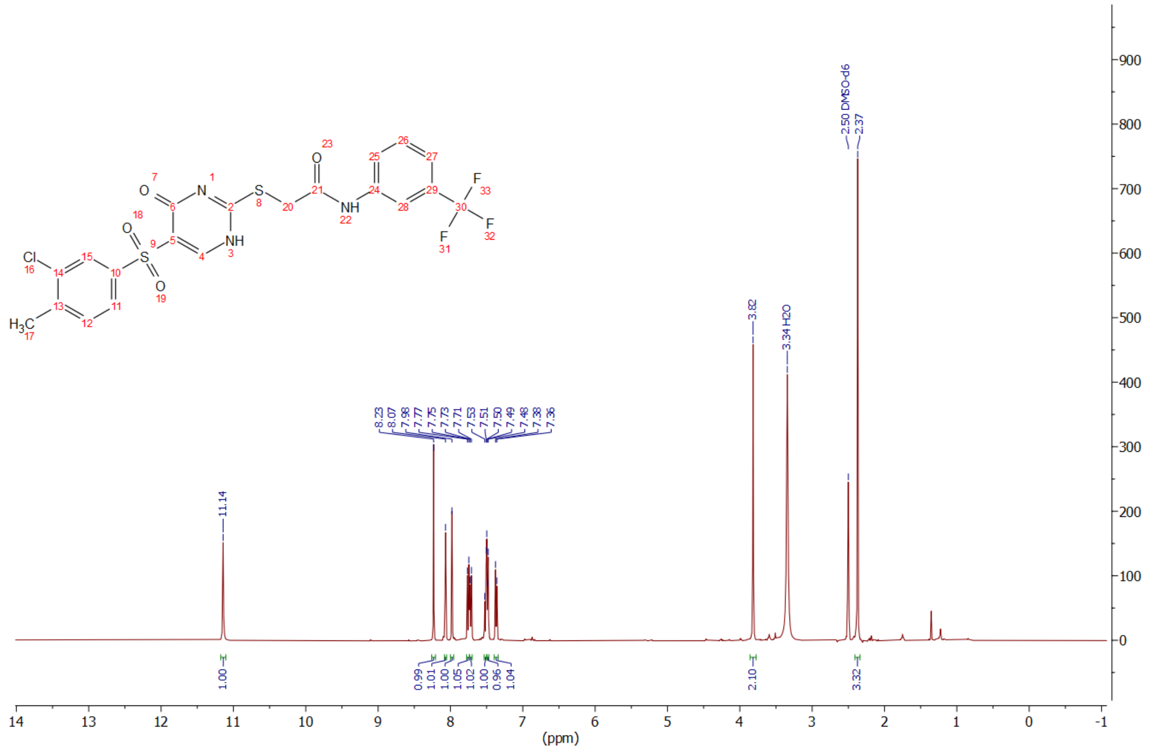

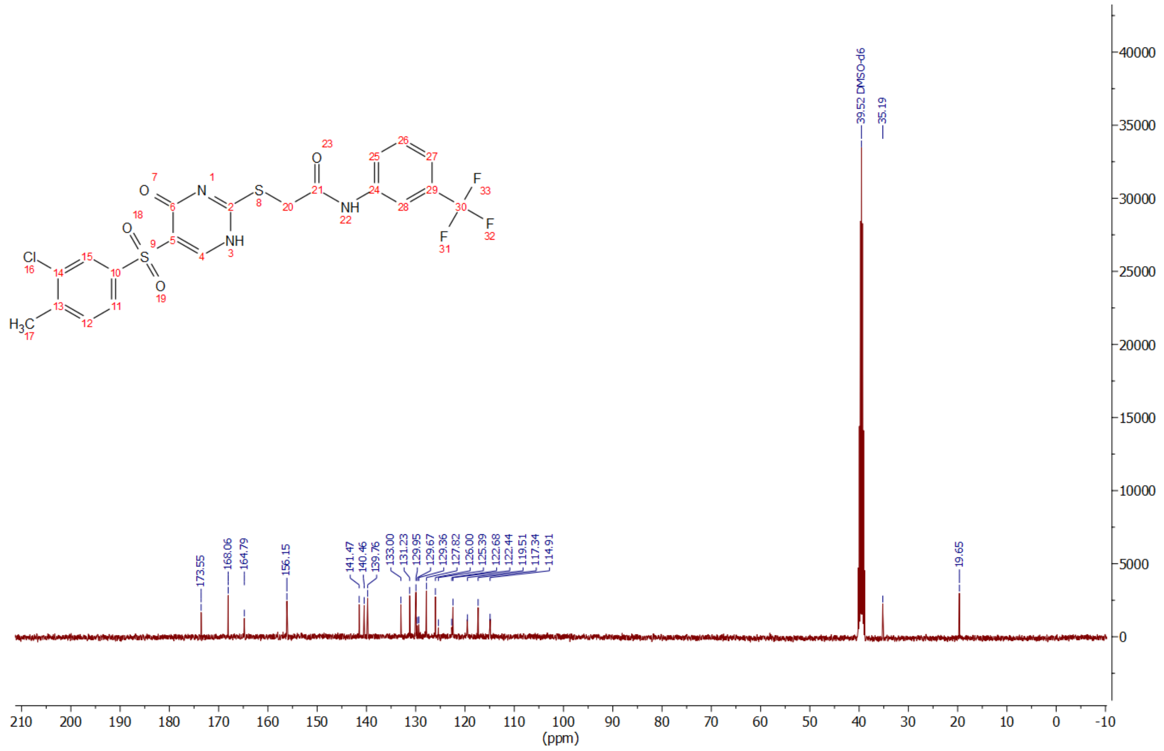

Figure S25. *^{1}H (400 MHz) and ^{13}C{1H} (101 MHz) NMR spectra in DMSO-d$_6$ of* ***compound 15***

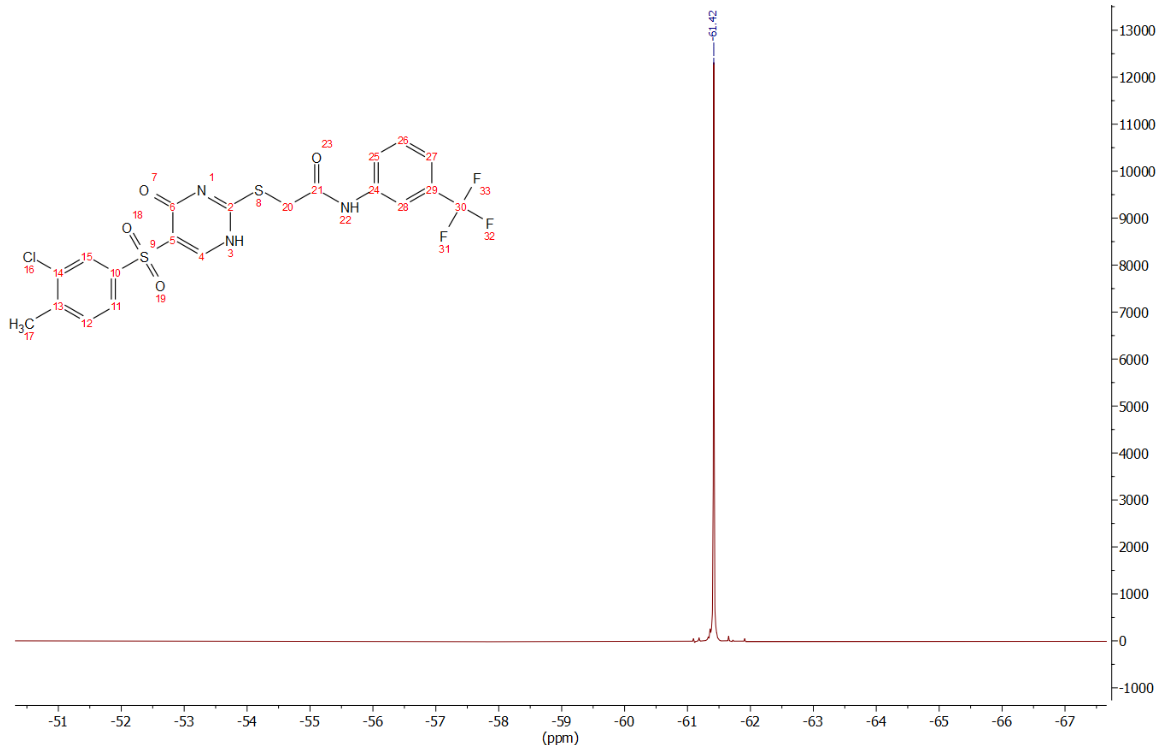

Figure S26. *^{19}F (376 MHz) NMR spectrum in DMSO-d$_6$ of* ***compound 15***

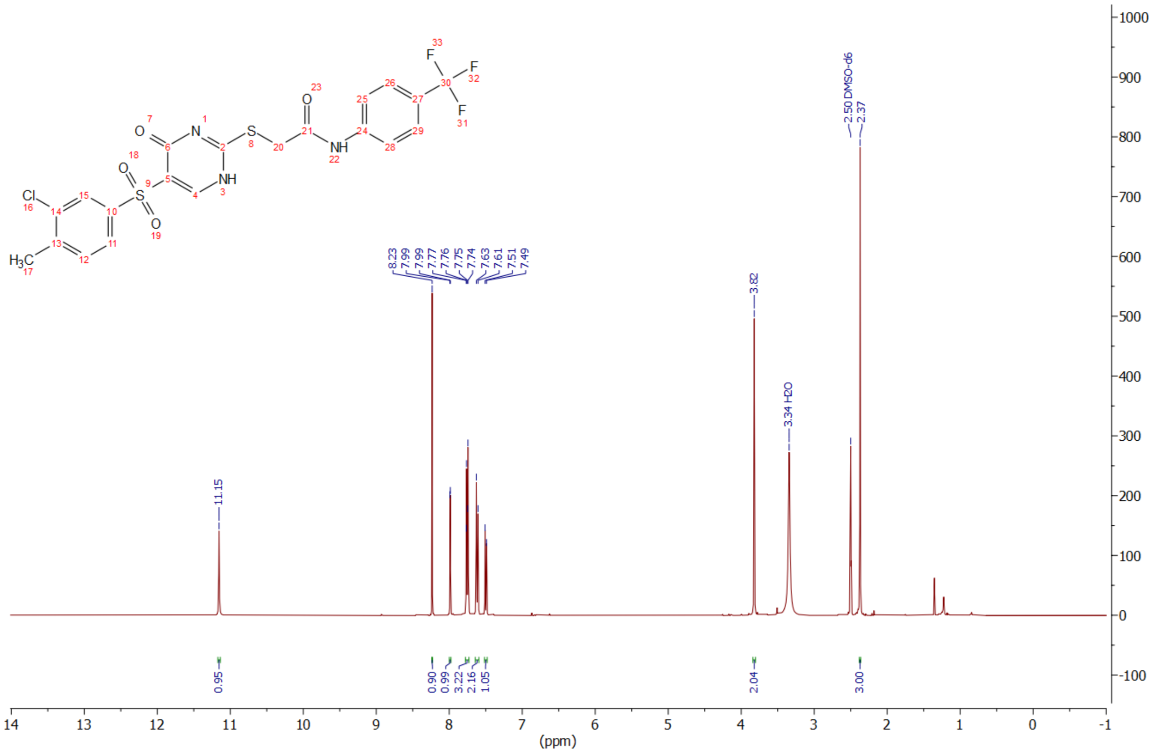

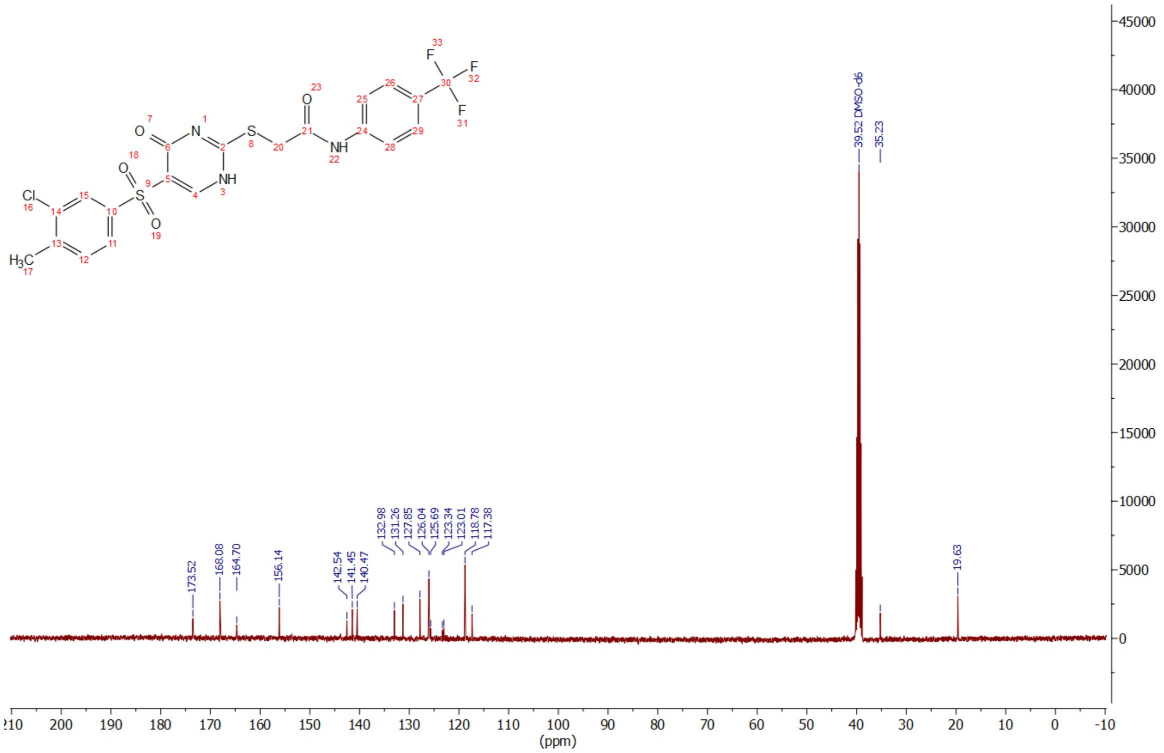

Figure S27. *^{1}H (400 MHz) and ^{13}C{1H} (101 MHz) NMR spectra in DMSO-d$_6$ of* ***compound 16***

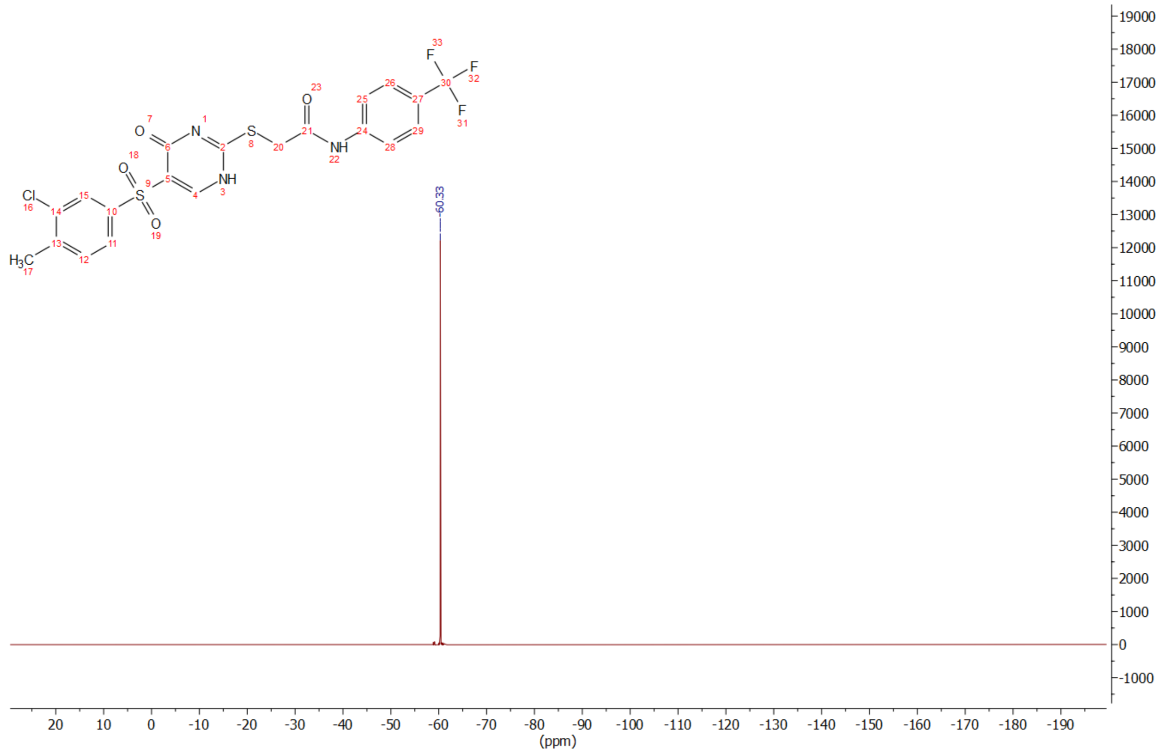

Figure S28. *^{19}F (376 MHz) NMR spectrum in DMSO-d_6 of* ***compound 16***

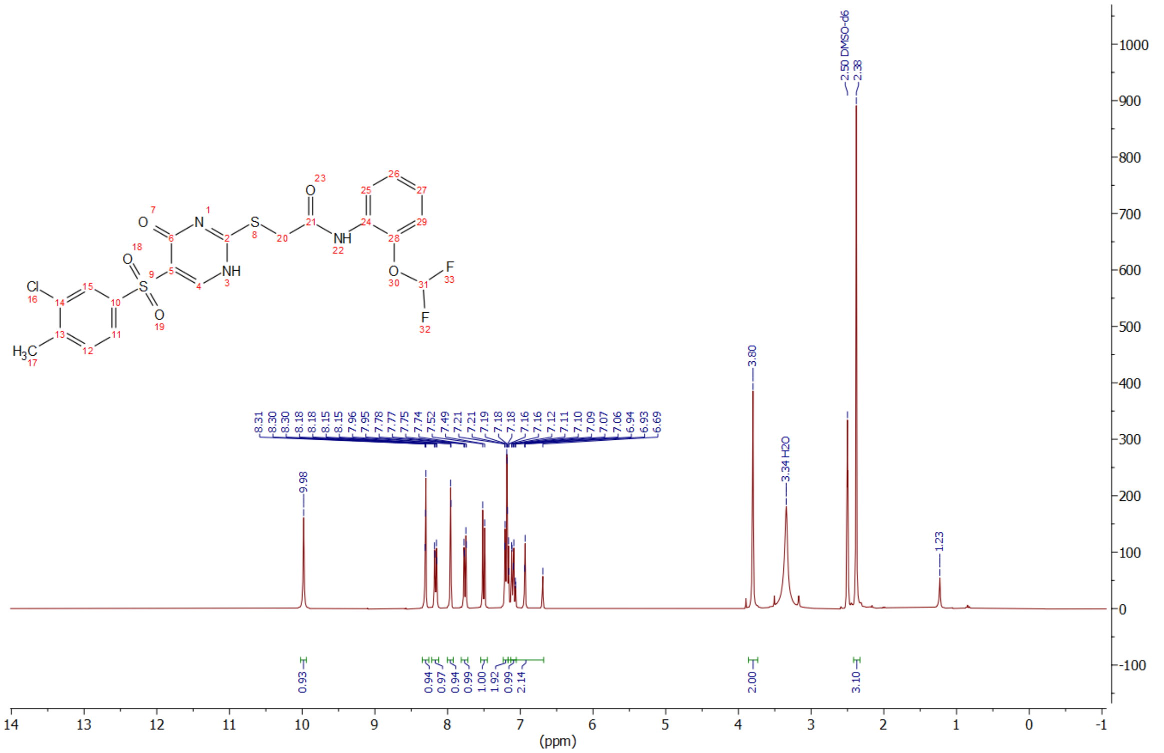

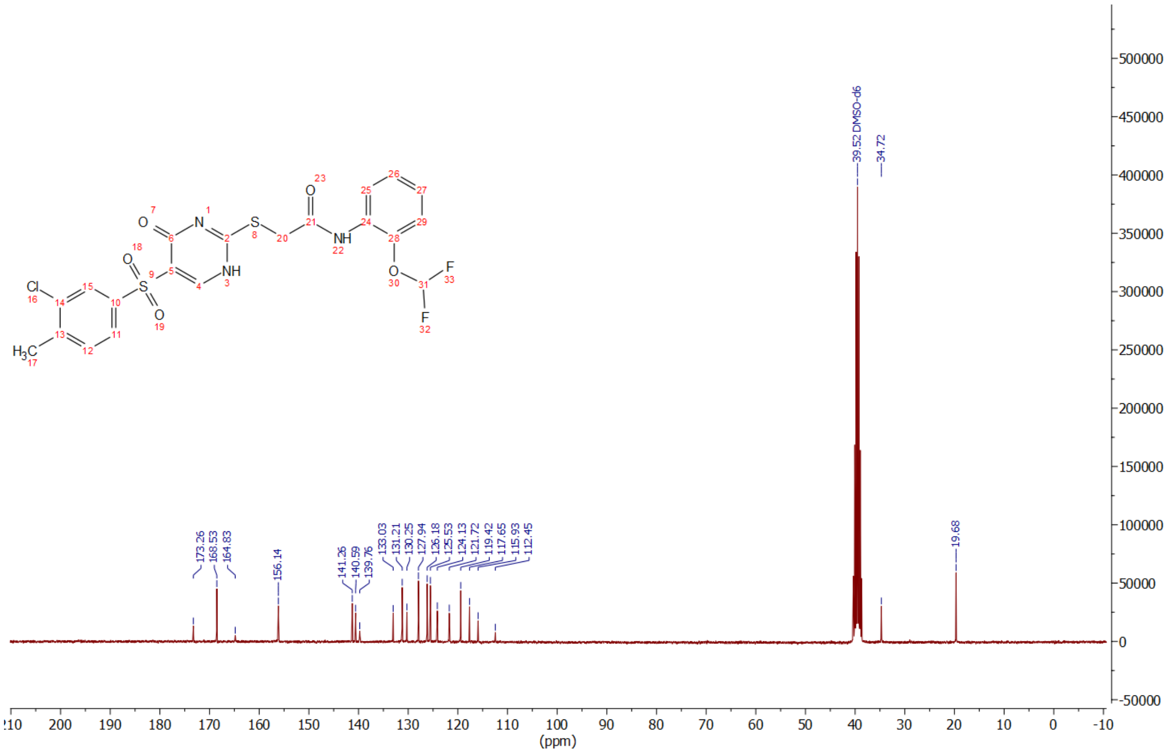

Figure S29. *¹H (300 MHz) and ¹³C{1H} (75 MHz) NMR spectra in DMSO-d₆ of* ***compound 17***

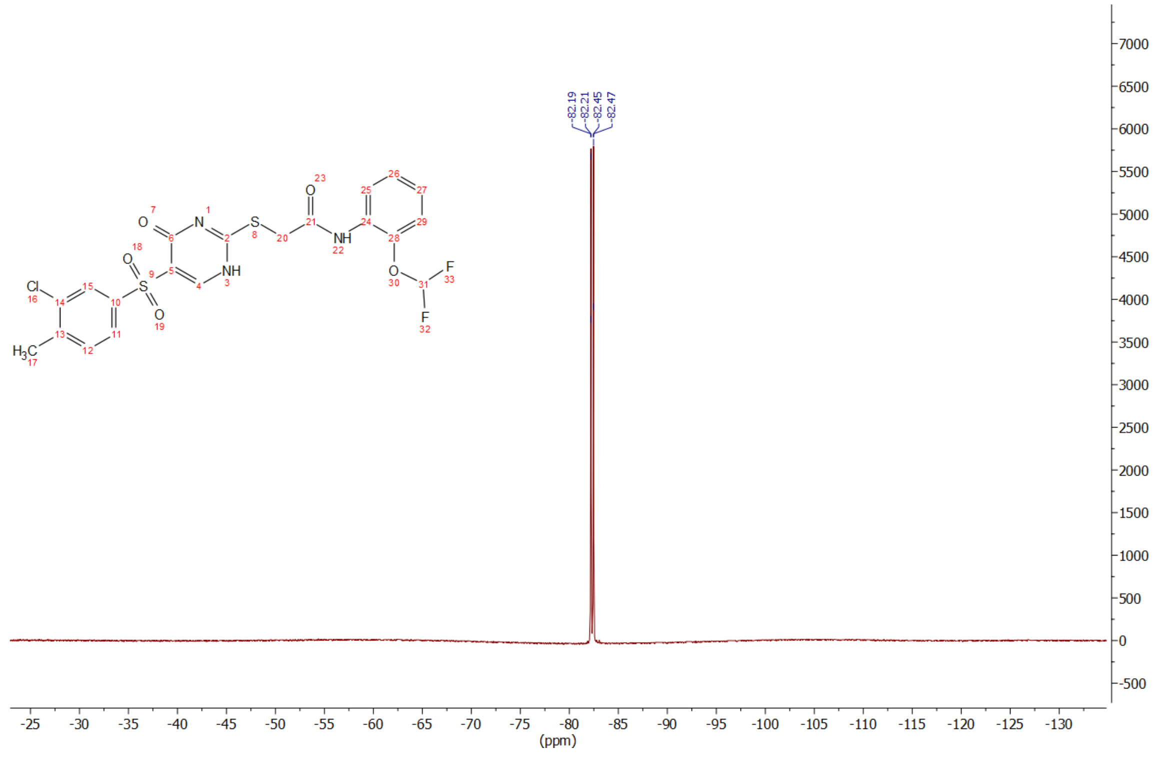

Figure S30. *^{19}F (282 MHz) NMR spectrum in DMSO-d$_6$ of* ***compound 17***

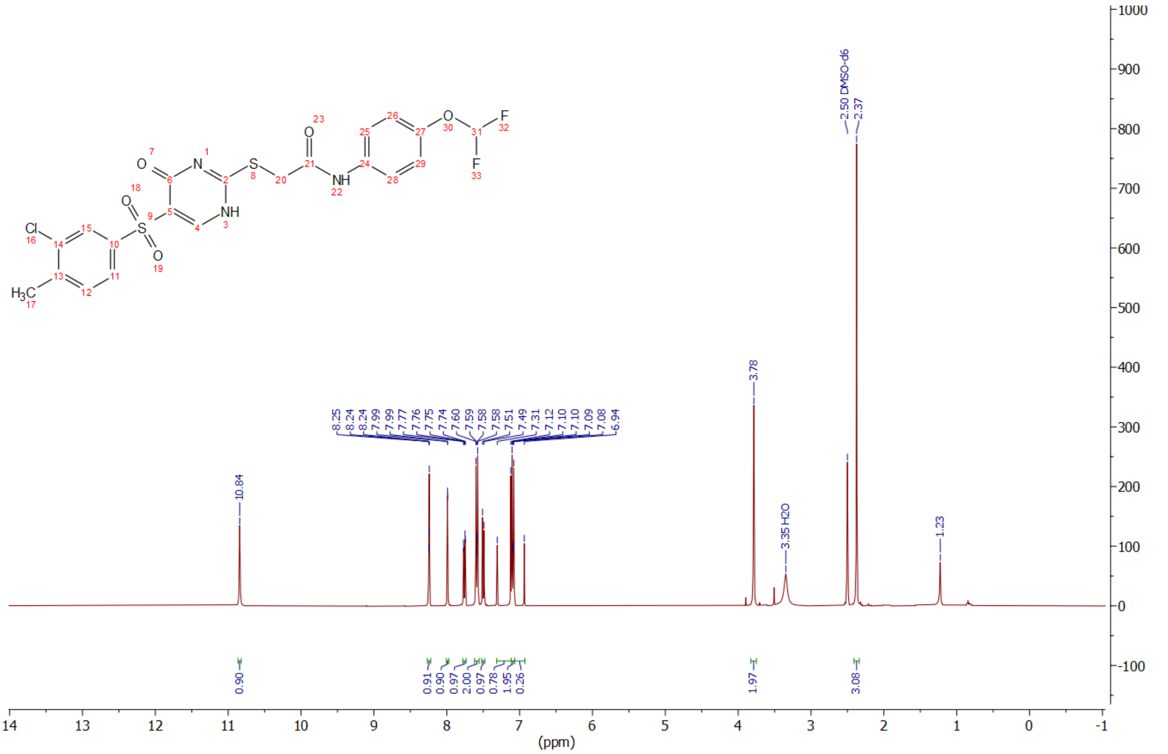

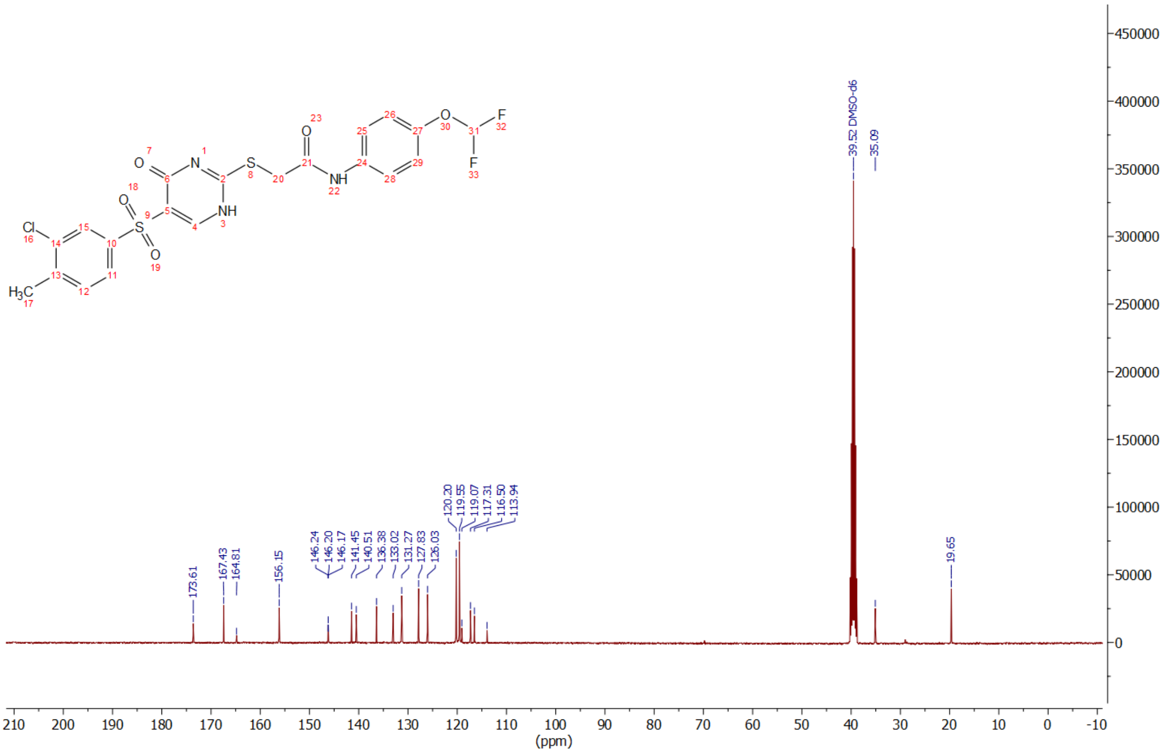

Figure S31. *^{1}H (400 MHz) and ^{13}C{1H} (101 MHz) NMR spectra in DMSO-d$_6$ of* ***compound 18***

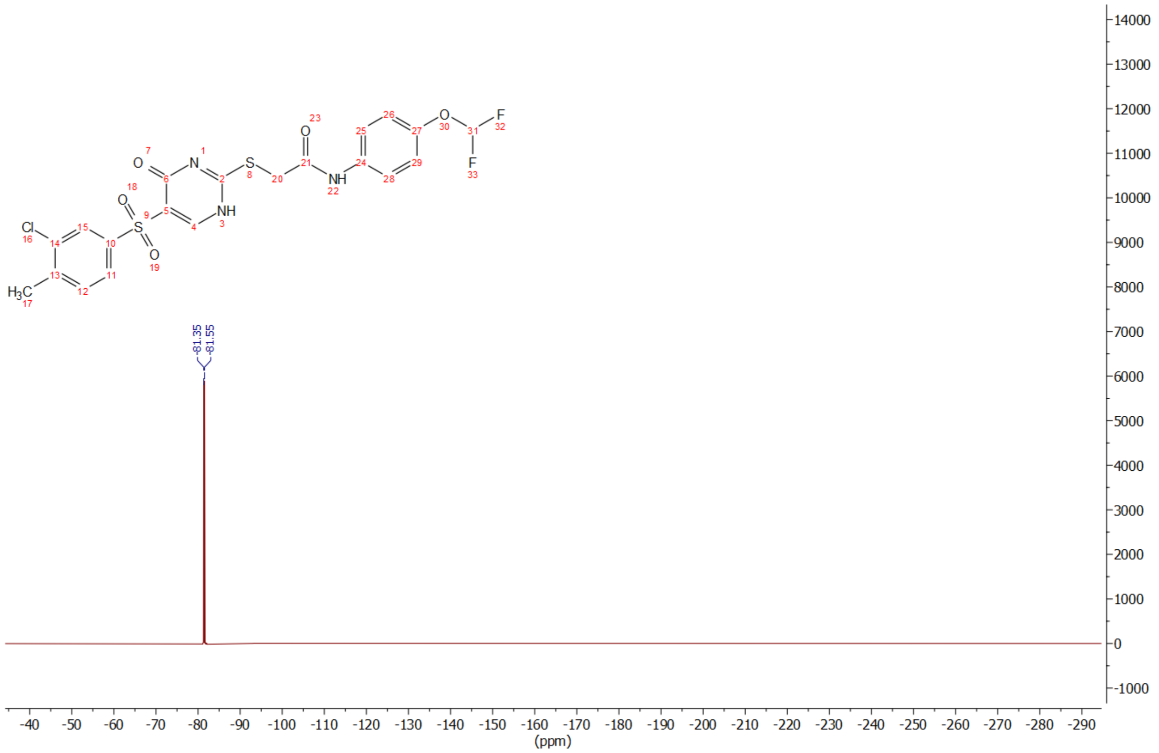

Figure S32. ^{19}*F (376 MHz) NMR spectrum in DMSO-d$_6$ of* ***compound 18***

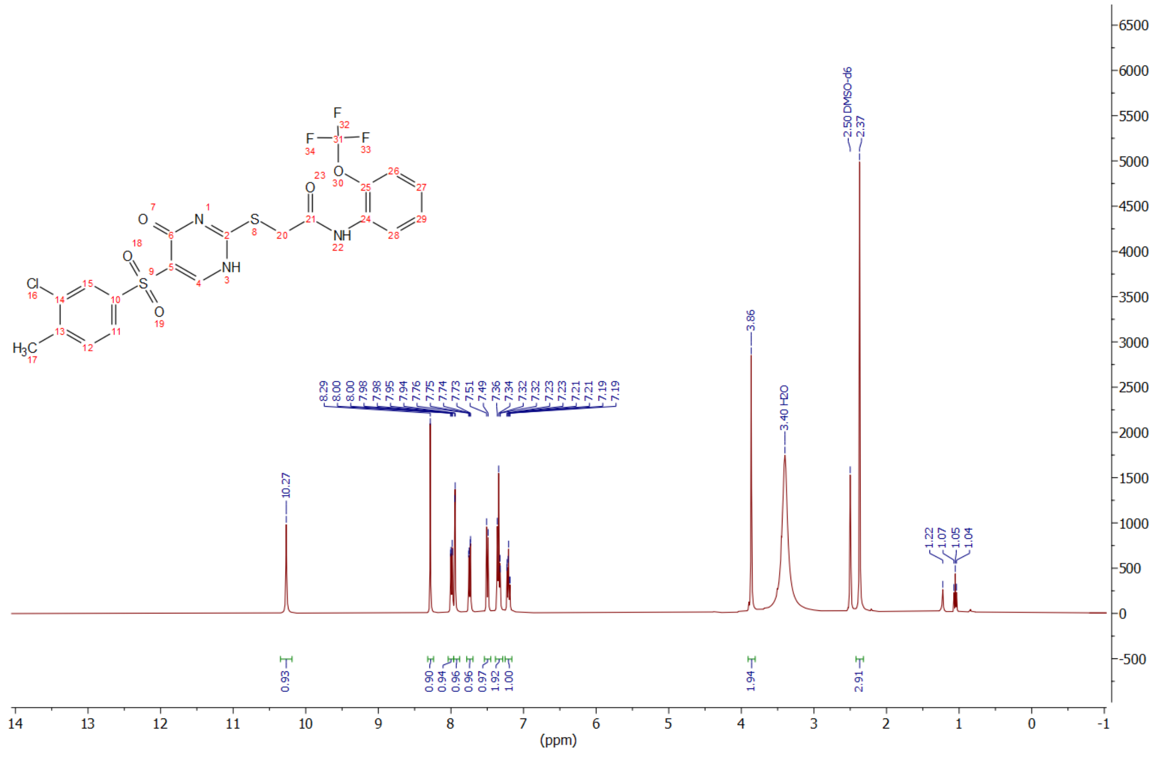

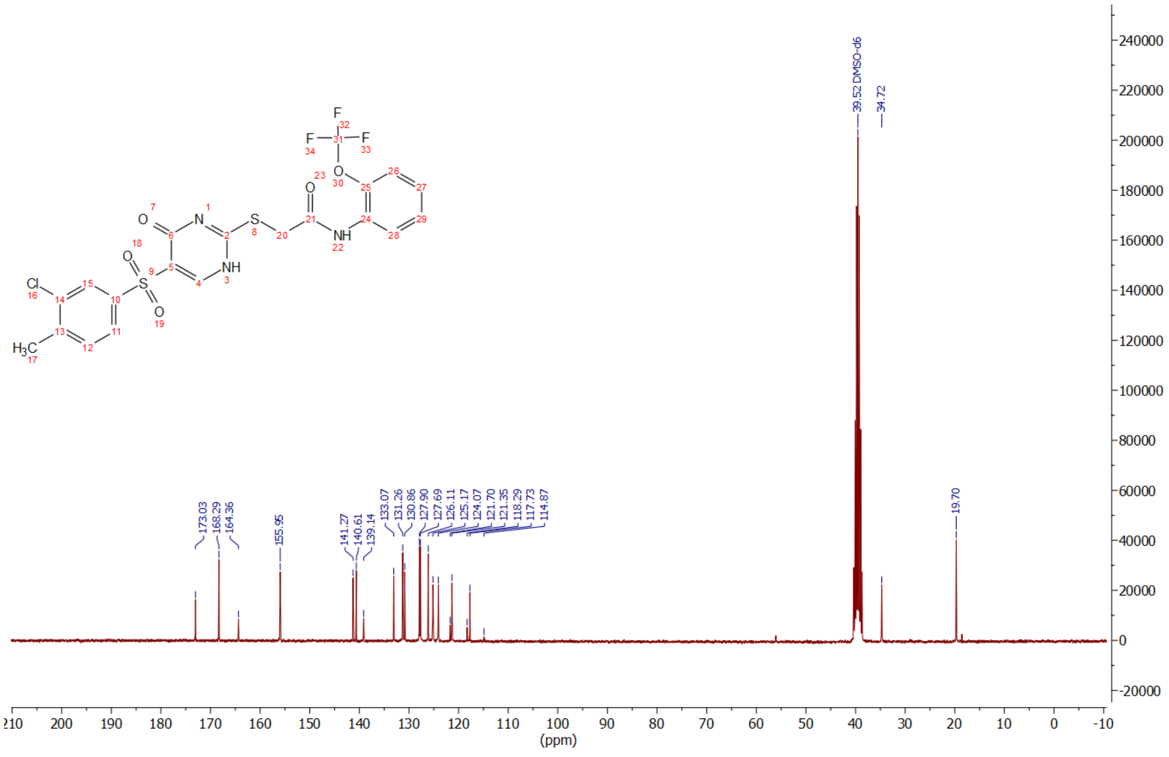

Figure S33. *^1H (400 MHz) and $^{13}C\{1H\}$ (101 MHz) NMR spectra in DMSO-d_6 of **compound 19***

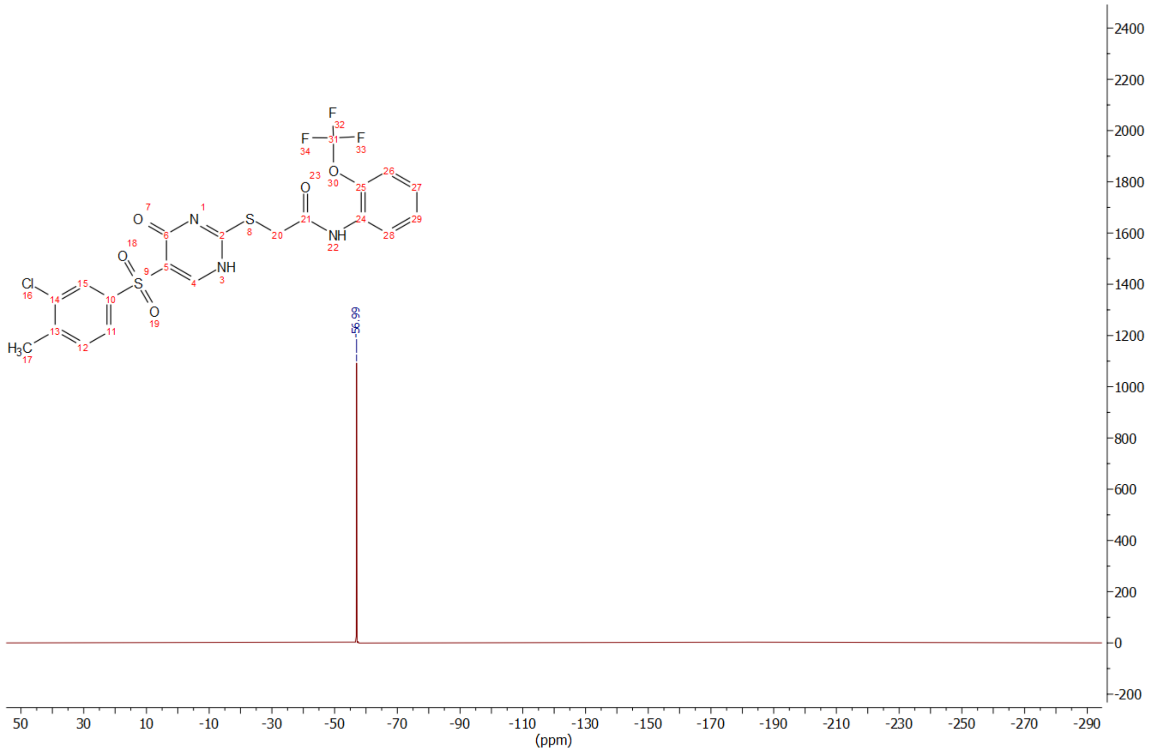

Figure S34. *^{19}F (377 MHz) NMR spectrum in DMSO-d$_6$ of* ***compound 19***

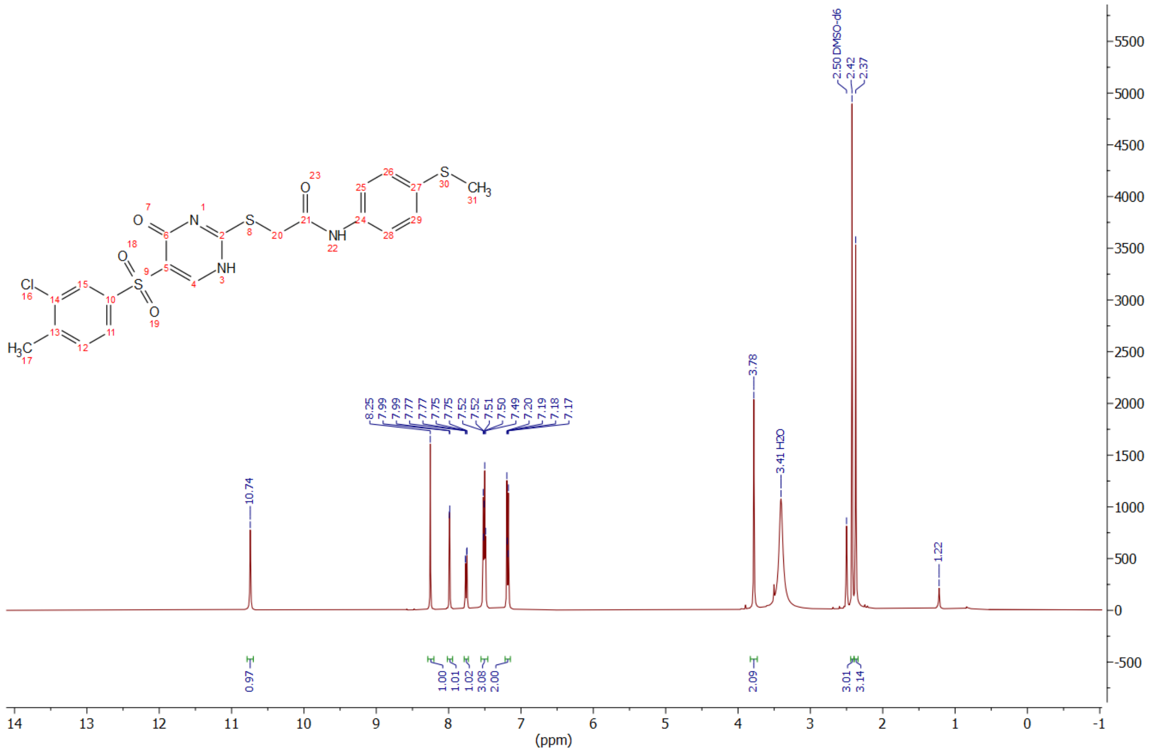

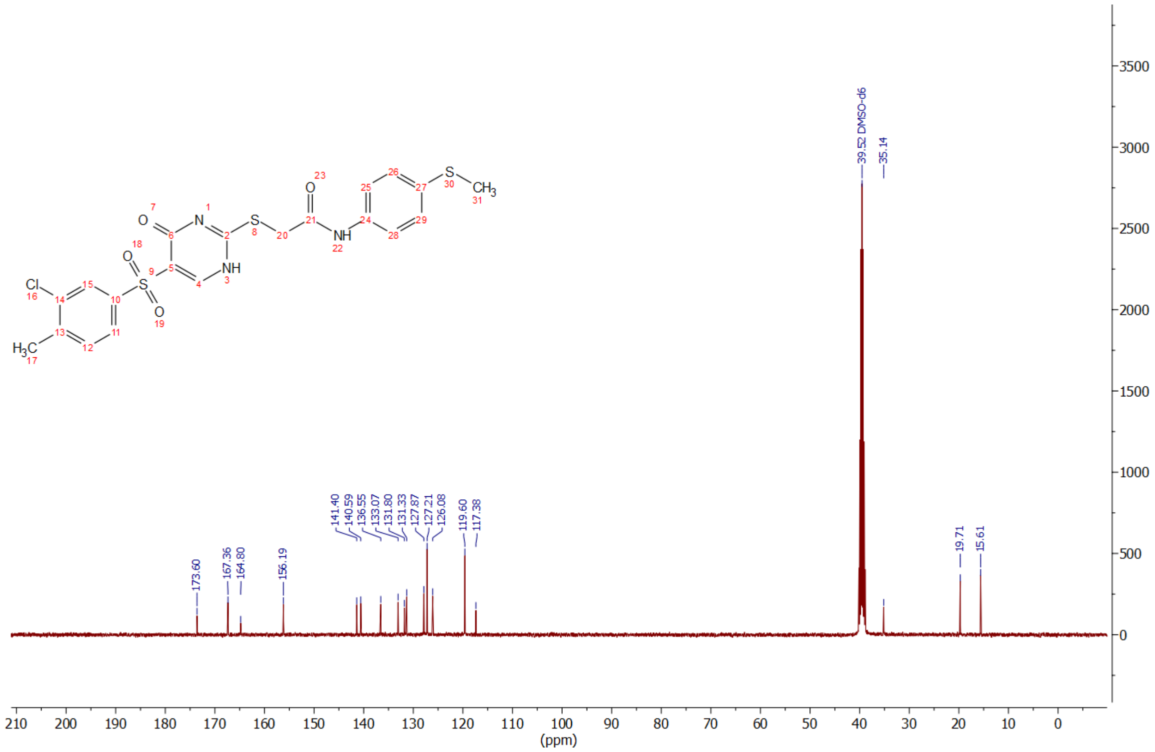

Figure S35. *^{1}H (400 MHz) and ^{13}C{1H} (101 MHz) NMR spectra in DMSO-d$_6$ of* ***compound 20***

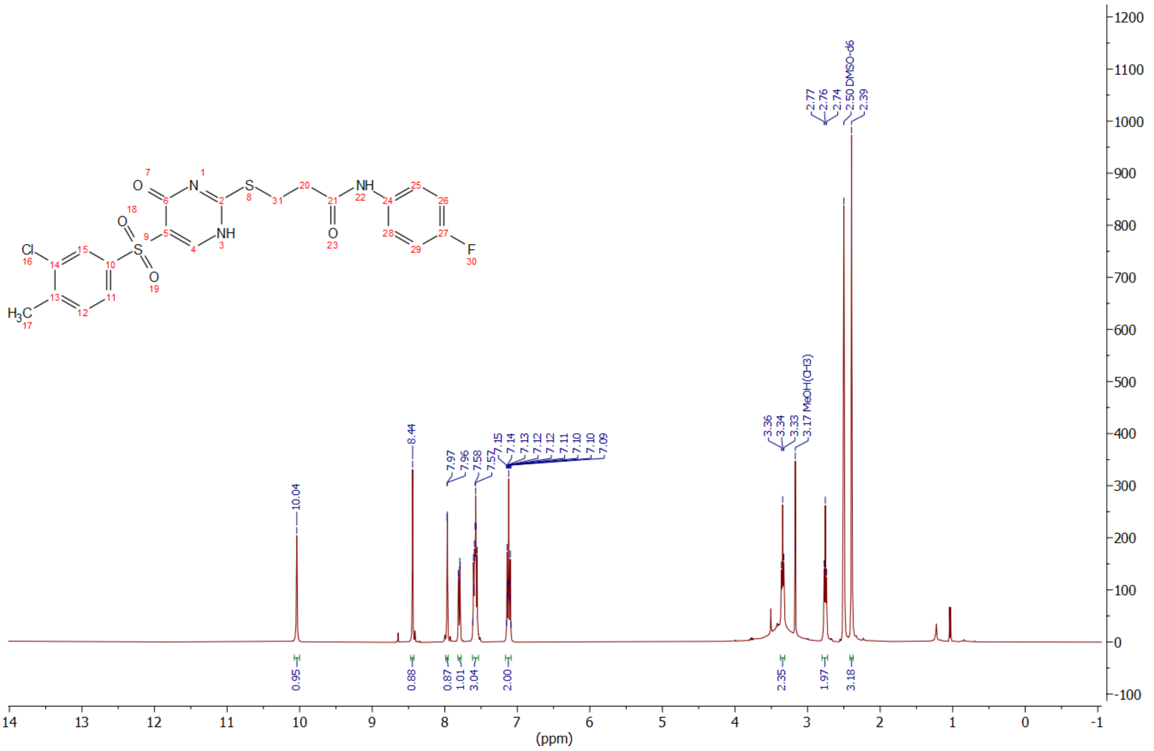

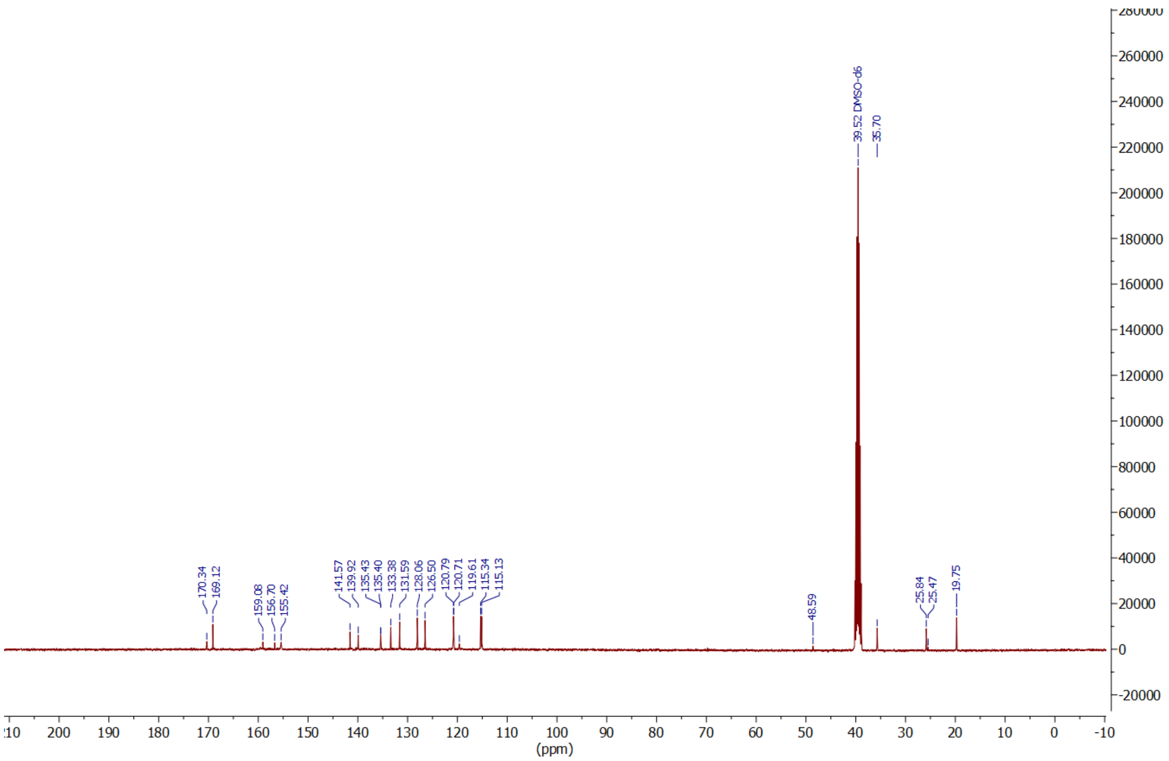

Figure S36. *^{1}H (400 MHz) and $^{13}C\{1H\}$ (101 MHz) NMR spectra in DMSO-d_6 of **compound 21***

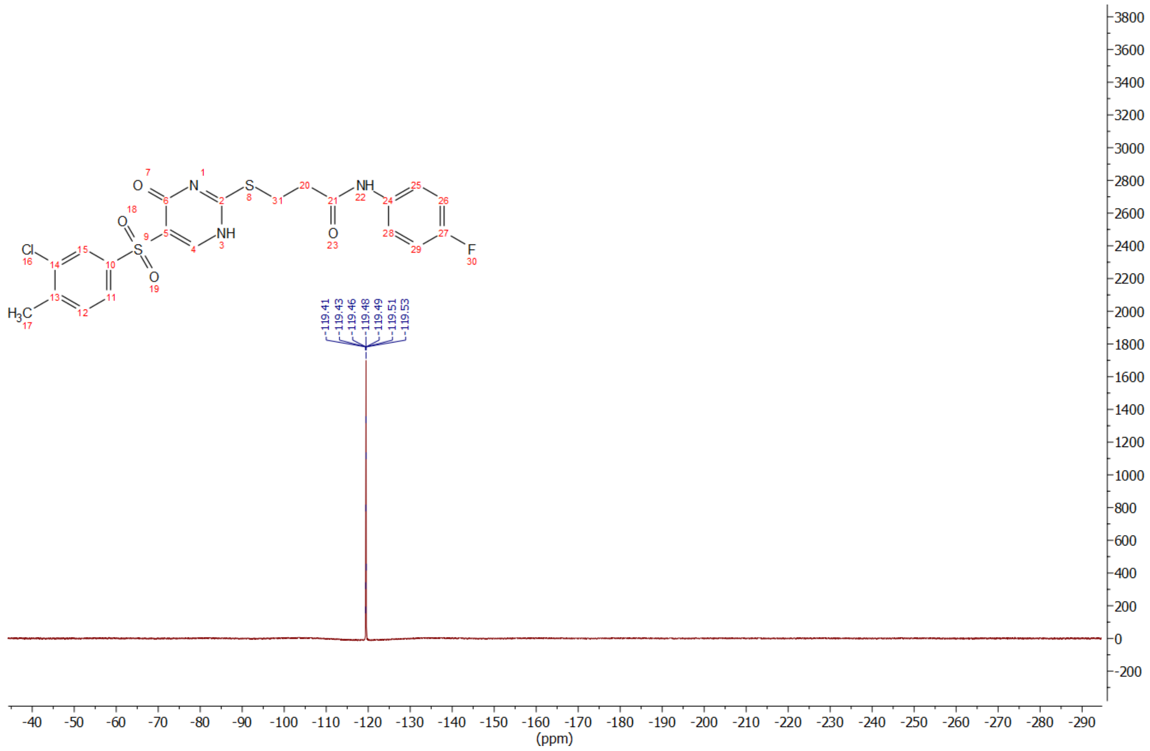

Figure S37. *^{19}F (376 MHz) NMR spectrum in DMSO-d_6 of* ***compound 21***

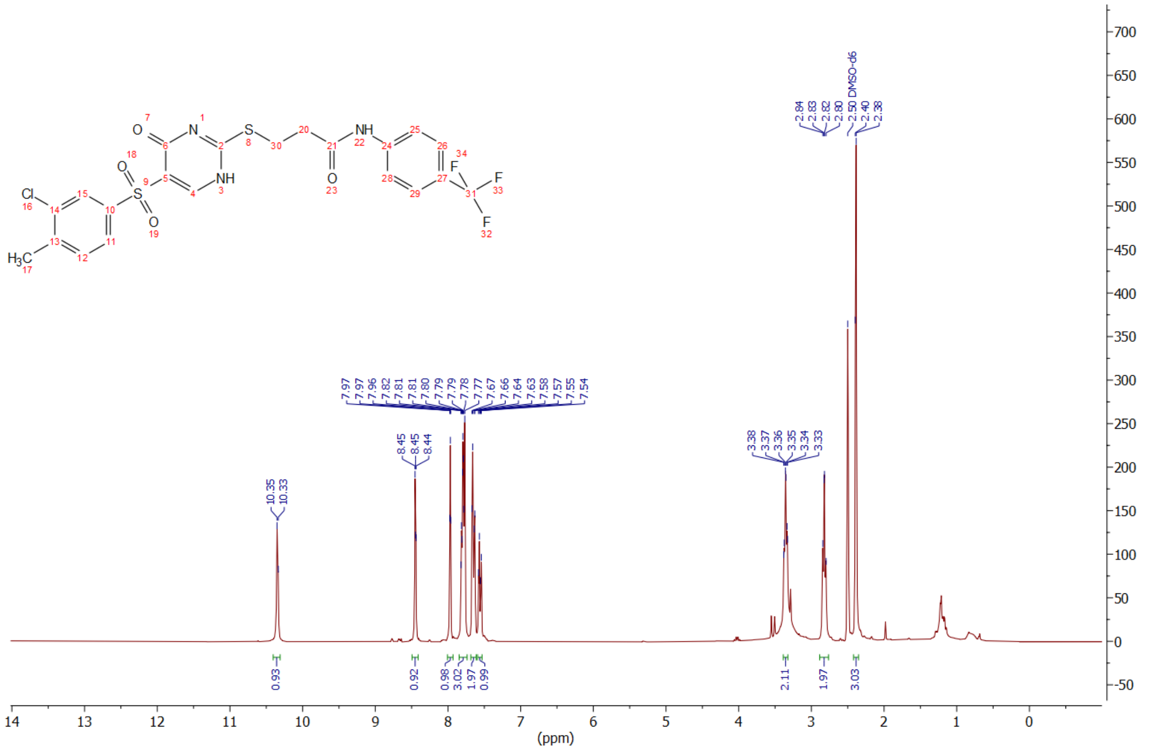

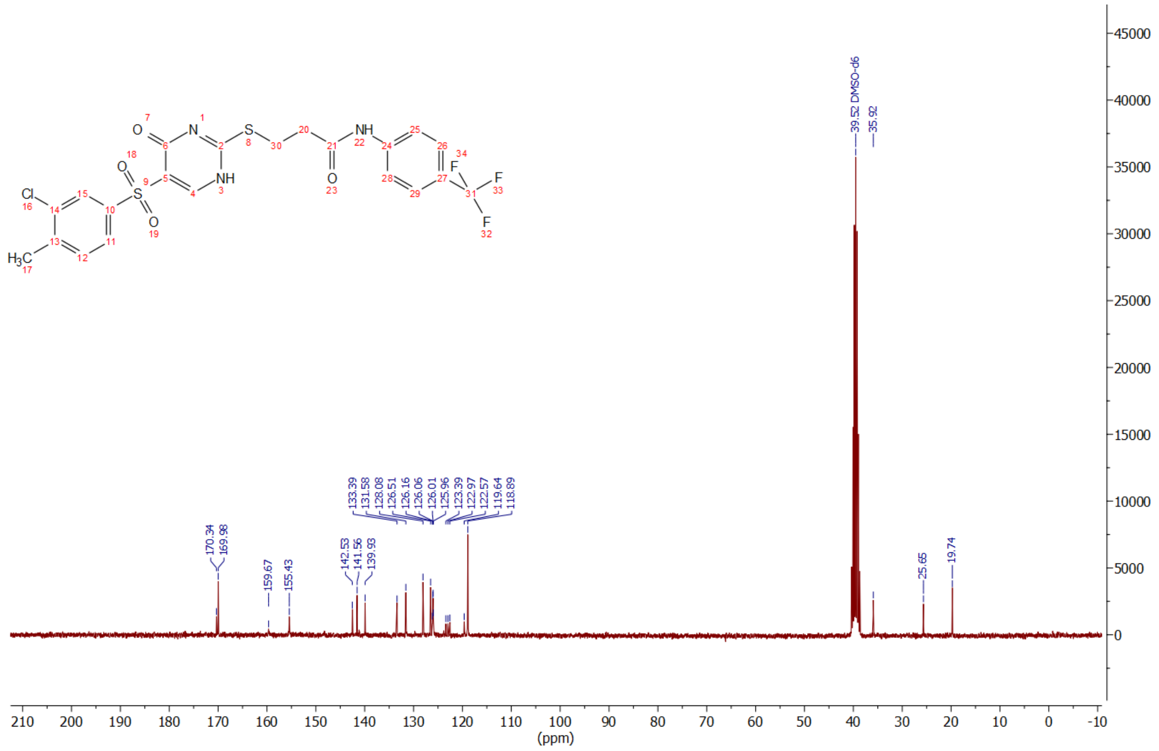

Figure S38. *^{1}H (300 MHz) and ^{13}C{1H} (75 MHz) NMR spectra in DMSO-d$_6$ of* ***compound 22***

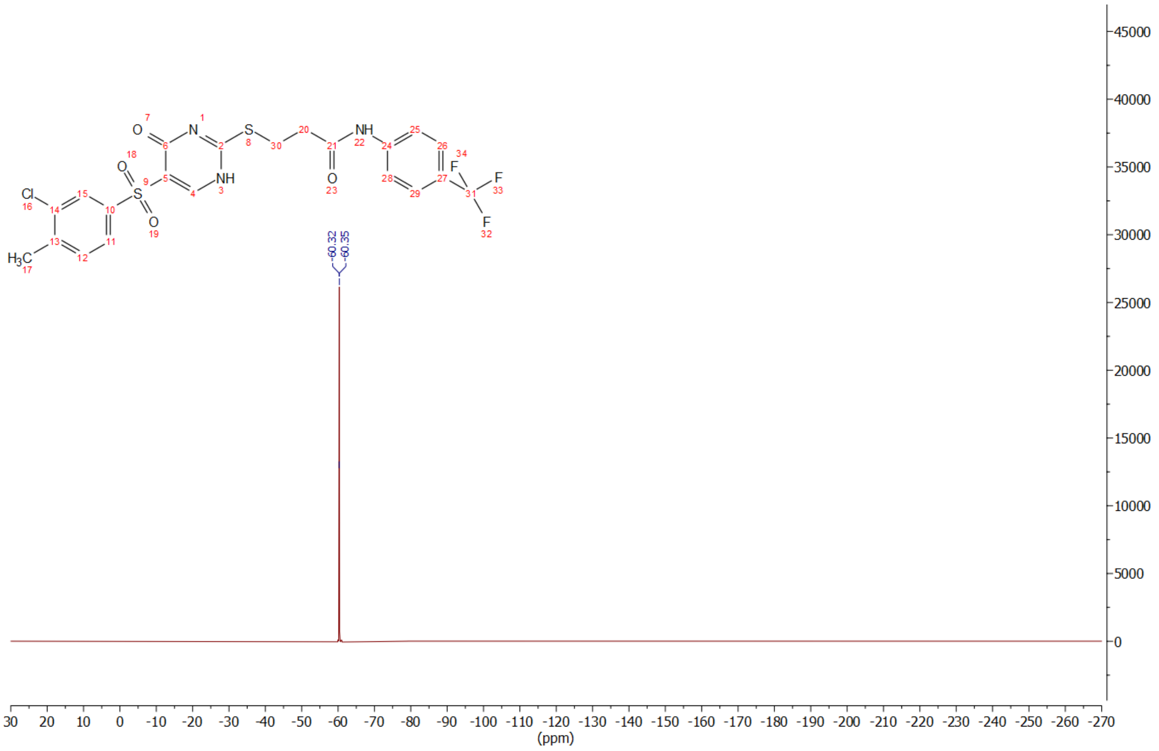

Figure S39. *^{19}F (282 MHz) NMR spectrum in DMSO-d$_6$ of* ***compound 22***

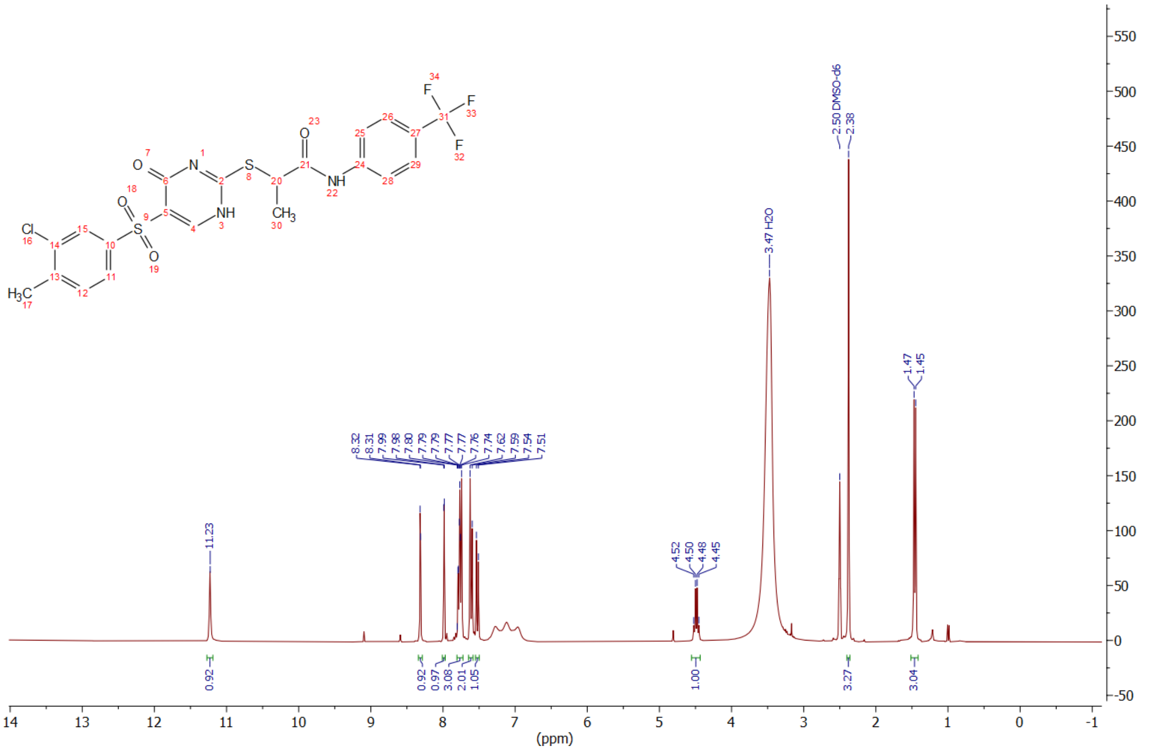

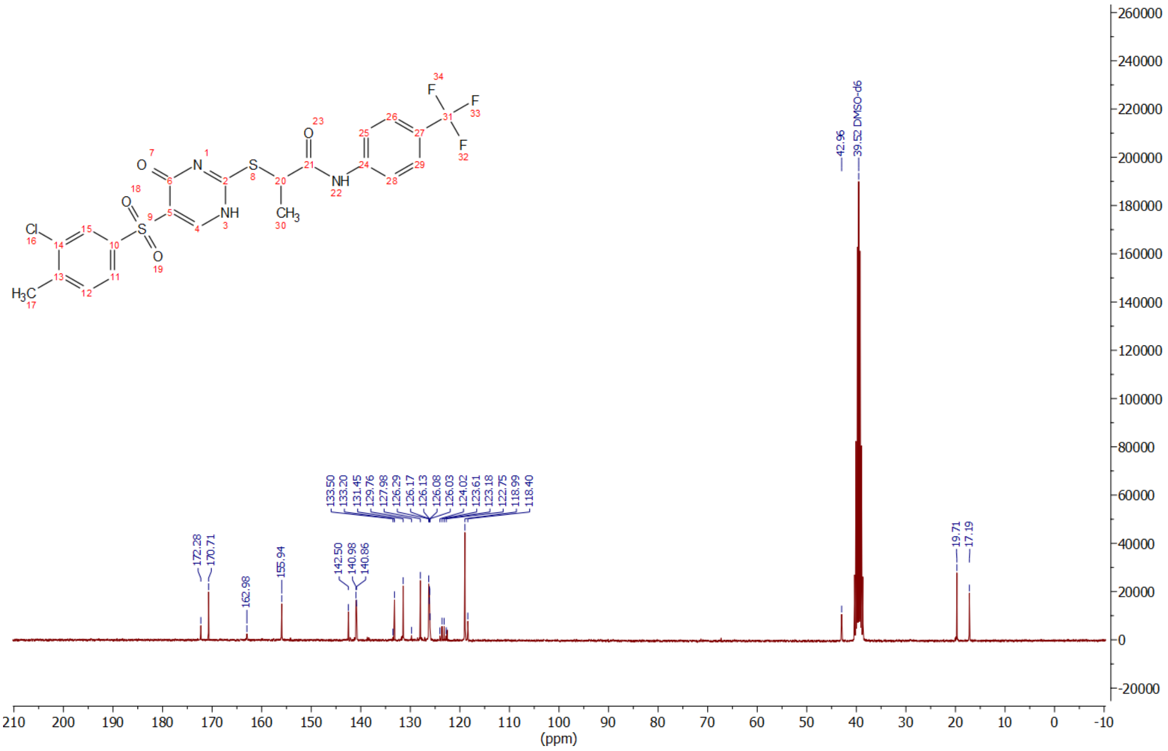

Figure S40. *^{1}H (300 MHz) and $^{13}C\{1H\}$ (75 MHz) NMR spectra in DMSO-d_6 of* ***compound 23***

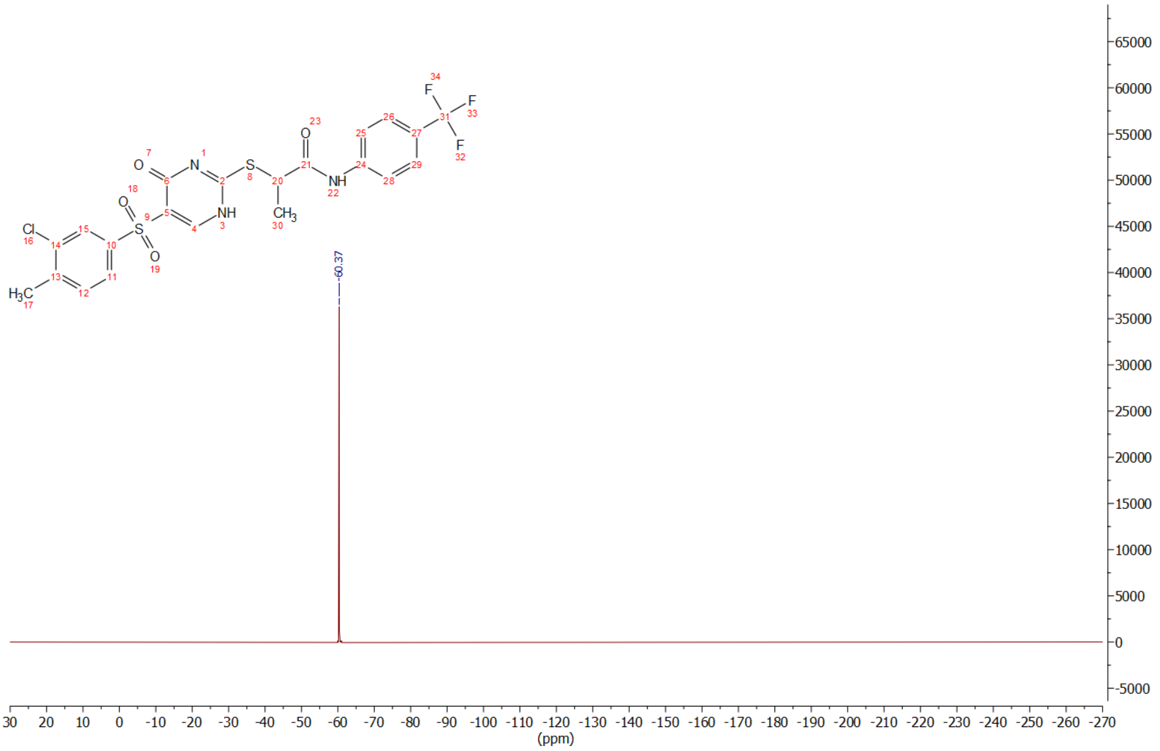

Figure S41. *^{19}F (282 MHz) NMR spectrum in DMSO-d_6 of* ***compound 23***

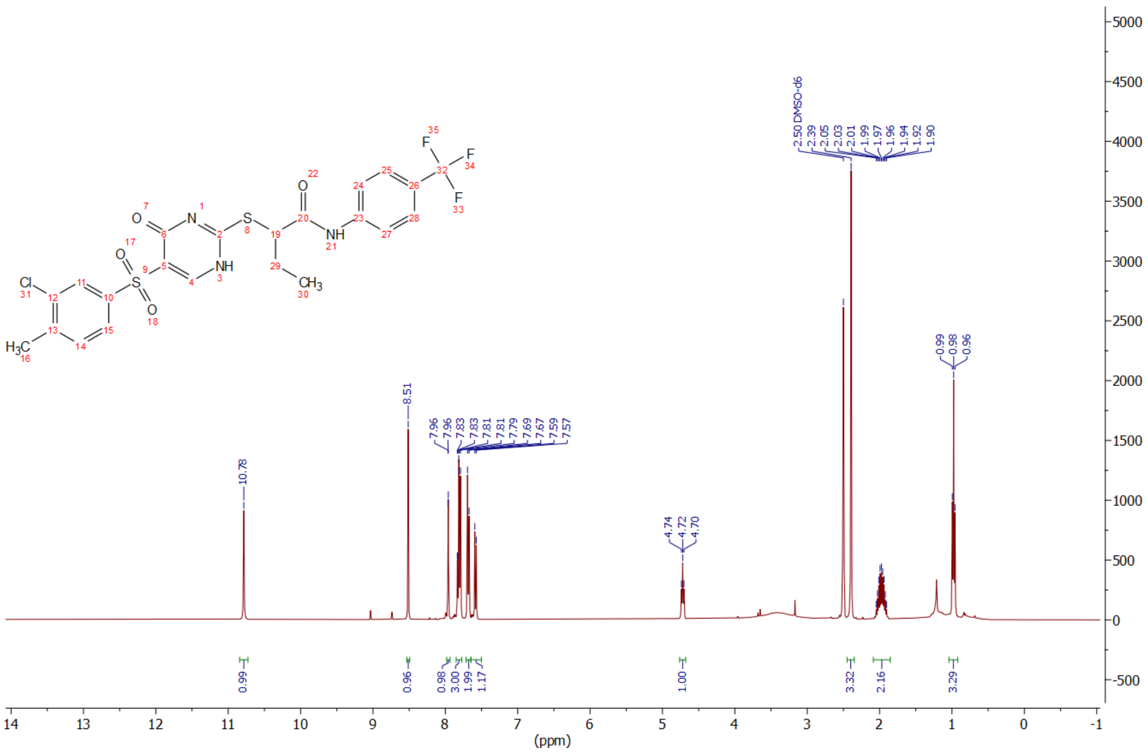

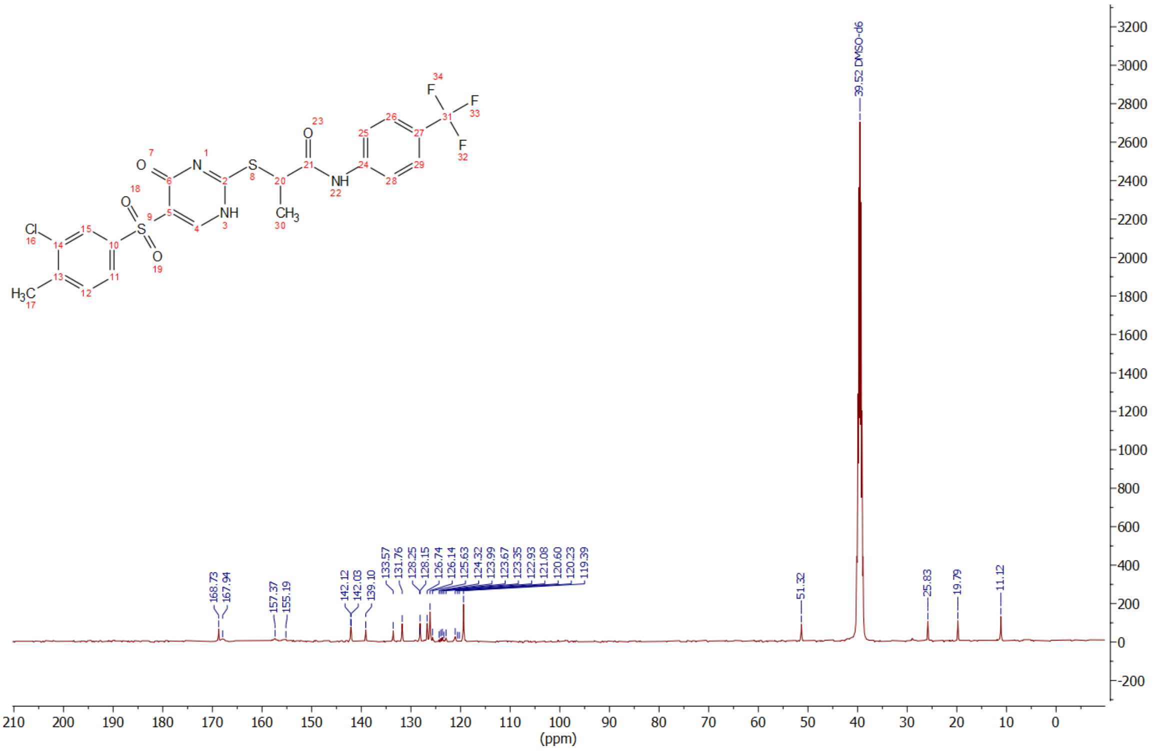

Figure S42. *^{1}H (400 MHz) and ^{13}C{1H} (101 MHz) NMR spectra in DMSO-d$_6$ of* ***compound 24***

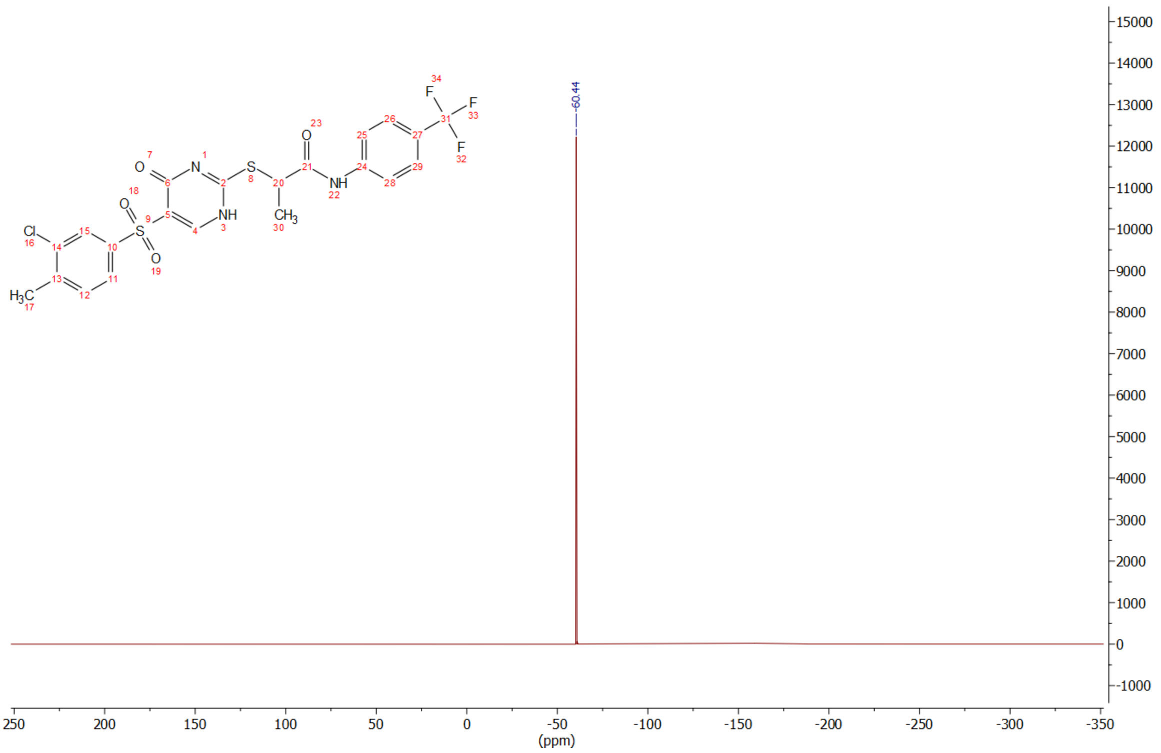

Figure S43. *^{19}F (377 MHz) NMR spectrum in DMSO-d_6 of* ***compound 24***

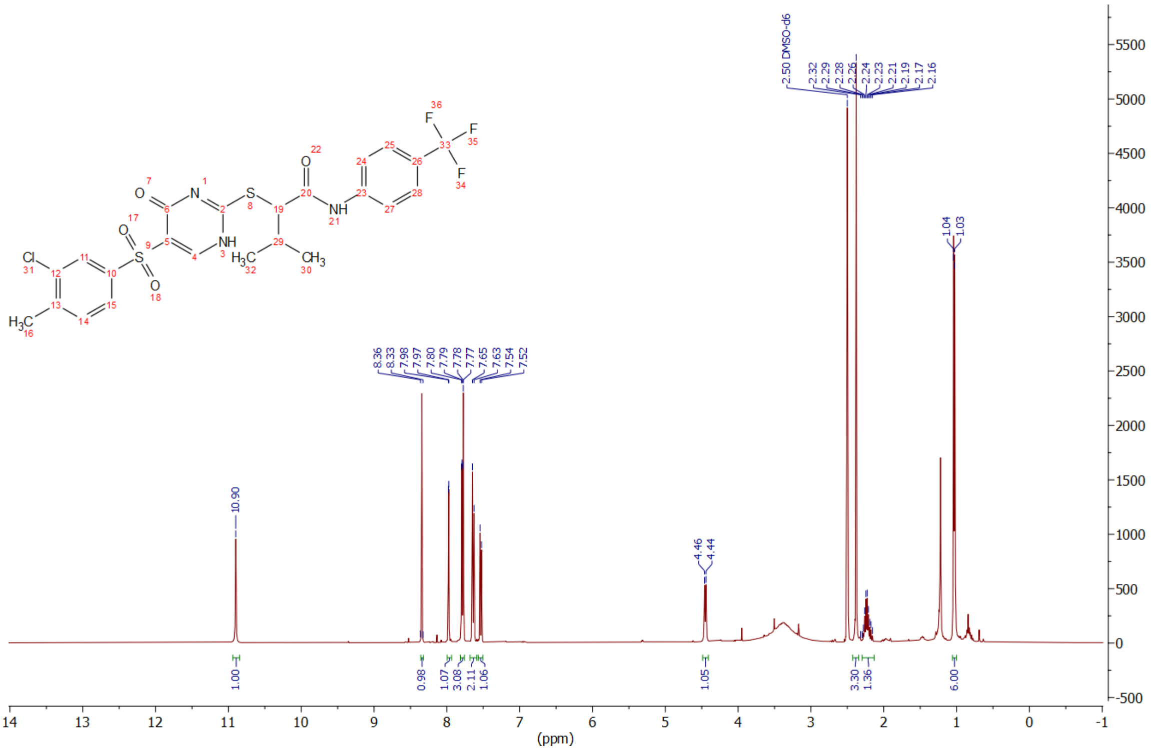

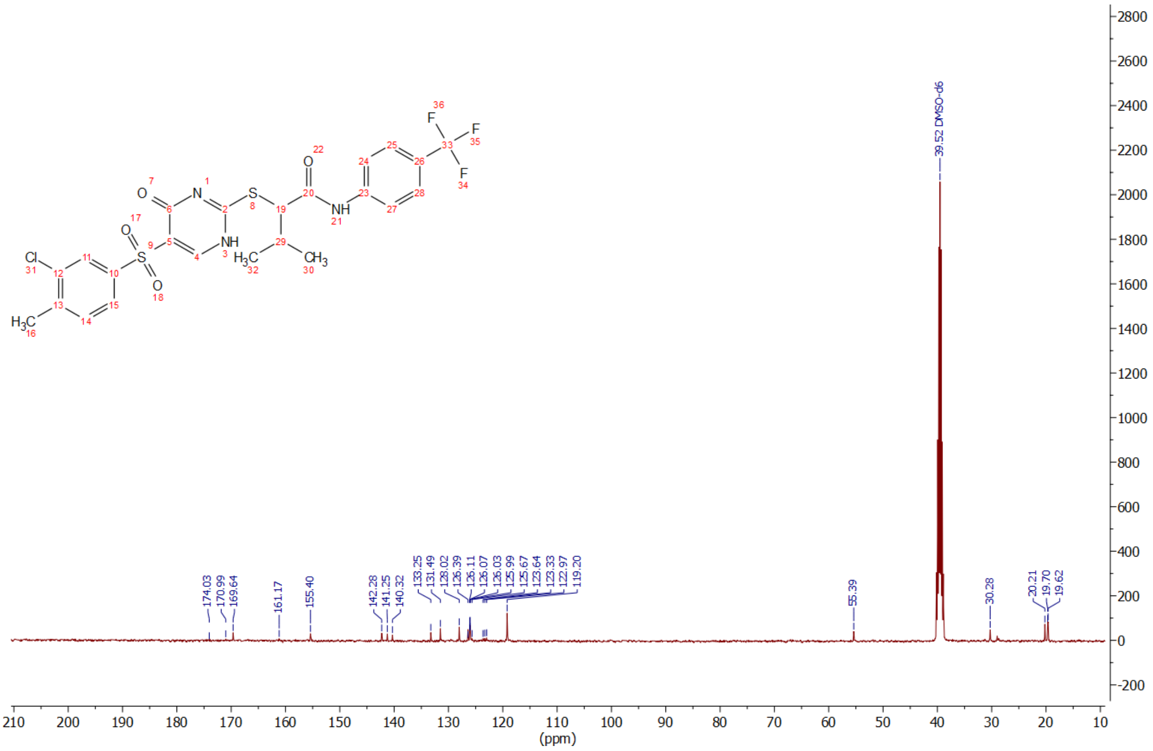

Figure S44. *^{1}H (400 MHz) and $^{13}C\{1H\}$ (101 MHz) NMR spectra in DMSO-d_6 of* ***compound 25***

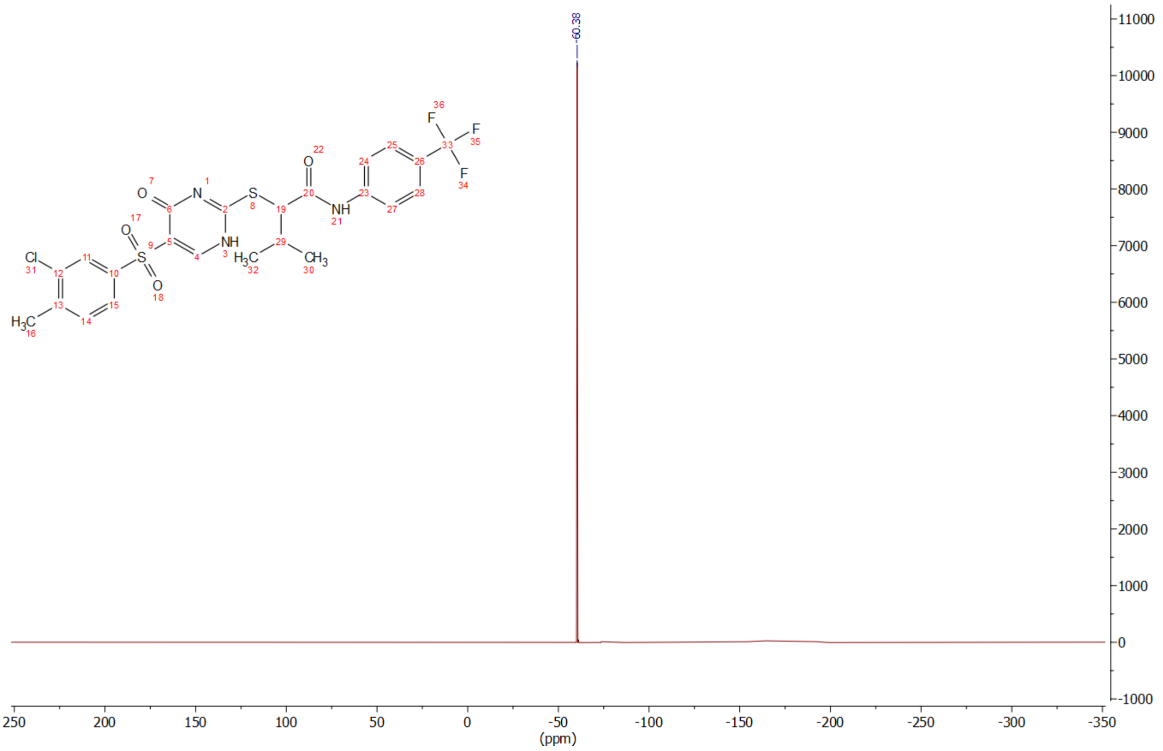

Figure S45. *^{19}F (377 MHz) NMR spectrum in DMSO-d_6 of* ***compound 25***

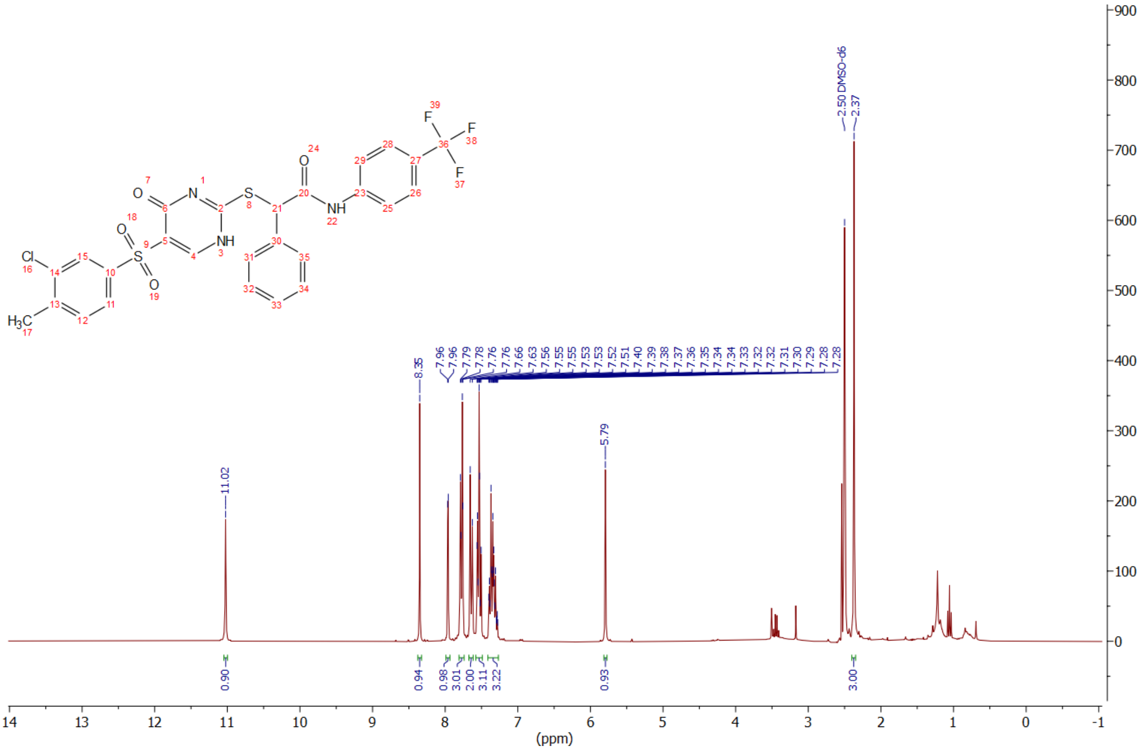

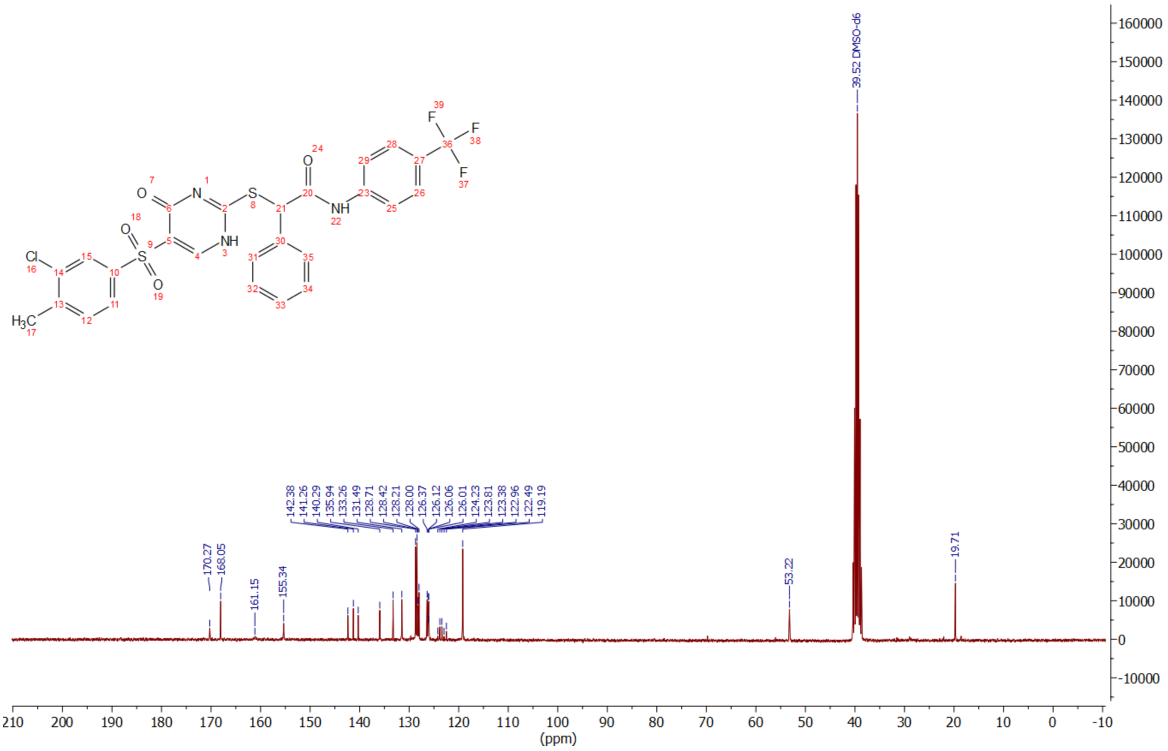

Figure S46. *^{1}H (300 MHz) and $^{13}C\{1H\}$ (75 MHz) NMR spectra in DMSO-d_6 of* ***compound 26***

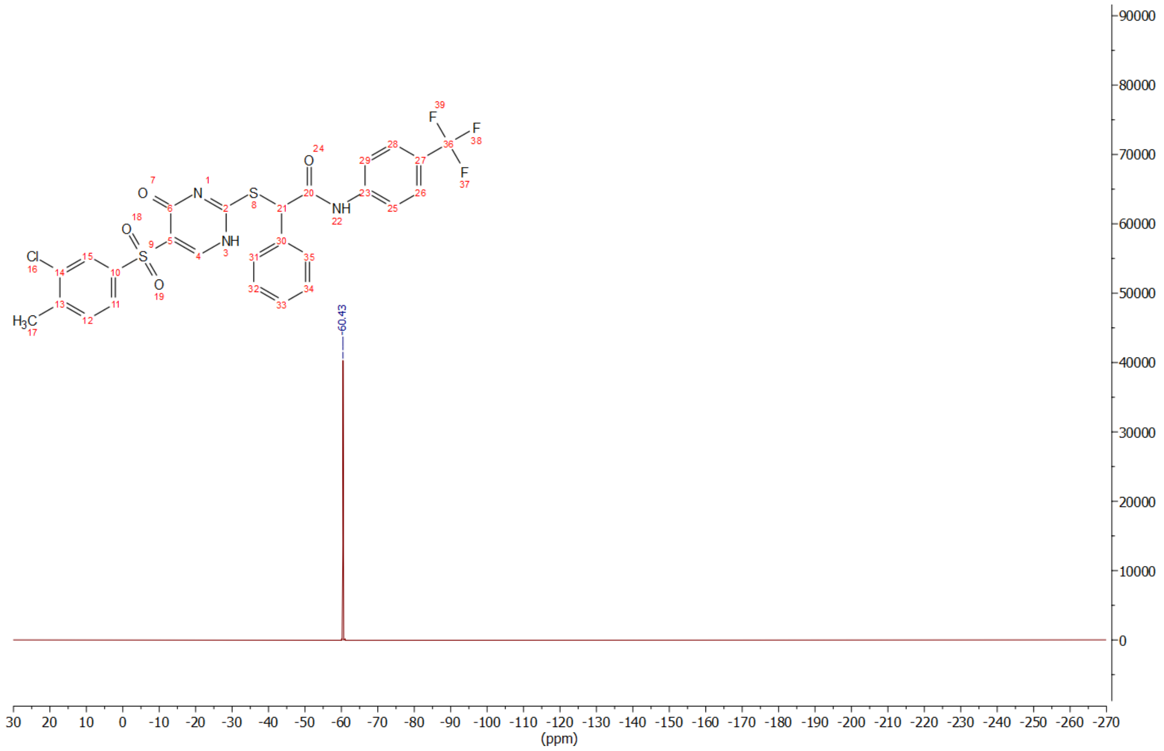

Figure S47. *^{19}F (282 MHz) NMR spectrum in DMSO-d_6 of* ***compound 26***

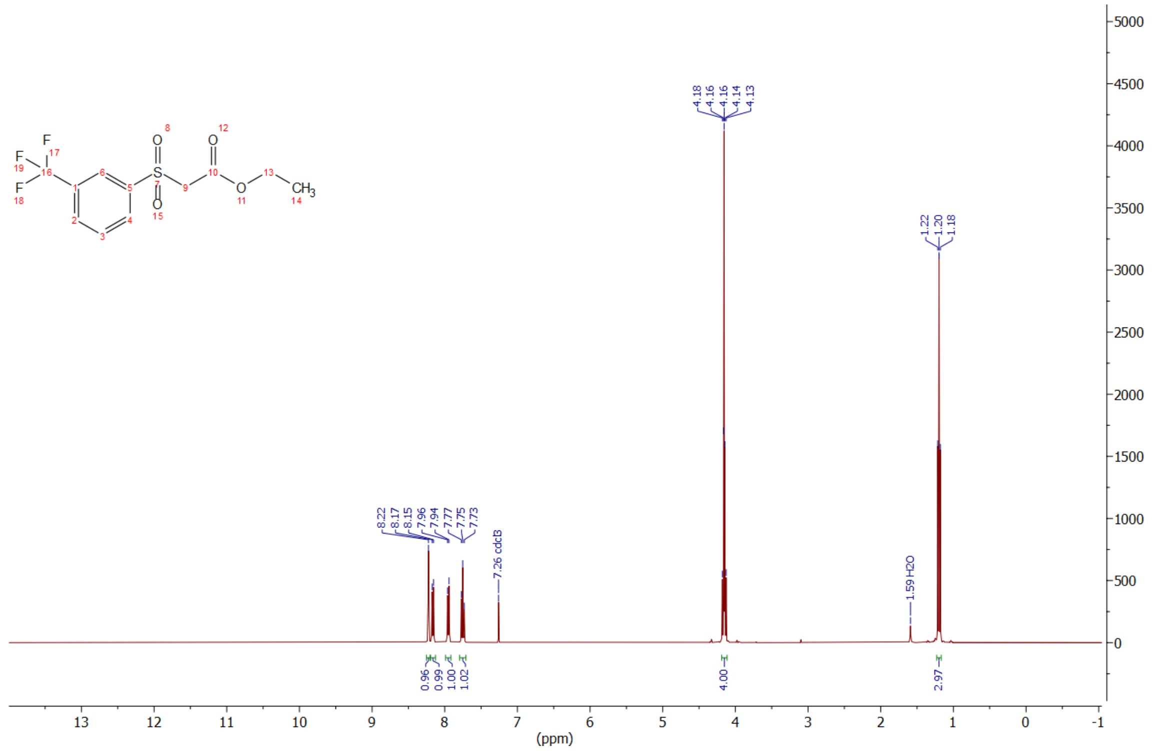

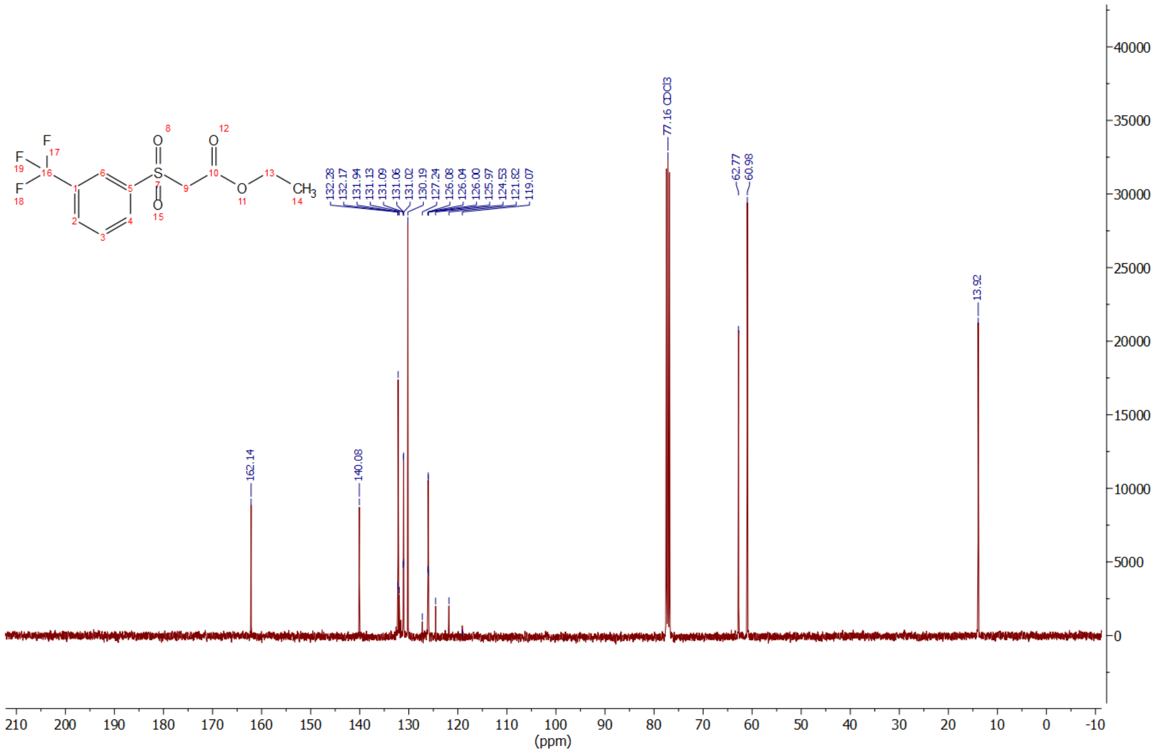

Figure S48. *^{1}H (400 MHz) and $^{13}C\{1H\}$ (101 MHz) NMR spectra in $CDCl_3$ of* ***X11l***

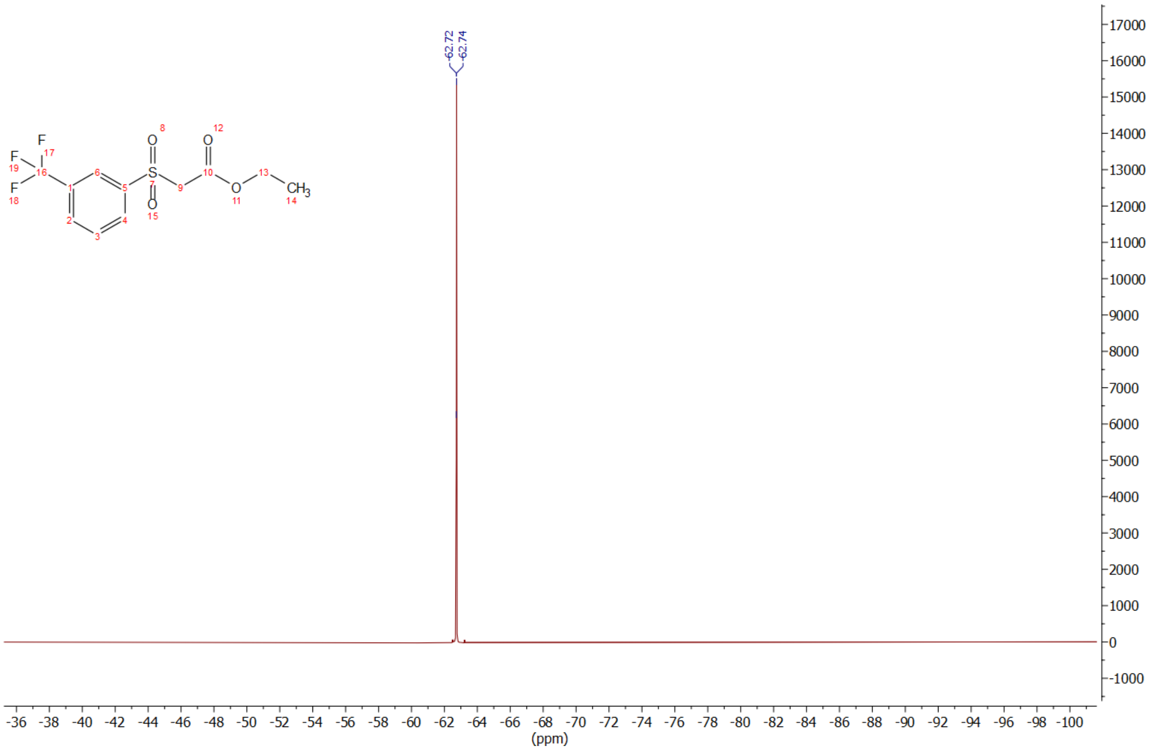

Figure S49. *^{19}F (376 MHz) NMR spectrum in CDCl3 of* ***X11l***

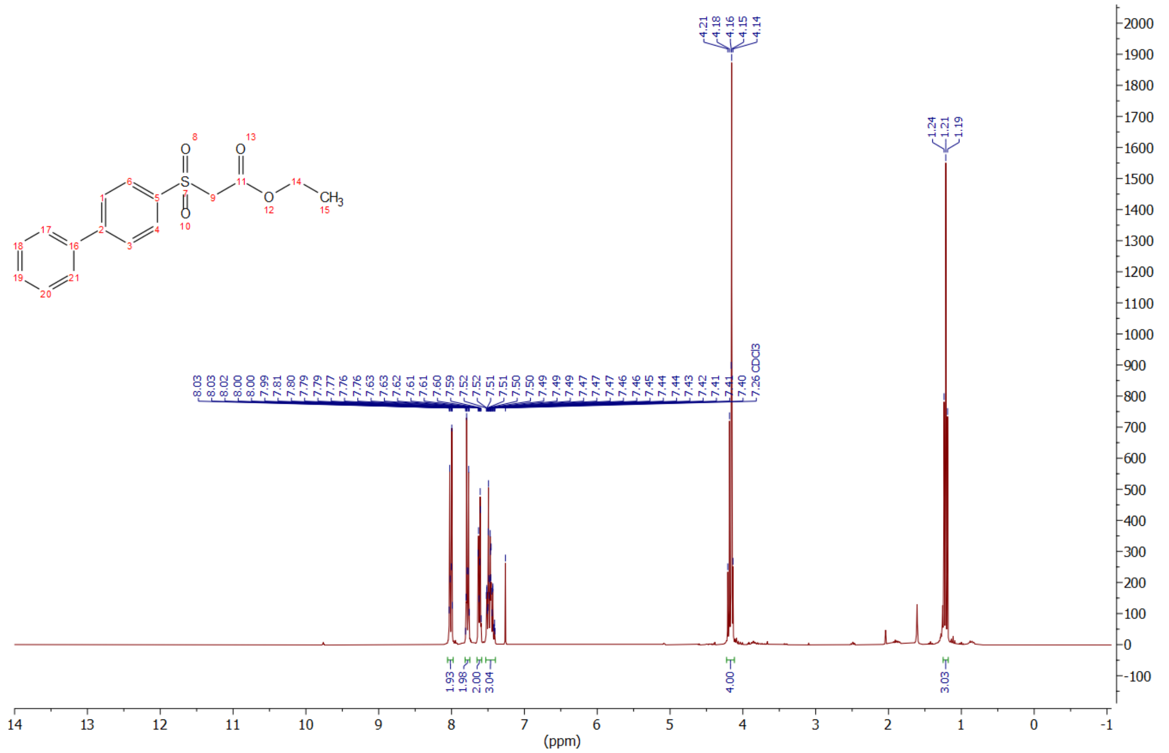

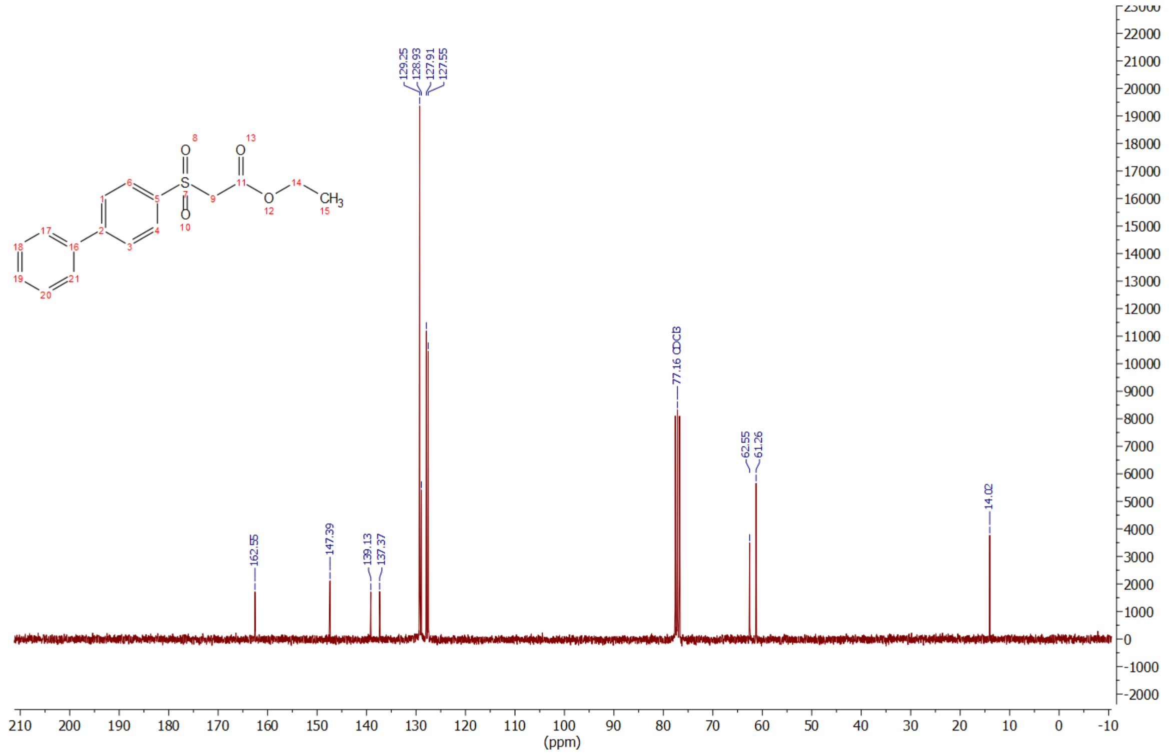

Figure S50. *^{1}H (300 MHz) and $^{13}C\{1H\}$ (75 MHz) NMR spectra in $CDCl_3$ of* ***X11m***

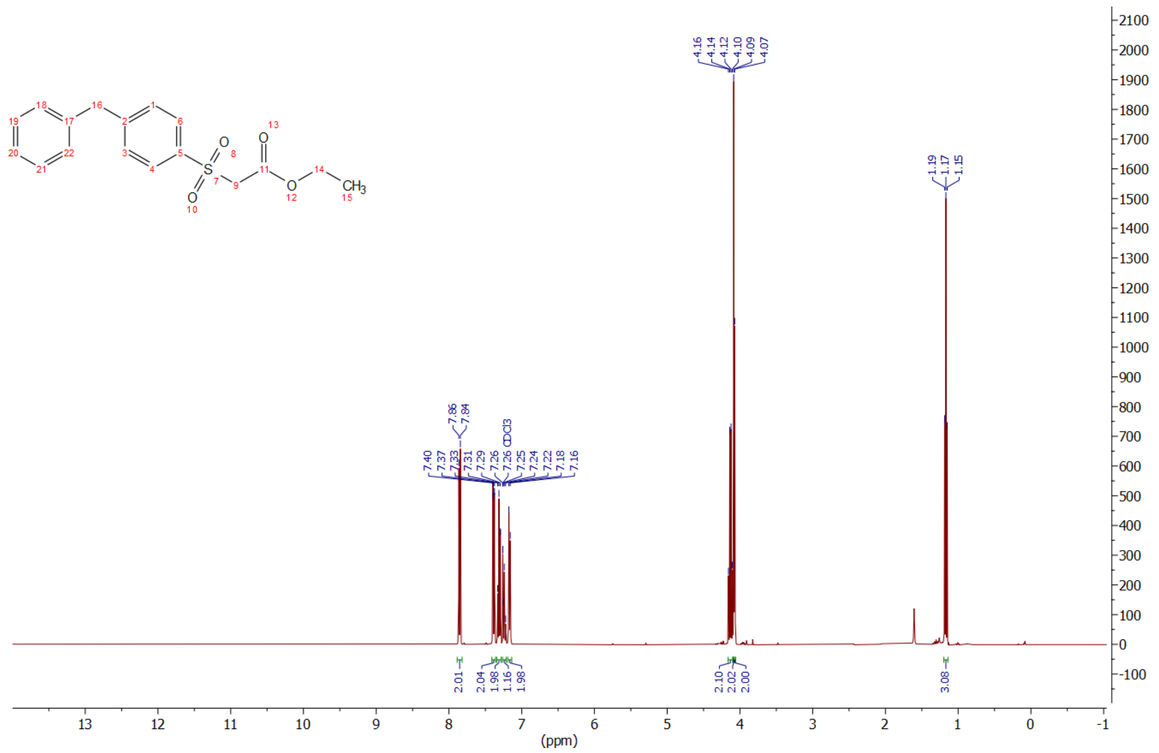

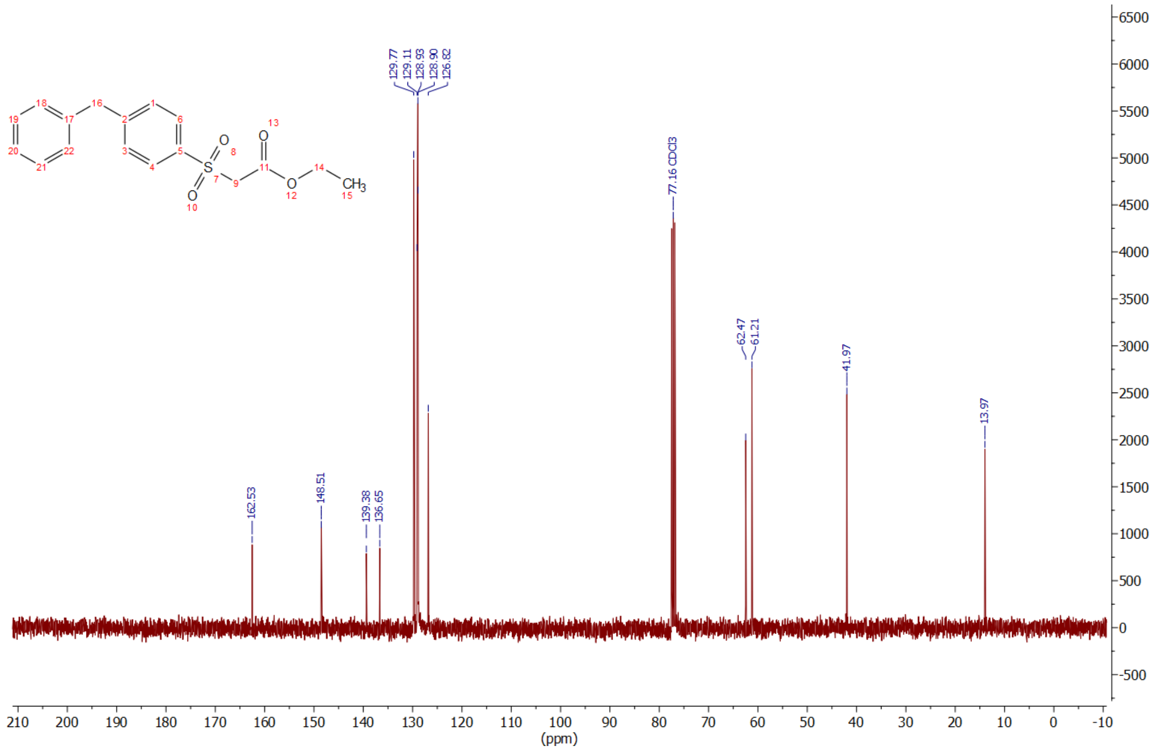

Figure S51. *^{1}H (400 MHz) and ^{13}C{1H} (101 MHz) NMR spectra in $CDCl_3$ of* ***X11n***

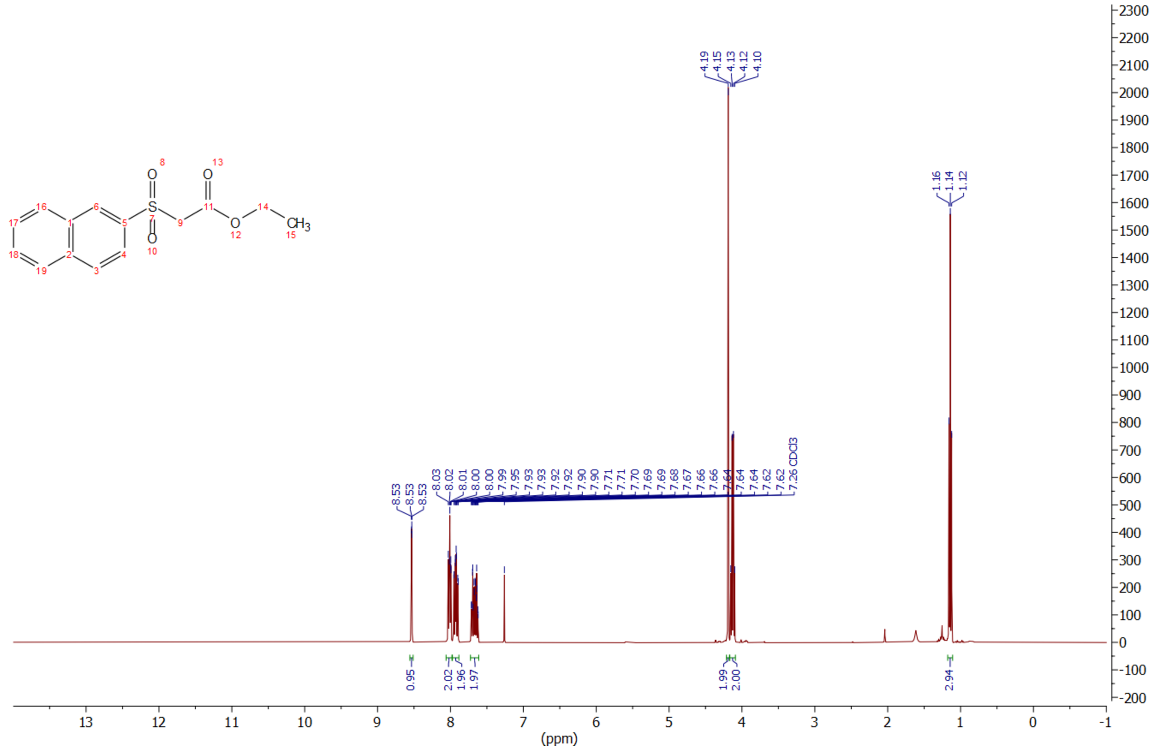

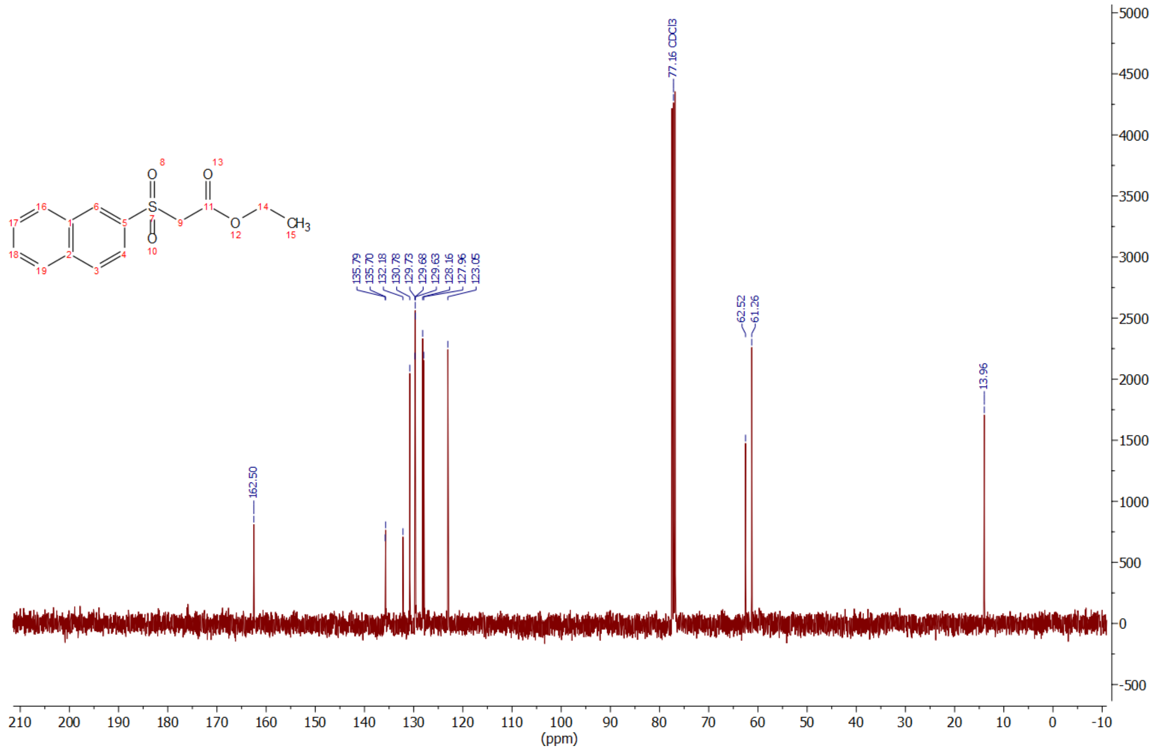

Figure S52. *^{1}H (400 MHz) and $^{13}C\{1H\}$ (101 MHz) NMR spectra in $CDCl_3$ of* ***X11p***

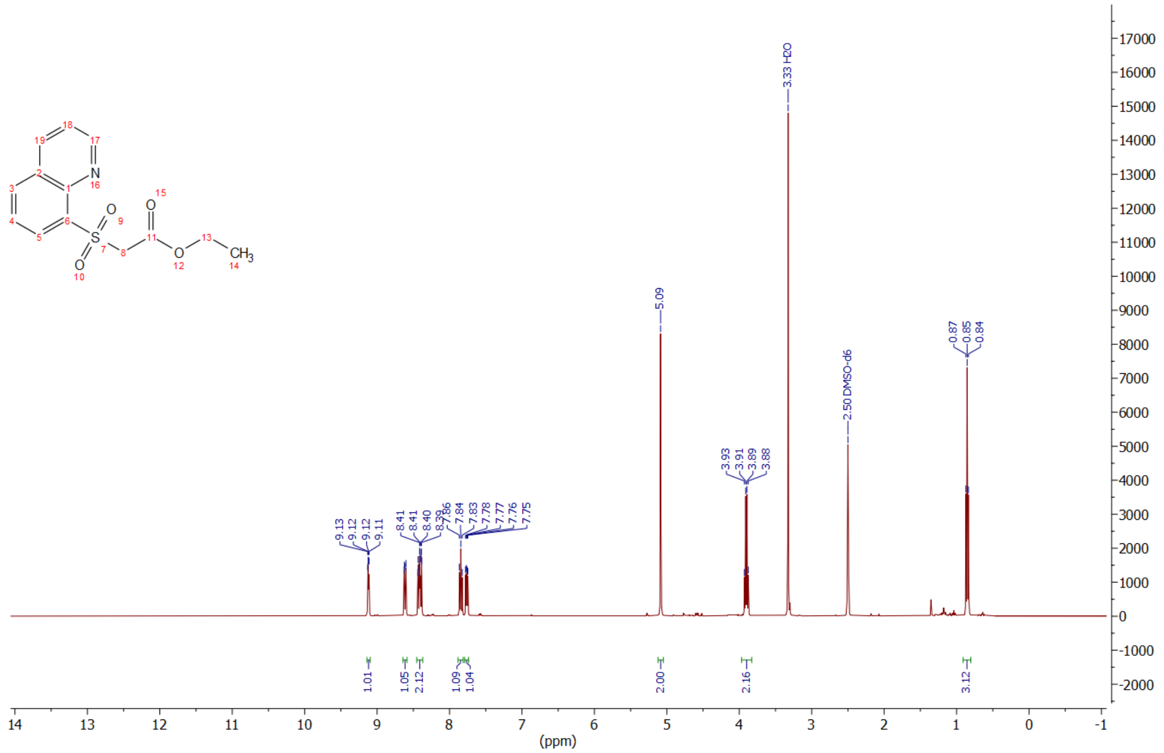

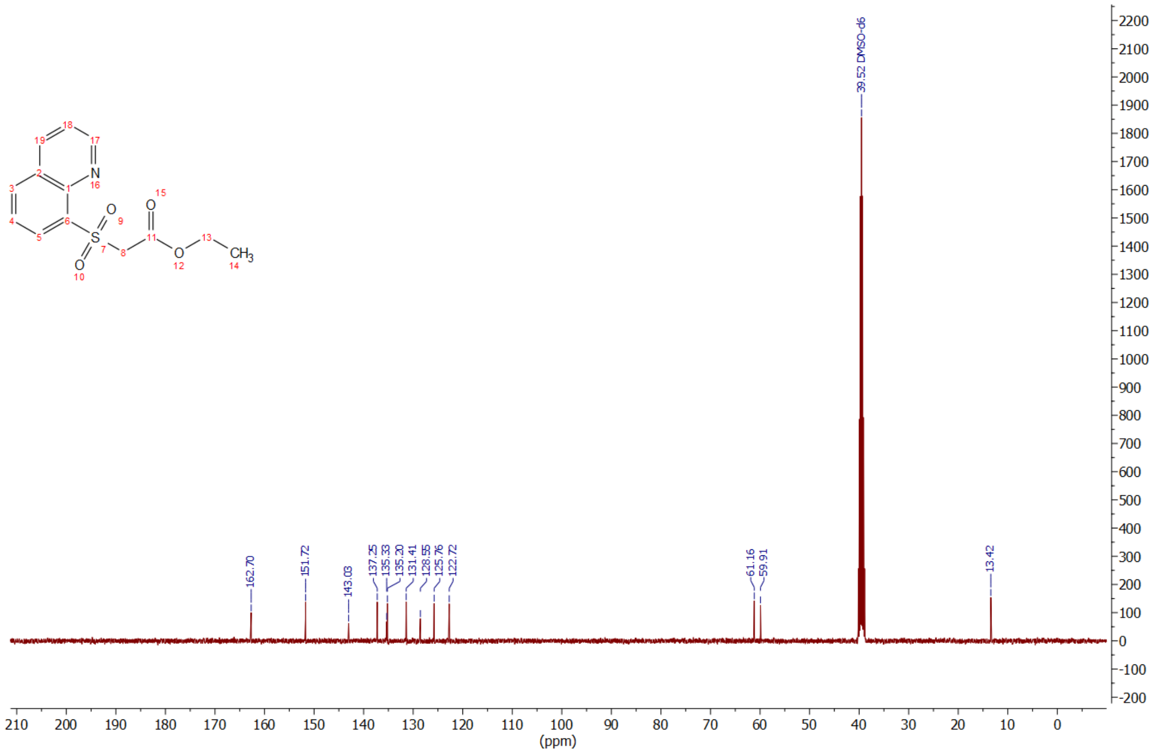

Figure S53. *^{1}H (400 MHz) and $^{13}C\{^{1}H\}$ (101 MHz) NMR spectra in DMSO-d_6 of* ***X11q***

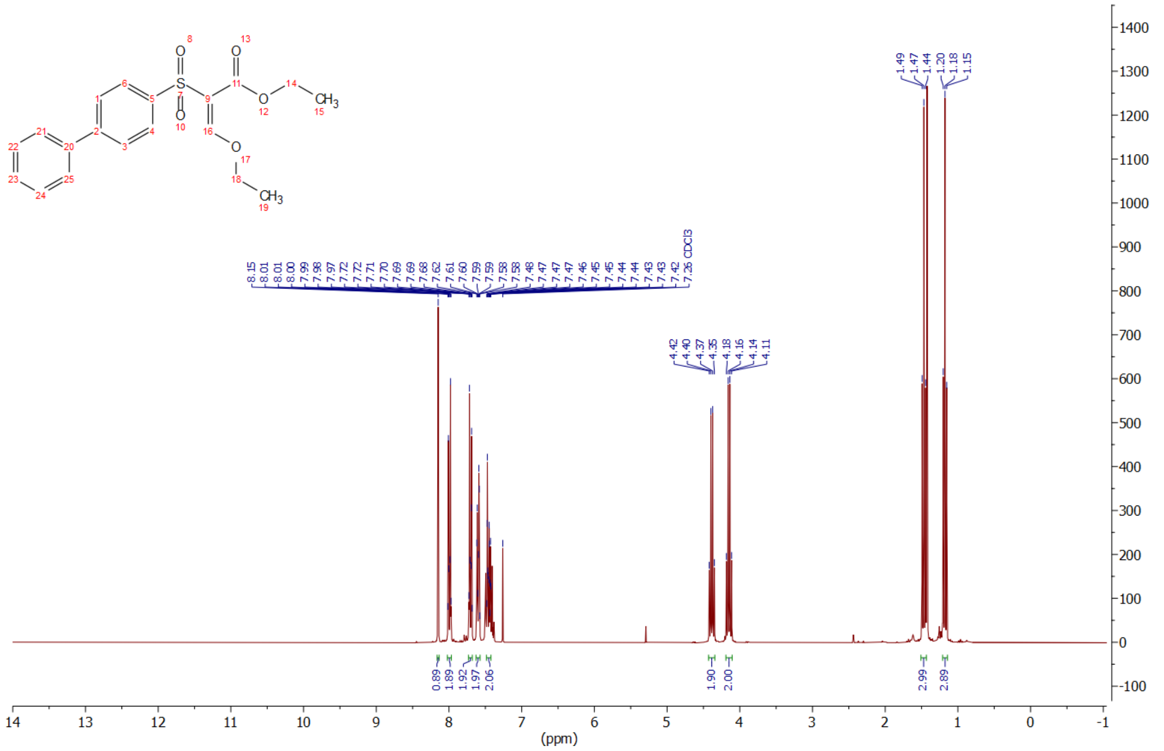

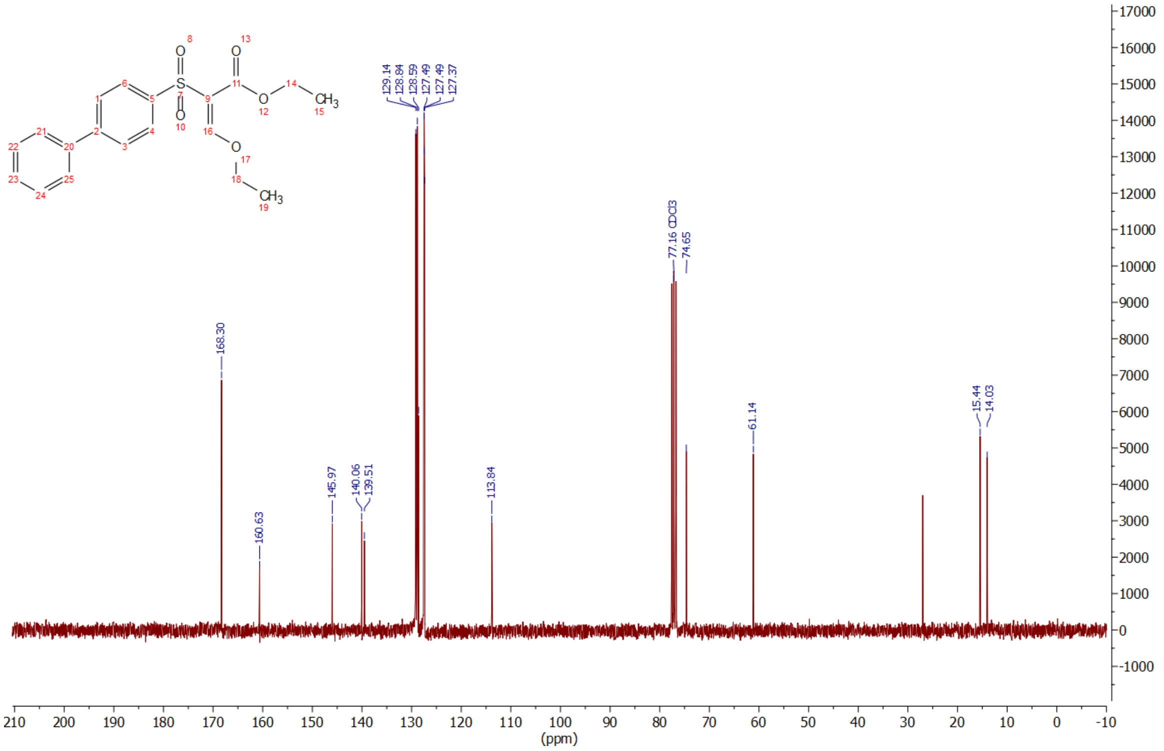

Figure S54. *^{1}H (300 MHz) and ^{13}C{1H} (75 MHz) NMR spectra in CDCl$_3$ of* ***X12m***

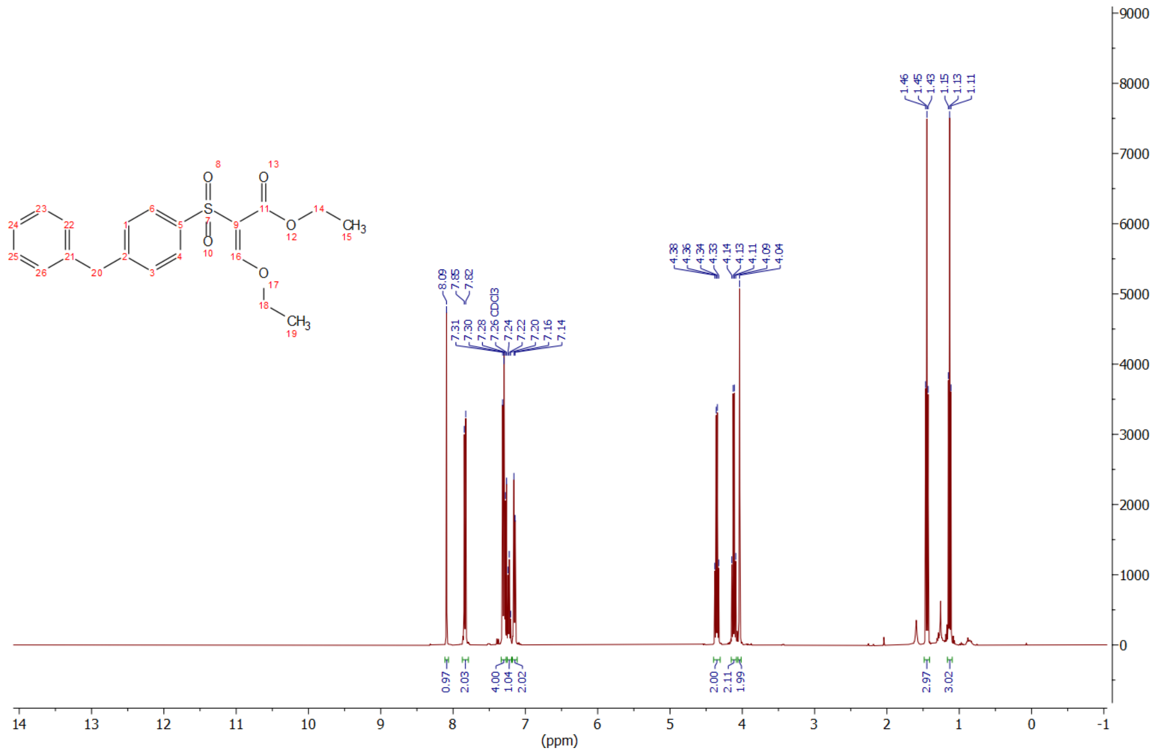

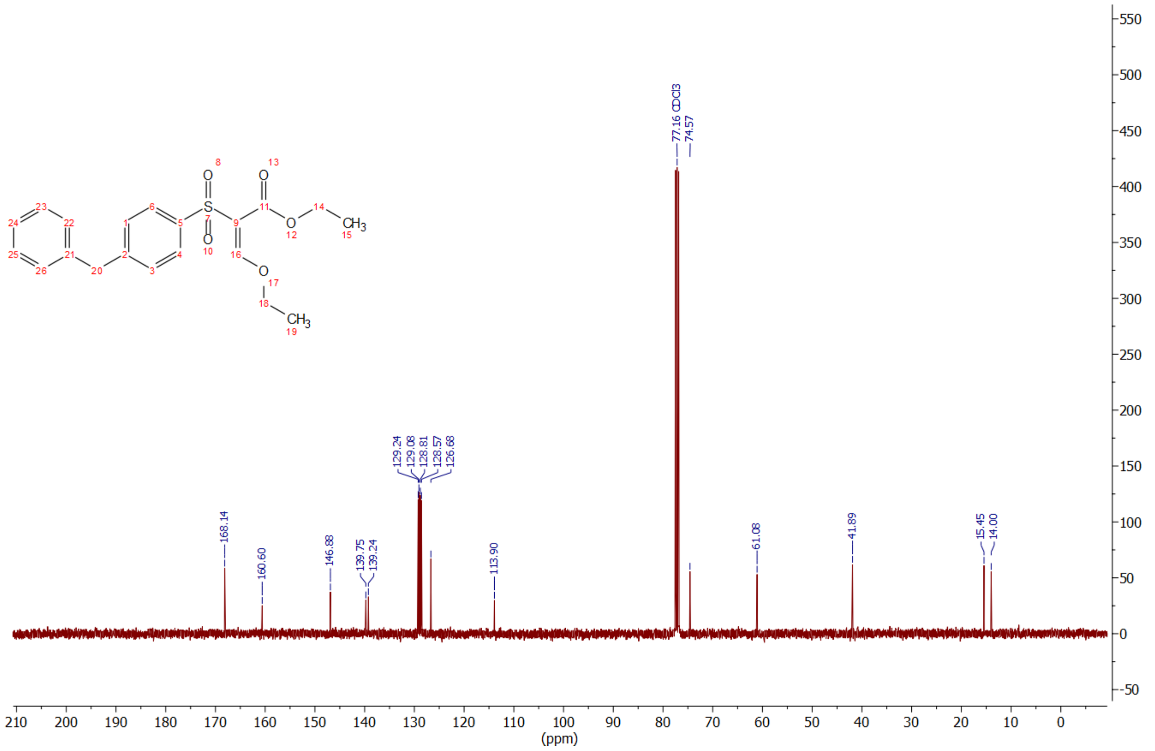

Figure S55. *^{1}H (400 MHz) and $^{13}C\{1H\}$ (101 MHz) NMR spectra in $CDCl_3$ of* ***X12n***

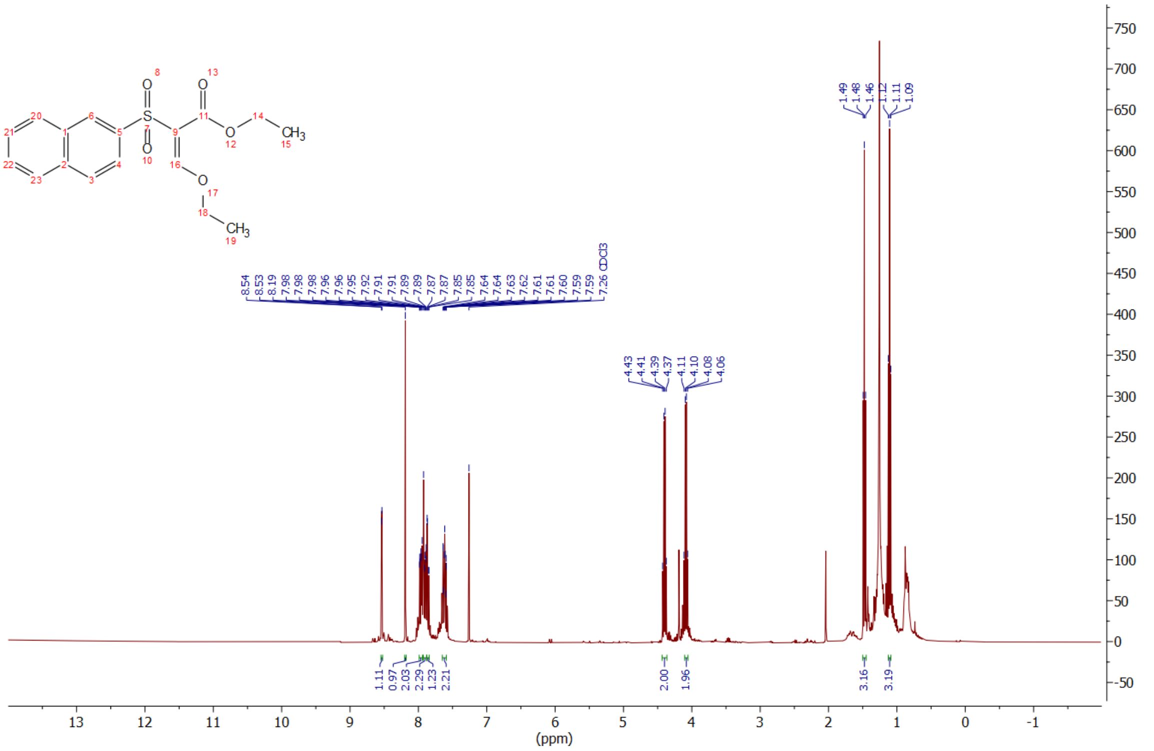

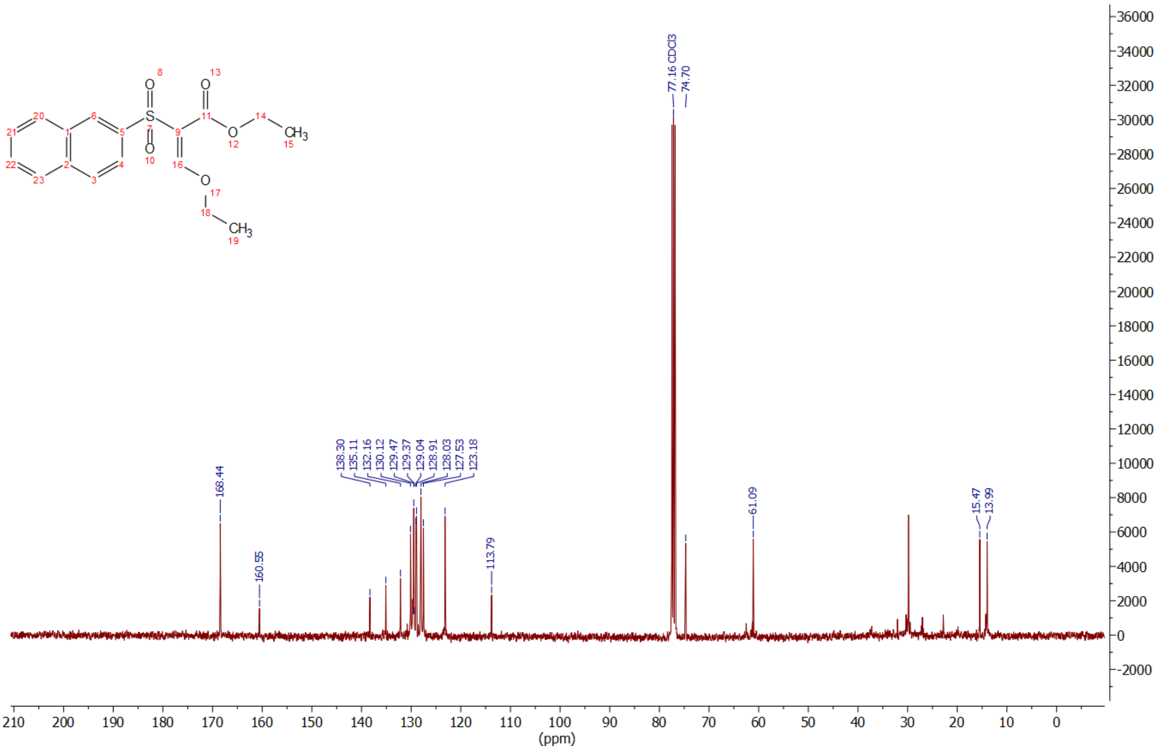

Figure S56. *^{1}H (400 MHz) and $^{13}C\{1H\}$ (101 MHz) NMR spectra in $CDCl_3$ of* ***X12p***

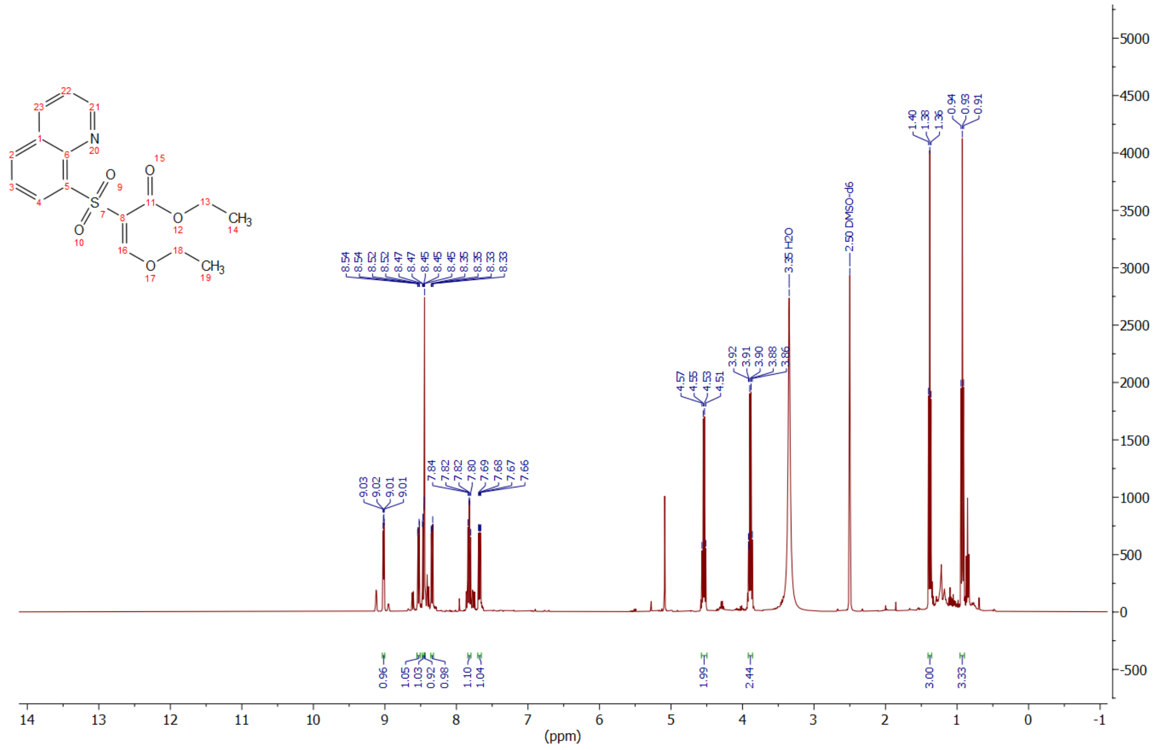

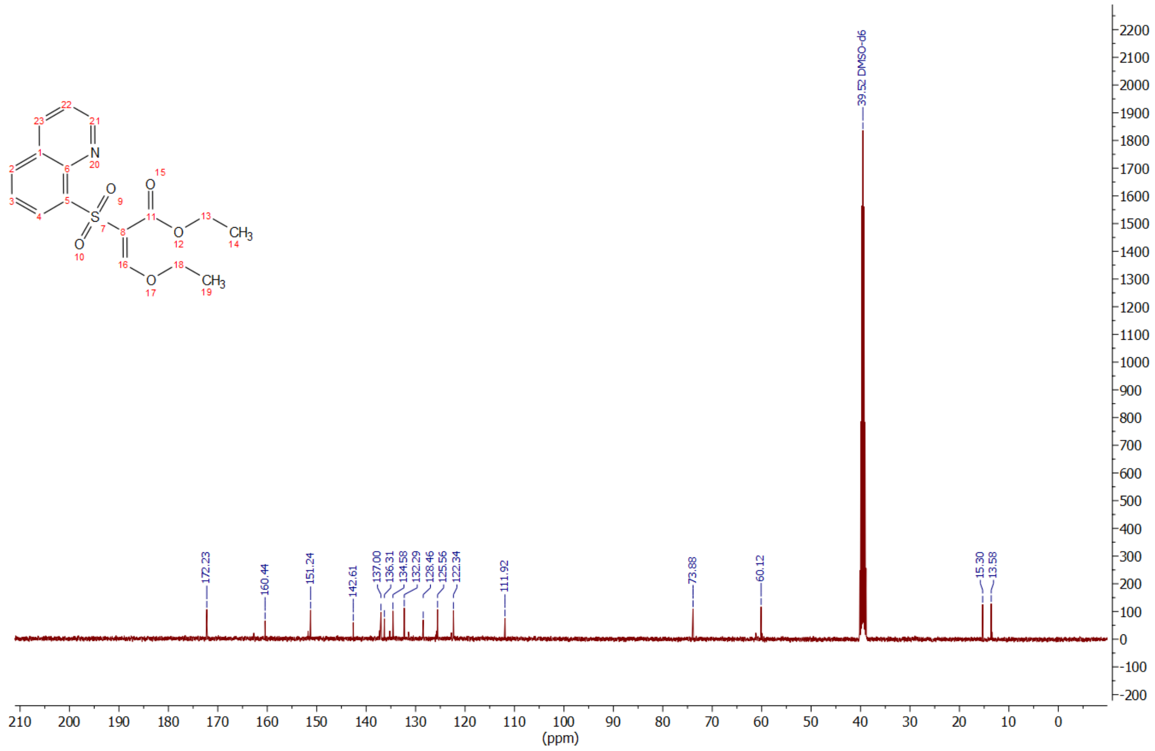

Figure S57. *^{1}H (400 MHz) and $^{13}C\{1H\}$ (101 MHz) NMR spectra in DMSO-d_6 of* ***X12q***

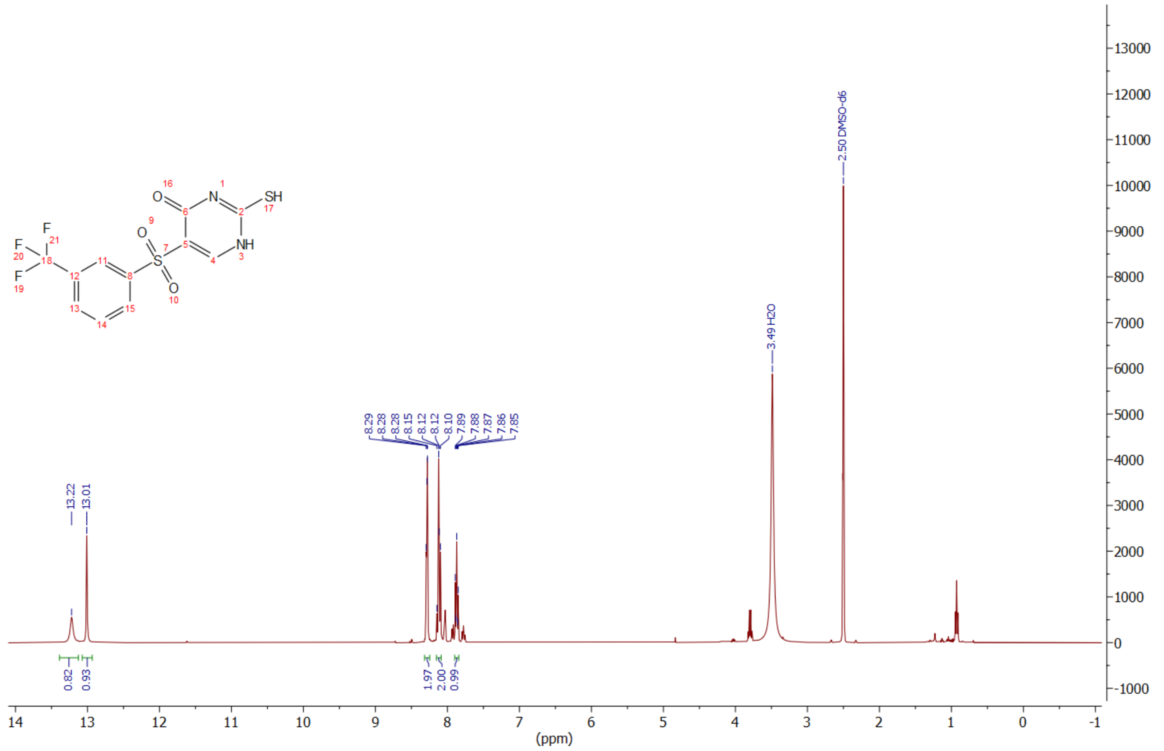

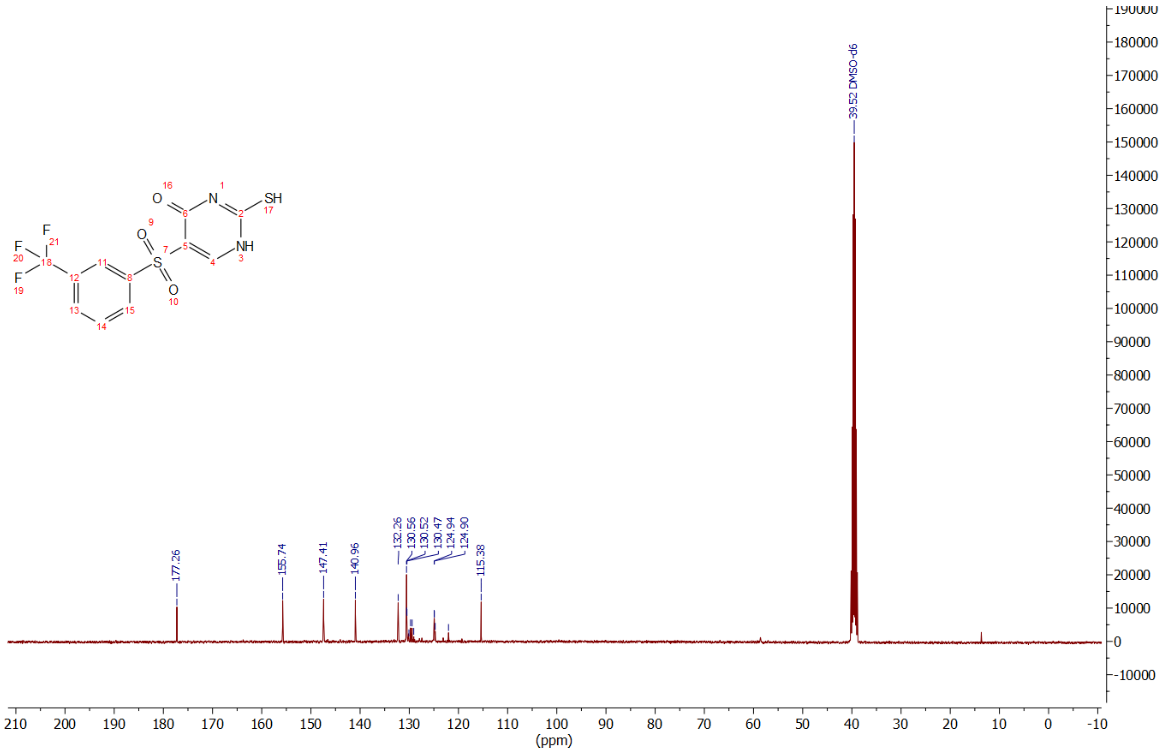

Figure S58. ^{1}H *(400 MHz) and* $^{13}C\{1H\}$ *(101 MHz) NMR spectra in DMSO-d*$_{6}$ *of* ***X13l***

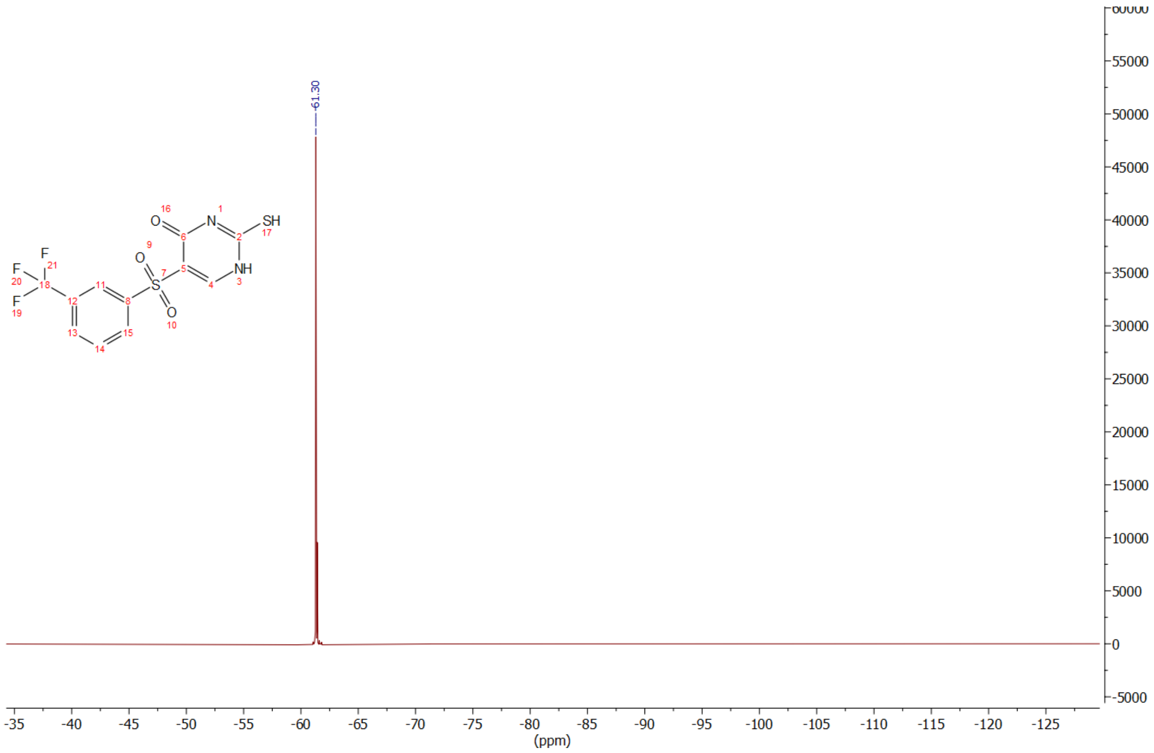

Figure S59. *^{19}F (376 MHz) NMR spectrum in DMSO-d$_6$ of* ***X13l***

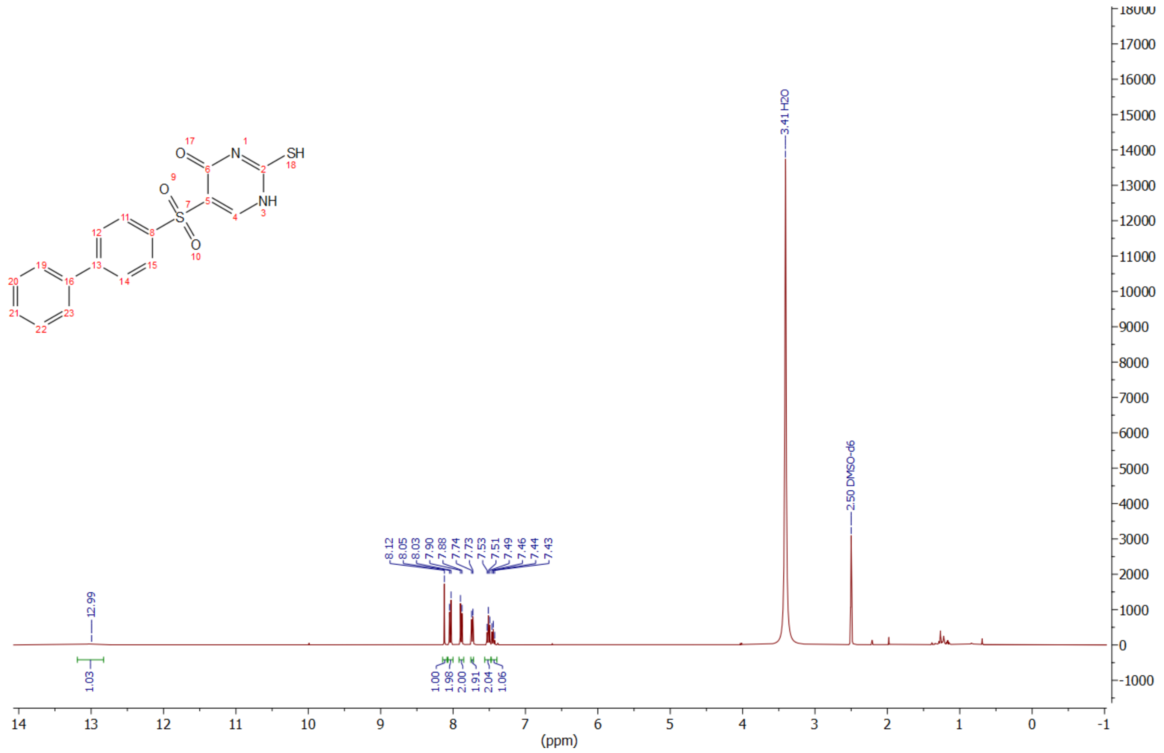

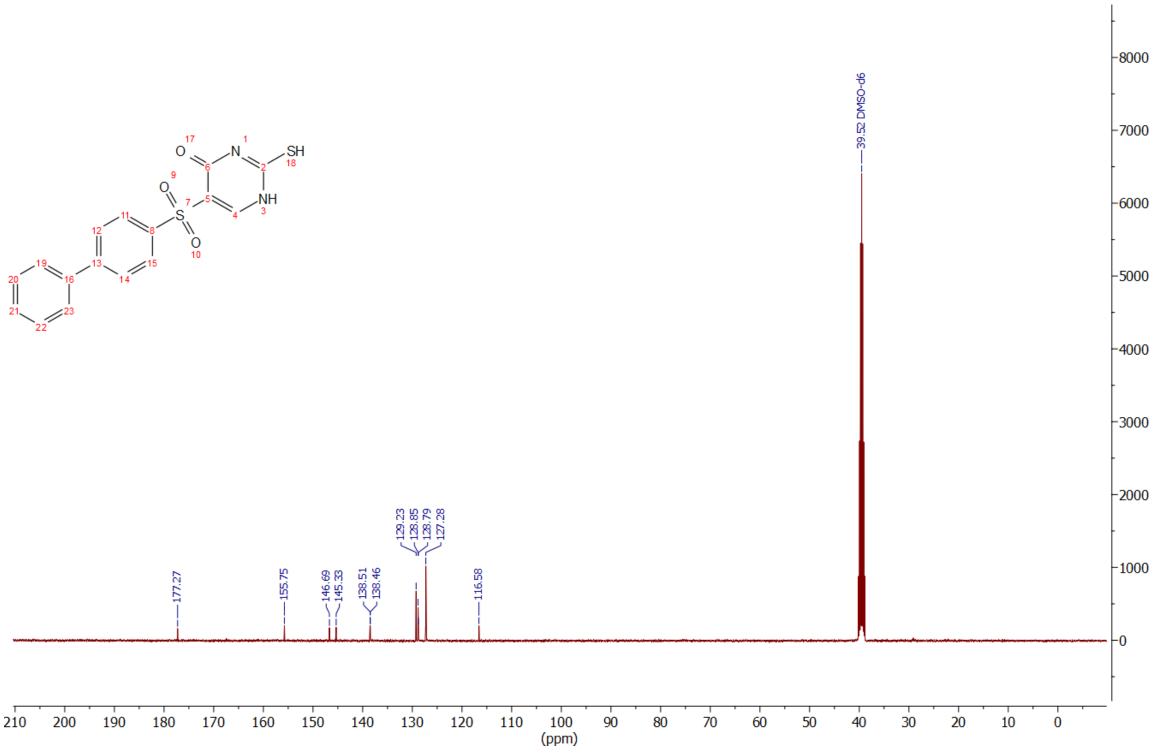

Figure S60. *^{1}H (400 MHz) and ^{13}C{1H} (101 MHz) NMR spectra in DMSO-d_6 of* ***X13m***

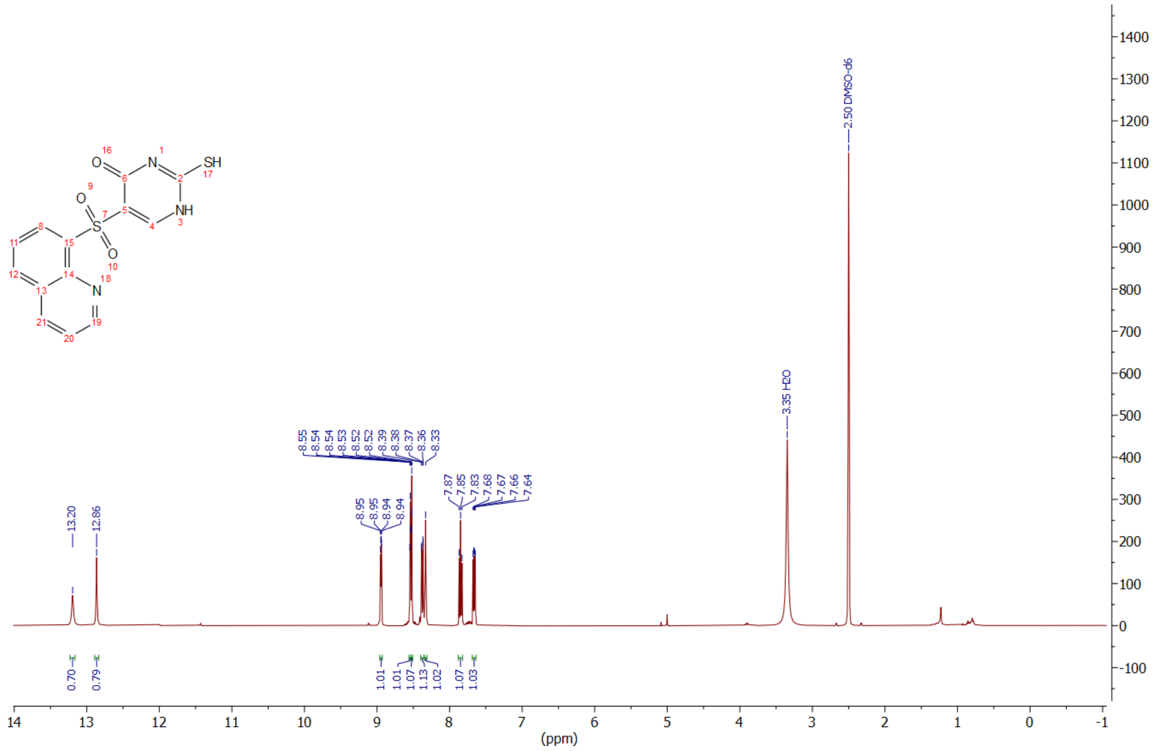

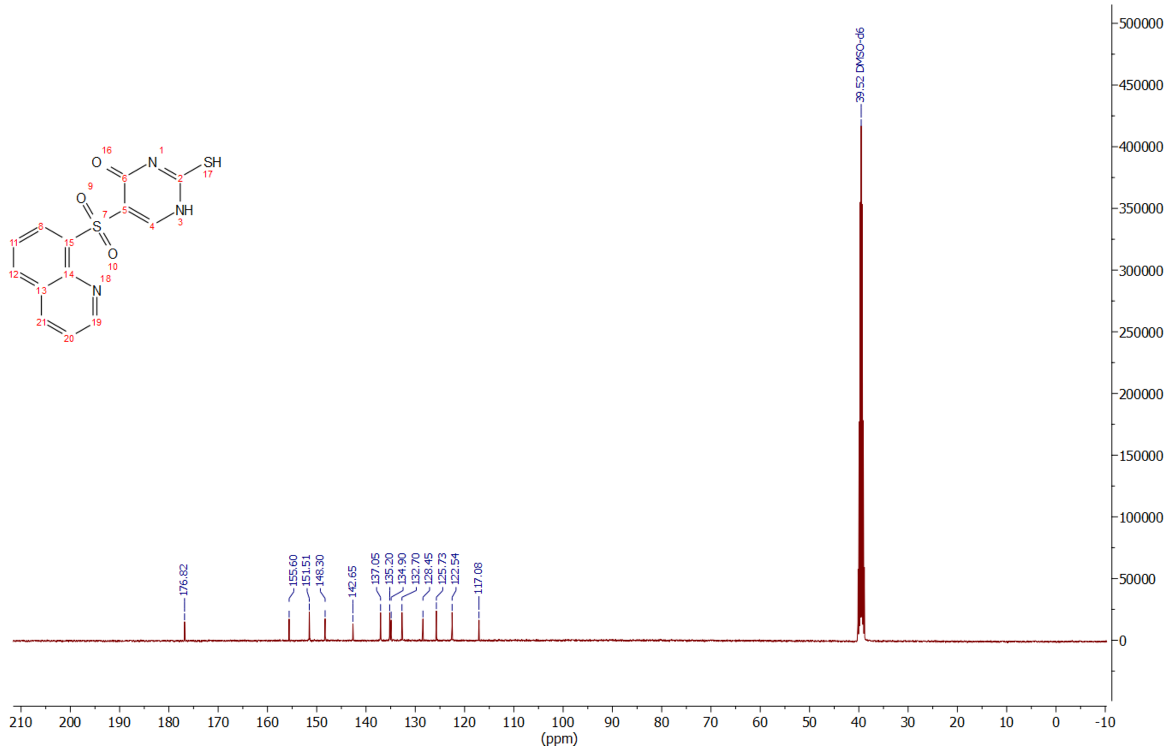

Figure S61. *^{1}H (400 MHz) and ^{13}C{1H} (101 MHz) NMR spectra in DMSO-d_6 of* ***X13q***

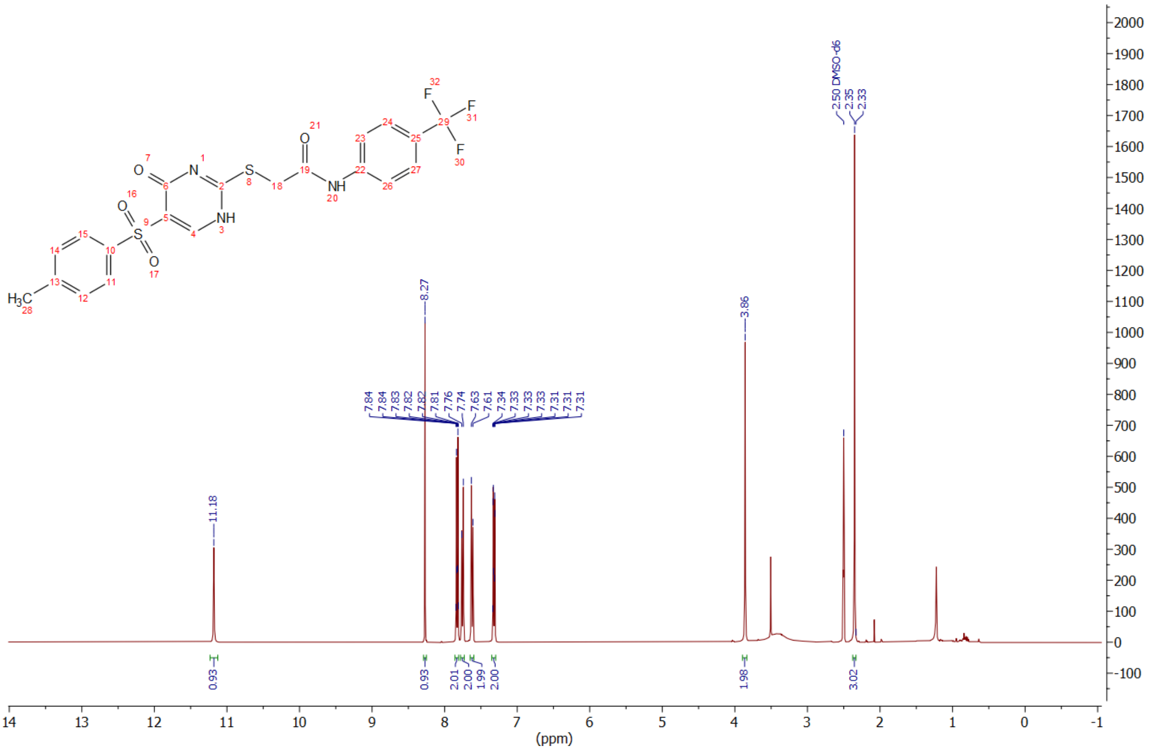

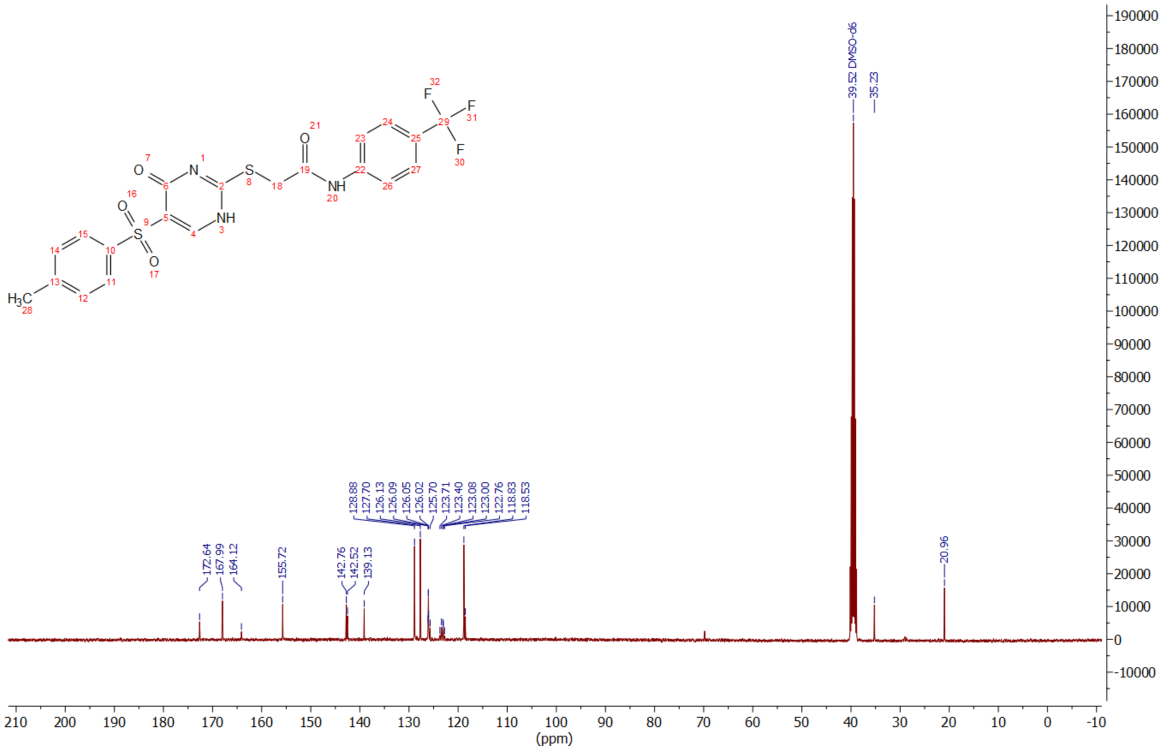

Figure S62. *^{1}H (400 MHz) and $^{13}C\{1H\}$ (101 MHz) NMR spectra in DMSO-d_6 of* ***compound 27***

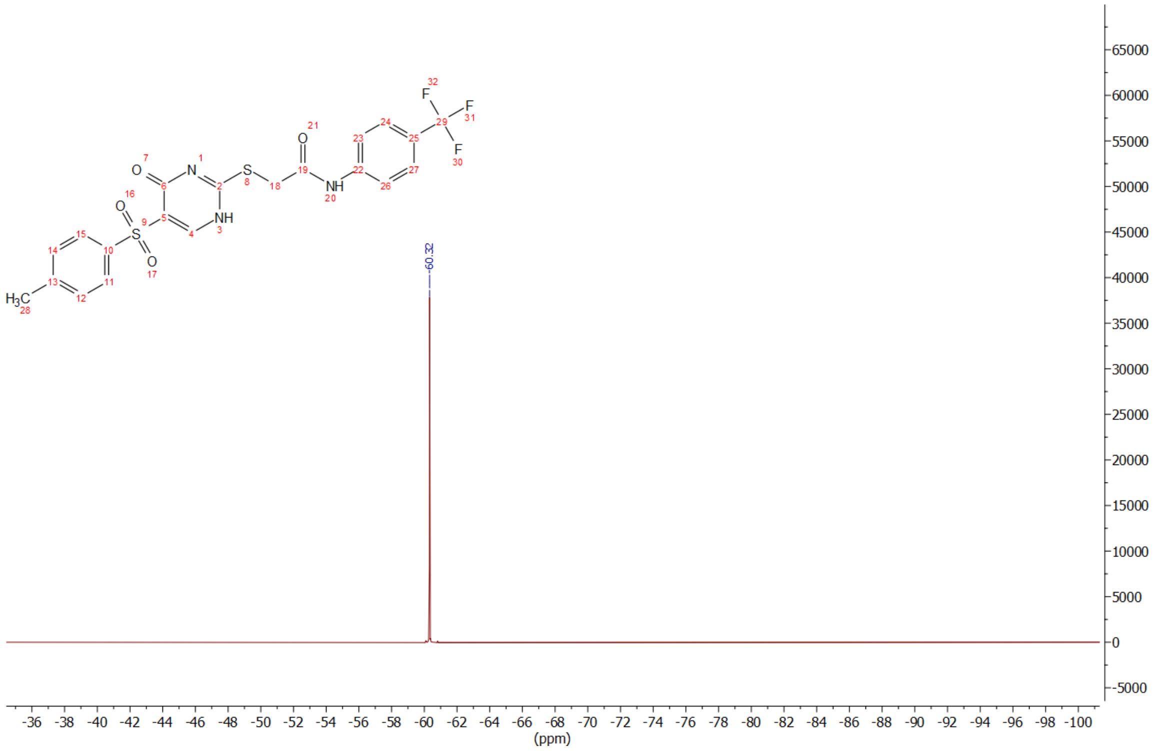

Figure S63. *^{19}F (376 MHz) NMR spectrum in DMSO-d_6 of* ***compound 27***

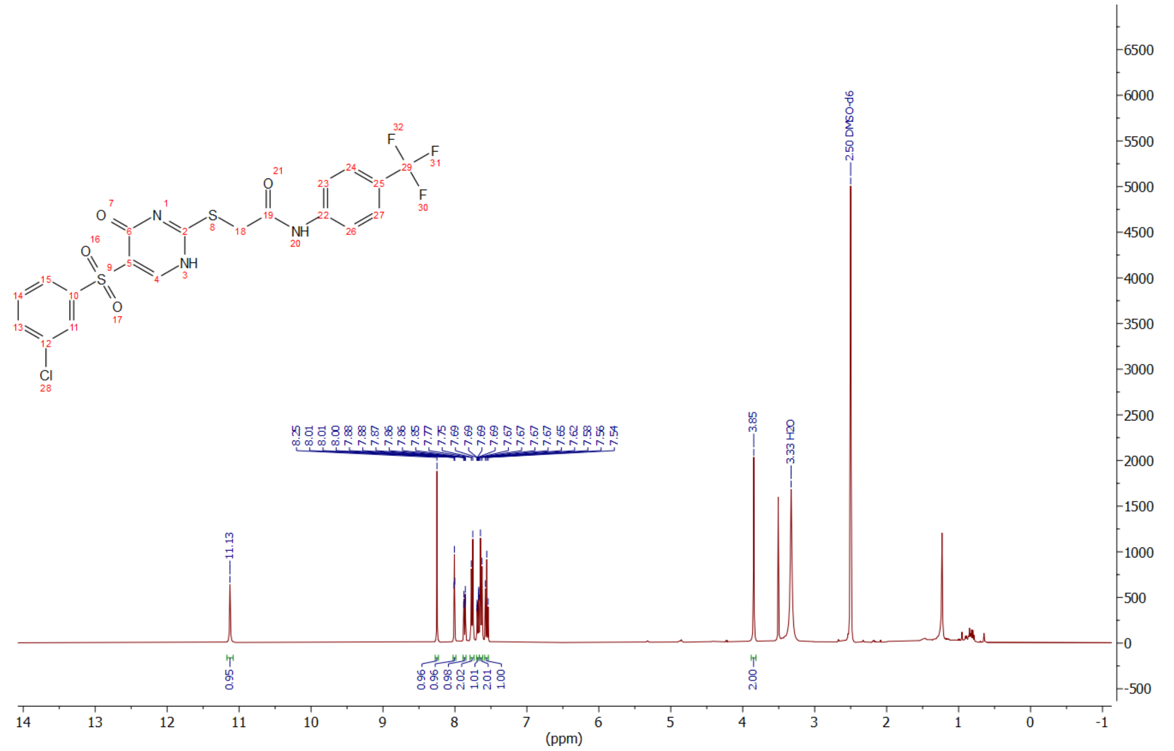

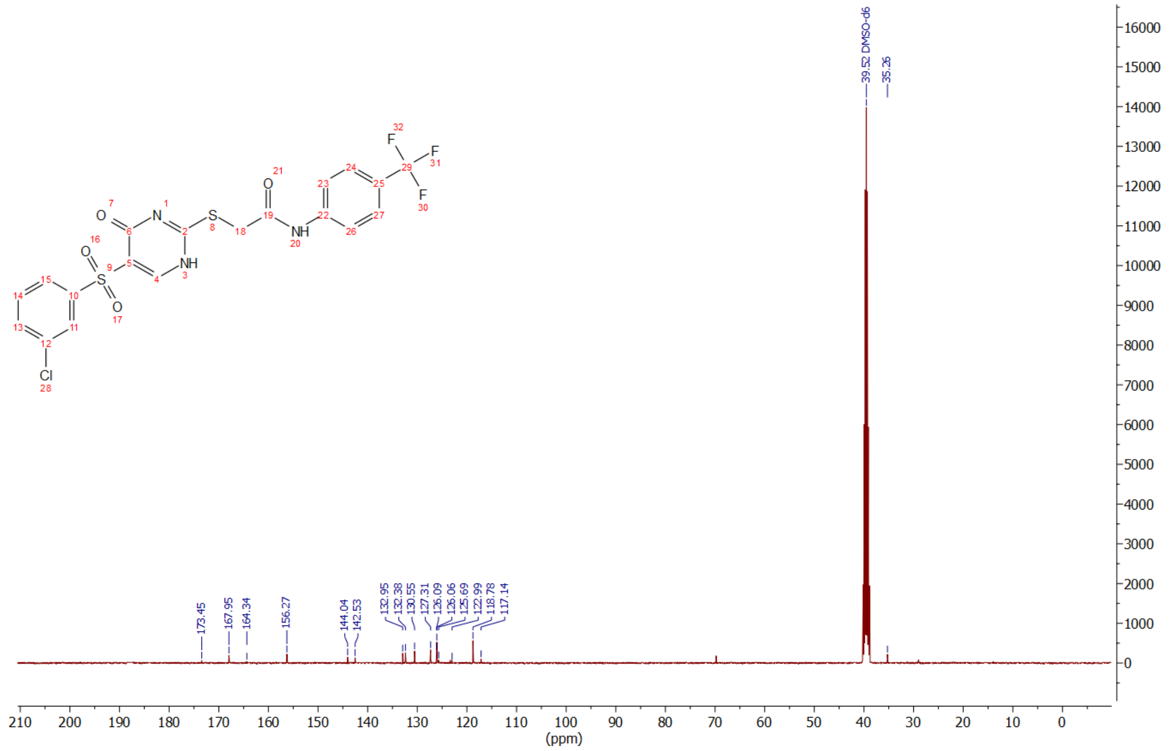

Figure S64. ^{1}*H (400 MHz) and* ^{13}*C{1H} (101 MHz) NMR spectra in DMSO-d*$_6$ *of* ***compound 28***

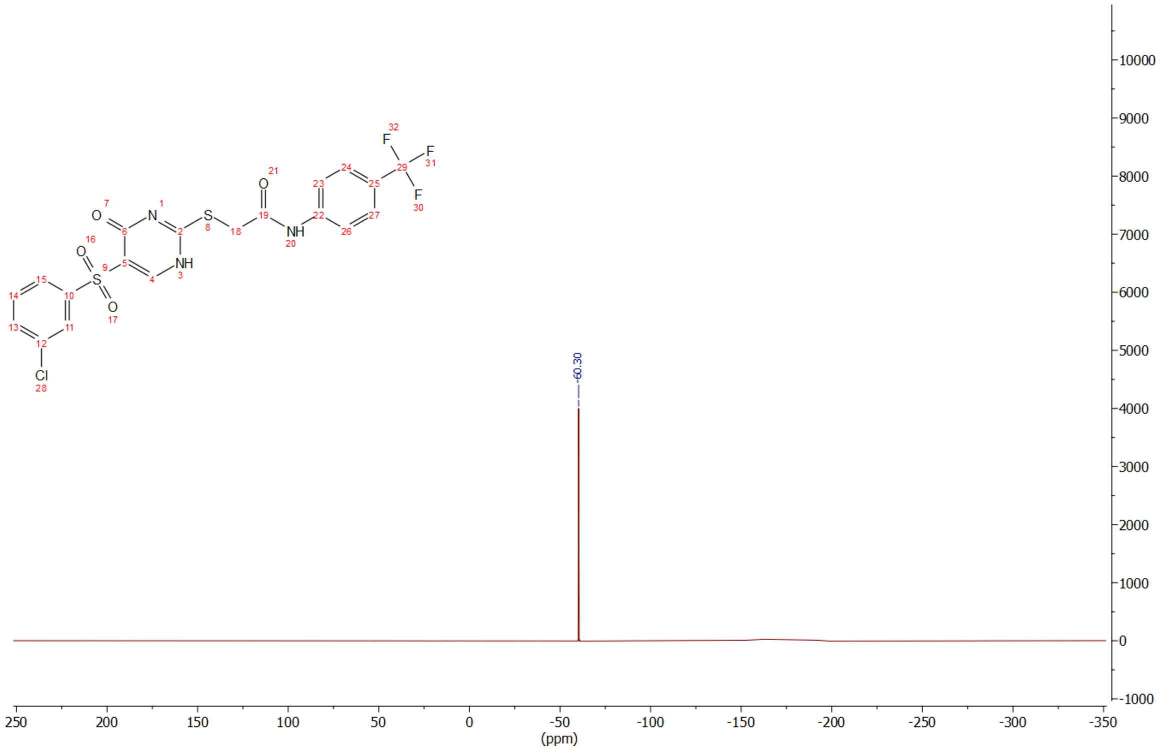

Figure S65. *^{19}F (377 MHz) NMR spectrum in DMSO-d_6 of* ***compound 28***

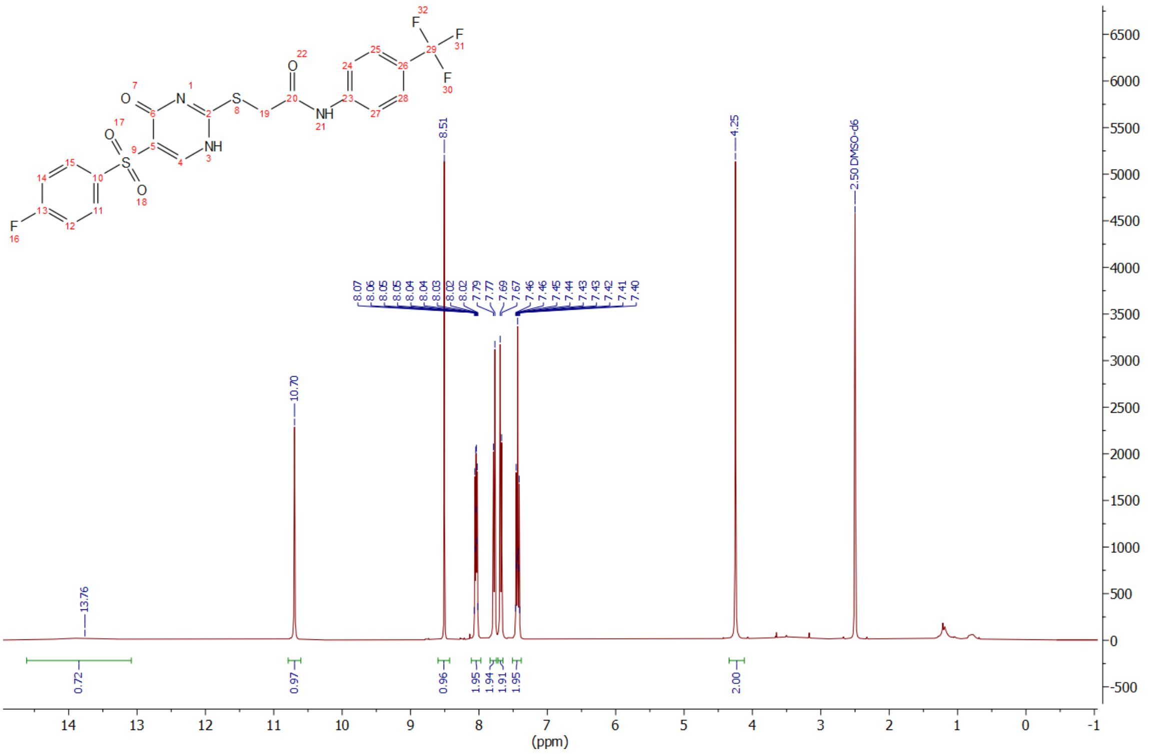

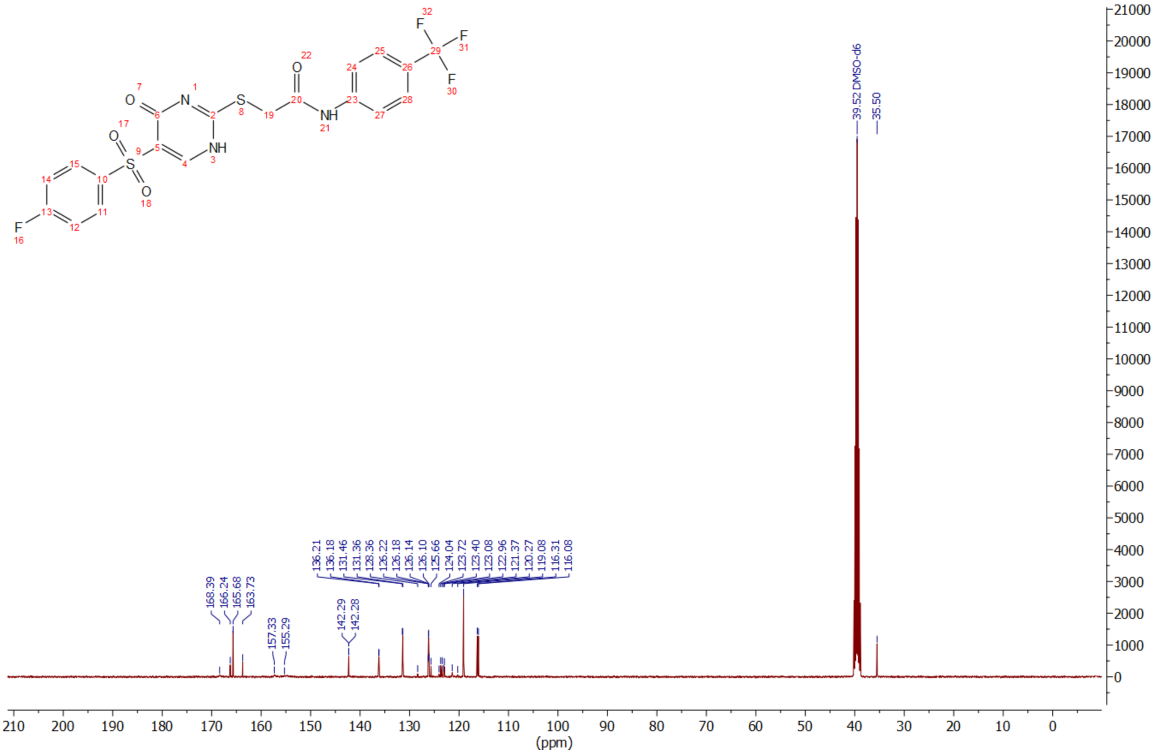

Figure S66. ^{1}H *(400 MHz) and* $^{13}C\{1H\}$ *(101 MHz) NMR spectra in DMSO-d*$_6$ *of* ***compound 29***

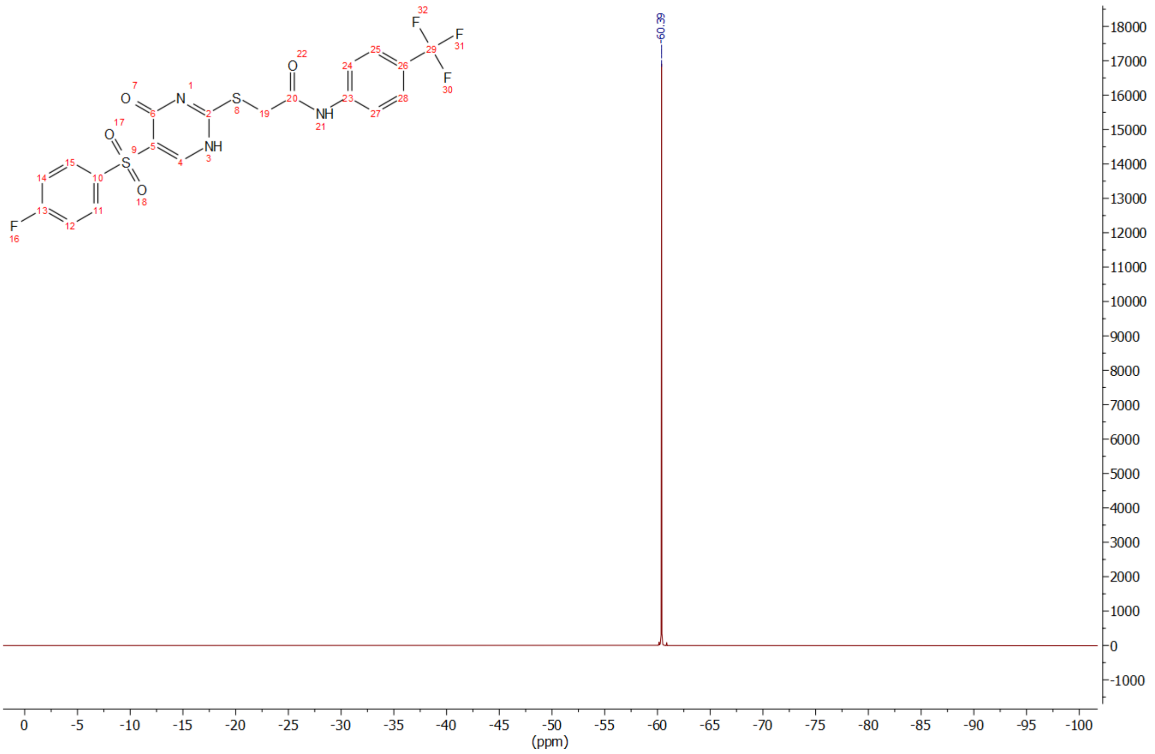

Figure S67. *^{19}F (377 MHz) NMR spectrum in DMSO-d_6 of* ***compound 29***

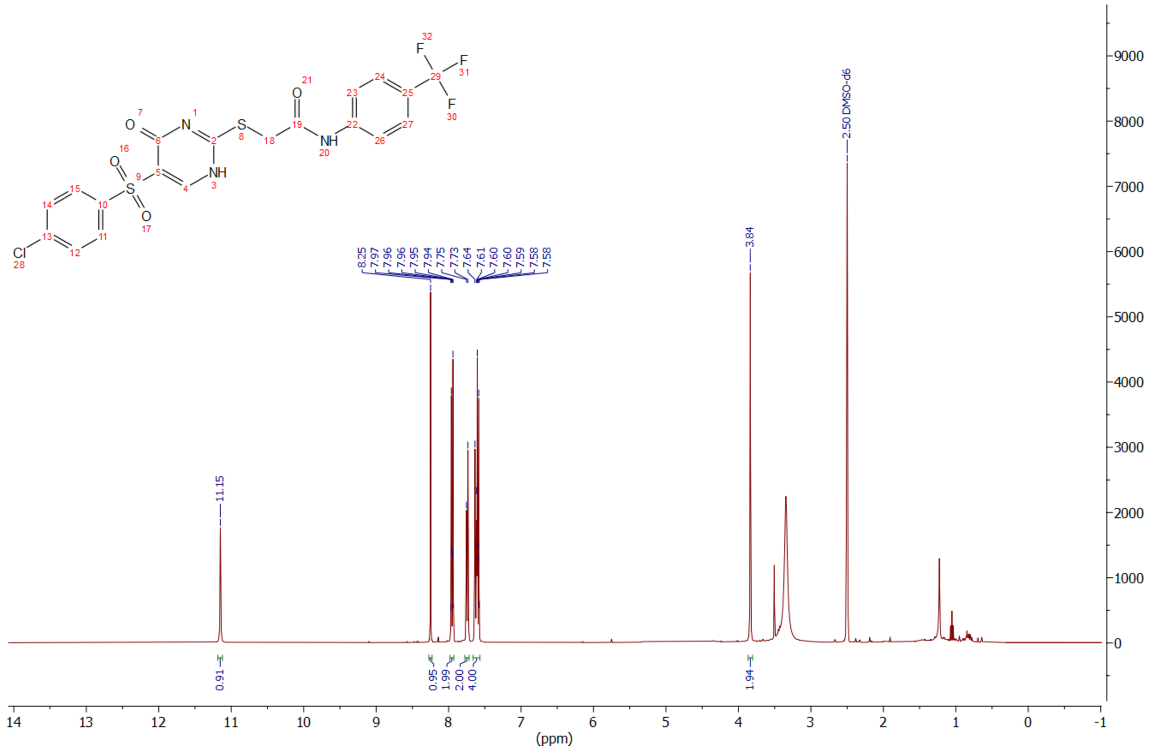

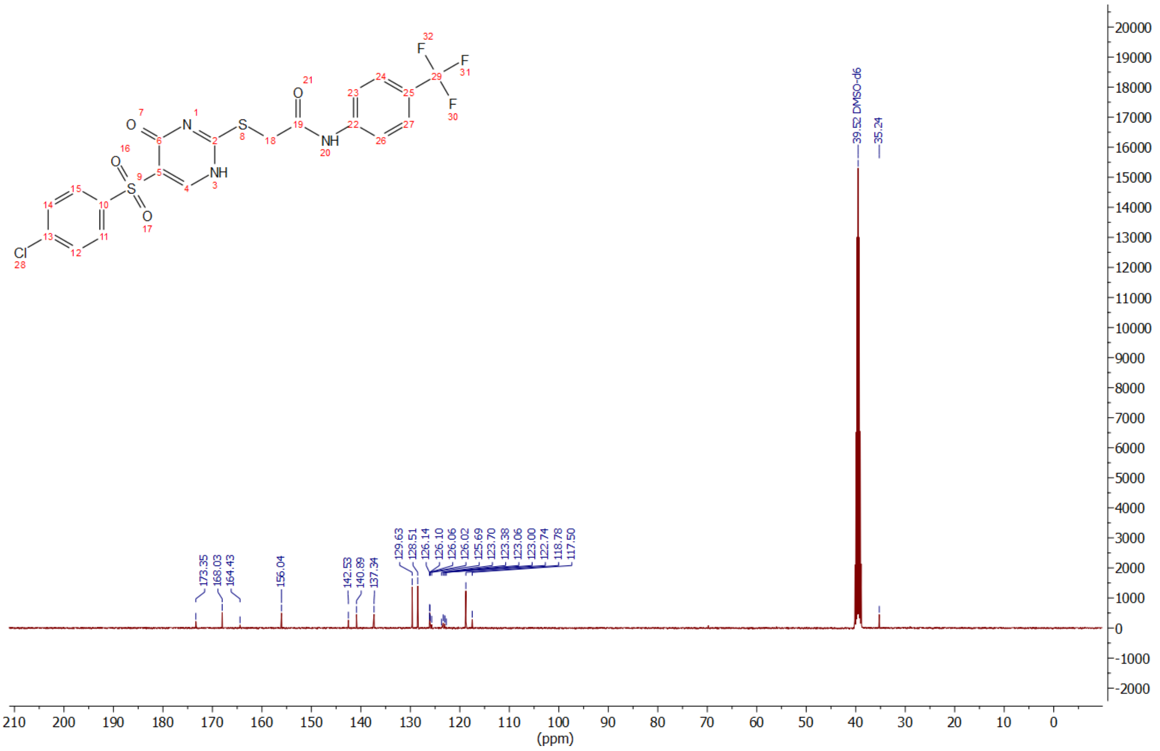

Figure S68. ^{1}H *(400 MHz) and* $^{13}C\{1H\}$ *(101 MHz) NMR spectra in DMSO-d*$_6$ *of* ***compound 30***

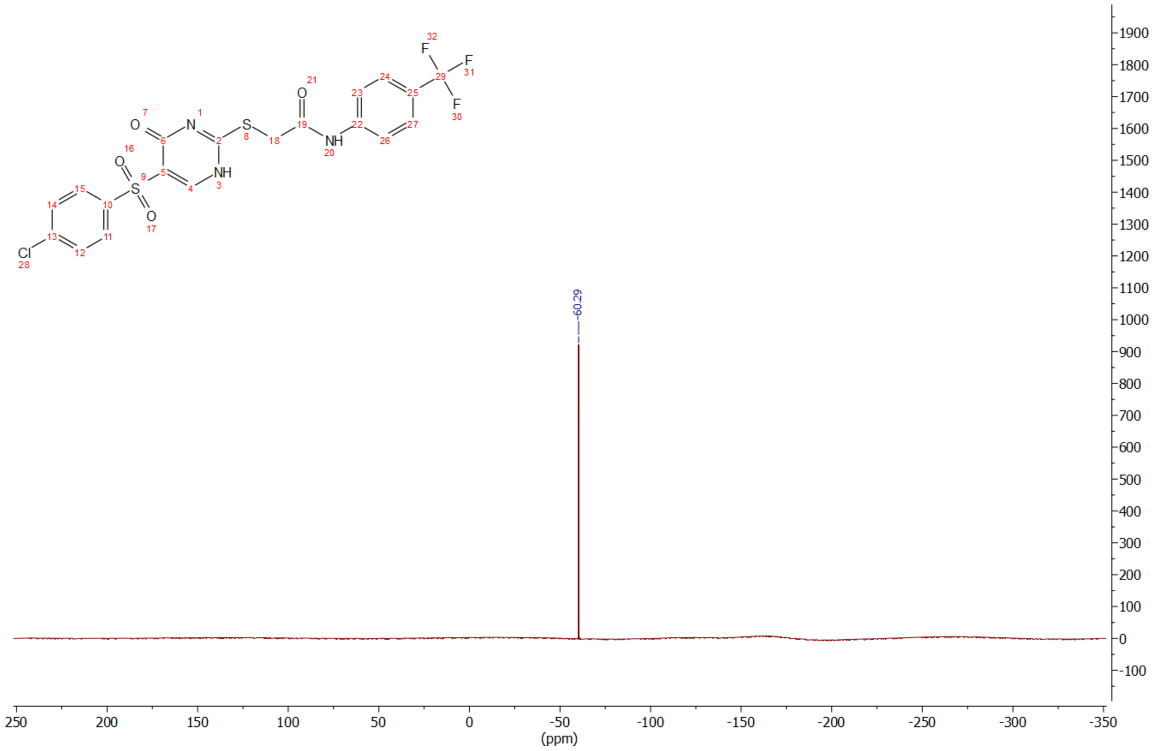

Figure S69. *^{19}F (377 MHz) NMR spectrum in DMSO-d_6 of* ***compound 30***

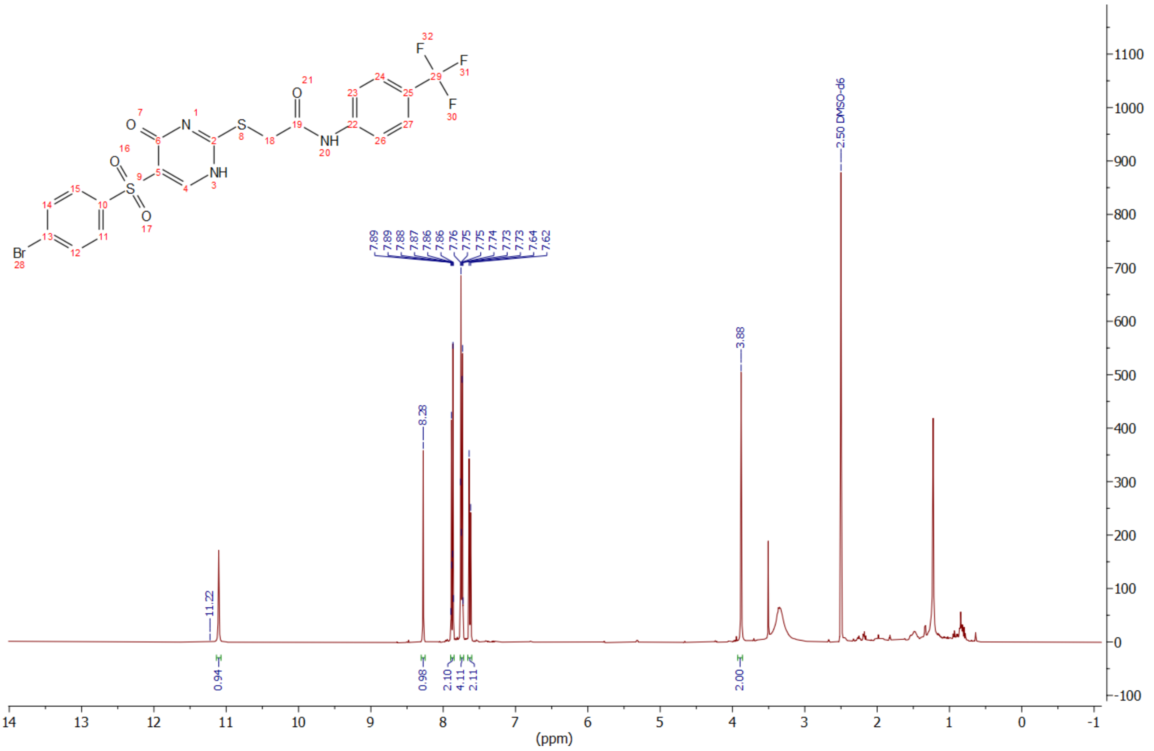

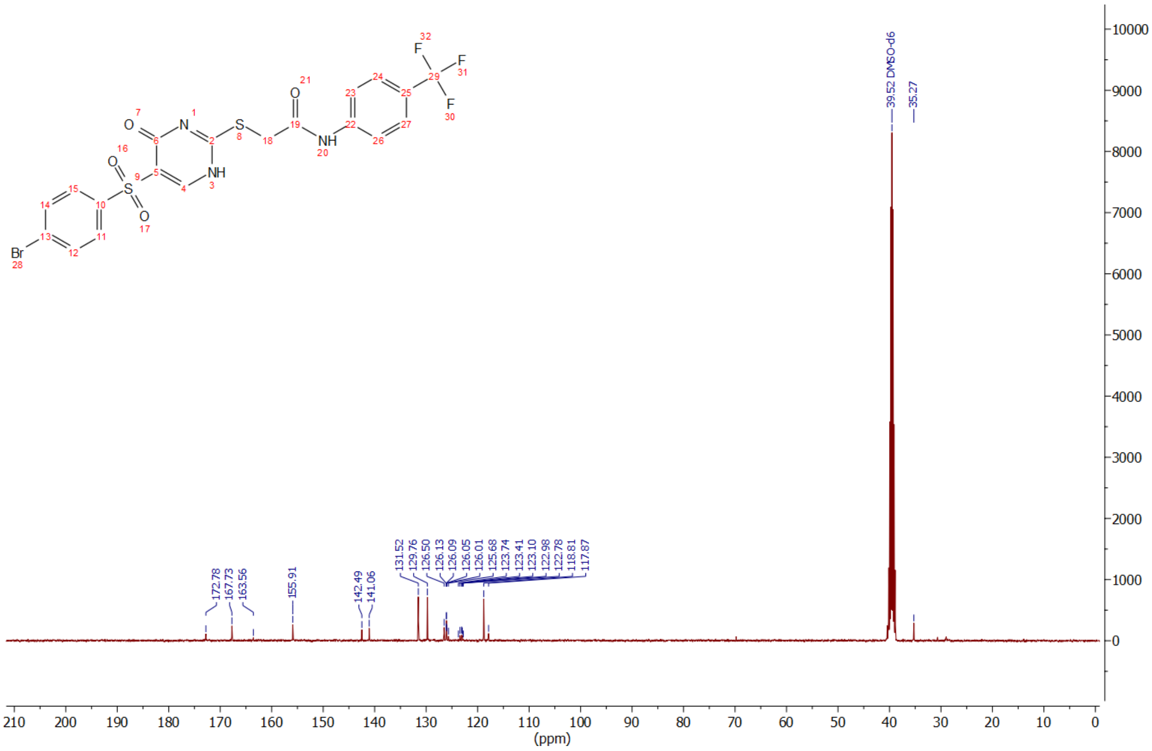

Figure S70. *^{1}H (400 MHz) and $^{13}C\{1H\}$ (101 MHz) NMR spectra in DMSO-d_6 of* ***compound 31***

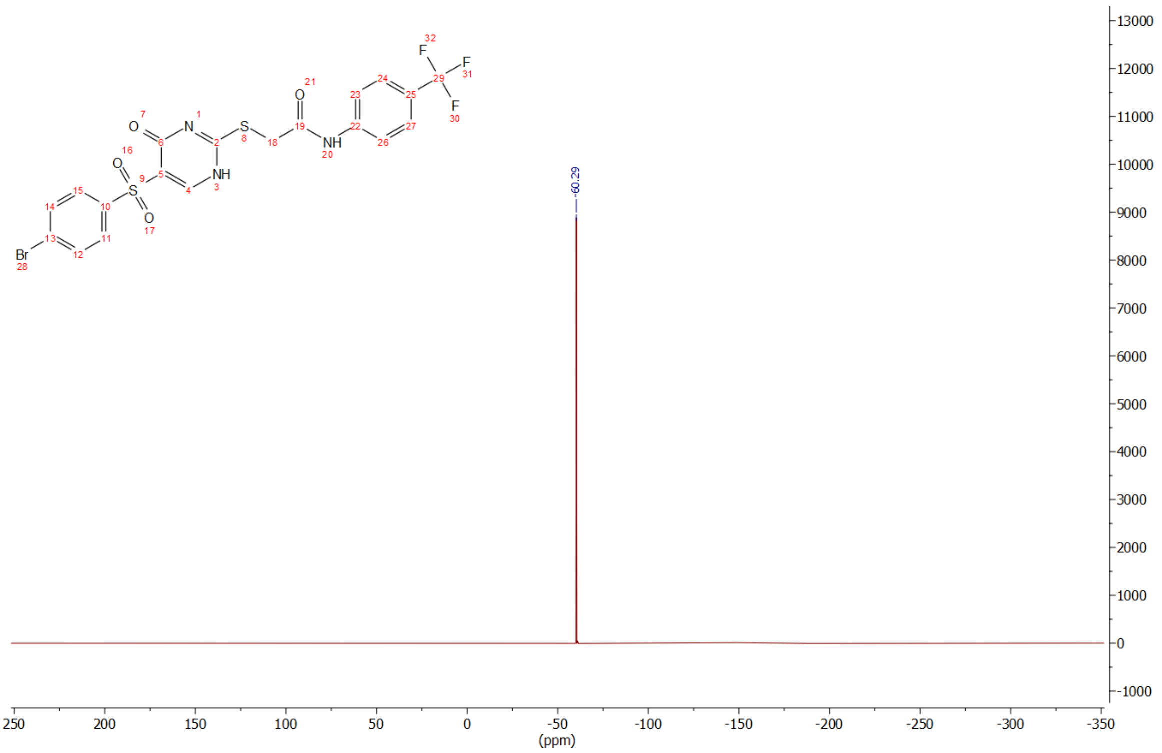

Figure S71. *^{19}F (377 MHz) NMR spectrum in DMSO-d_6 of* ***compound 31***

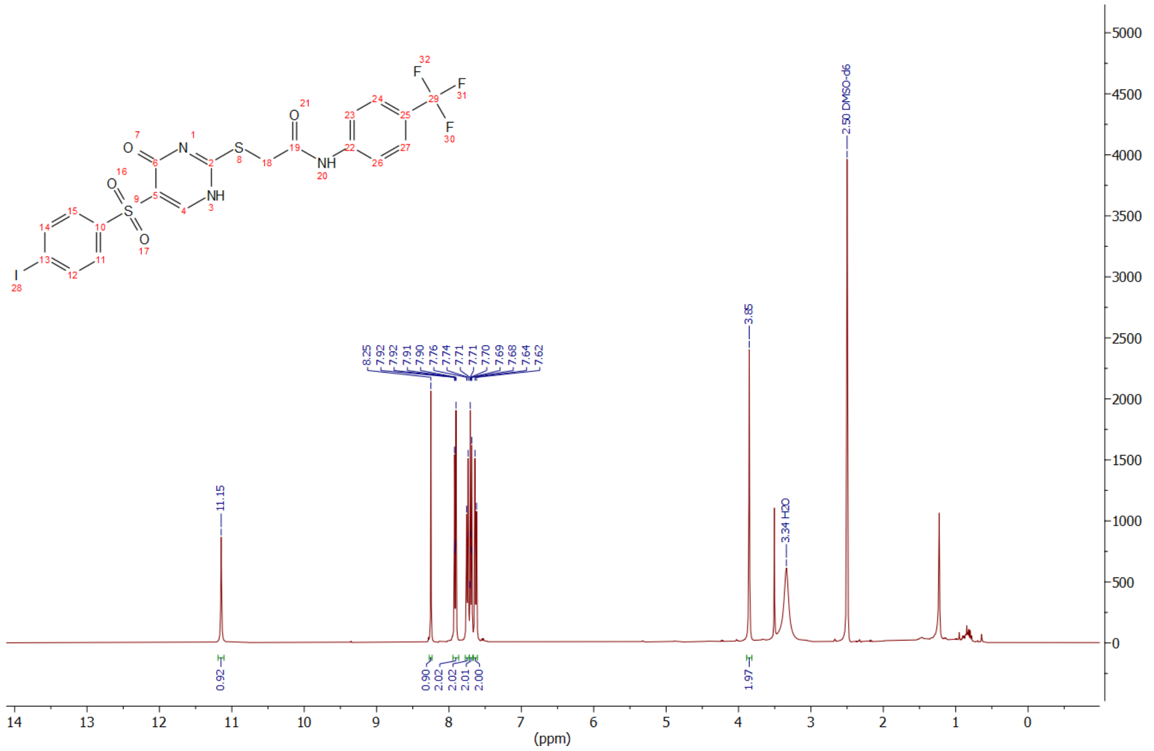

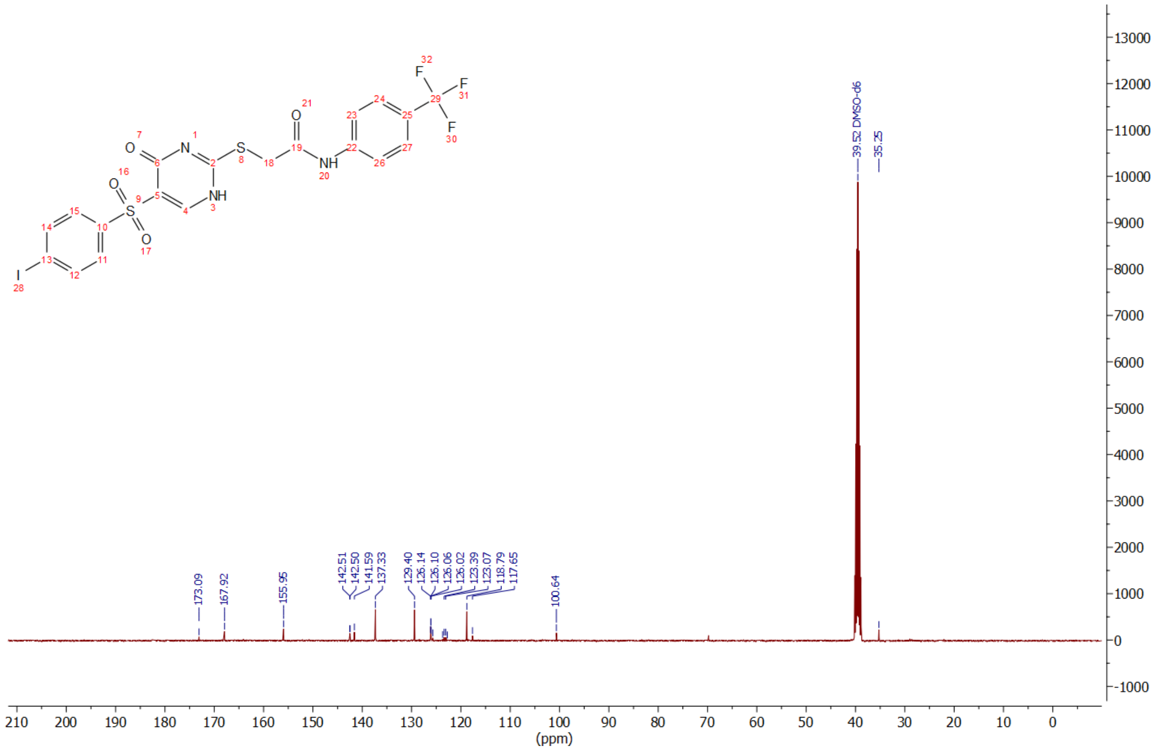

Figure S72. ^{1}H *(400 MHz) and* $^{13}C\{1H\}$ *(101 MHz) NMR spectra in DMSO-*d_6 *of* ***compound 32***

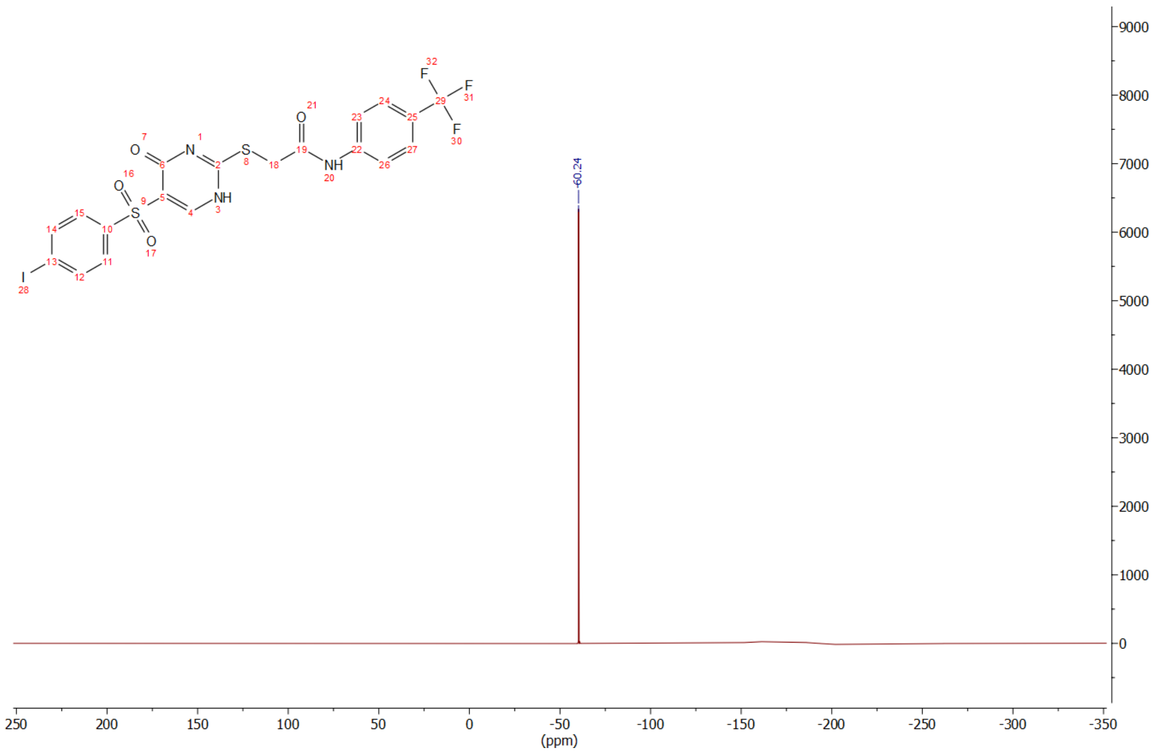

Figure S73. *^{19}F (377 MHz) NMR spectrum in DMSO-d_6 of* ***compound 32***

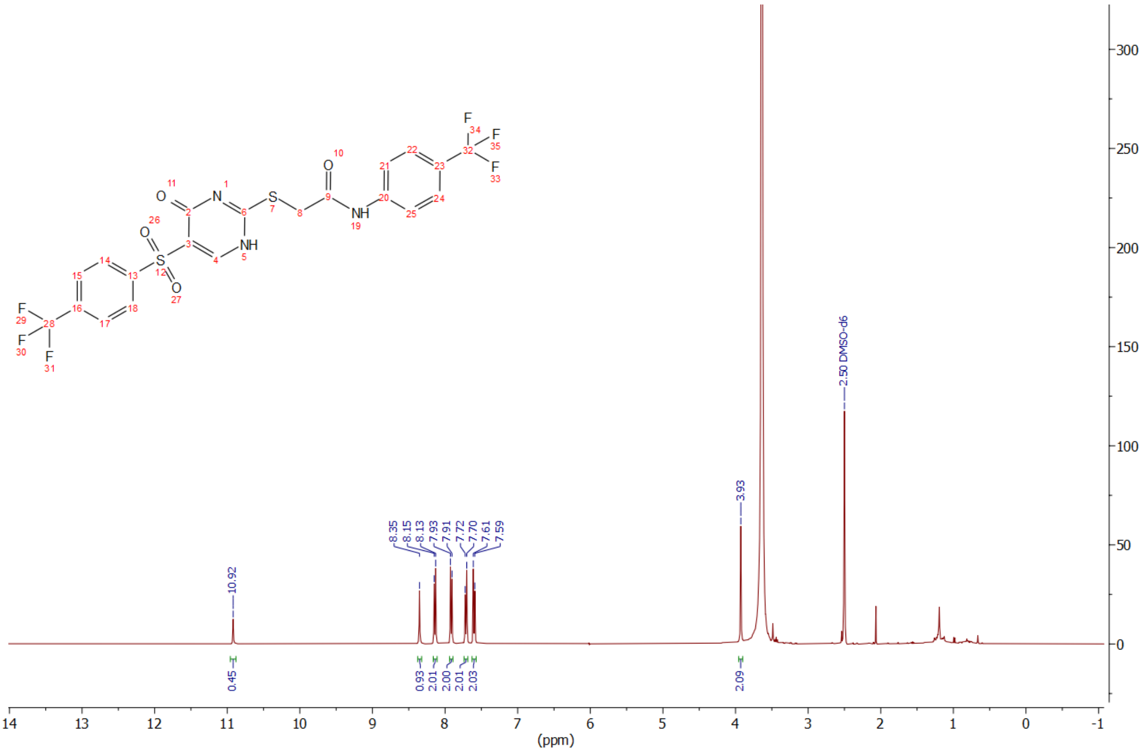

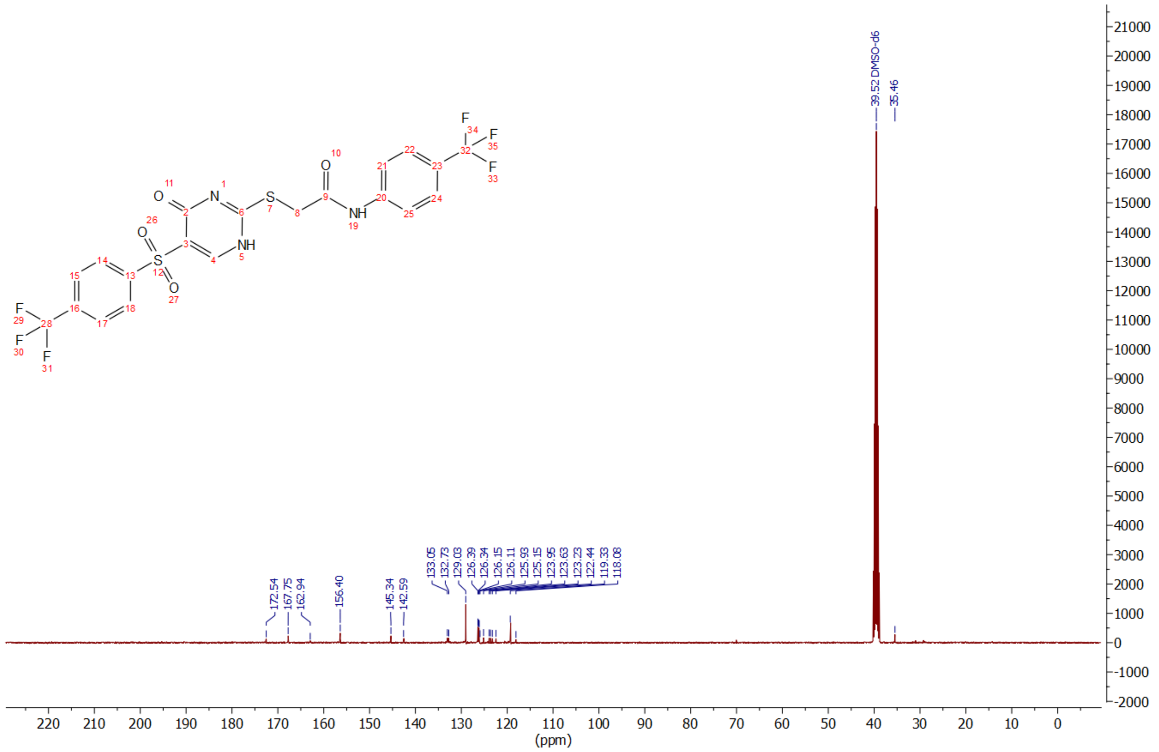

Figure S74. *^{1}H (400 MHz) and $^{13}C\{1H\}$ (101 MHz) NMR spectra in DMSO-d_6 of* ***compound 33***

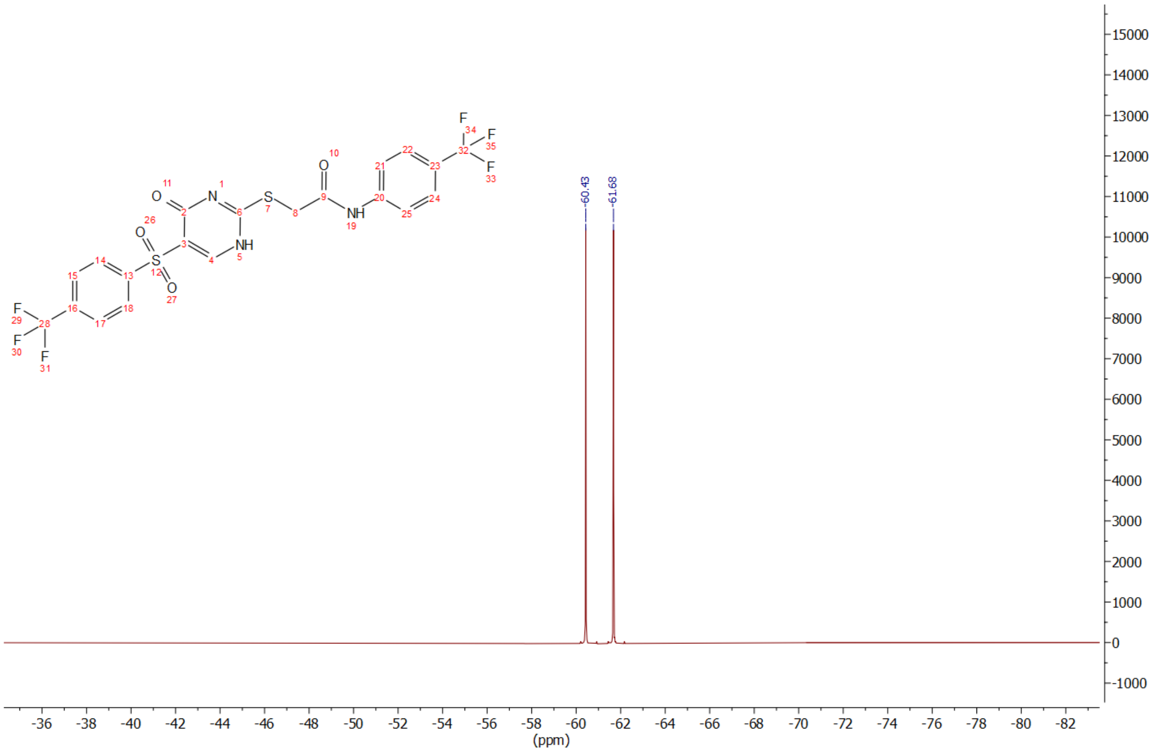

Figure S75. ^{19}*F (376 MHz) NMR spectrum in DMSO-d$_6$ of* ***compound 33***

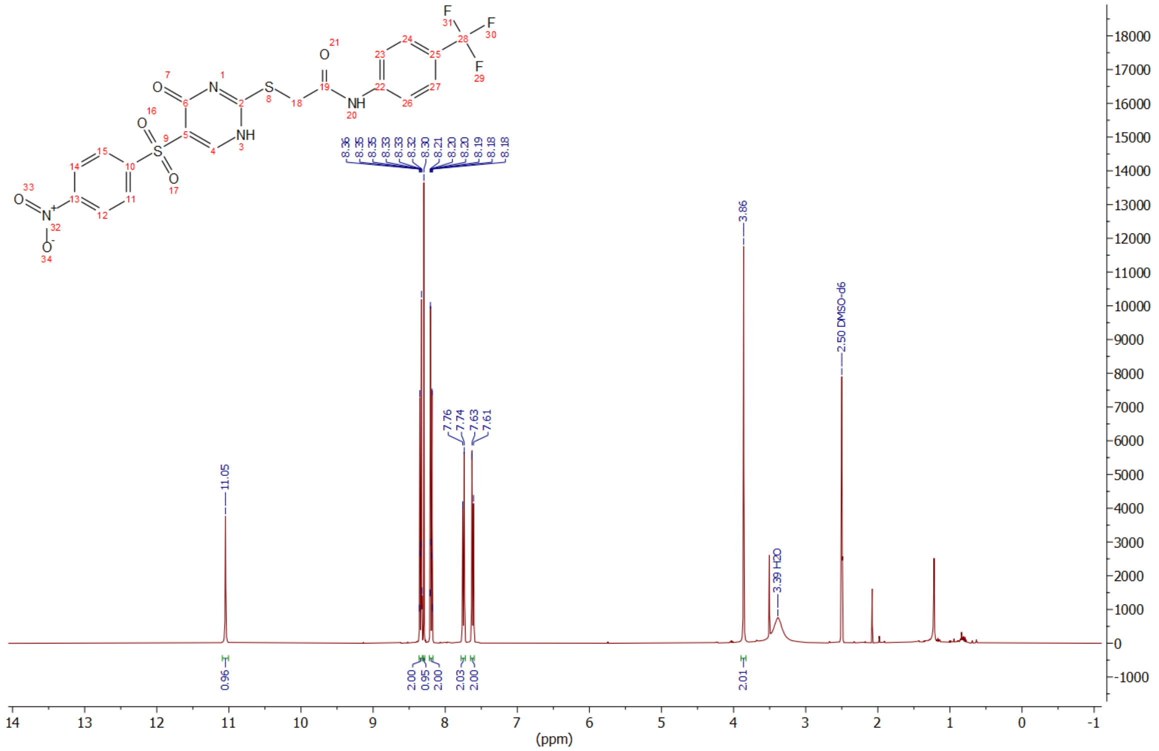

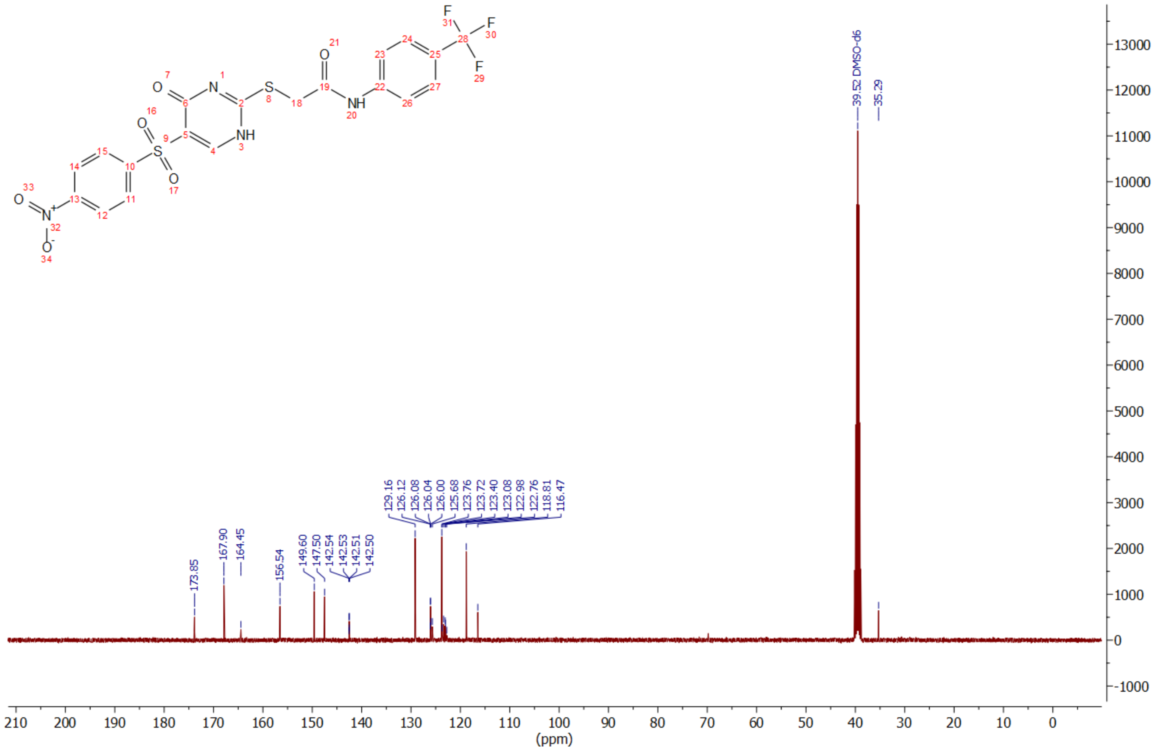

Figure S76. *^{1}H (400 MHz) and ^{13}C{1H} (101 MHz) NMR spectra in DMSO-d_6 of* ***compound 34***

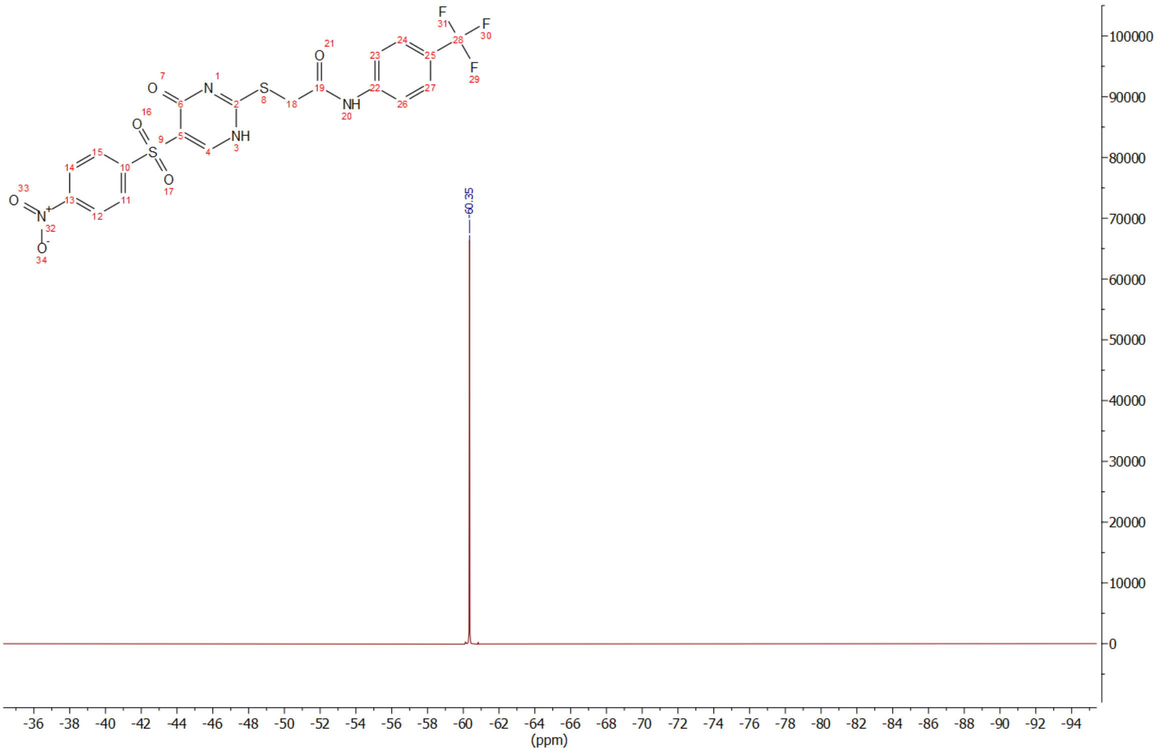

Figure S77. *^{19}F (376 MHz) NMR spectrum in DMSO-d$_6$ of* ***compound 34***

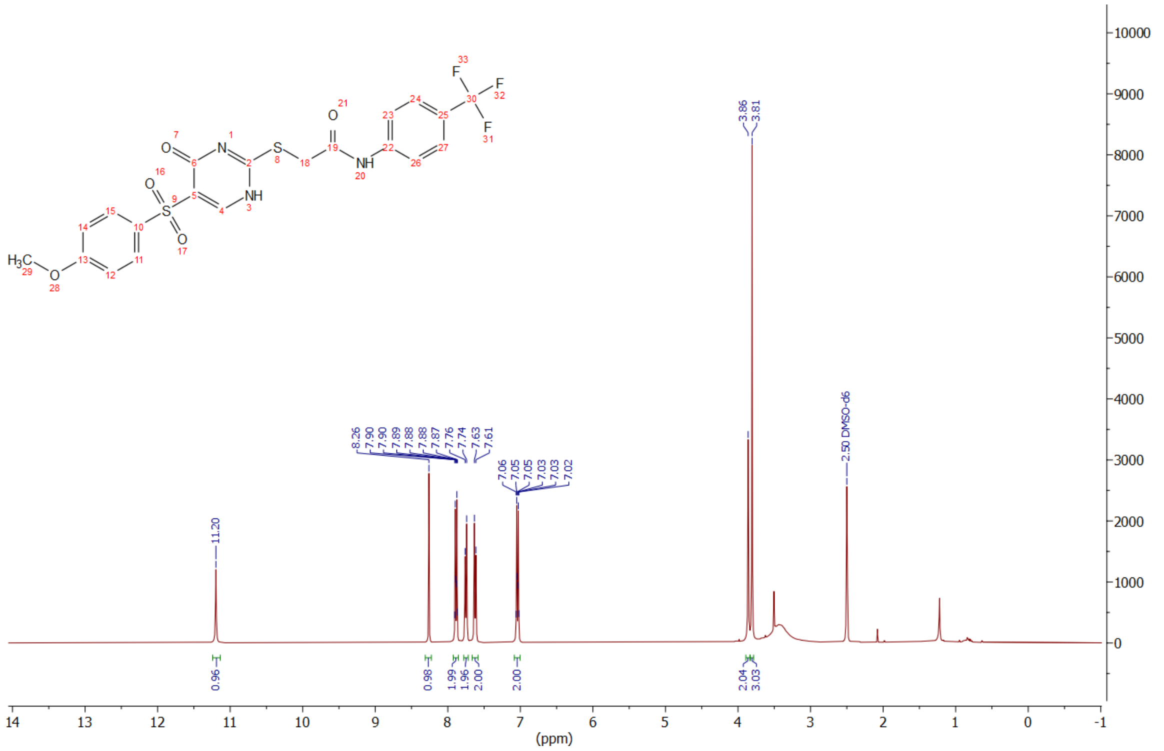

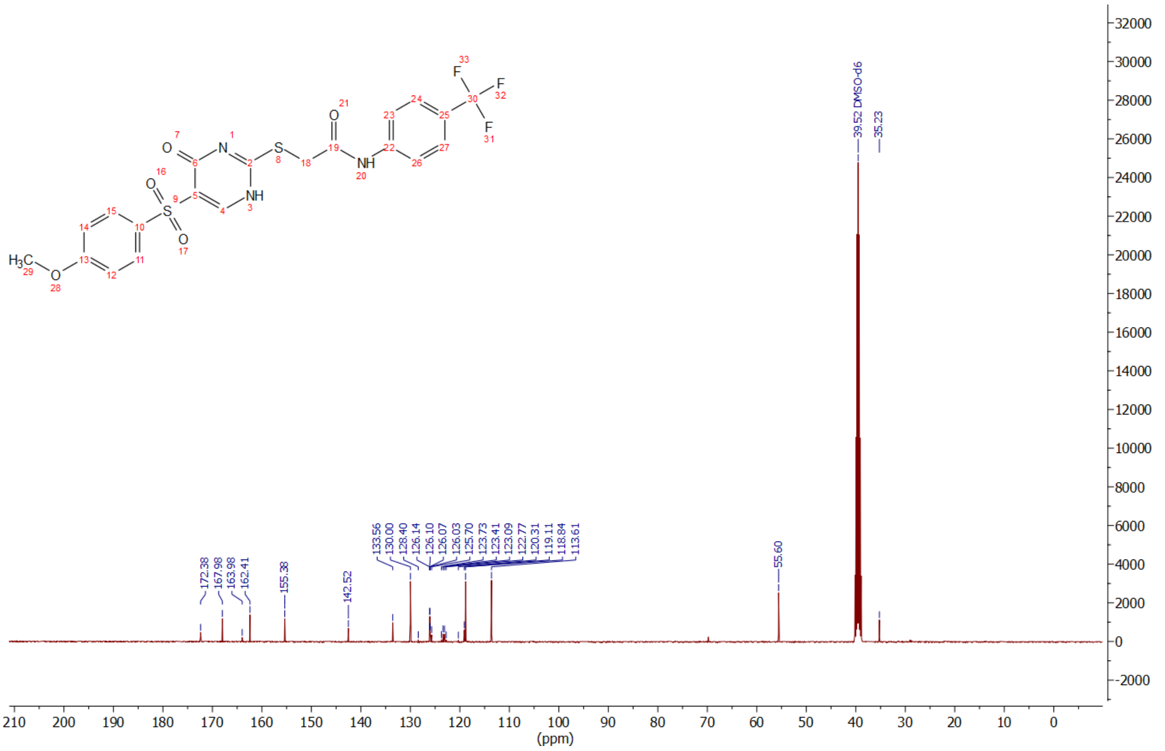

Figure S78. *^{1}H (400 MHz) and ^{13}C{1H} (101 MHz) NMR spectra in DMSO-d_6 of* ***compound 35***

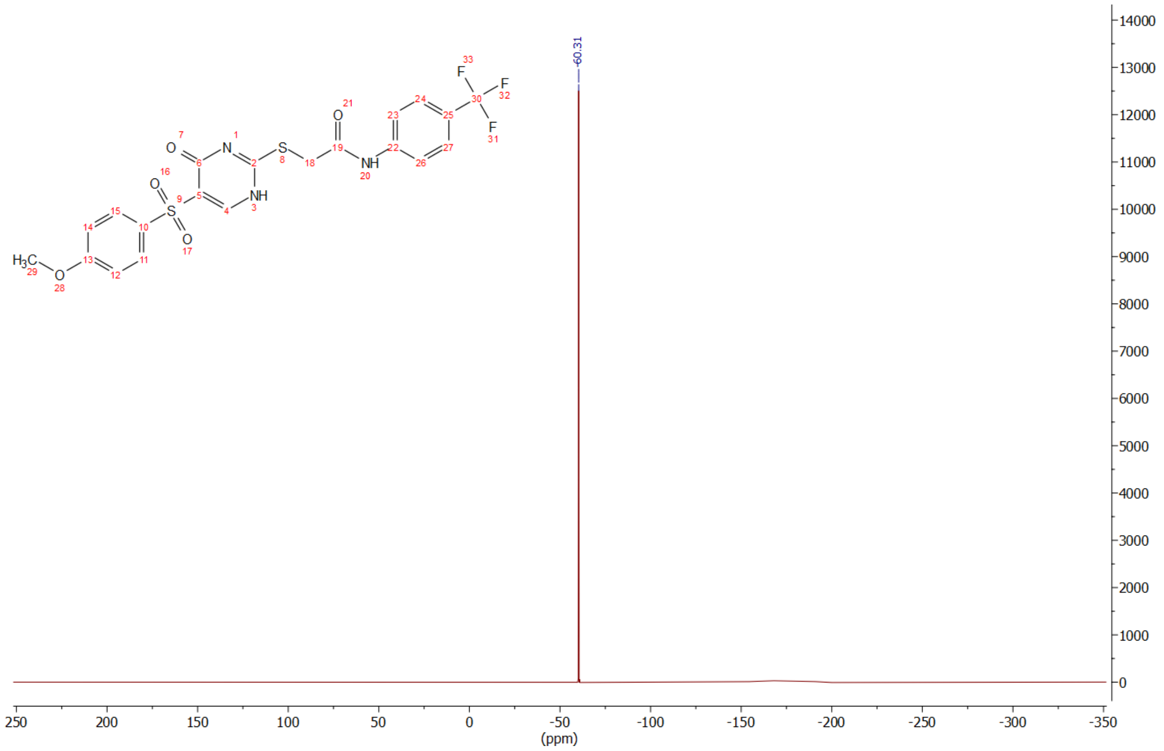

Figure S79. *^{19}F (377 MHz) NMR spectrum in DMSO-d_6 of* ***compound 25***

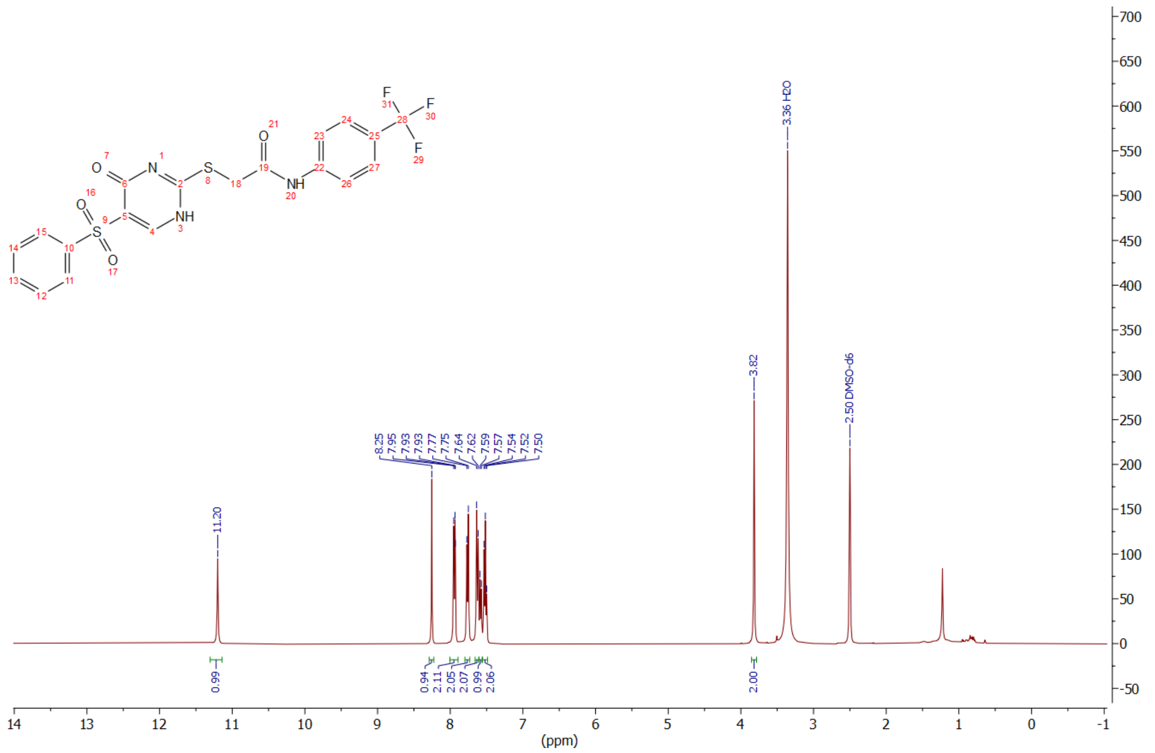

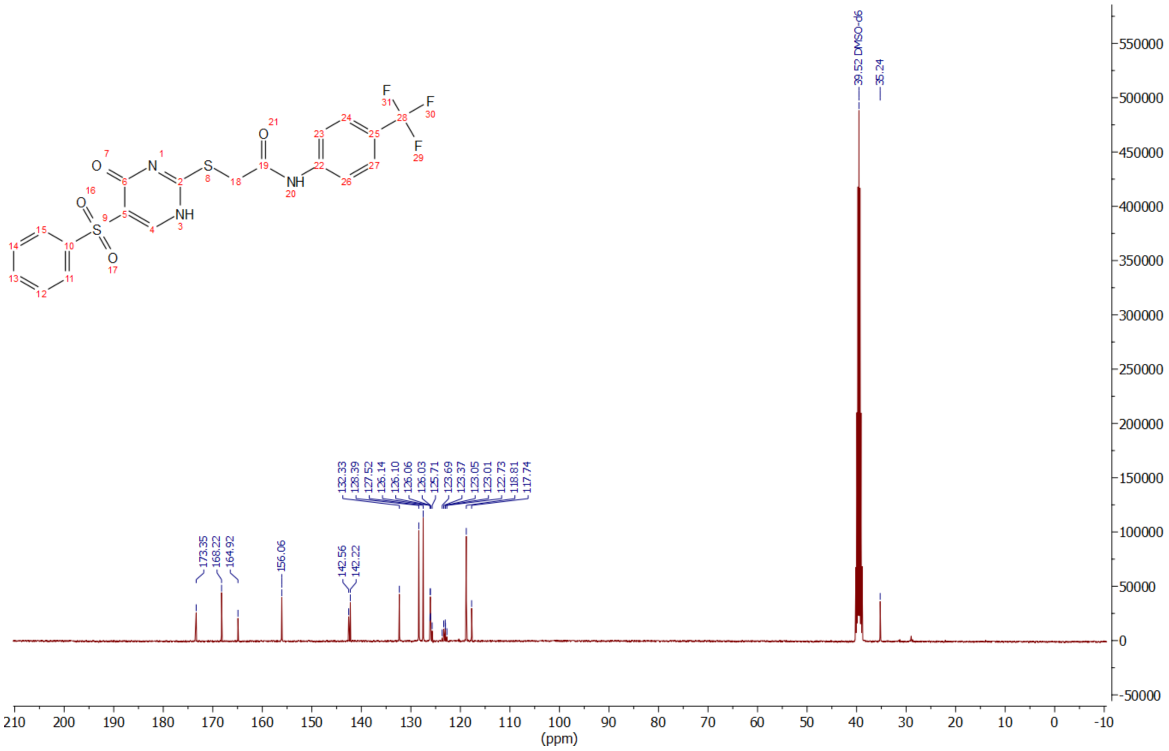

Figure S80. *^{1}H (400 MHz) and $^{13}C\{1H\}$ (101 MHz) NMR spectra in DMSO-d_6 of* ***compound 36***

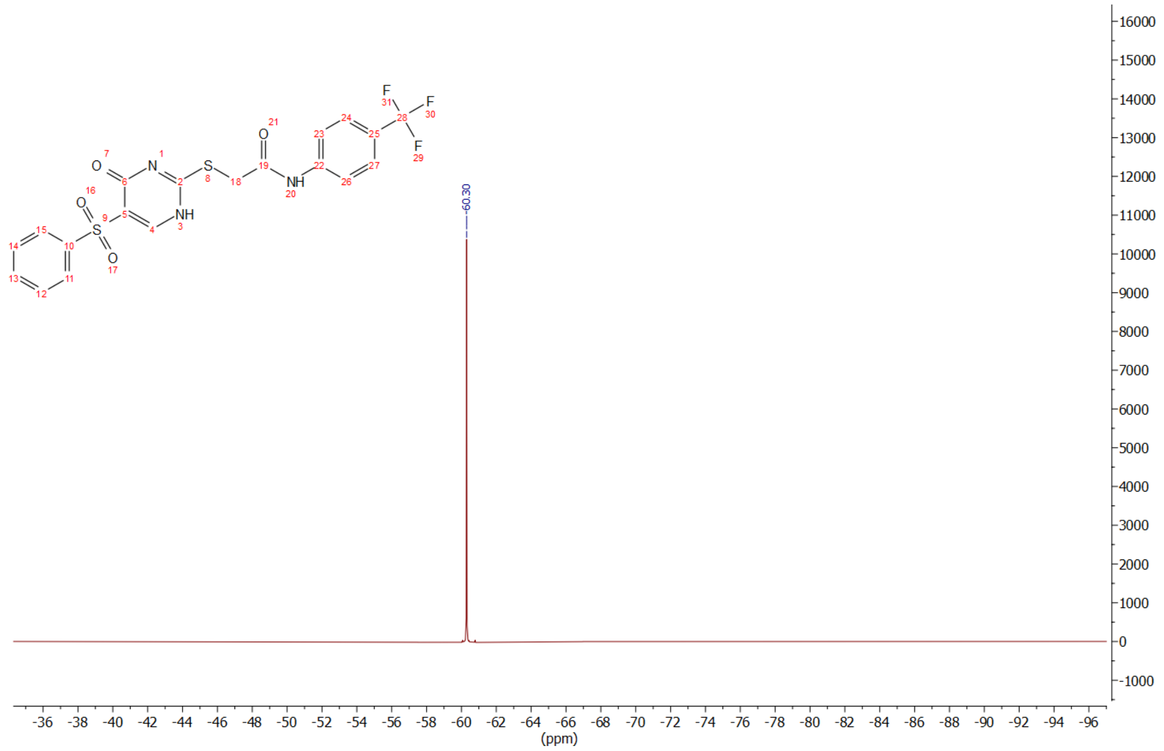

Figure S81. *^{19}F (376 MHz) NMR spectrum in DMSO-d_6 of* ***compound 36***

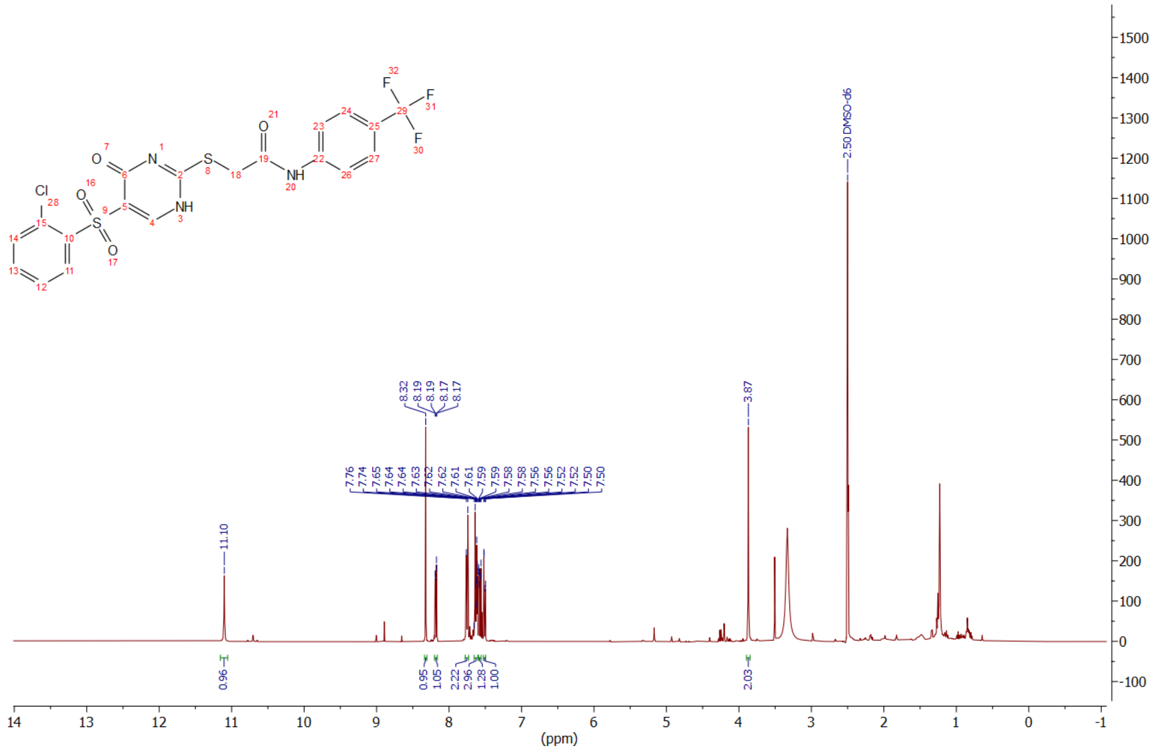

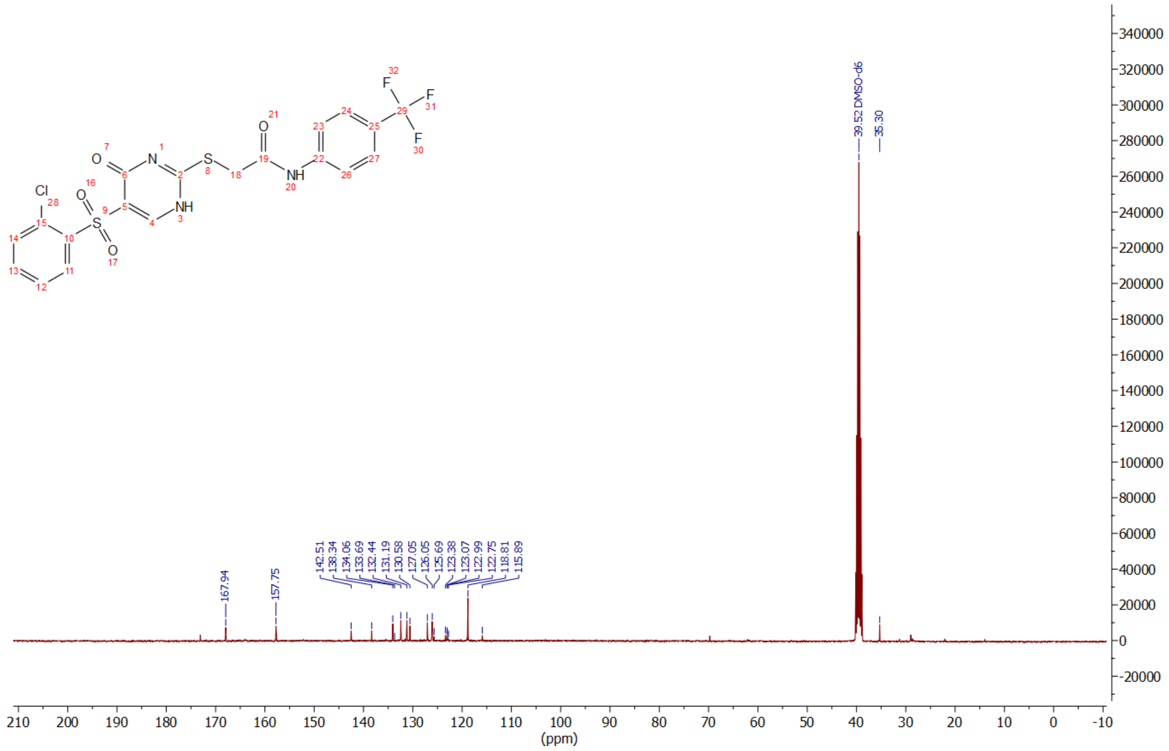

Figure S82. *^{1}H (400 MHz) and ^{13}C{1H} (101 MHz) NMR spectra in DMSO-d$_6$ of* ***compound 37***

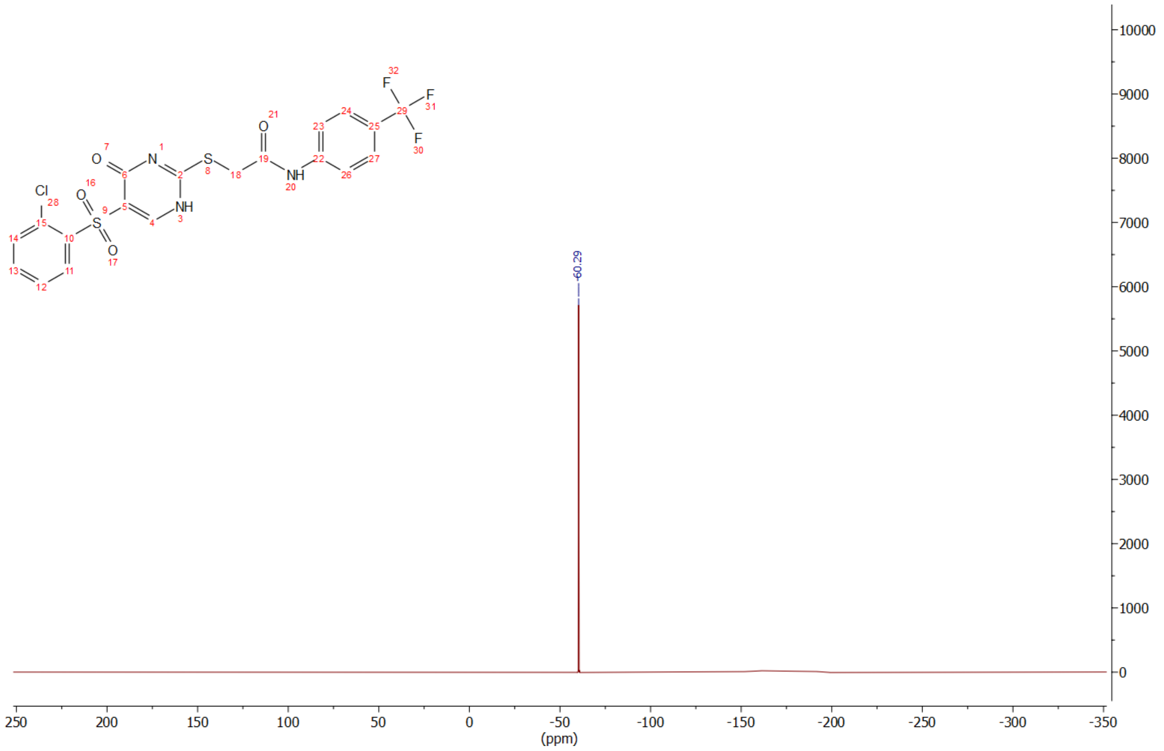

Figure S83. *^{19}F (377 MHz) NMR spectrum in DMSO-d$_6$ of* ***compound 37***

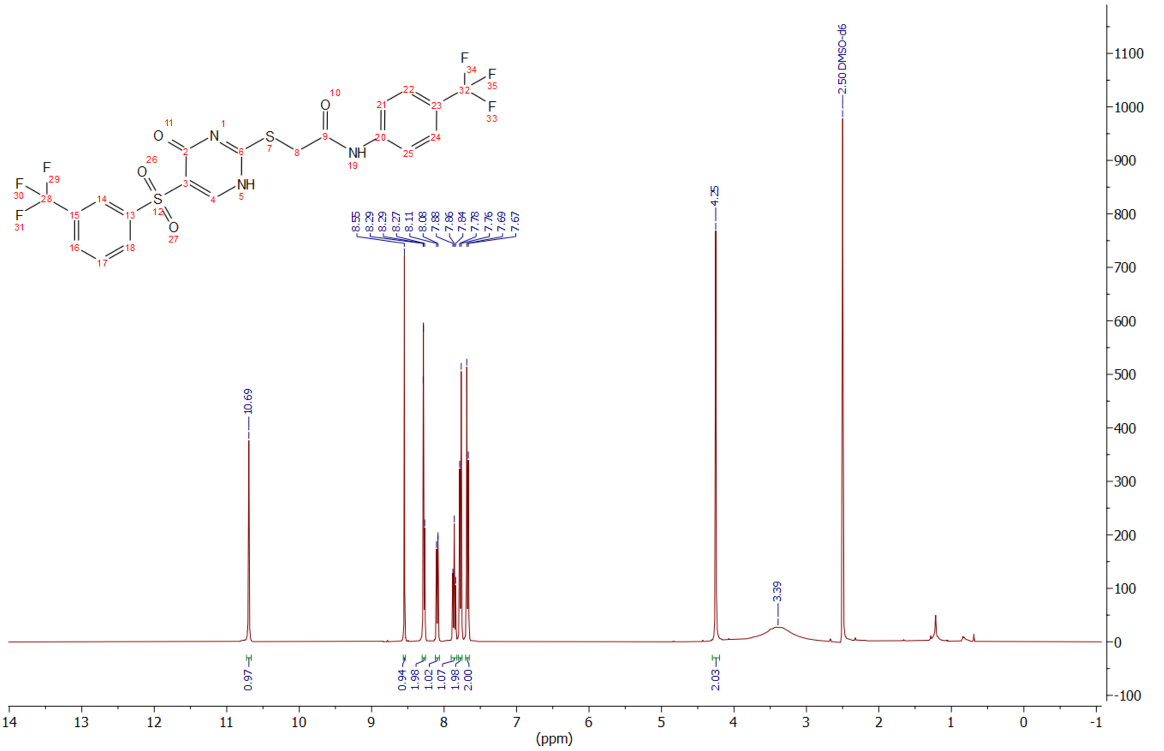

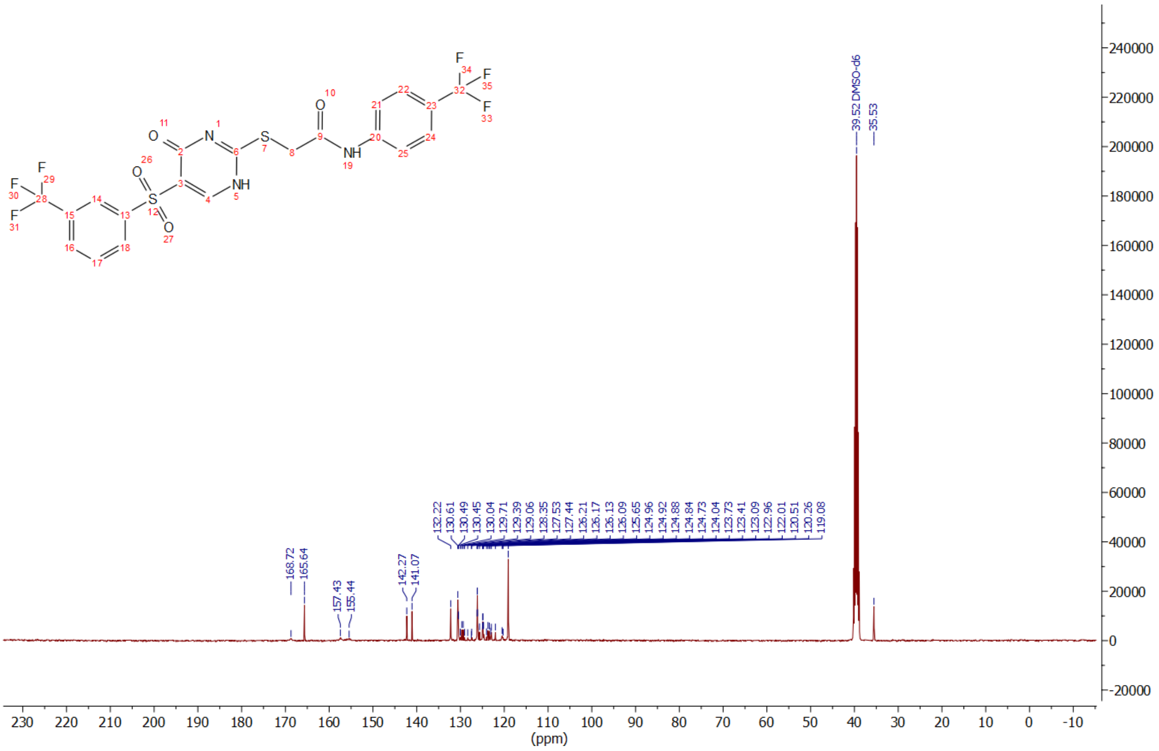

Figure S84. *^{1}H (400 MHz) and $^{13}C\{1H\}$ (101 MHz) NMR spectra in DMSO-d_6 of* ***compound 38***

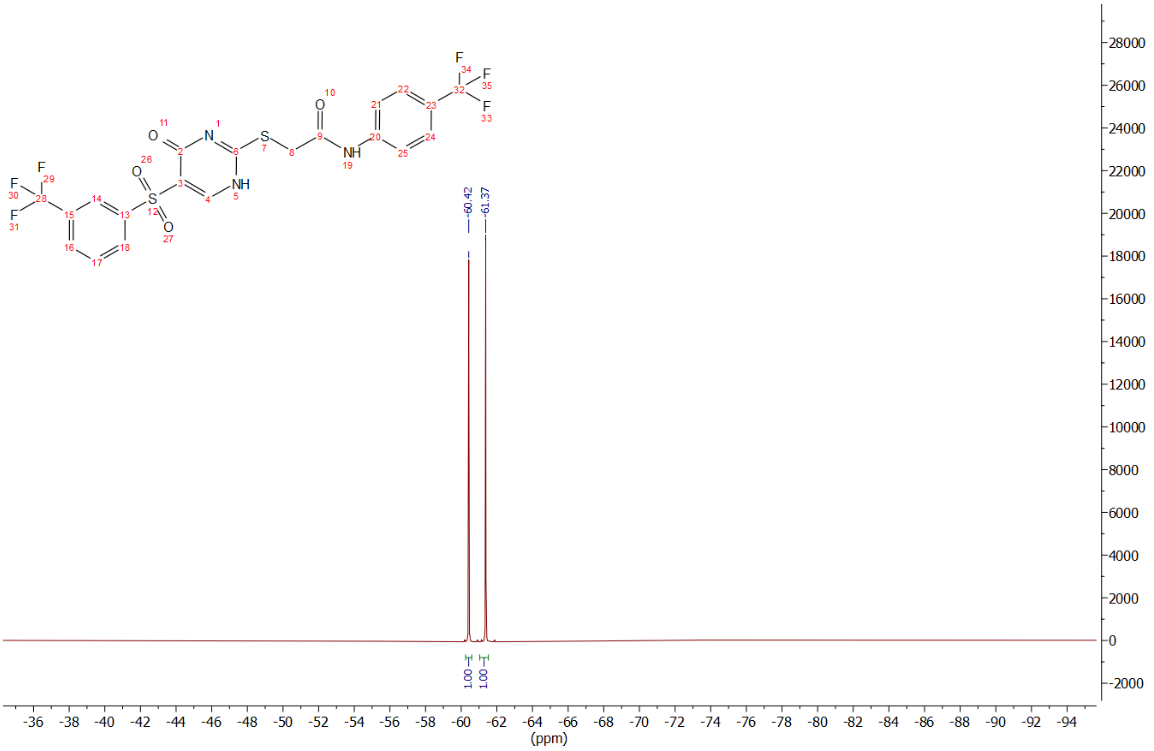

Figure S85. *^{19}F (376 MHz) NMR spectrum in DMSO-d$_6$ of* ***compound 38***

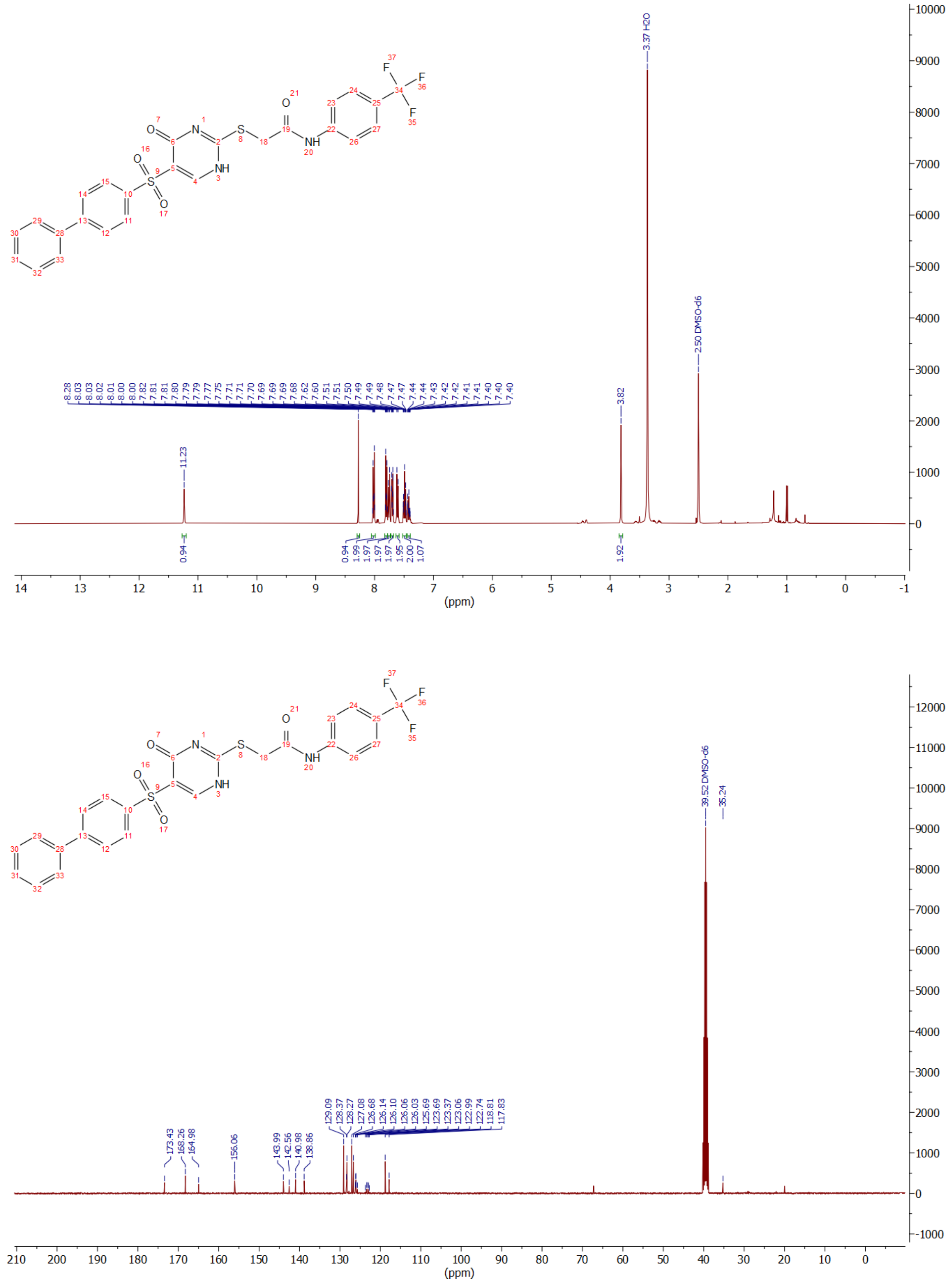

Figure S86. *^{1}H (400 MHz) and ^{13}C{1H} (101 MHz) NMR spectra in DMSO-d$_6$ of* ***compound 39***

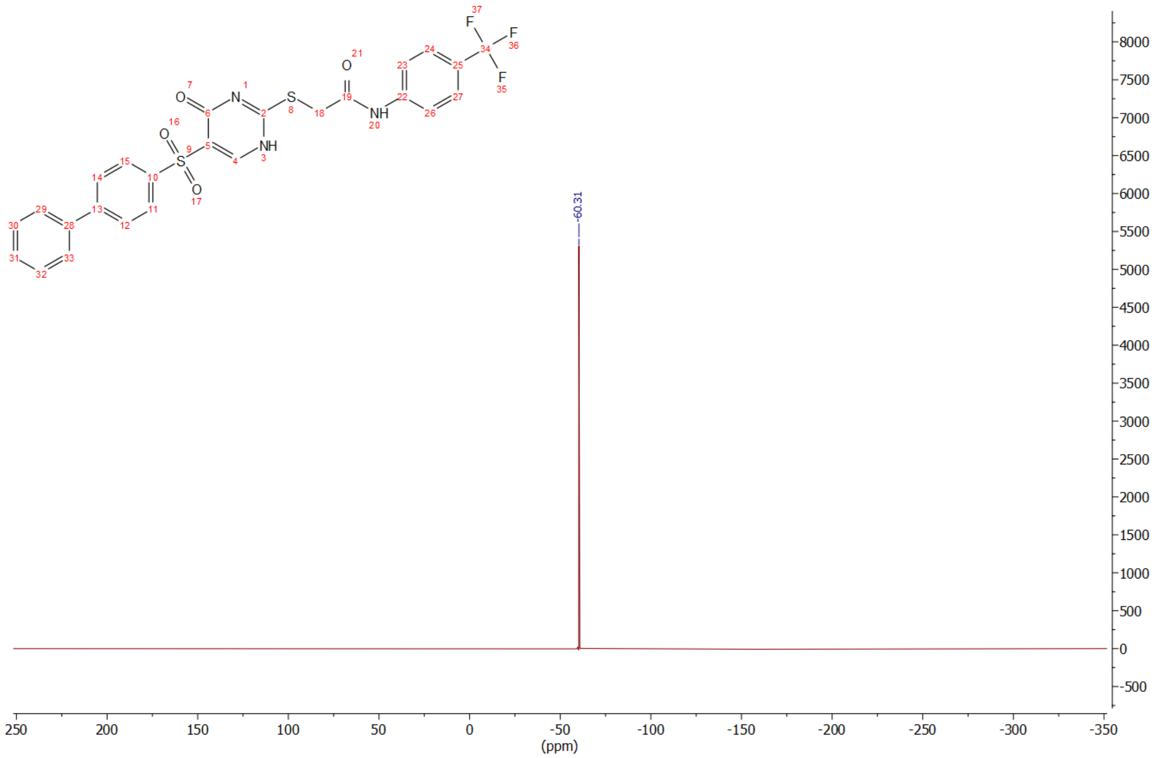

Figure S87. *^{19}F (377 MHz) NMR spectrum in DMSO-d_6 of* ***compound 39***

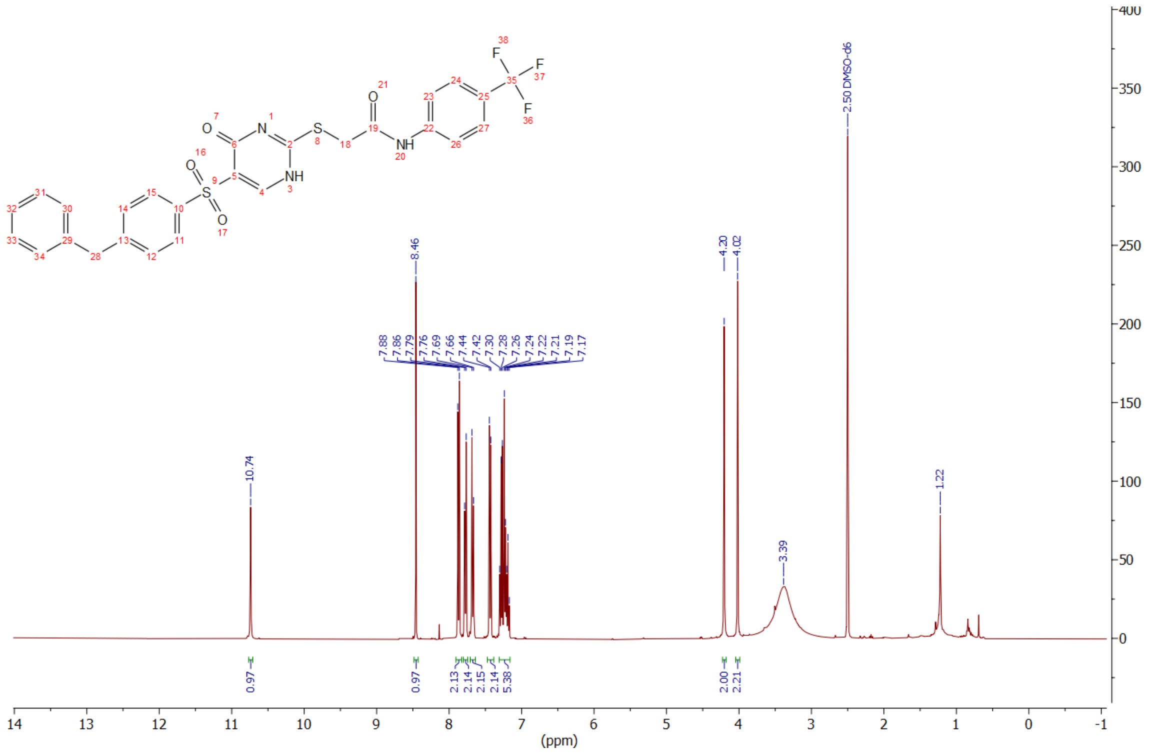

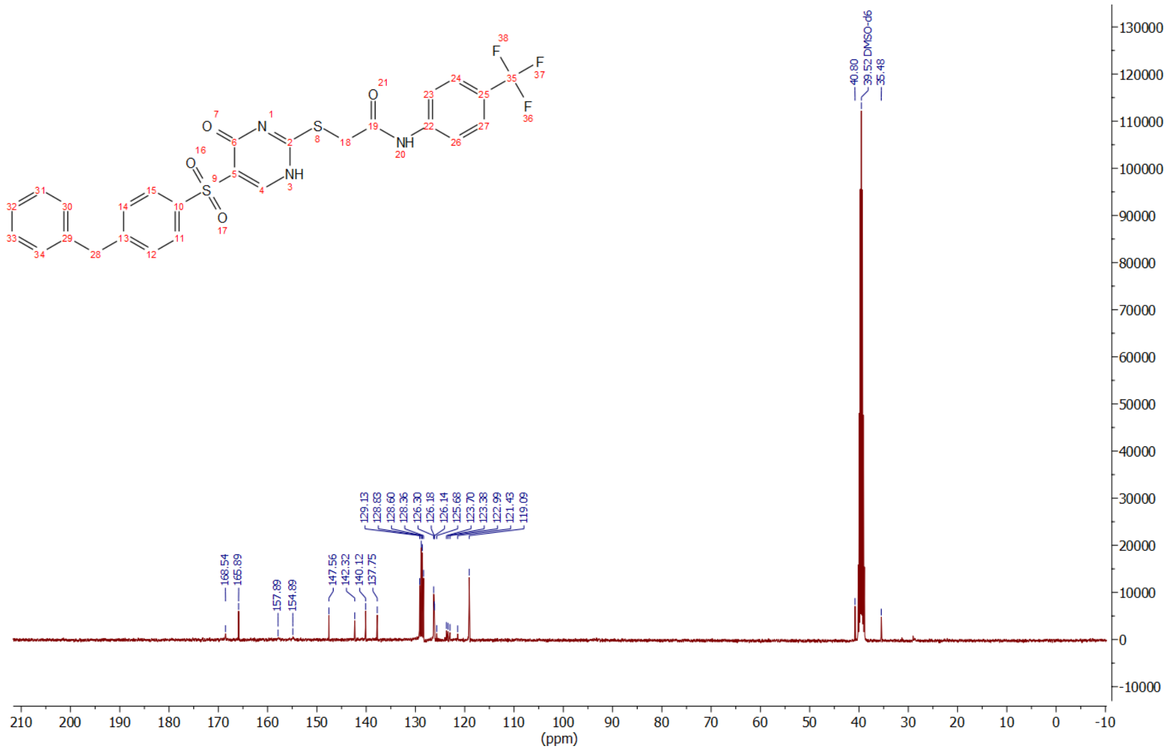

Figure S88. *^{1}H (400 MHz) and $^{13}C\{1H\}$ (101 MHz) NMR spectra in DMSO-d_6 of* ***compound 40***

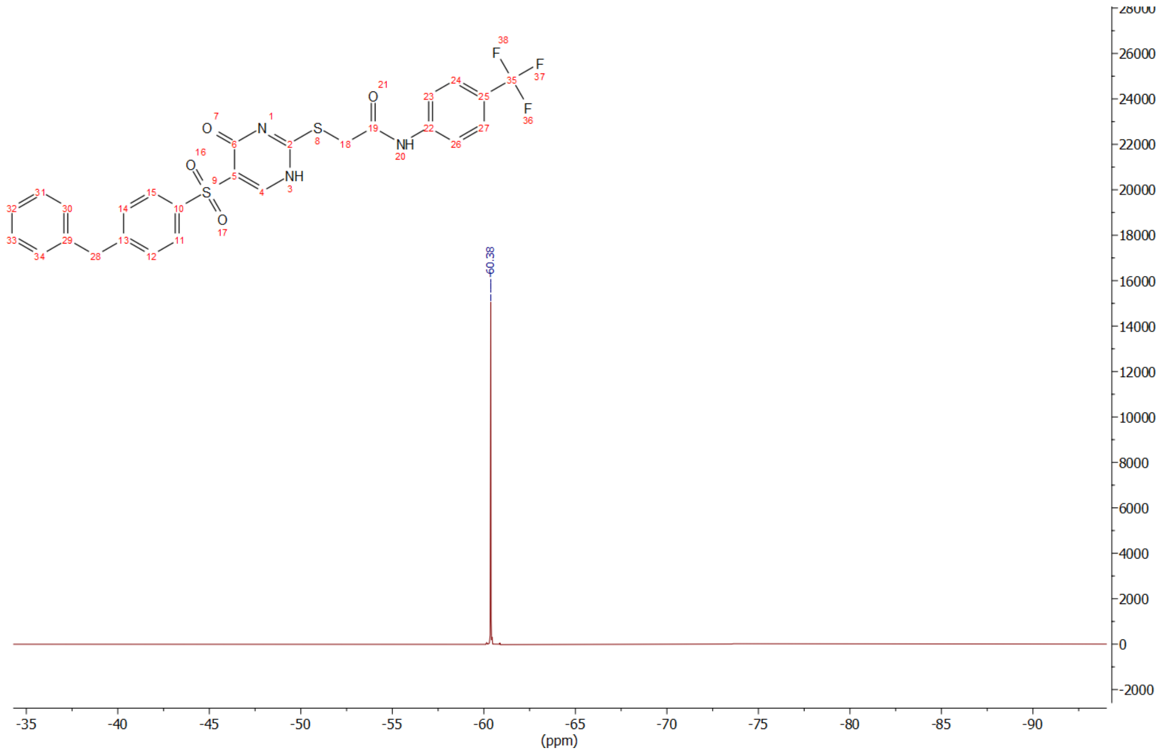

Figure S89. *^{19}F (376 MHz) NMR spectrum in DMSO-d$_6$ of* ***compound 40***

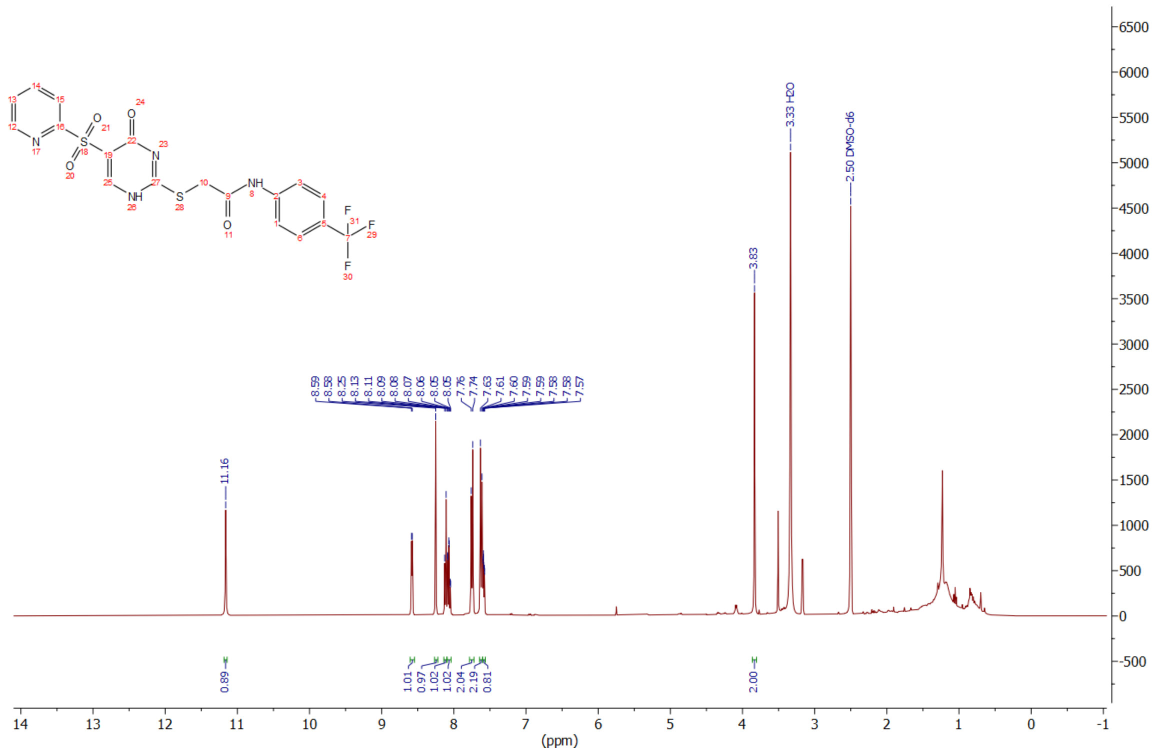

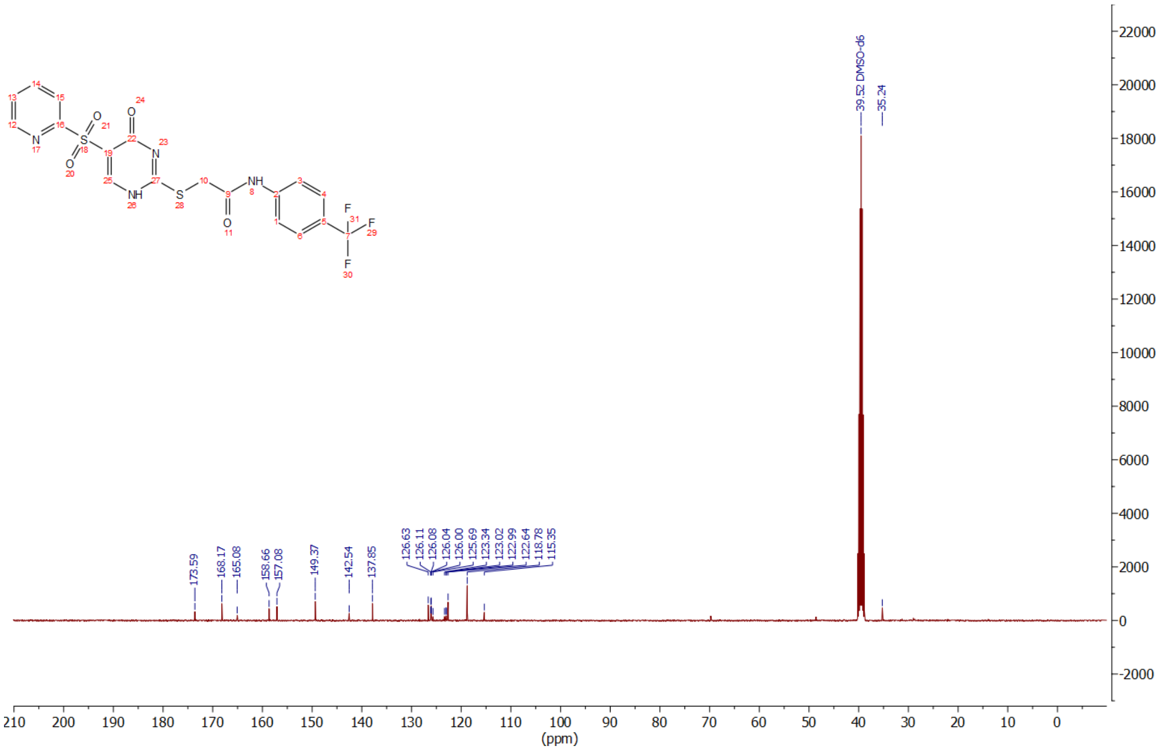

Figure S90. *^{1}H (400 MHz) and $^{13}C\{1H\}$ (101 MHz) NMR spectra in DMSO-d_6 of* ***compound 41***

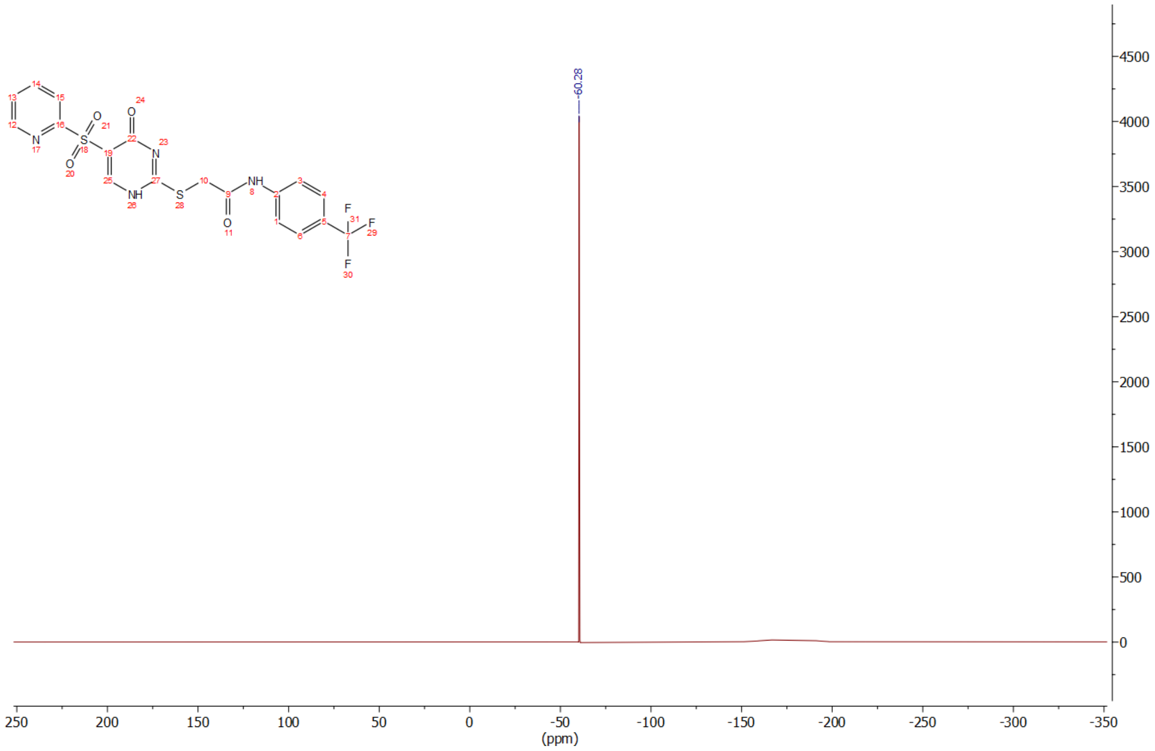

Figure S91. *^{19}F (377 MHz) NMR spectrum in DMSO-d_6 of* ***compound 41***

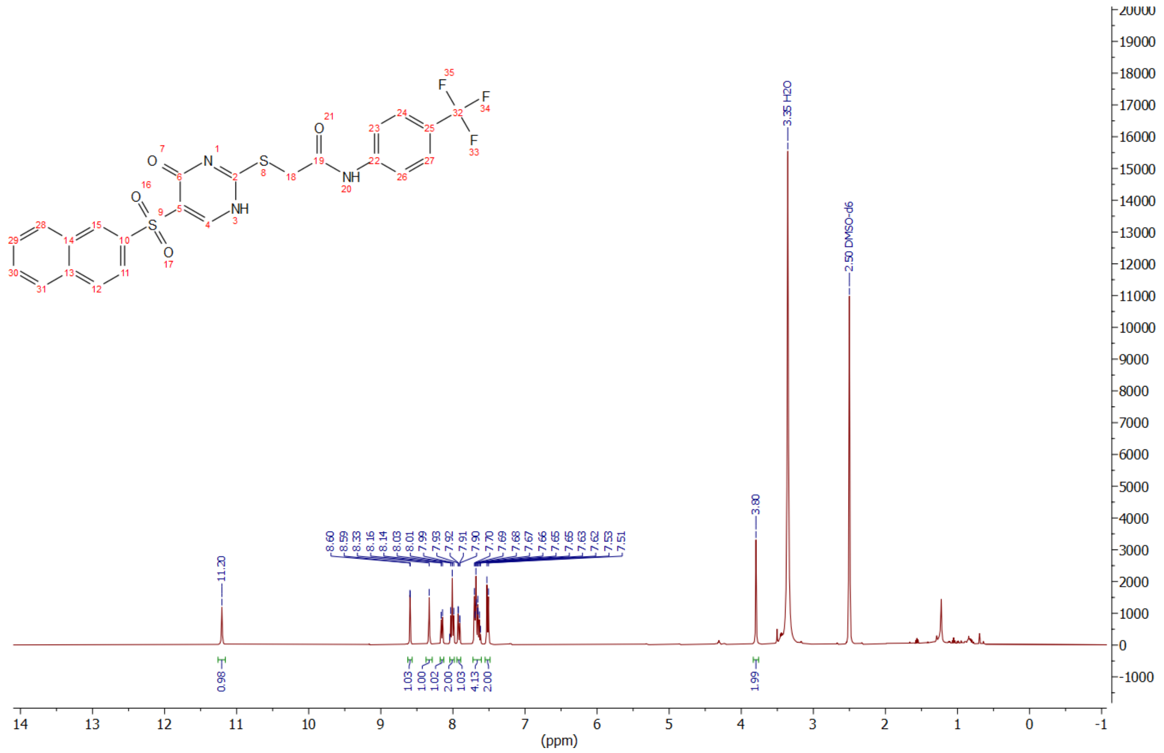

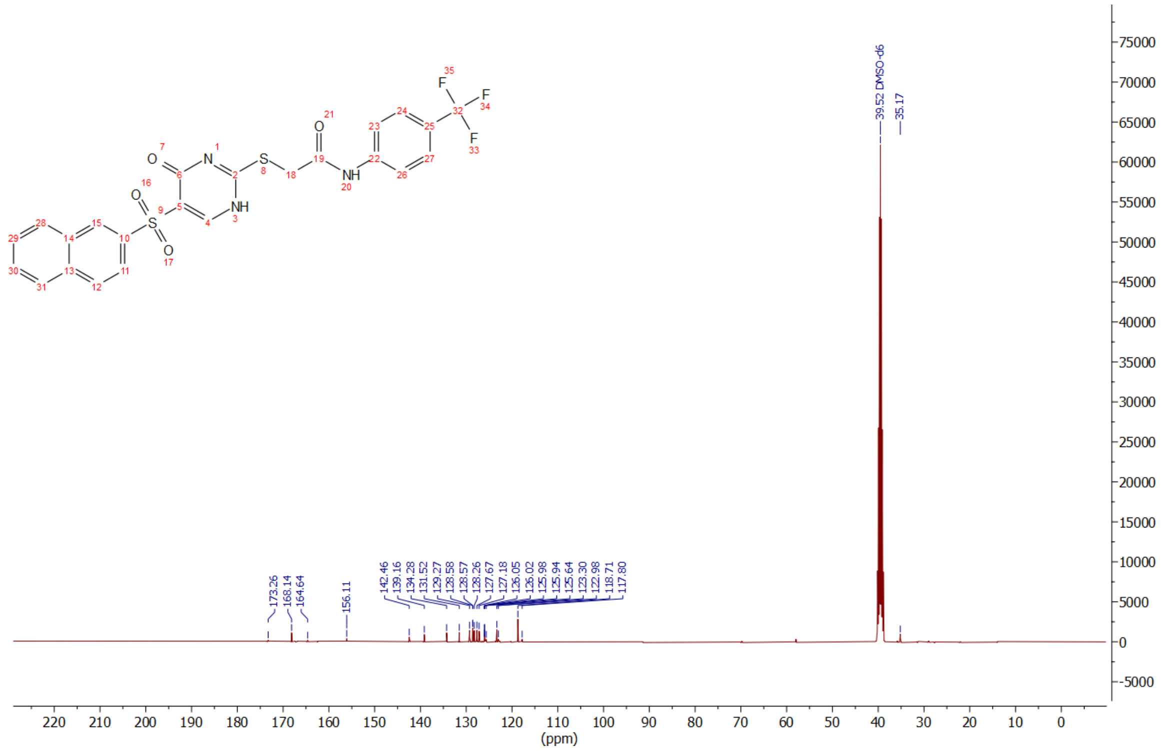

Figure S92. *^{1}H (400 MHz) and ^{13}C{1H} (101 MHz) NMR spectra in DMSO-d_6 of* ***compound 42***

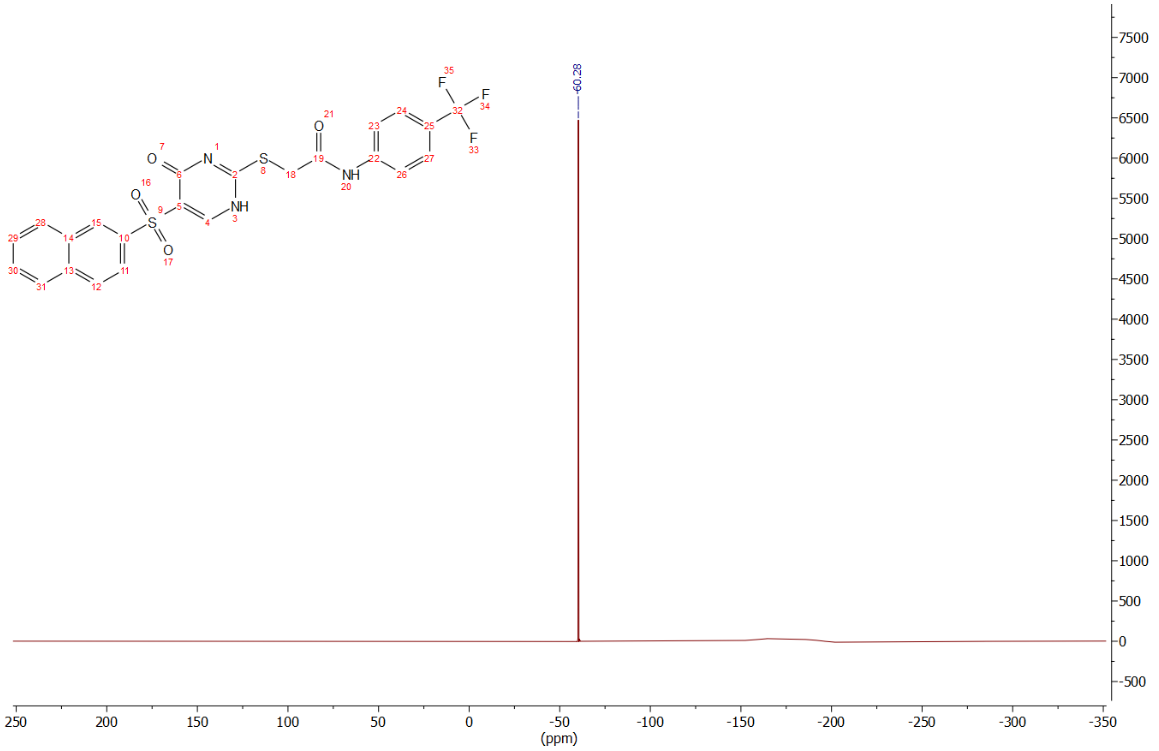

Figure S93. *^{19}F (377 MHz) NMR spectrum in DMSO-d$_6$ of* ***compound 42***

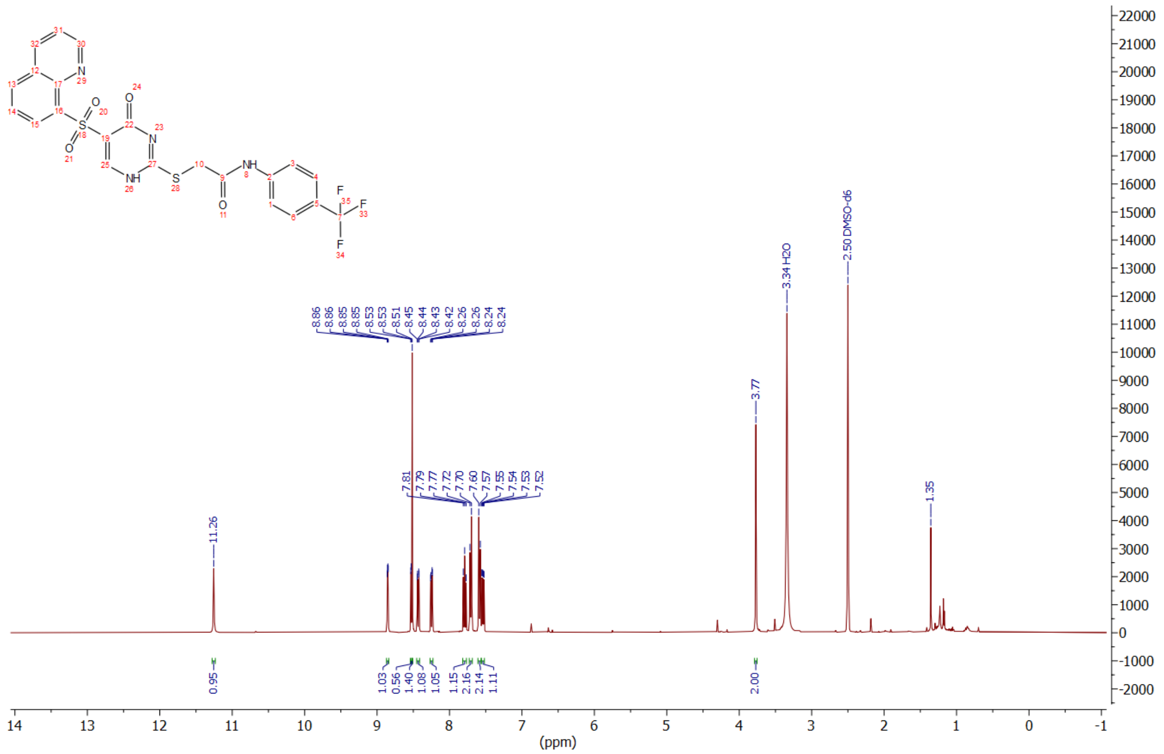

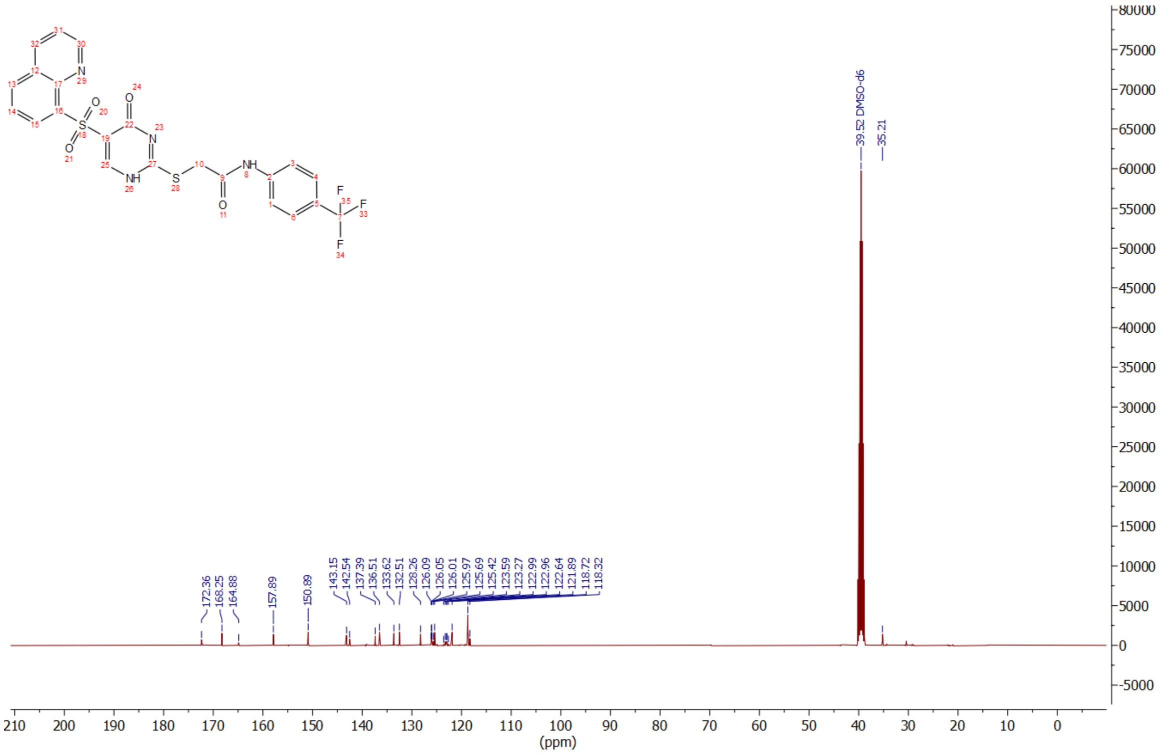

Figure S94. ^{1}H (400 MHz) and ^{13}C{1H} (101 MHz) NMR spectra in DMSO-d_6 of ***compound 43***

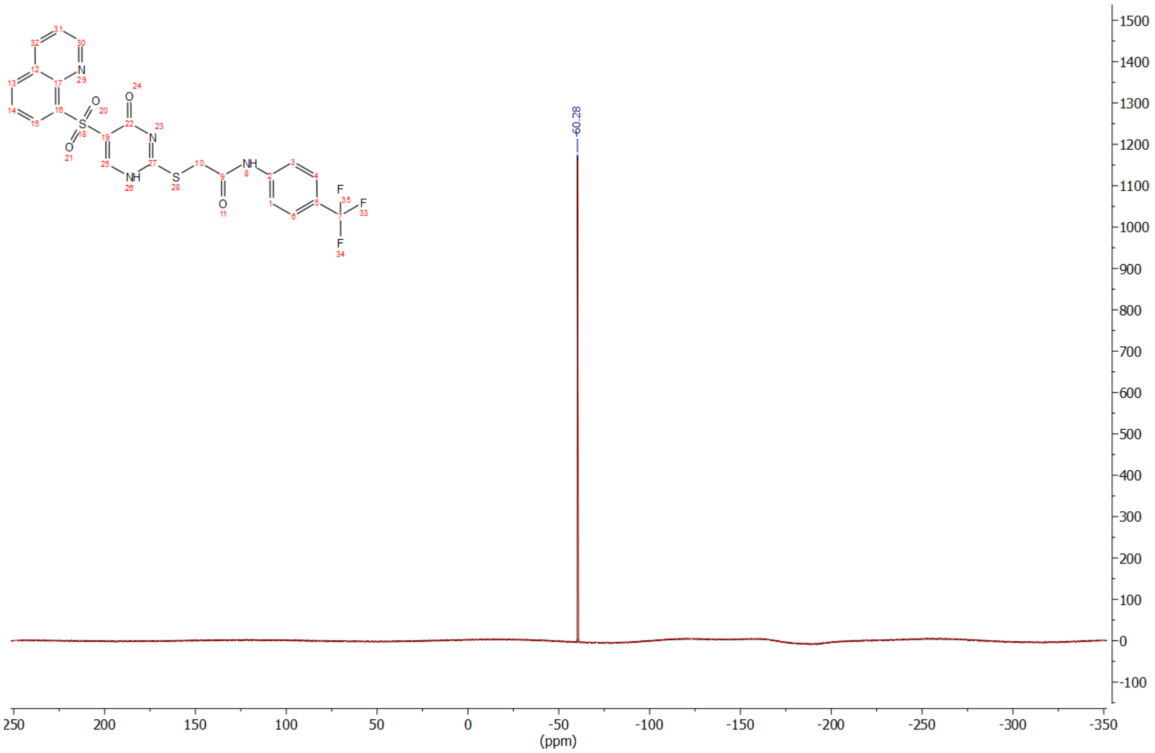

Figure S95. *^{19}F (377 MHz) NMR spectrum in DMSO-d_6 of* ***compound 43***

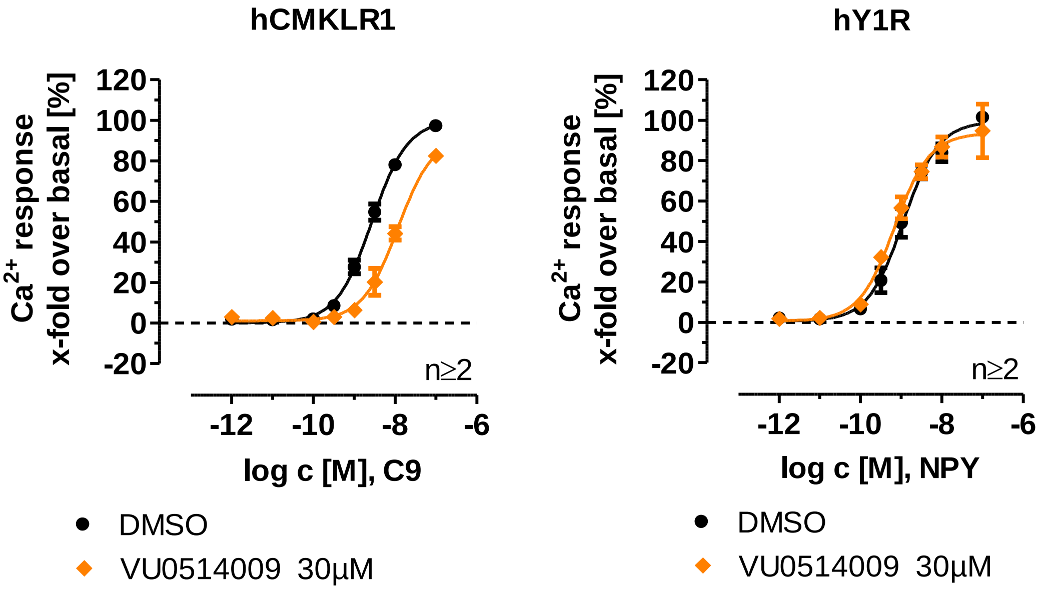

Figure S96: Selectivity of VU0514009 at two different class A GPCRs.

COS7 cells stably expressing hCMKLR1-eYFP or hY1R-eYFP and a chimeric G protein $G\alpha_{\Delta 6qi4myr}$ were stimulated with different concentrations of chemerin-9 (C9) and constant 30 µM of VU0514009, resulting in double concentration-response curves (dCRC). Control (ctl) contains 0.3% of DMSO instead of the compound. All measurements were performed in duplicates, at least two times and represented the mean±SEM.

A

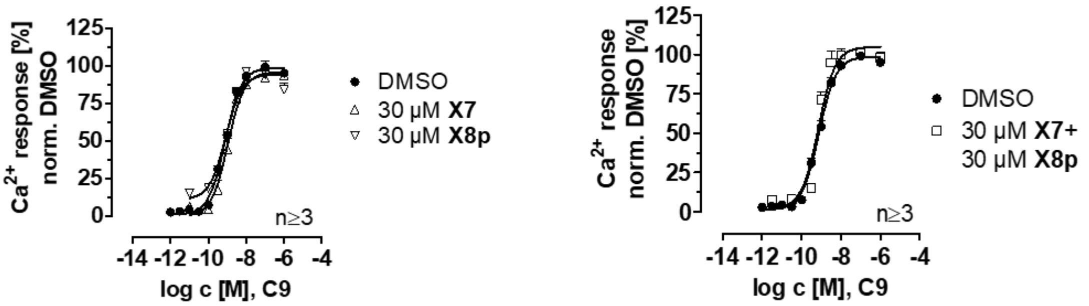

B

name	MW [g/mol]	Ca²⁺ (G protein)							
		EC$_{50}$ [nM]	Emax	±	SEM	pEC$_{50}$	±	SEM	EC$_{50}$ fold over ctl
DMSO (ctl)	78.13	0.8	99	±	1.3	9.1	±	0.04	-
**X7**	337.76	1.1	96	±	2.9	9.0	±	0.08	1.4
**X8p**	282.06	0.8	94	±	2.8	9.1	±	0.10	1.0
**X7 + X8p**	mix	0.7	105	±	3.8	9.1	±	0.10	0.9

Figure S97: Behavior of fragments, which build up compound 16

(**A**) Ca^{2+} flux assay of **X7** (western fragment) or **X8p** (eastern fragment) as well as a mixture of both compounds (see **Figures 2, 3** for structures). HEK293 cells stably transfected with hCMKLR1-eYFP and a chimeric G protein $G\alpha_{\Delta6qi4myr}$ were stimulated with different chemerin-9 (C9) concentrations and constant 30 µM compound concentration. The control (ctl) only contains the respective amount of DMSO instead of compound. Data represent the mean ± SEM. All measurements were performed in duplicates and at least three times. (**B**) Table describes the values from results presented in A.

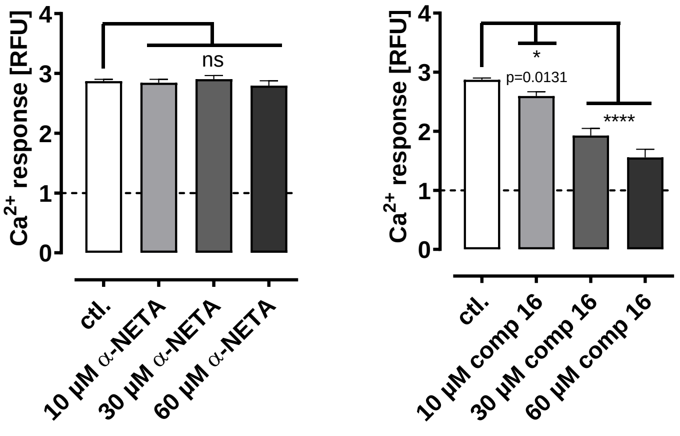

Figure S98: Comparison of the inhibitory effect of α-NETA and 16 without pre-incubation.

The Ca^{2+}-response was measured directly while applying either α-NETA or compound **16** (compared to 0.6% DMSO (ctl.)) along with 2.5 nM of the ligand chemerin-9 (EC_{50} value) to stably transfected HEK293 cells containing CMKLR1-eYFP and a chimeric G protein $G\alpha_{\Delta6qi4myr}$. The assay was conducted in triplicate at least five times. Data are presented as mean±SEM. An ANOVA with Dunnett's Multiple Comparison Test was carried out using Prism Version 10 software for statistical analysis. * $p<0.05$, **** $p<0.0001$, ns - not significant.

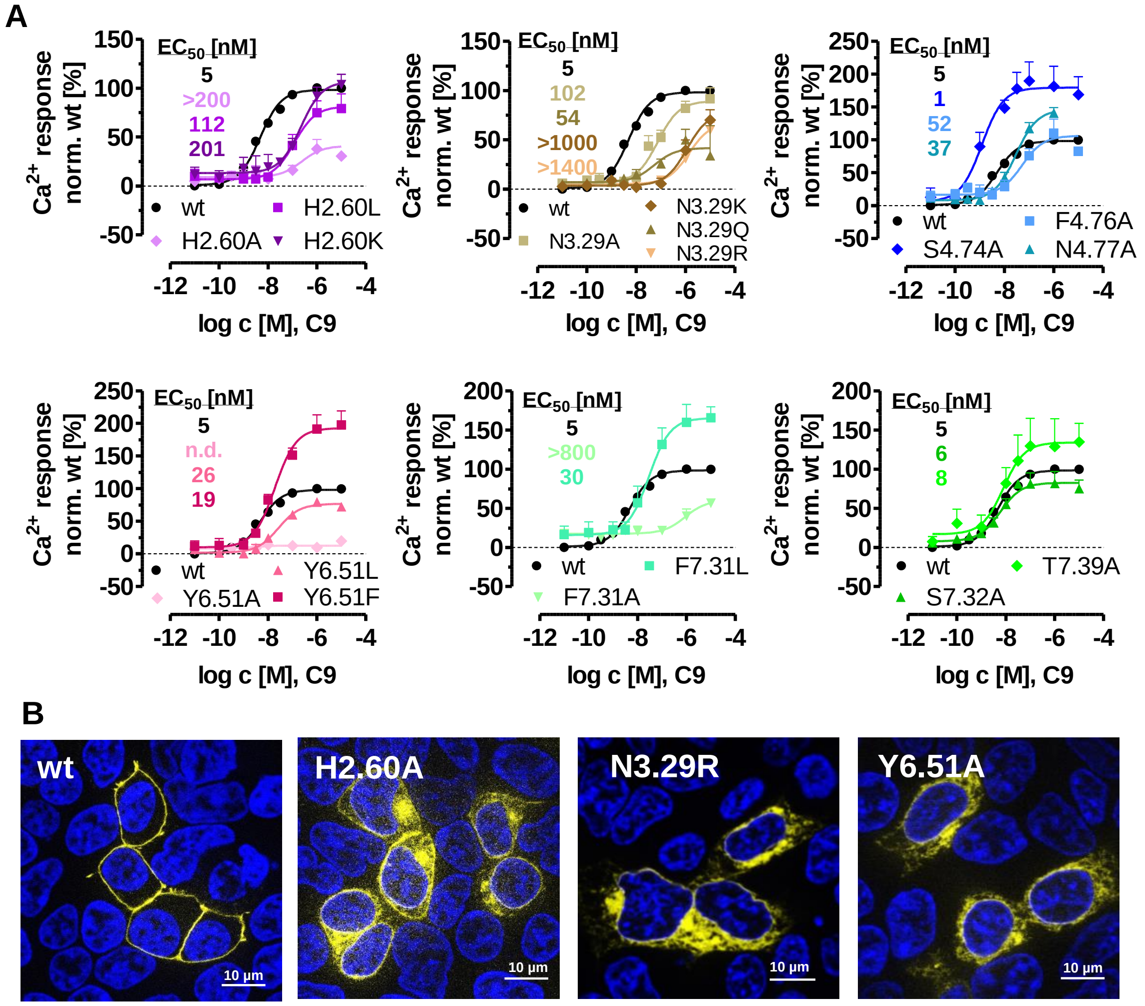

Figure S99: Ca^{2+} flux data and microscopic images of CMKLR1 variants with impaired membrane localization

(**A**) Ca^{2+} flux assay was performed with HEK293 transiently transfected with CMKLR1-eYFP variants and a chimeric G protein $G\alpha_{\Delta 6qi4myr}$. Cells were incubated with different concentrations of chemerin-9. The maximal Ca^{2+} response was determined relative to the basal value, and results were conducted to fit using the log(agonist) vs. response (three parameters) model in Prism version 5.03. (**B**) Receptor localization of receptor variants which are not expressed in the membrane (H2.60A (residue 95), N3.29R (residue 116) and Y6.51A (residue 276)) and therefore not stimulated with compound **16** and C9 for double concentration-response curve. (see **Figure 6**). HEK293 cells were transiently transfected with 1000 ng receptor-eYFP variants (yellow fluorescence) in µ-slide 8 Well (Ibidi). Cell nuclei were stained with Hoechst33342 (blue fluorescence). Scale bar 10 µm.

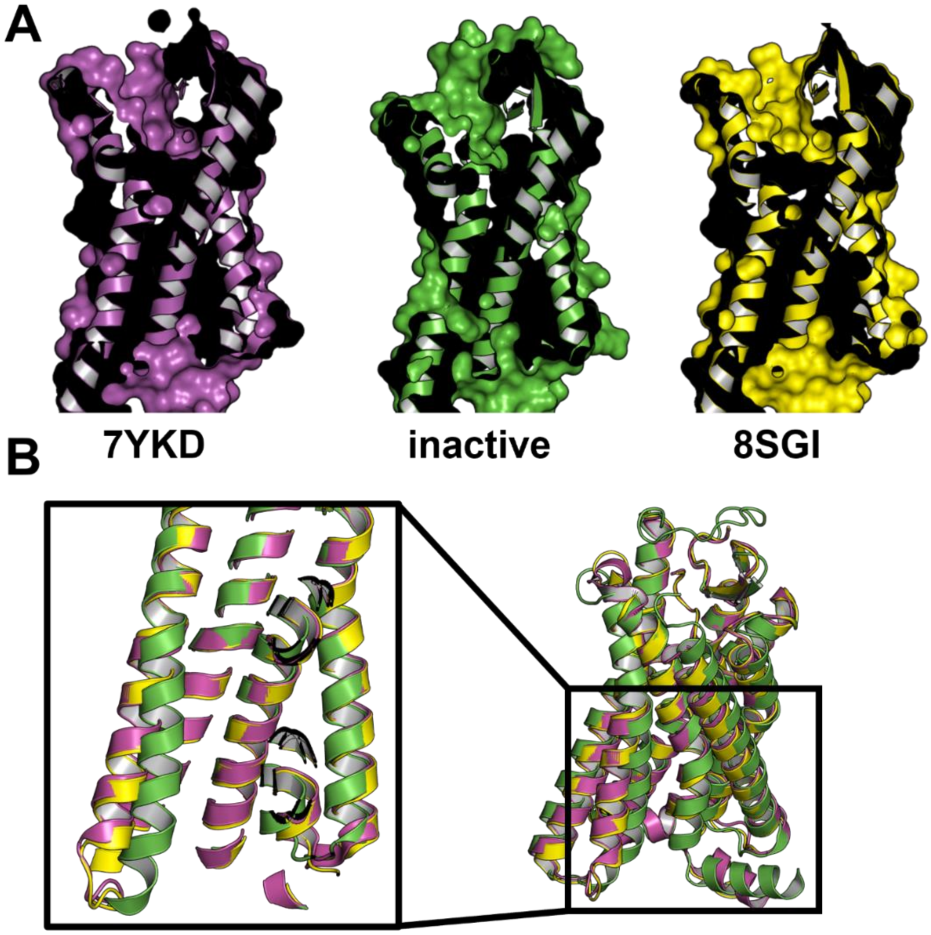

Figure S100: Comparison of modeled inactive conformation of CMKLR1 to experimentally determined active conformation

(**A**) All three structures exhibit an orthosteric binding pocket towards the extracellular area. The pocket geometries of the two experimentally determined structures, 7YKD (purple) and 8SG1 (yellow), are similar, while the inactive AlphaFold2 model (green) is slightly smaller. (**B**) The active and inactive structures overlay shows a minor deviation in both receptors. The main difference is observed in the orientation of TM6. The backbone RMSD value is around 2 Å.

Table S1. Analytical data and sequence of peptides

Peptide	Sequence	MW_{obs} [m/z]	MW_{mono} [g/mol]	Purity[a] (HPLC)
C9[b]	YFPGQFAFS	1063.56	1062.48	>98%[1,2]
TAMRA-C9[b]	TAMRA-YFPGQFAFS	1475.65	1474.62	>98%[1,2]
pNPY	YPSKPDNPGEDAPAED-LARYYSALRHYIN-LITRQRY	4252.10	4251.12	>98%[1,2]

[a]The purity was investigated by using two different HPLC columns: [1]Phenomenex Jupiter Peptide Proteo C-12, 250×4.6 mm, 90 Å, 4 µm; [2]Phenomenex Aeris Peptide XB-C18, 250×4.6 mm, 100 Å, 3.6 µm. Molecular weight was detected by MALDI-MS. C9 - chemerin-9, MW - molecular weight, mono - monoisotopic, obs – observed. [b]Peptides have been described before.[14,15]

Supplementary: Protocol Capture Molecular Modeling

3. Generation of energetical minimized CMKLR1 and GPR1 structures

Structural models of the inactive state structures of both CMKLR1 and GPR1 were generated with AlphaFold2 by Heo and Feig [1] and the prepared models downloaded from GPCRdb [2]. Later, the model was cut to the transmembrane area and helix 8 according to the proposed structure by GPCRdb [2]. The models were visual inspected and energetically minimized with the standard Rosetta membrane relax function [3]. A span file was generated using the TOPCONS web server (https://topcons.net/) with the utilized respective sequence [4]:

3.1 Sequence for CMKLR1 and GPR1 models

```
-------------------------------
(later excluded part of the protein written in brackets [])
>CMKLR1(https://www.uniprot.org/uniprotkb/Q99788/entry):
[MRMEDEDYNTSISYGDEYPDYLDSIVVL]
EDLSPLEARVTRIFLVVVYSIVCFLGILGNGLVIIIATFKMKKTVNMVWFLNLAVADFLF
NVFLPIHITYAAMDYHWVFGTAMCKISNFLLIHNMFTSVFLLTIISSDRCISVLLPVWSQ
NHRSVRLAYMACMVIWVLAFFLSSPSLVFRDTANLHGKISCFNNFSLSTPGSSSWPTHSQ
MDPVGYSRHMVVTVTRFLCGFLVPVLIITACYLTIVCKLQRNRLAKTKKPFKIIVTIIIT
FFLCWCPYHTLNLLELHHTAMPGSVFSLGLPLATALAIANSCMNPILYVFMGQDFKKFKV
ALFSRL
[VNALSEDTGHSSYPSHRSFTKMSSMNERTSMNERETGML]
-------------------------------
>GPR1 https://www.uniprot.org/uniprotkb/P46091/entry
[MEDLEETLFEEFENYSYDLDYYSLES]
DLEEKVQLGVVHWVSLVLYCLAFVLGIPGNAIVIWFTGFKWKKTVTTLWFLNLAIADFIF
LLFLPLYISYVAMNFHWPFGIWLCKANSFTAQLNMFASVFFLTVISLDHYIHLIHPVLSH
RHRTLKNSLIVIIFIWLLASLIGGPALYFRDTVEFNNHTLCYNNFQKHDPDLTLIRHHVL
TWVKFIIGYLFPLLTMSICYLCLIFKVKKRSILISSRHFWTILVVVVAFVVCWTPYHLFS
IWELTIHHNSYSHHVMQAGIPLSTGLAFLNSCLNPILYVLISKKFQARFRSSVAEI
[LKYTLWEVSCSGTVSEQLRNSETKNLCLLETAQ]
--------------------------------
```

3.2 Options for Energy Minimization (500 generated structures in 50 runs):

```
-------------------------------
# i/o
-in:file:spanfile <CMKLR1/GPR1>.span
-in:file:s <CMKLR1/GPR1>.pdb
-out:pdb
-out:path:all output/
-nstruct 10
# membrane options
-membrane:no_interpolate_Mpair
-membrane:Menv_penalties
-rg_reweight .1
-score:weights stage3_rlx_membrane.wts
```

```
# relax options
-default_max_cycles 200
-relax:min_type lbfgs_armijo_nonmonotone
-relax:minimize_bond_angles
-relax:minimize_bond_lengths
-relax:jump_move true
-score:weights stage3_rlx_membrane.wts
-use_bicubic_interpolation
-hybridize:stage1_probability 1.0
-sog_upper_bound 15
-relax:constrain_relax_to_start_coords
-in:detect_disulf
# reduce memory footprint
-chemical:exclude_patches LowerDNA  UpperDNA Cterm_amidation
SpecialRotamer
VirtualBB ShoveBB VirtualDNAPhosphate VirtualNTerm CTermConnect
sc_orbitals
pro_hydroxylated_case1 pro_hydroxylated_case2 ser_phosphorylated
thr_phosphorylated
tyr_phosphorylated tyr_sulfated lys_dimethylated lys_monomethylated
lys_trimethylated lys_acetylated glu_carboxylated cys_acetylated
tyr_diiodinated
N_acetylated C_methylamidated MethylatedProteinCterm
-linmem_ig 10
# run multiple processes to produce output for one file
-multiple_processes_writing_to_one_directory
-------------------------------
CMKLR1.span
region predicted using Topcons
7  307
antiparallel
n2c
   14      34
   49      69
   88     108
  127     147
  194     214
  231     251
  268     288
-------------------------------
GPR1.span
region predicted using Topcons
7  297
antiparallel
n2c
   13      33
   50      70
```

```
  91     111
 128     148
 182     202
 218     238
 259     279
-------------------------------
```

Based on the built structures, the RMSD to the best model was calculated, a rmsd-total_score plot generated, and the structures investigated. It must be noted that most structures had an RMSD-value of below 2 Å and a clustering was omitted.

4. Molecular Docking with RosettaLigand

The ligand was prepared according to the standard RosettaLigand protocol with BCL [5,6] and translated to a Rosetta readable params file. The initial ligand structure was aligned in the binding pocket of CMKLR1. After the final binding pose of compound **16** in CMKLR1 was identified, this was taken as input for the starting pose in GPR1.

After generating 5*200 docking poses, the ligand RMSD to the pose with the best docking score *interface_delta_X* as binding affinity metric was calculated. The best ten poses were visualized and analyzed in PyMOL (version 2.5.4), and individual clusters were redocked in a refinement step. Here, the parameter set of the transform mover was changed to allow minimal changes, only. Both the docking score and fulfillment of the experimental results were taken into account when selecting the final docking pose.

4.1 Conformer generation with BCL

```
-------------------------------
~/bcl/build/linux64_release/bin/bcl-apps-static.exe
molecule:ConformerGenerator -conformation_comparer SymmetryRMSD 0.25
-max_iterations 2000 -top_models 100 -cluster -ensemble_filenames
cpd16.sdf      -conformers_single_file      cpd16_conf.sdf      -
explicit_aromaticity -generate_3D -add_h
python2.7
~/$Rosetta/main/source/scripts/python/public/molfile_to_params.py -n
cpd16   -p   cpd16   --conformers-in-one-file   cpd16_conf.sdf   --
extra_torsion_output
-------------------------------
```

4.2 Docking with RosettaLigand

```
-------------------------------
/$Rosetta/main/source/bin/rosetta_scripts @flags.options -s
<CMKLR1/GPR1>.pdb -in:file:extra_res_fa cpd16.params
-------------------------------
#flags.options
-out:path:all output/

#the packing options allow Rosetta to sample additional rotamers for
#protein sidechain angles chi 1 (ex1) and chi 2 (ex2)
#no_optH false tells Rosetta to optimize hydrogen placements
#flip_HNQ tells Rosetta to consider HIS,ASN,GLN hydrogen flips
```

```
#ignore_ligand_chi prevents Roseta from adding additional ligand
rotamer

-packing
     -ex1
     -ex2
     -no_optH false
     -flip_HNQ true
     -ignore_ligand_chi true

-parser
     -protocol dock.xml

#-overwrite

#Ligand docking is not yet benchmarked with the updated scoring
function
#This flag restores certain parameters to previously published
values

-mistakes
     -restore_pre_talaris_2013_behavior true

-nstruct 200
-------------------------------
#dock.xml
<ROSETTASCRIPTS>
    <SCOREFXNS>
        <ScoreFunction name="ligand_soft_rep"
weights="ligand_soft_rep">
            <Reweight scoretype="fa_elec" weight="0.42"/>
            <Reweight scoretype="hbond_bb_sc" weight="1.3"/>
            <Reweight scoretype="hbond_sc" weight="1.3"/>
            <Reweight scoretype="rama" weight="0.2"/>
        </ScoreFunction>

        <ScoreFunction name="hard_rep" weights="ligand">
            <Reweight scoretype="fa_intra_rep" weight="0.004"/>
            <Reweight scoretype="fa_elec" weight="0.42"/>
            <Reweight scoretype="hbond_bb_sc" weight="1.3"/>
            <Reweight scoretype="hbond_sc" weight="1.3"/>
            <Reweight scoretype="rama" weight="0.2"/>
        </ScoreFunction>
    </SCOREFXNS>
    <LIGAND_AREAS>
        <LigandArea name="docking_sidechain" chain="X" cutoff="6.0"
add_nbr_radius="true" all_atom_mode="true" minimize_ligand="10"/>
```

```
        <LigandArea name="final_sidechain" chain="X" cutoff="6.0"
add_nbr_radius="true" all_atom_mode="true"/>
        <LigandArea name="final_backbone" chain="X" cutoff="7.0"
add_nbr_radius="false" all_atom_mode="true"
Calpha_restraints="0.3"/>
    </LIGAND_AREAS>

    <INTERFACE_BUILDERS>
        <InterfaceBuilder name="side_chain_for_docking"
ligand_areas="docking_sidechain"/>
        <InterfaceBuilder name="side_chain_for_final"
ligand_areas="final_sidechain"/>
        <InterfaceBuilder name="backbone"
ligand_areas="final_backbone" extension_window="3"/>
    </INTERFACE_BUILDERS>

    <MOVEMAP_BUILDERS>
        <MoveMapBuilder name="docking"
sc_interface="side_chain_for_docking" minimize_water="false"/>
        <MoveMapBuilder name="final"
sc_interface="side_chain_for_final" bb_interface="backbone"
minimize_water="false"/>
    </MOVEMAP_BUILDERS>

    <SCORINGGRIDS ligand_chain="X" width="25" name="vdw">
        <ClassicGrid grid_name="classic" weight="1.0"/>
    </SCORINGGRIDS>

    <MOVERS>
        <Transform name="transform" chain="X" box_size="6.0"
move_distance="0.1" angle="20.0" cycles="500" repeats="3"
temperature="5" grid_set="vdw" initial_perturb="3.0" />
### for refinement docking: move_distance="0.04" angle="5.0"
        <HighResDocker name="high_res_docker" cycles="6"
repack_every_Nth="3" scorefxn="ligand_soft_rep"
movemap_builder="docking"/>
        <FinalMinimizer name="final" scorefxn="hard_rep"
movemap_builder="final"/>
        <InterfaceScoreCalculator name="add_scores" chains="X"
scorefxn="hard_rep" compute_grid_scores="True" grid_set="vdw"/>

        <ParsedProtocol name="low_res_dock">
            <Add mover_name="transform"/>
        </ParsedProtocol>

        <ParsedProtocol name="high_res_dock">
            <Add mover_name="high_res_docker"/>
            <Add mover_name="final"/>
        </ParsedProtocol>
```

```
        <ParsedProtocol name="reporting">
            <Add mover_name="add_scores"/>
        </ParsedProtocol>
    </MOVERS>

    <PROTOCOLS>
        <Add mover_name="low_res_dock"/>
        <Add mover_name="high_res_dock"/>
        <Add mover_name="reporting"/>
    </PROTOCOLS>
</ROSETTASCRIPTS>
-------------------------------
```

5. Molecular Docking with DiffDock

Molecular docking with the DiffDock algorithm was performed in a GoogleColab environment using NVDIA Tesla T4 GPU runtime [7]. The docking process took 20 min. A copy of the python code is provided as Jupyter Notebook file in the supplementary (based on the codes originally developed by Corso et al. [8]). In short, DiffDock begins by taking an initial ligand pose with ligand conformers generated by RDKit [9] and docks it to the provided target. This process involves searching for the docking position with the highest likelihood, utilizing 100 inference steps based on the probability distribution established in the trained DiffDock model. Additionally, the algorithm calculates both the associated confidence score and the AutoDock Vina (smina) affinity score for each docking event. Due to scoring and minimization with AutoDock Vina, an evaluation of the docking pose is possible [10]. Subsequently, a new ligand pose is generated, and the docking computations are iteratively performed for all the proposed ligand poses. The final stage involves ranking these ligand poses based on their respective confidence scores obtained from the docking process, with the smina affinity scores also being reported for each. The results were downloaded and visually inspected in PyMOL (version 2.5.4). As the sdfs do not contain hydrogens, BCL was utilized to add hydrogens. The final sdf was converted in Rosetta to params and refined with RosettaLigand.

5.1 Preparing the ranked sdfs for refinement

```
-------------------------------
/bcl/build/linux64_release/bin/bcl-apps-static.exe
molecule:Properties  -add_h  -input_filenames  <rank21/rank1>.sdf  -
output cpd16.sdf

python2.7
~/$Rosetta/main/source/scripts/python/public/molfile_to_params.py -n
cpd16    -p    cpd16    --conformers-in-one-file    cpd16.sdf    --
extra_torsion_output
-------------------------------
```

5.2 Refinement with RosettaLigand

For the refinement, the prepared structure was docked 5*200 times with the same parameter set as described before with and without "*low_res_dock*" – transform mover. The results were analyzed in the same way and refined in an additional docking step of the best results.

5.3 Refinement with Rosetta Energy Minimization

In our analysis, due to the initial low confidence scores of the top-ranked binding poses in CMKLR1, we manually reviewed all ranked results to ensure they met the experimental constraints. We selected the binding pose with the highest overlapping with experimental constraints (rank 21) for further refinement using Rosetta's energetical minimization (relax) application. As previously detailed, we converted the ranked SDF file into a params file format readable by Rosetta. This file, along with the previously prepared span file and option file, was used for the minimization process. The structure with docked compound **16** was relaxed 100 (10x10) times and rescored without ligand present. Next to the energetical top scored pose, two distinguishable structures with both preferable energy score and RMSD value to the top scoring pose and each other as individual clusters were selected. After rerunning DiffDock the confidence level increased drastically from 2.29 to 0.1.

Again, the top performing poses were used as input for a refinement with RosettaLigand as described before.

6. Molecular Docking with DynamicBind

Molecular docking with the DynamicBind algorithm was performed in a GoogleColab environment using NVDIA Tesla T4 GPU runtime [7], which took approximately 40 minutes. A copy of the python code is provided as Jupyter Notebook file in the supplementary (based on the codes originally developed by Lu et al. [11]). Like DiffDock, DynamicBind utilizes ligand conformers generated by RDKit [9] and retrieves the AlphaFold structure from the AlphaFold Protein Structure Database [12]. Throughout iterative prediction steps, the model concurrently forecasts updates for both the ligand and protein, utilizing a scoring module (contact-LDDT) to identify the most appropriate complex structure from its outputs. Finally, the highest-confidence ligand-bound state and binding pose is predicted. An affinity is predicted internally by a model trained on the PDBBind dataset [13]. Again, the downloaded results were visually inspected in PyMOL (version 2.5.4) and a refinement with conducted in RosettaLigand. Therefore, a params file was prepared with the final predicted ligand pose and the same process as described before conducted. As the sdf's do not contain hydrogens, BCL was utilized to add hydrogens. The ligand pose was extracted as sdf file and converted a to params file to be refined with RosettaLigand. Like with the DiffDock result, the prepared structure was docked 5*200 times with the same parameter set as described before with and without "*low_res_dock*" – transform mover. The results were analyzed in the same way and refined in an additional docking step of the best results.

7. Energy Breakdown

The best three docking models were selected for an energetical hotspot analysis with Residue energy breakdown:

```
-------------------------------
/$Rosetta/main/source/bin/residue_energy_breakdown.default.linuxgcc
release -s *.pdb -out:file:silent data.out -in:file:extra_res_fa
cpd16.params
-------------------------------
```

A following python script was utilized to investigate the output and select the per residue energy contributions relevant for the peptide and generating a contact map:

```
-------------------------------
```

```
import pandas as pd
import seaborn as sns
import matplotlib.pyplot as plt
import numpy as np

assign_CMKLR1 = {'19_TYR' : 'Y1.39',
'60_PHE' : 'F2.53','61_PHE' : 'F2.56',
'64_LEU' : 'L2.57','67_HIS' : 'H2.60',
'68_ILE' : 'I2.61','70_TYR' : 'Y2.63',
'71_ALA' : 'A2.64','74_ASP' : 'D2.67',
'75_TYR' : 'Y2.68','76_HIS' : 'H2ECL1',
'77_TRP' : 'W2ELC1','12_ARG' : 'R1.32',
'84_CYS' : 'C3.25','85_LYS' : 'K3.26',
'87_SER' : 'S3.28','88_ASN' : 'N3.29',
'89_PHE' : 'F3.30','91_LEU' : 'L3.32',
'92_ILE' : 'I3.33','95_MET' : 'M3.36',
'142_LEU' : 'L4.56', '153_ALA' : 'A4.67',
'143_SER' : 'S4.57','145_PRO' : 'P4.59',
'146_SER' : 'S4.60','149_PHE' : 'R4.63',
'150_ARG' : 'R4.64','151_ASP' : 'D4.65',
'154_ASN' : 'N4.68','155_LEU' : 'L4.69',
'159_ILE' : 'I4.73','160_SER' : 'S4.74',
'161_CYS' : 'C4.75','162_PHE' : 'F4.76',
'163_ASN' : 'N4.77','164_ASN' : 'N4.78',
'165_PHE' : 'F4.79','166_SER' : 'S4.80',
'167_THR' : 'T4.81','168_PRO' : 'P4.82',
'175_TRP' : 'W203', '178_HIS' : 'H5.24',
'182_ASP' : 'D5.28','185_GLY' : 'G5.31',
'186_TYR' : 'Y5.32','189_HIS' : 'H5.35',
'192_VAL' : 'V5.38', '197_PHE' : 'F5.43',
'193_THR' : 'T5.39','196_ARG' : 'R5.42',
'245_TRP' : 'W6.48','248_TYR' : 'Y6.51',
'249_HIS' : 'H6.52','251_LEU' : 'L6.54',
'252_ASN' : 'N6.55','255_GLU' : 'E6.58',
'256_LEU' : 'L6.59','257_HIS' : 'H6.60',
'258_HIS' : 'H6.61',
'259_THR' : 'T6ECL3','261_MET' : 'M6ECL3',
'264_SER' : 'S7.29','266_PHE' : 'F7.31',
'267_SER' : 'S7.32','268_LEU' : 'L7.33',
'269_GLY' : 'G7.34','270_LEU' : 'L7.35',
'271_PRO' : 'P7.36','273_ALA' : 'A7.38',
'274_THR' : 'T7.39', '96_PHE' : 'F3.37'}

assign_GPR1 = {'19_TYR' : 'Y1.39',
'67_TYR' : 'Y2.60', '70_TYR' : 'Y2.63',
'71_VAL' : 'V2.64', '75_PHE' : 'F101',
```

```
'88_SER' : 'S3.29', '92_GLN' : 'N3.35',
'150_ARG' : 'R4.64', '162_TYR' : 'Y4.76',
'163_ASN' : 'N4.77', '177_HIS' : 'H5.35',
'184_LYS' : 'K5.42', '236_TYR' : 'Y6.51',
'240_SER' : 'S6.55', '243_GLU' : 'E6.58',
'256_MET' : 'M7.31', '257_GLN' : 'Q7.32',
'260_ILE' : 'I7.35', '261_PRO' : 'P7.36',
'264_THR' : 'T7.39'
         }
def df_prep_bw(file, chain):
    df = pd.read_csv(file, sep='\s+')
    df = df.loc[(df['pdbid2'] != '--') & (df['pdbid2'] ==
chain)]
    df = df[['resi1', 'restype1', 'total']]
    df['ori_df'] =
df['resi1'].astype(str).str.cat(df['restype1'].astype(str),
sep='_')
    df['model'] = file[:-13]
    df = df.loc[(df['total'] > 0.1) | (df['total'] < -0.1)]
    df['BW'] = df['ori_df'].map(lambda x: assign_CMKLR1[x])
    # for GPR1 this was changed accordingly
    df['TM'] = df['BW'].map(lambda x: x[1])
    return df
chain = "1X"
df1  = df_prep_bw('data.out', chain)
fig, ax = plt.subplots(figsize=(6,12))
sns.barplot(x = 'total', y = 'BW', data = df1)
plt.title("Energy breakdown per residues <CMKLR1/GPR1>",
fontsize=10)
ax.set_ylabel('CMKLR1 residues', fontsize=10)
ax.set_xlabel('Score [REU]', fontsize=10)
title = 'CMKLR1_best_BW.svg'
print(title)
fig.savefig(title, dpi=300)
-------------------------------
```

This energy breakdown together with the binding pose visualized in PyMOL (version 2.5.4) was used to determine the results with the highest consensus with the experimental data.

8. Bibliography

(1) Heo L, Feig M. Multi-state modeling of G-protein coupled receptors at experimental accuracy. Proteins. 2022, 90(11), 1873–85. DOI:10.1002/prot.26382

(2) Pándy-Szekeres G, Caroli J, Mamyrbekov A, Kermani AA, Keserű GM, Kooistra AJ, et al. GPCRdb in 2023: state-specific structure models using AlphaFold2 and new ligand resources. Nucleic Acids Res. 2023, 51(D1), D395–402. DOI:10.1093/nar/gkac1013

(3) Leman JK, Weitzner BD, Lewis SM, Adolf-Bryfogle J, Alam N, Alford RF, et al. Macromolecular modeling and design in Rosetta: recent methods and frameworks. Nat Methods. 2020, 17(7), 665–80. DOI:10.1038/s41592-020-0848-2

(4) Tsirigos KD, Peters C, Shu N, Käll L, Elofsson A. The TOPCONS web server for consensus prediction of membrane protein topology and signal peptides. Nucleic Acids Res. 2015, 43(W1), W401-7. DOI:10.1093/nar/gkv485
(5) Kothiwale S, Mendenhall JL, Meiler J. BCL::Conf: Small molecule conformational sampling using a knowledge based rotamer library. J Cheminform. 2015, 7(1), 1–15. DOI:10.1186/s13321-015-0095-1
(6) Mendenhall J, Brown BP, Kothiwale S, Meiler J. BCL::Conf: Improved Open-Source Knowledge Based Sampling Using the Crystallography Open Database. Physiol Behav. 2021, 176(5), 139–48. DOI:10.1021/acs.jcim.0c01140.BCL
(7) Google. Welcome to Colaboratory! (2018). https://colab.research.google.com.
(8) Corso G, Stärk H, Jing B, Barzilay R, Jaakkola T. DiffDock: Diffusion Steps, Twists, and Turns for Molecular Docking. In: International Conference on Learning Representations (ICLR) (ICIR, 2023) [Internet]. 2022, 1–33. DOI:ArXiv:2210.01776
(9) RDKit: Open-source cheminformatics. 2021 https://www.rdkit.org doi:10.5281/zenodo.4639764.
(10) Trott O, Olson AJ. AutoDock Vina: Improving the speed and accuracy of docking with a new scoring function, efficient optimization, and multithreading. J Comput Chem. 2009, 31(2), 455–61. DOI:10.1002/jcc.21334
(11) Lu W, Zhang J, Huang W, Zhang Z, Jia X, Wang Z, et al. DynamicBind: predicting ligand-specific protein-ligand complex structure with a deep equivariant generative model. Nat Commun [Internet]. 2024, 15(1), 1071. DOI:10.1038/s41467-024-45461-2
(12) Varadi M, Anyango S, Deshpande M, Nair S, Natassia C, Yordanova G, et al. AlphaFold Protein Structure Database: Massively expanding the structural coverage of protein-sequence space with high-accuracy models. Nucleic Acids Res. 2022, 50(D1), D439–44. DOI:10.1093/nar/gkab1061
(13) Wang R, Fang X, Lu Y, Yang CY, Wang S. The PDBbind Database: Methodologies and Updates. J Med Chem [Internet]. 2005, 48(12), 4111–9. DOI:10.1021/jm048957q
(14) Czerniak AS, Kretschmer K, Weiß T, Beck-Sickinger AG. The chemerin receptor CMKLR1 requires full-length chemerin for high affinity in contrast to GPR1 as demonstrated by a new nanoluciferase-based binding assay. ChemMedChem. 2022, 17, e202200413. DOI:10.1002/cmdc.202200413
(15) Zhang X, Weiß T, Cheng MH, Chen S, Ambrosius CK, Czerniak AS, Li K, Feng M, Bahar I, Beck-Sickinger AG, Zhang C. Structural basis of G protein-coupled receptor CMKLR1 activation and signaling induced by a chemerin-derived agonist. *PLoS Biol.* **2023**, 21, e3002188. DOI: 10.1371/journal.pbio.3002188
